# Supplementary material for: Investigation of the Compatibility between Warheads and Peptidomimetic Sequences of Protease Inhibitors—A Comprehensive Reactivity and Selectivity Study
Source: Int J Mol Sci. 2023 Apr 13;24(8):7226. doi: 10.3390/ijms24087226 (PMC10138721; doi:10.3390/ijms24087226)
Supplement: Supplementary file 1 [file ijms-24-07226-s001.zip › ijms-2333892-supplementary.pdf]

Supporting information

Investigation of the compatibility between warheads and peptidomimetic sequences of protease inhibitors - a comprehensive reactivity and selectivity study

Patrick Müller[1†], Mergim Meta[1†], J. Laurenz Meidner[1†], Marvin Schwickert[1], Jessica Meyr[2], Kevin Schwickert[1], Christian Kersten[1], Collin Zimmer[1], Stefan Hammerschmidt[1], Ariane Frey[1], Albin Lahu[1], Sergio de la Hoz-Rodríguez[3], Laura Agost-Beltrán[3], Santiago Rodríguez[3], Kira Diemer[2], Wilhelm Neumann[2], Florenci V. Gonzàlez[3], Bernd Engels[2] and Tanja Schirmeister[1*]

[1] Institute of Pharmaceutical and Biomedical Sciences, Johannes Gutenberg University Mainz, Mainz, Germany

[2] Institute of Physical and Theoretical Chemistry, Julius-Maximilians-University of Wuerzburg, Wuerzburg, Germany

[3] Departament de Química Inorgànica I Orgànica, Universitat Jaume I, 12080 Castello, Spain

[*] Author to whom correspondence should be addressed.

[†] These authors contributed equally to this work.

Content

***In vitro* results of the synthesized compounds**…………………………………………… S2–4

Reactivity assay……………………………………………………………………... S5–20

Fluorometric inhibition assays……………………………………………………. S21–23

Protein Expression…………………………………………………………………. S23–25

Molecular Docking…………………………………………...……………….…… S26–30

Quantum mechanics simulations / kinetics……………………………………... S31–34

Synthesis…………………………………………………………………………… S35–98

Spectra and chromatograms………….....……………………………………….…… S99–172

References…………………………………………………………….…….…… S173–174

In vitro results of the synthesized compounds

Table S1. *In vitro* results of the tested compounds against the corresponding protease. The peptidic sequence targeting the protease is shown on top.

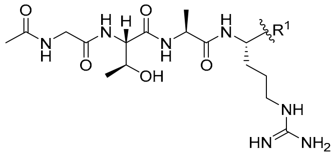

R^1	uPA	Proteasome *β*5-subunit	CatS	M^{Pro}	Rhodesain
	K_i (µM)/ k_{inact} (s^{-1})/ k_{2nd} (M^{-1} s^{-1})				
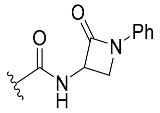 **103**	0.141 ± 0.027 a)	n.a.	n.a.	n.a.	n.a.
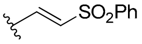 **106**	n.a.	n.a.	n.a.	n.a.	n.a.
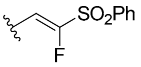 **107**	n.a.	n.a.	n.a.	n.a.	n.a.

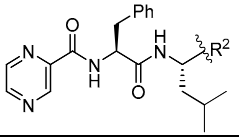

R^2	uPA	Proteasome *β*5-subunit	CatS	M^{Pro}	Rhodesain
	K_i (µM)/ k_{inact} (s^{-1})/ k_{2nd} (M^{-1} s^{-1})				
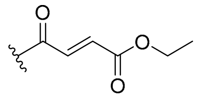 **68**	n.a.	n.a.	n.a.*	n.a.	0.009 ± 0.001/ 0.006 ± 0.001/ 6.65 x 10^5 ± 1.57 x10^4
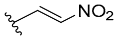 **59**	n.a.	n.a.	0.619 ± 0.054 a)	n.a.	0.002 ± 0.005 a)
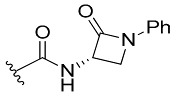 **72**	n.a.	n.a.	n.a.	n.a.	n.a.
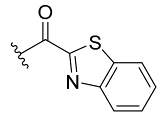 **62**	n.a.	n.a.	n.a.	n.a.	n.a.
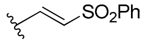 **58**	n.a.	n.a.	0.126 ± 0.071/ 0.013 ± 0.002/ 1.00 x 10^5 ± 1.25 x10^4	n.a.	0.331 ± 0.041/ 0.019 ± 0.001/ 5.85 x 10^4 ± 1.66 x10^3
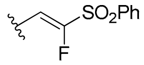 **55**	n.a.	n.a.	0.934 ± 0.138 a)	n.a.	0.462 ± 0.039 a)
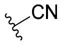 **74**	n.a.	n.a.	0.238 ± 0.023a)	n.a.	0.560 ± 0.267

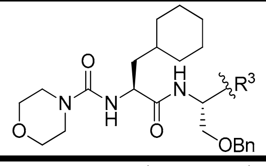

K_i (μM)/ k_{inact} (s^{-1})/ k_{2nd} (M^{-1} s^{-1})

R³	uPA	Proteasome *β*5-subunit	CatS	M^Pro	Rhodesain
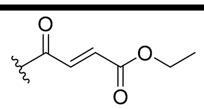 **40**	n.a.	n.a.	0.022 ± 0.004/ 0.0023 ± 0.00007/ 1.04 x 10^5 ± 1.04 x10^4	n.a.	0.035 ± 0.009/ 0.003 ± 0.0001/ 1.03 x 10^5 ± 1.09 x10^4
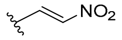 **49**	n.a.	n.a.	0.012 ± 0.001 [a)]	n.a.	0.002 ± 0.0002 [a)]
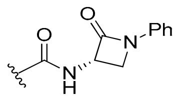 **45**	n.a.	n.a.	0.86 ± 0.13/ 0.00046 ± 0.00002/ 5.34 x 10^5 ± 8.29 x10^4	n.a.	n.a.
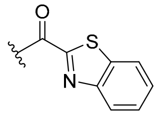 **42**	n.a.	n.a.	0.233 ± 0.024 [a)]	n.a.	0.324 ± 0.089 [a)]
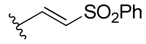 **35**	n.a.	n.a.	0.003 ± 0.0006/ 0.02022 ± 0.0006/ 7.24 x 10^6 ± 2.36 x10^5	n.a.	0.022 ± 0.002/ 0.006 ± 0.0004/ 2.57 x 10^5 ± 1.04 x10^3
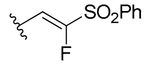 **36**	n.a.	n.a.	3.215 ± 0.612 [a)]	n.a.	n.a.
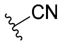 **30**	n.a.	n.a.	0.001 ± 0.0001 [a)]	n.a.	0.021 ± 0.002

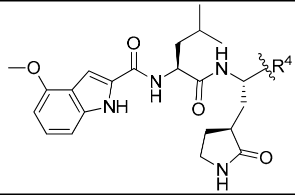

K_i (μM)/ k_{inact} (s^{-1})/ k_{2nd} (M^{-1} s^{-1})

R⁴	uPA	Proteasome *β*5-subunit	CatS	M^pro	Rhodesain
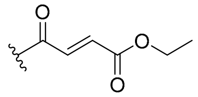 **81**	n.a.	-	n.a*	n.a*	0.099 ± 0.024/ 0.006 ± 0.0009/ 6.41 x 10^4 ± 3.00 x10^3-
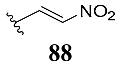 **88**	n.a.	n.a.	0.388 ± 0.277 [a)]	2.197 ± 0.184 a)	0.002 ± 0.0001 [a)]
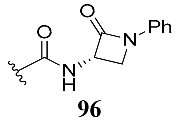 **96**	n.a.	n.a.	n.a.	n.a.	n.a.
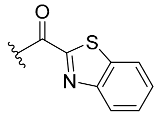 **90**	n.a.	n.a.	n.a.	0.026 ± 0.003 a)	n.a.
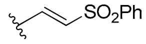 **93**	n.a.	n.a.	0.330 ± 0.065/ 0.0087 ± 0.0001/ 2.62 x 10^4 ± 2.41 x10^3	3.531 ± 1.112/ 0.0137 ± 0.0061/ 2.79 x 10^3 ± 3.12 x10^2	2.002 ± 0.604/ 0.029 ± 0.008/ 1.49 x 10^4 ± 1.09 x10^4

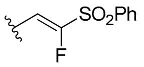 **94**	n.a.	n.a.	1.351 ± 0.09 a)	0.517 ± 0.033 a)	0.217 ± 0.031 a)
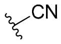 **84**	n.a.	n.a.	0.104 ± 0.009	0.031 ± 0,005 a))	0.042 ± 0.005

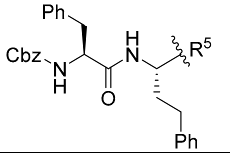

K_i (μM)/ k_{inact} (s^{-1})/ k_{2nd} (M^{-1} s^{-1})					
**R⁵**	**uPA**	**Proteasome β-5-subunit**	**CatS**	**M^Pro**	**Rhodesain**
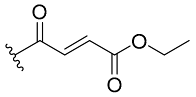 **22**	n.a.	n.a.	0.018 ± 0.003/ 0.0022 ± 0.0001/ 1.24 x 10^5 ± 1.86 x10^4	n.a.	0.079 ± 0.007/ 0.008 ± 0.001/ 1.06 x 10^5 ± 4.57 x10^2
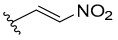 **13**	n.a.	n.a.	0.201± 0.093 a)	n.a.	0.060 ± 0.006 nM a)
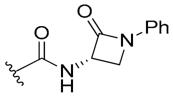 **24**	n.a.	n.a.	n.a.	n.a.	n.a.
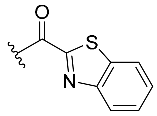 **18**	n.a.	n.a.	n.a.	n.a.	n.a.
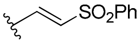 **16**	n.a.	n.a.	0.001 ± 0.0006/ 0.0037 ± 0.0006/ 2.88 x 10^6 ± 8.56 x10^5	n.a.	0.020 ± 0.002/ 0.005 ± 0.0001/ 2.90 x 10^5 ± 3.17 x10^3
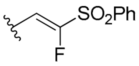 **17**	n.a.	n.a.	0.966 ± 0.104)	n.a.	3.565 ± 0.437 a)
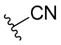 **25**	n.a.	n.a.	0.136 ± 0.012 a)	n.a.	0.088 ± 0.0174 a)

a) Calculated from IC_{50} value with the Cheng–Prusoff equation.
n.d. not determined.
n.a. not active.

Reactivity assay

Method A (^{1}H-NMR):

The corresponding Michael-acceptor (0.07 mmol, 1 eq) and the reference compound 1,3-dioxolan (0.5 eq) were dissolved in DMSO-d_6 (0.5 mL). The reaction was measured in time intervals before (0 min) and 5, 30, 60, 120 and 240 mins after the addition of the corresponding nucleophile (PhEtSH/EtONa, 2 eq) in presence or absence of Et_3N (2 eq). The CH_2-signal of 1,3-dioxlan was set at 5.3 ppm to get uniform shifts of the olefin signals in the ^{1}H-NMR spectra.

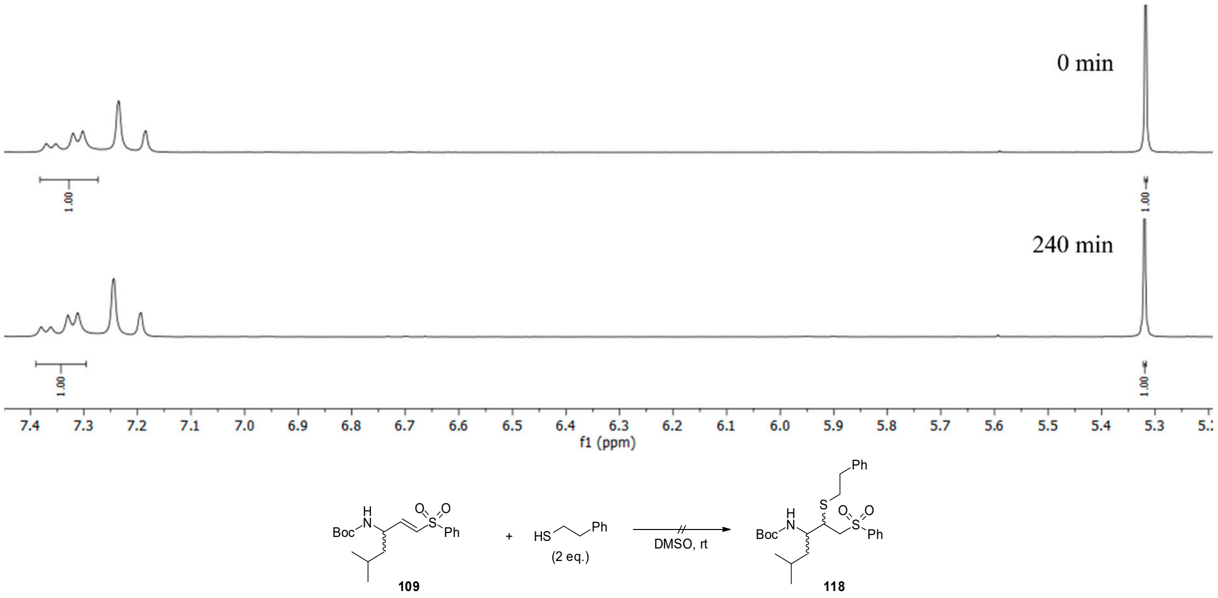

Figure S1. ^{1}H-NMR spectra of **109** after certain time intervals. Reaction of 2-phenylethanthiol with the vinyl sulfone moiety.

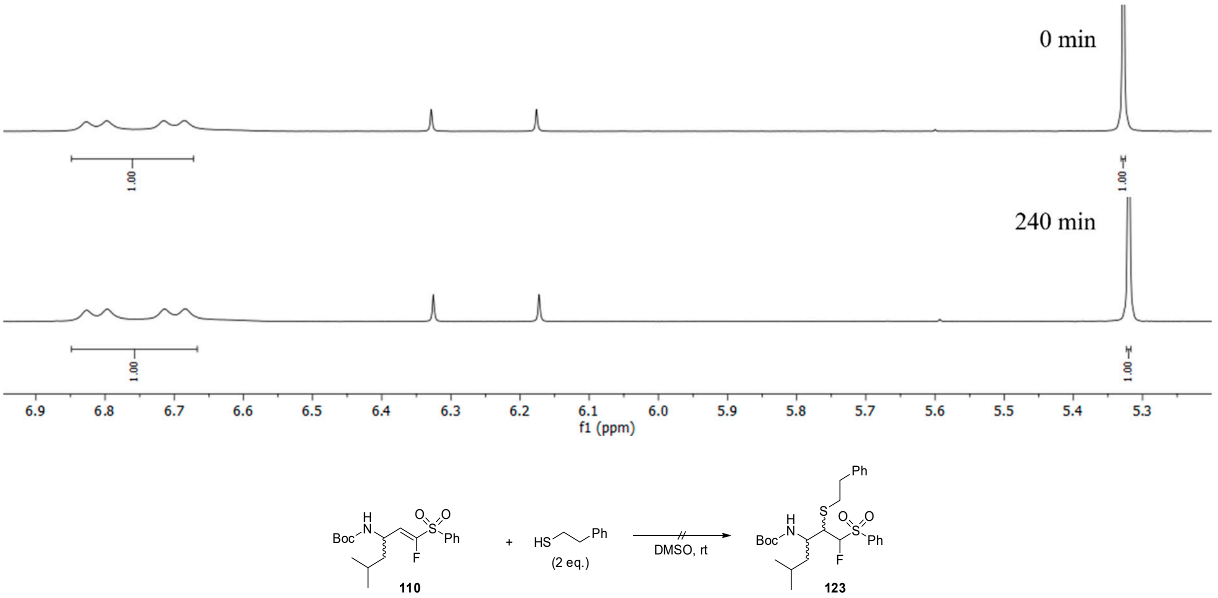

Figure S2. ^{1}H-NMR spectra of **110** after certain time intervals. Reaction of 2-phenylethanthiol with the F-vinyl sulfone moiety.

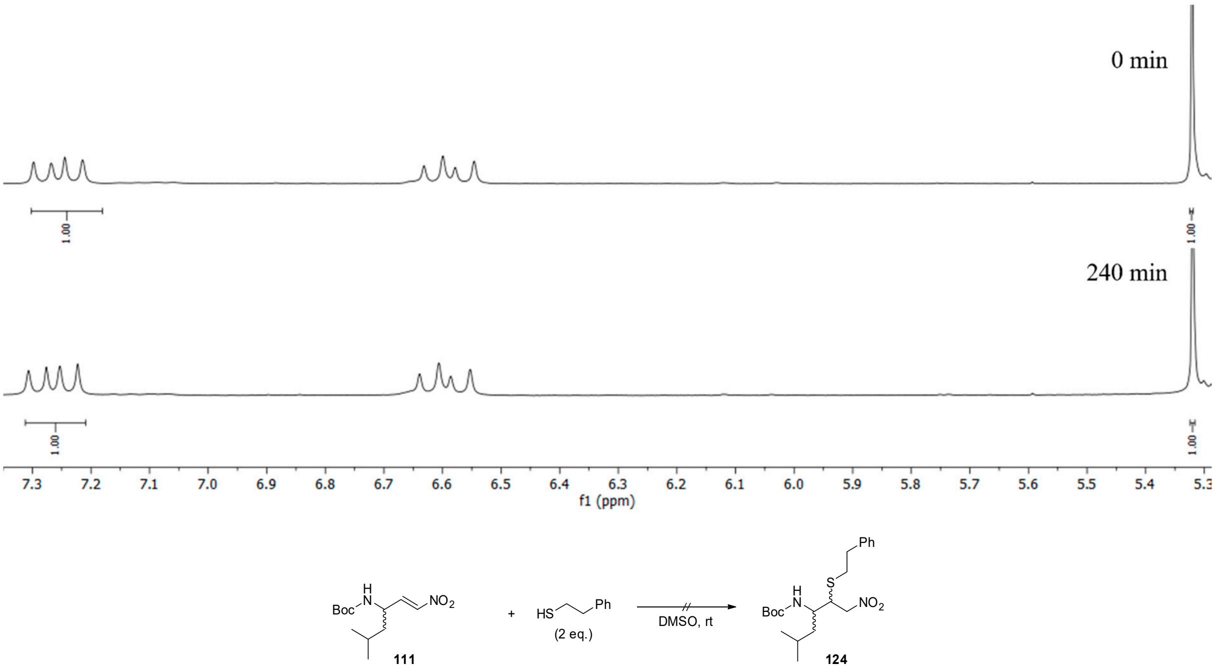

Figure S3. ^{1}H-NMR spectra of **111** after certain time intervals. Reaction of 2-phenylethanthiol with the nitroalkene moiety.

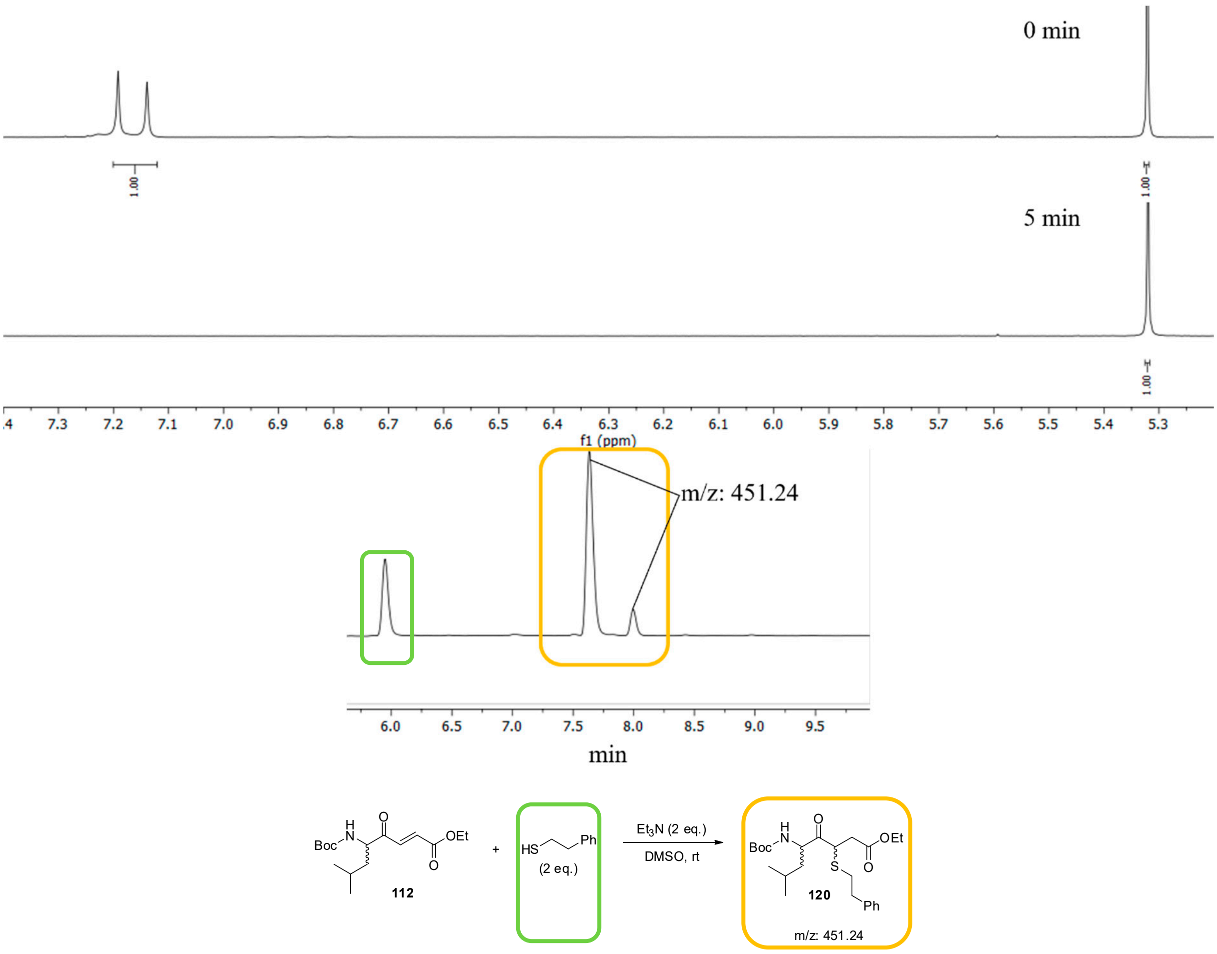

Figure S4. ^{1}H-NMR spectra of **112** after certain time intervals. Reaction of 2-phenylethanthiol with the 4-oxoenoate moiety in presence of Et_3N. LC/MS analysis of the same reaction at 10 min.

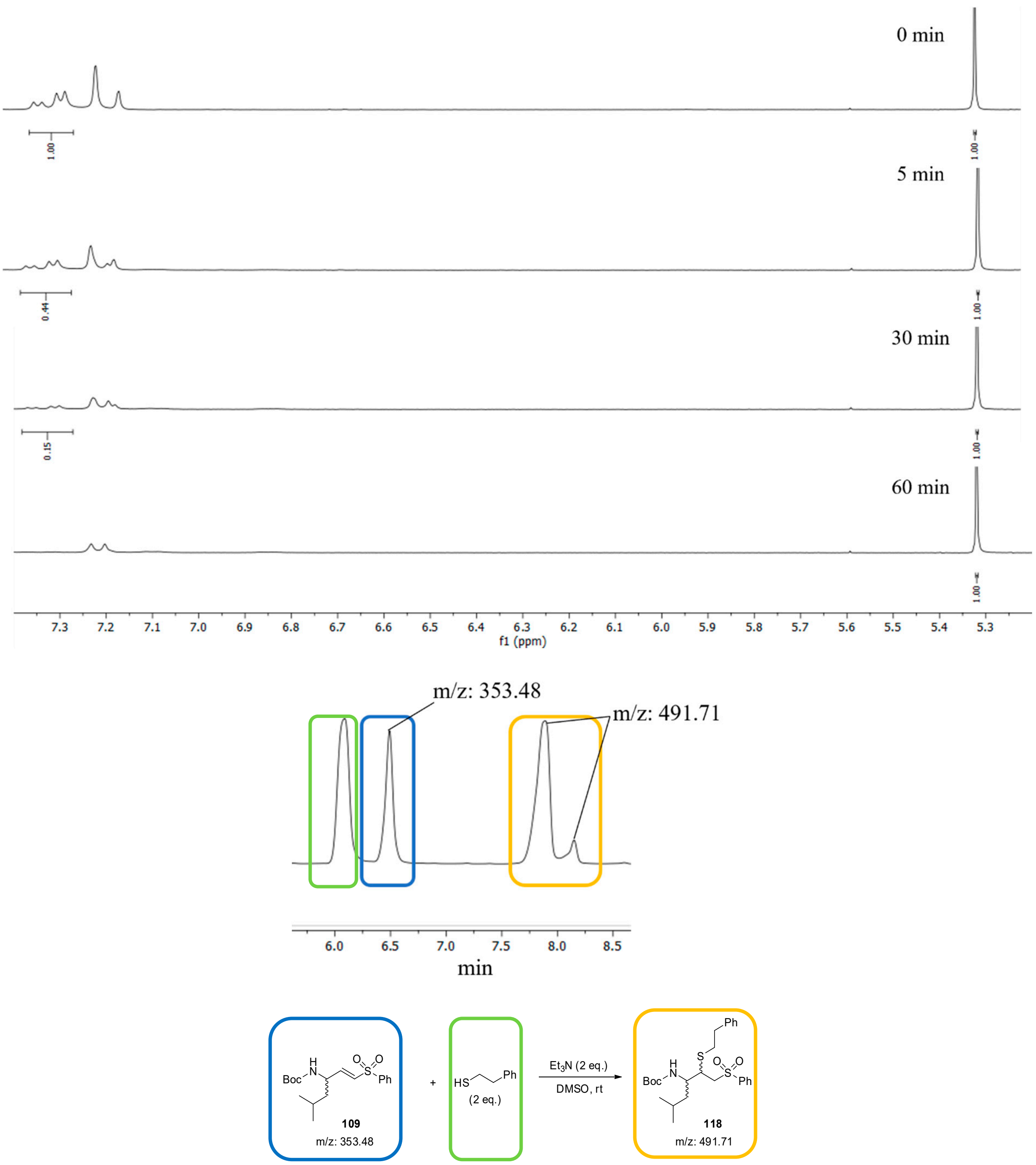

Figure S5. ^{1}H-NMR spectra of **119** after certain time intervals. Reaction of 2-phenylethanthiol with the vinyl sulfone moiety in presence of Et_3N. LC/MS analysis of the same reaction at 10 min.

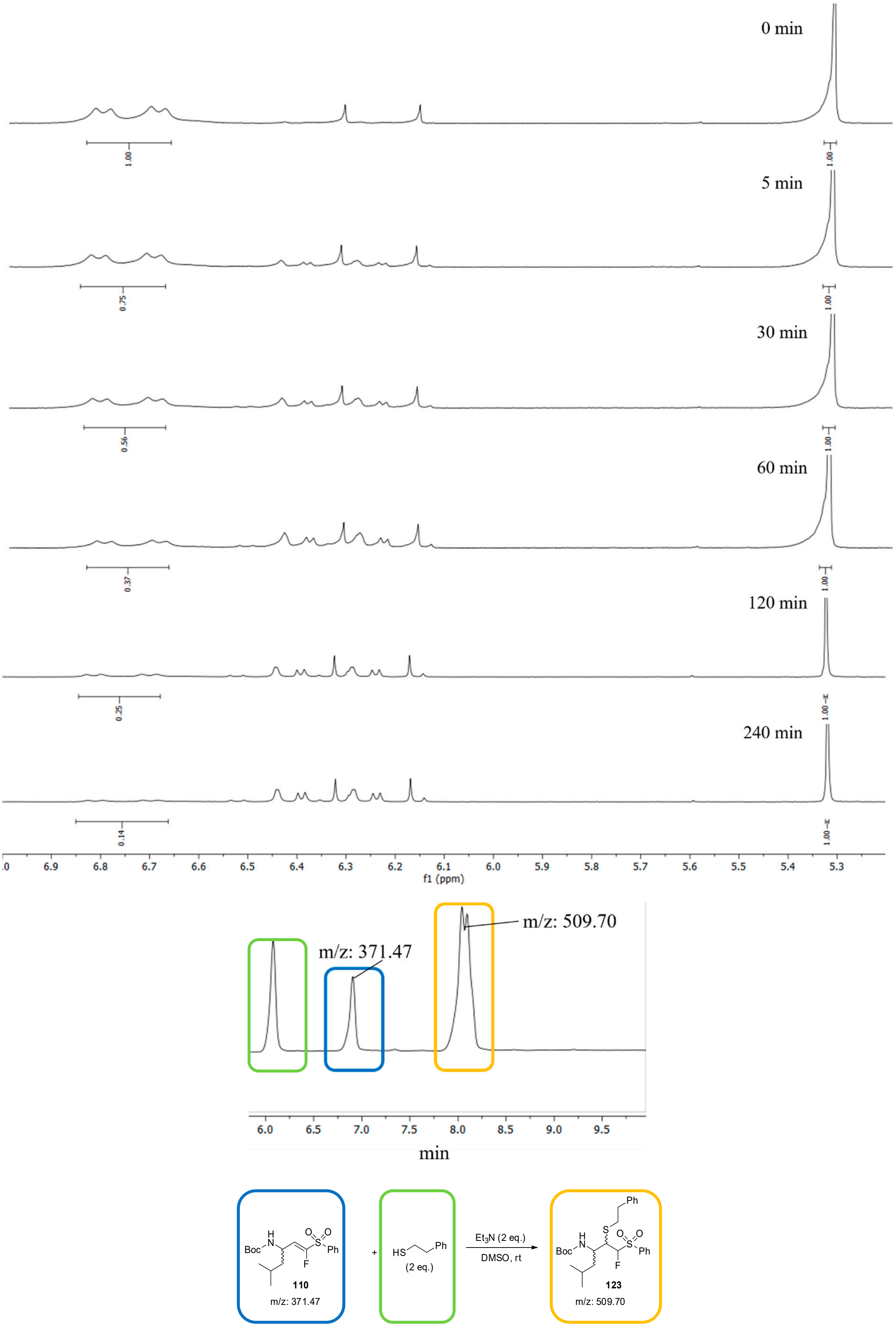

Figure S6. ^{1}H-NMR spectra of **110** after certain time intervals. Reaction of 2-phenylethanthiol with the F-vinyl sulfone moiety in presence of Et_3N. LC/MS analysis of the same reaction at 120 min.

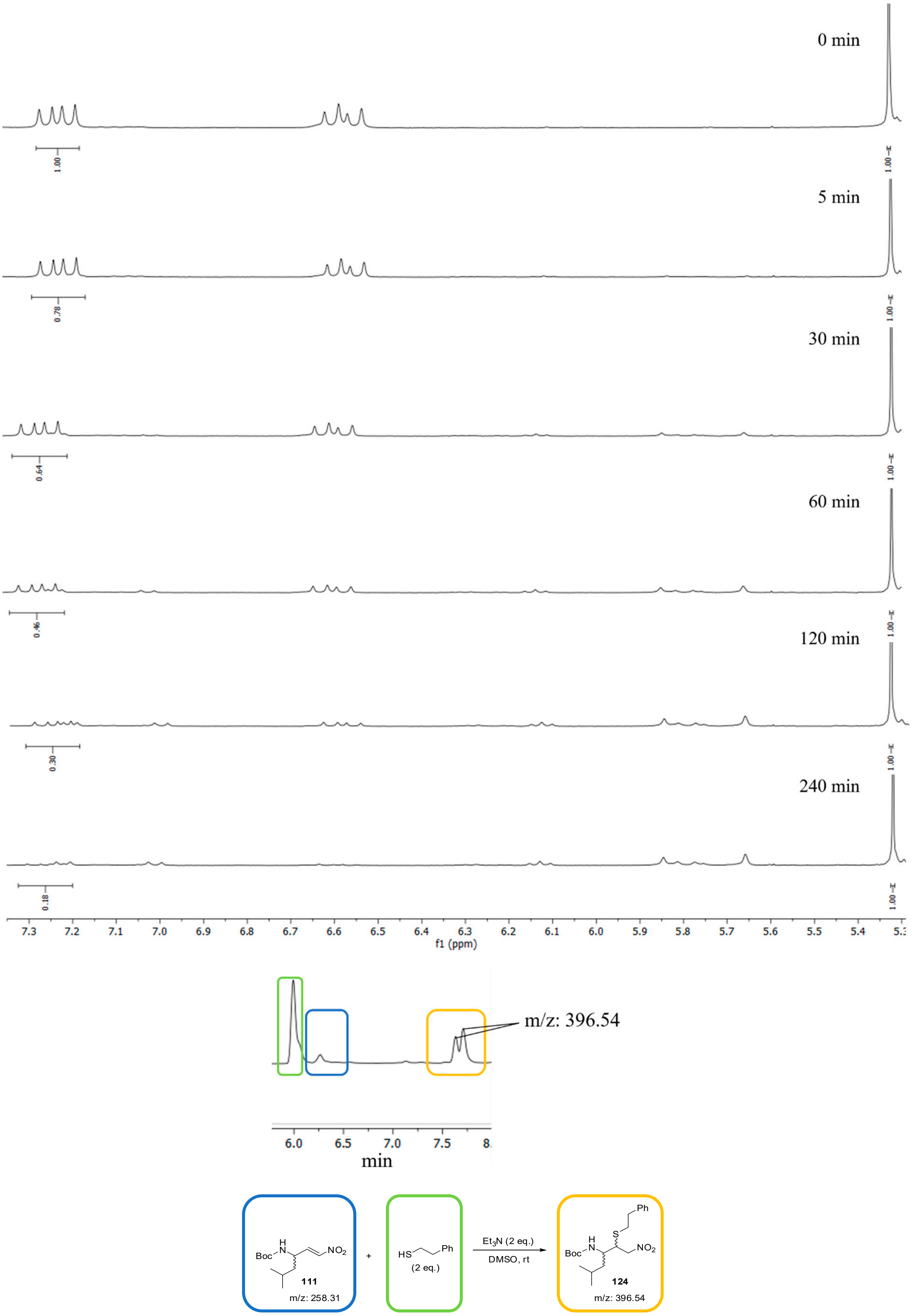

Figure S7. ^{1}H-NMR spectra of **111** after certain time intervals. Reaction of 2-phenylethanthiol with the nitroalkene moiety in presence of Et_3N. LC-MS analysis of the same reaction at 240 min. The blue marked peak was identified by NMR analysis as **111**.

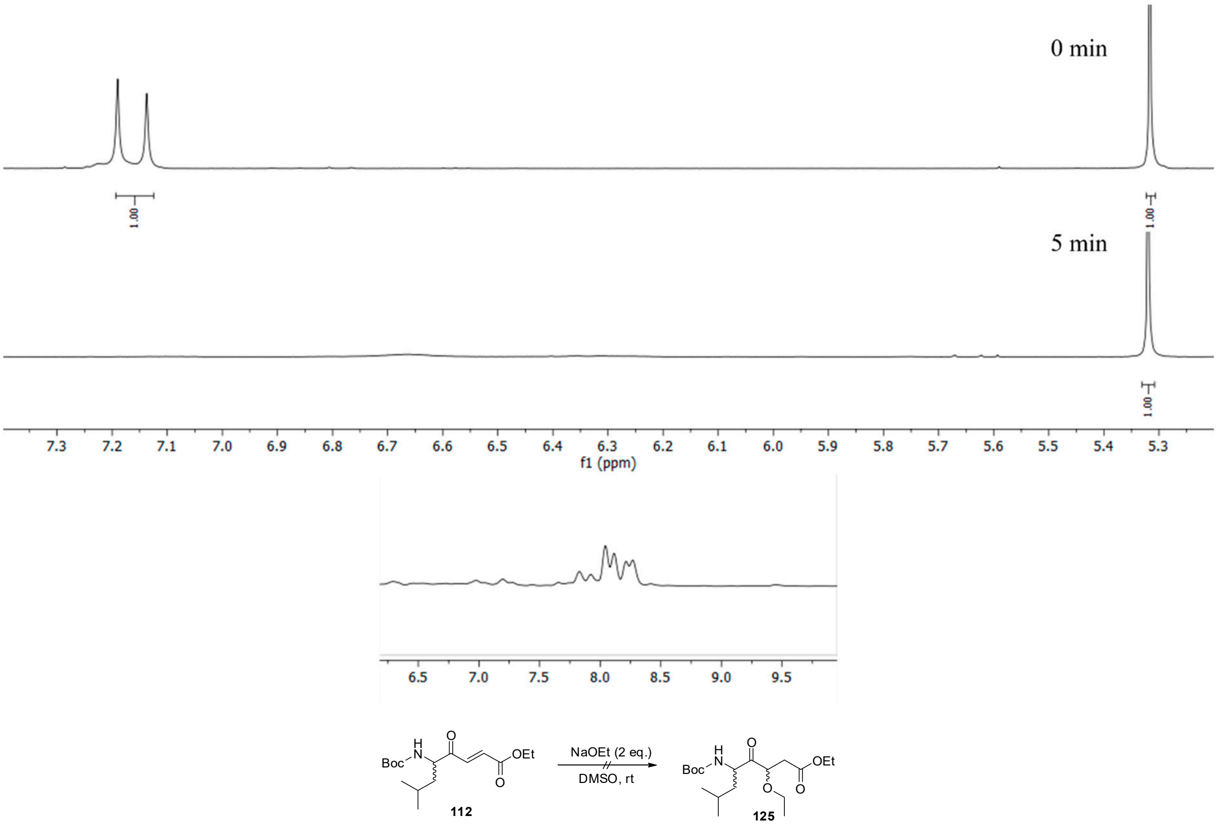

Figure S8. ^{1}H-NMR spectra of **112** after certain time intervals. Reaction of sodium ethanolate with the oxoenoate moiety. LC/MS analysis of the same reaction at 10 min.

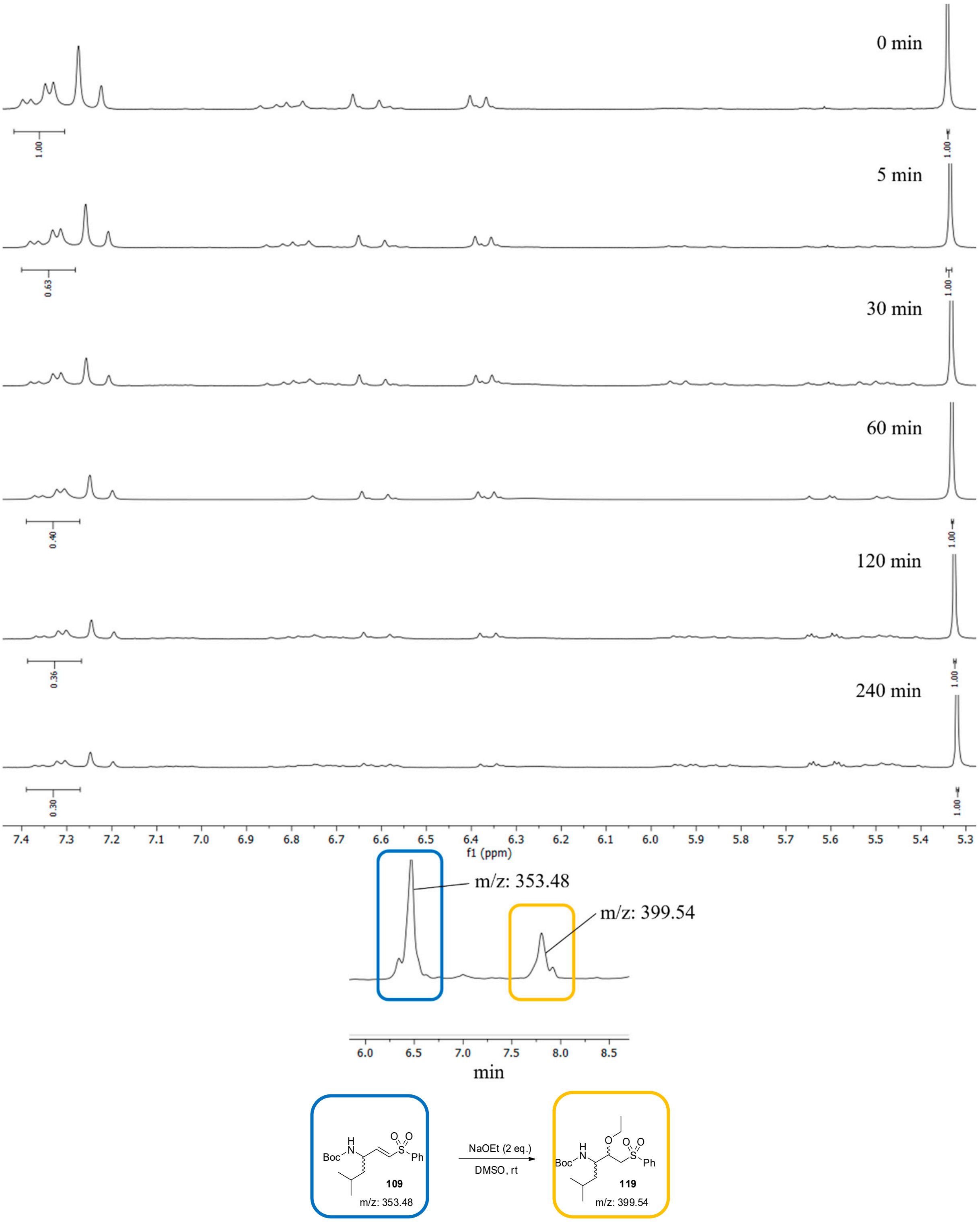

Figure S9. ^{1}H-NMR spectra of **109** after certain time intervals. Reaction of sodium ethanolate with the vinyl sulfone moiety. LC/MS analysis of the same reaction at 10 min.

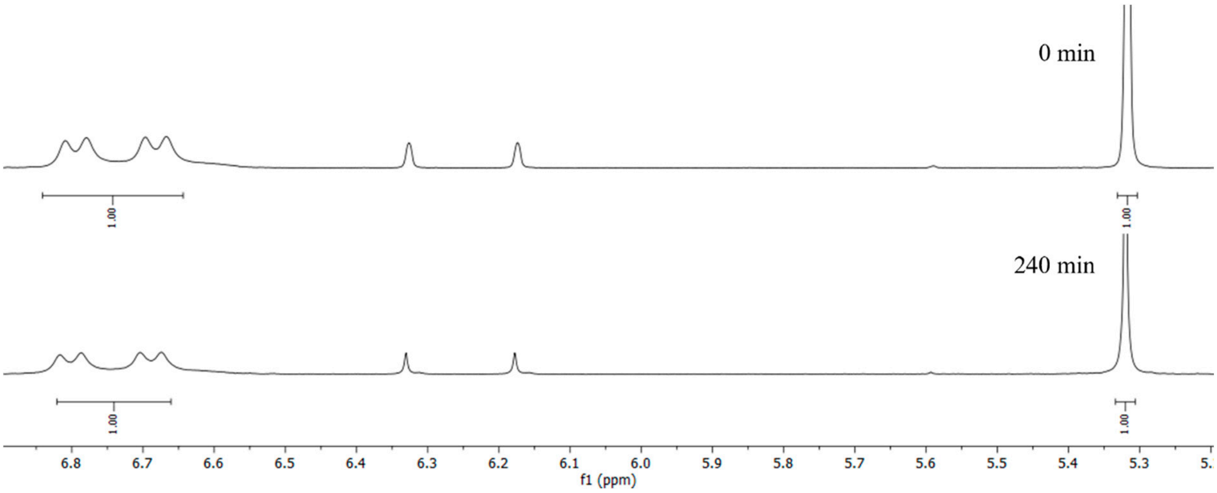

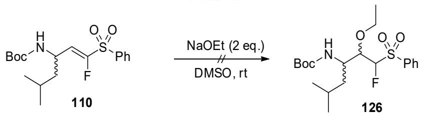

Figure S10. ^{1}H-NMR spectra of **110** after certain time intervals. Reaction of sodium ethanolate with the F-vinyl sulfone moiety.

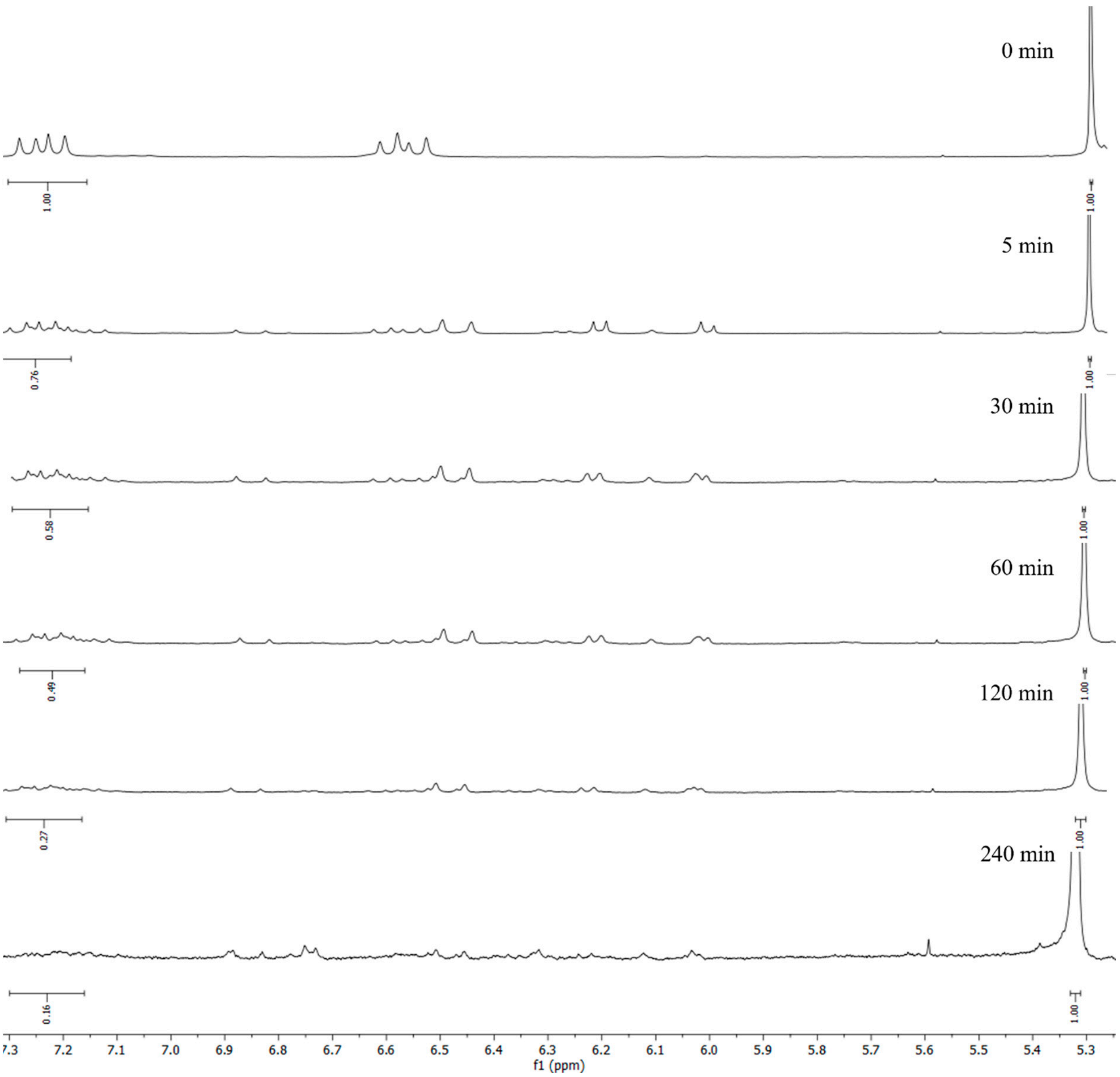

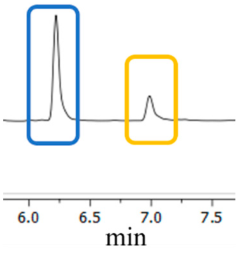

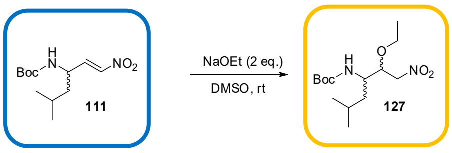

Figure S11. ^{1}H-NMR spectra of **111** after certain time intervals. Reaction of sodium ethanolate with the nitroalkene moiety. LC/MS analysis of the same reaction at 10 min. The blue marked peak was identified by NMR analysis as **111**. The yellow marked peak corresponds to the supposed product **127**, identification with LC-MS was not possible.

Method A (^{13}C-NMR):

The α-ketobenzothiazole or nitrile **115**, **117** (0.07 mmol, 1 eq) was dissolved in DMSO-d_6 (0.5 mL). The reaction was measured in time intervals before (0 min), 5, 30, 60, 120 and 240 mins after the addition of the corresponding nucleophile (PhEtSH/EtONa, 2 eq) in presence or absence of Et_3N (2 eq). The reference signal of DMSO-d_6 was set at 39.52 ppm.

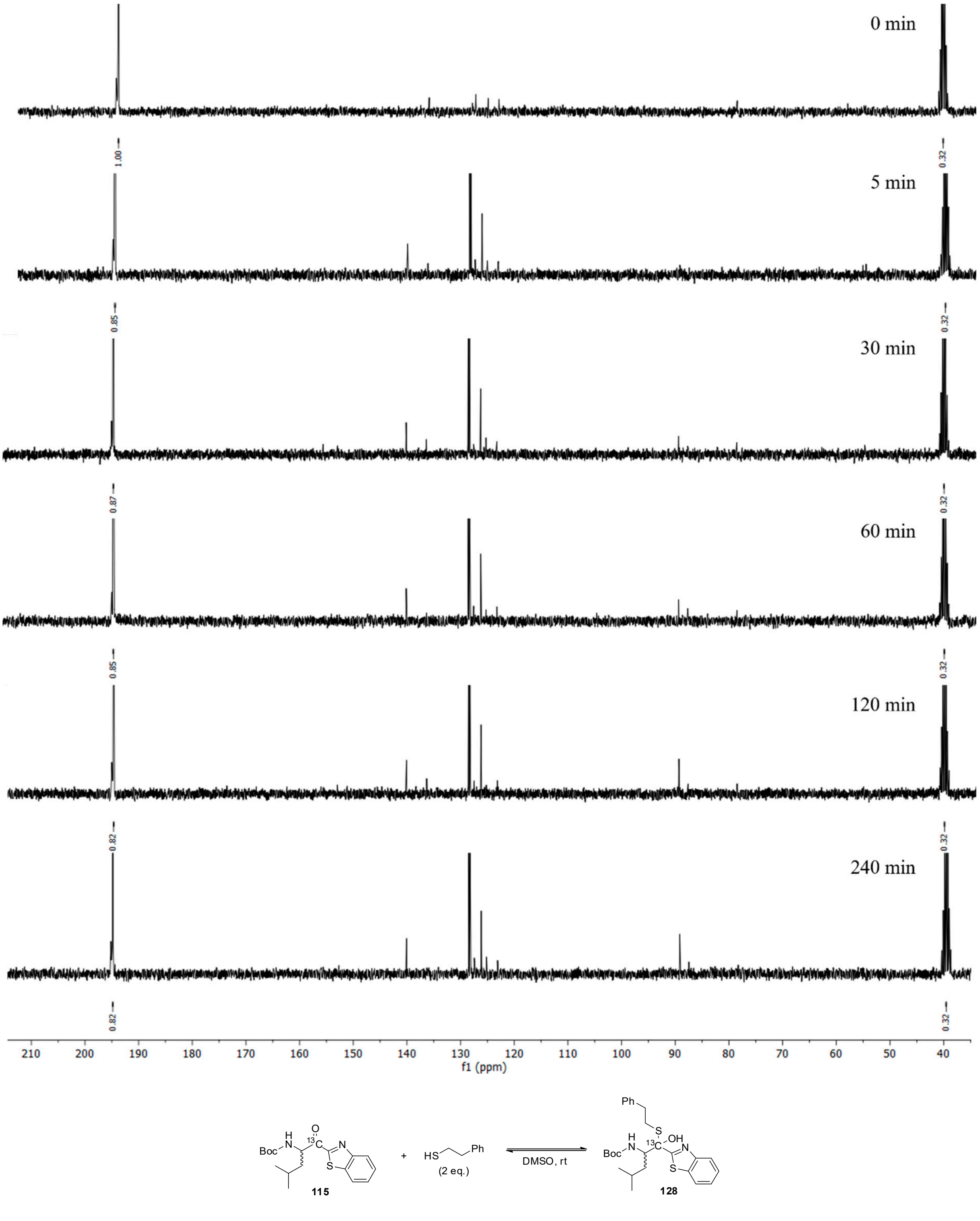

Figure S12. ^{13}C-NMR spectra of **115** after certain time intervals. Reaction of 2-phenylethanthiol with the ketobenzothiazole moiety.

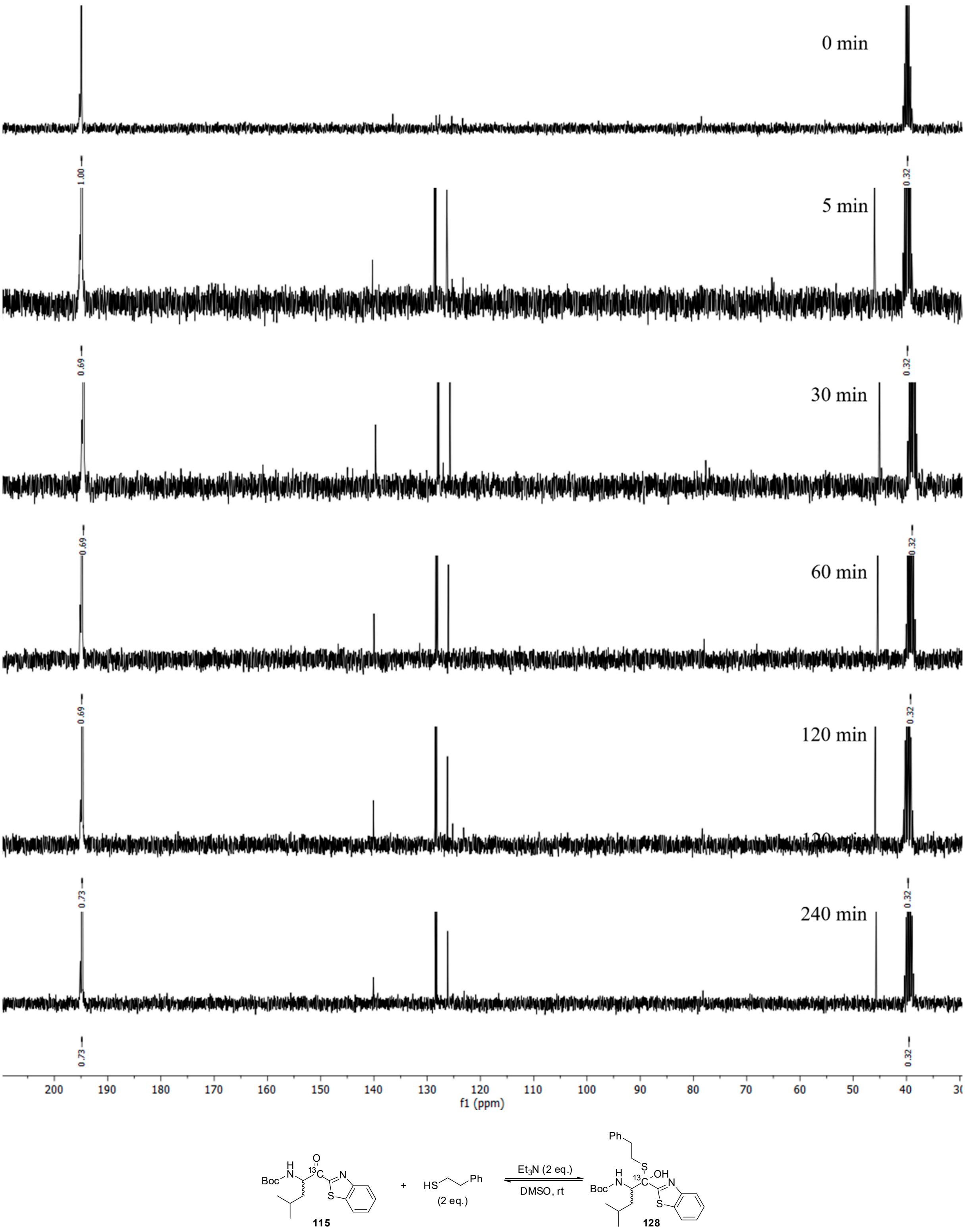

Figure S13. ^{13}C-NMR spectra of **115** after certain time intervals. Reaction of 2-phenylethanthiol with the ketobenzothiazole moiety in presence of Et_3N.

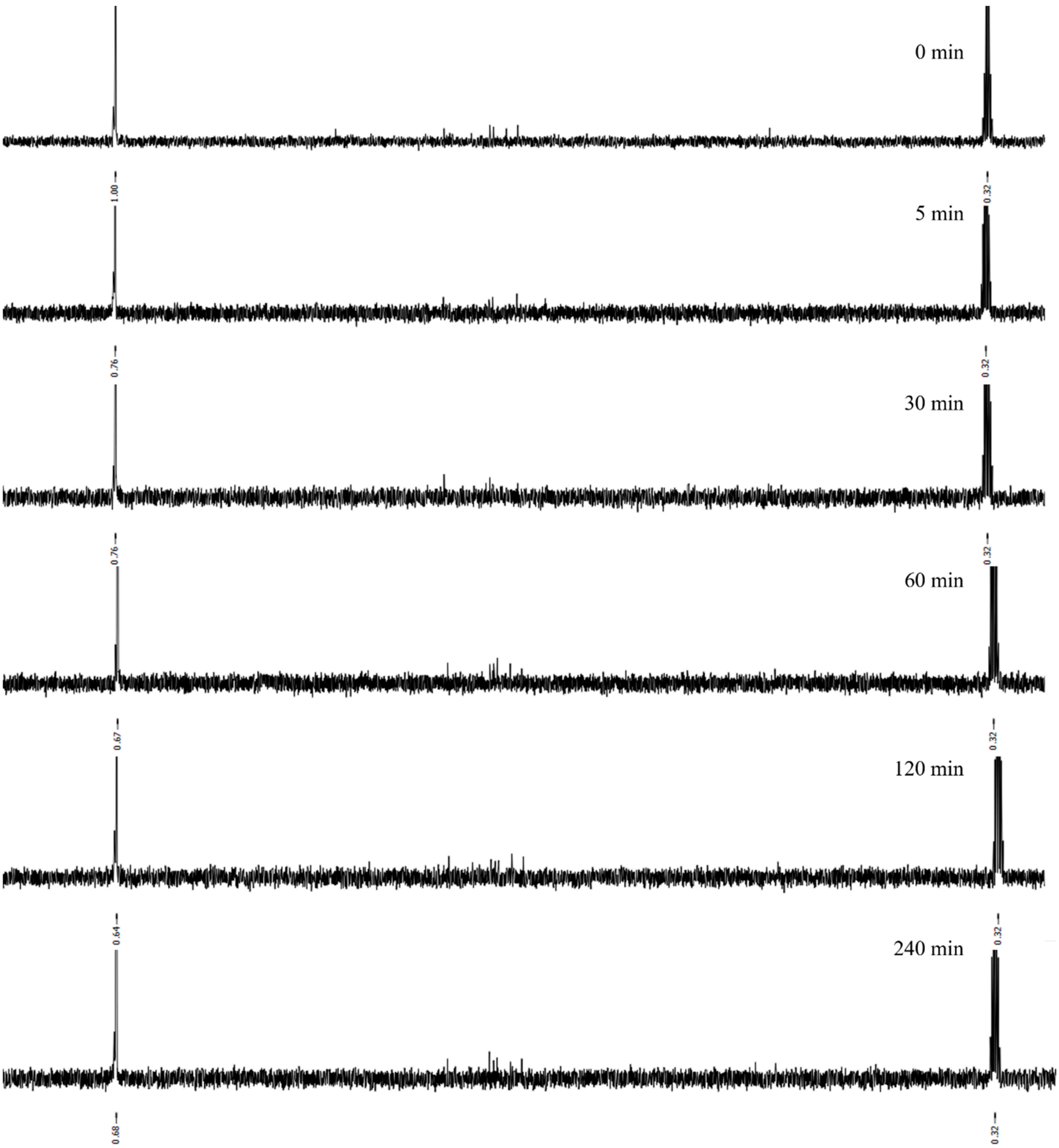
0 min
5 min
30 min
60 min
120 min
240 min
1.00
0.32
0.76
0.32
0.76
0.32
0.67
0.32
0.64
0.32
0.68
0.32

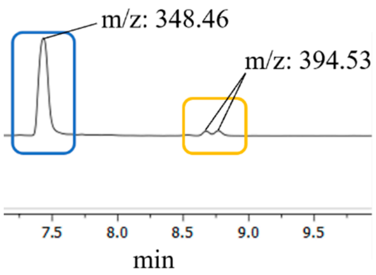
m/z: 348.46
m/z: 394.53
7.5
8.0
8.5
9.0
9.5
min

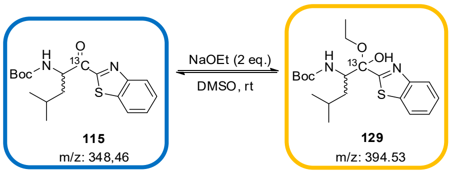
NaOEt (2 eq.)
DMSO, rt
115
m/z: 348,46
129
m/z: 394.53

Figure S14. ^{13}C-NMR spectra of **115** after certain time intervals. Reaction of sodium ethanolate with the α-ketobenzothiazole moiety. LC/MS analysis of the same reaction at 10 min.

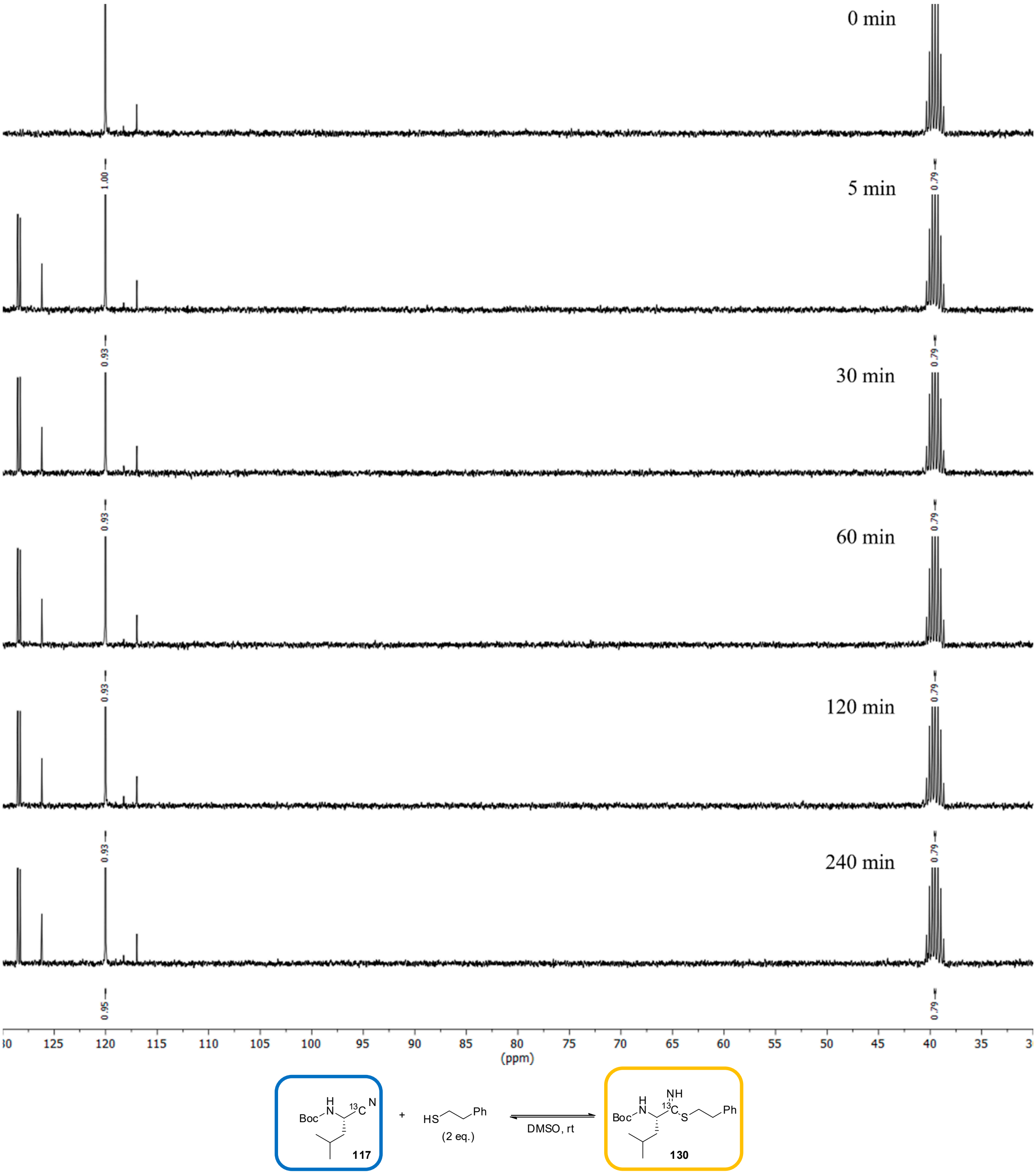

Figure S15. ^{13}C-NMR spectra of **117** after certain time intervals. Reaction of 2-phenylethanthiol with the nitrile moiety. The blue marked peak was identified by NMR analysis as **117**. The yellow marked peak corresponds to the supposed product **130**, identification with LC-MS was not possible.

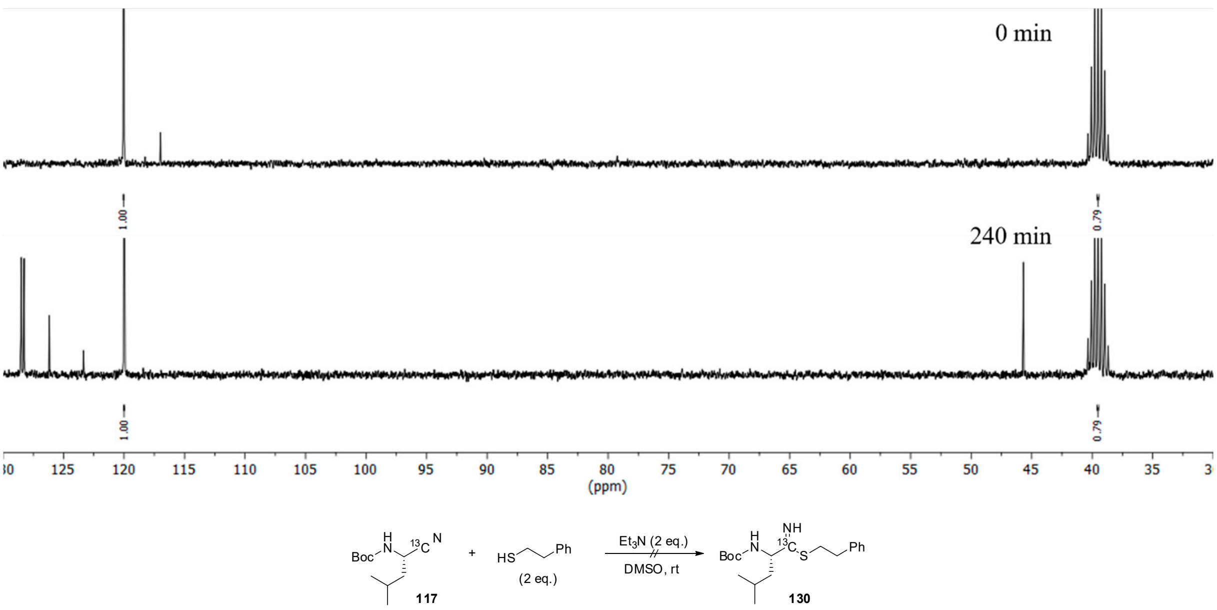

Figure S16. ^{13}C-NMR spectra of **117** after certain time intervals. Reaction of 2-phenylethanthiol with the nitrile moiety in presence of Et_3N.

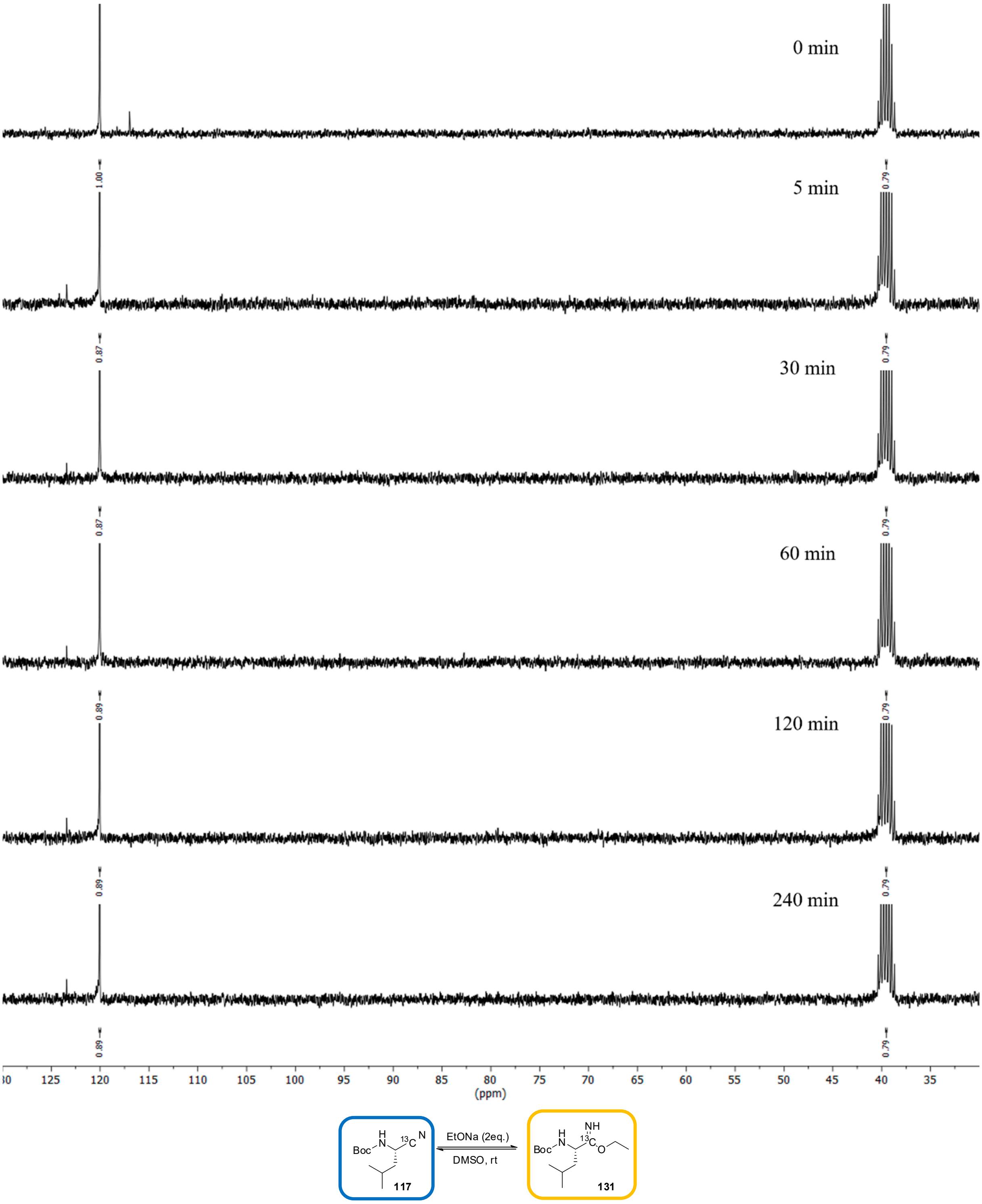

Figure S17. ^{13}C-NMR spectra of **117** after certain time intervals. Reaction of sodium ethanolate with the nitrile moiety. The blue marked peak was identified by NMR analysis as **117**. The yellow marked peak corresponds to the supposed product **131**, identification with LC-MS was not possible.

Method B (LC/MS):

The *β*-lactam **108** (0.07 mmol, 1eq) was solved in DMSO-d_6 (0.5 mL). The reaction was measured in time intervals before (0 min), 5, 30, 60, 120 and 240 mins after the addition of the corresponding nucleophile (PhEtSH/EtONa, 2 eq) in presence of Et_3N (2 eq) or not. In case of PhEtSH as the nucleophile the reaction got quenched by pouring 10 µL of the reaction mixture into a solution of 25 mM TRIS (pH 7.00) and 27 mM maleimide in a 1:1 mixture of acetonitrile/H_2O (990 µL). For the reaction with EtONa as the nucleophile, the reaction solution got quenched by pouring 10 µL of the mixture in 990 µL of a acetonitrile/Tris (50 mM) solution. The quenched reaction mixtures were measured and analyzed via LC-MS.

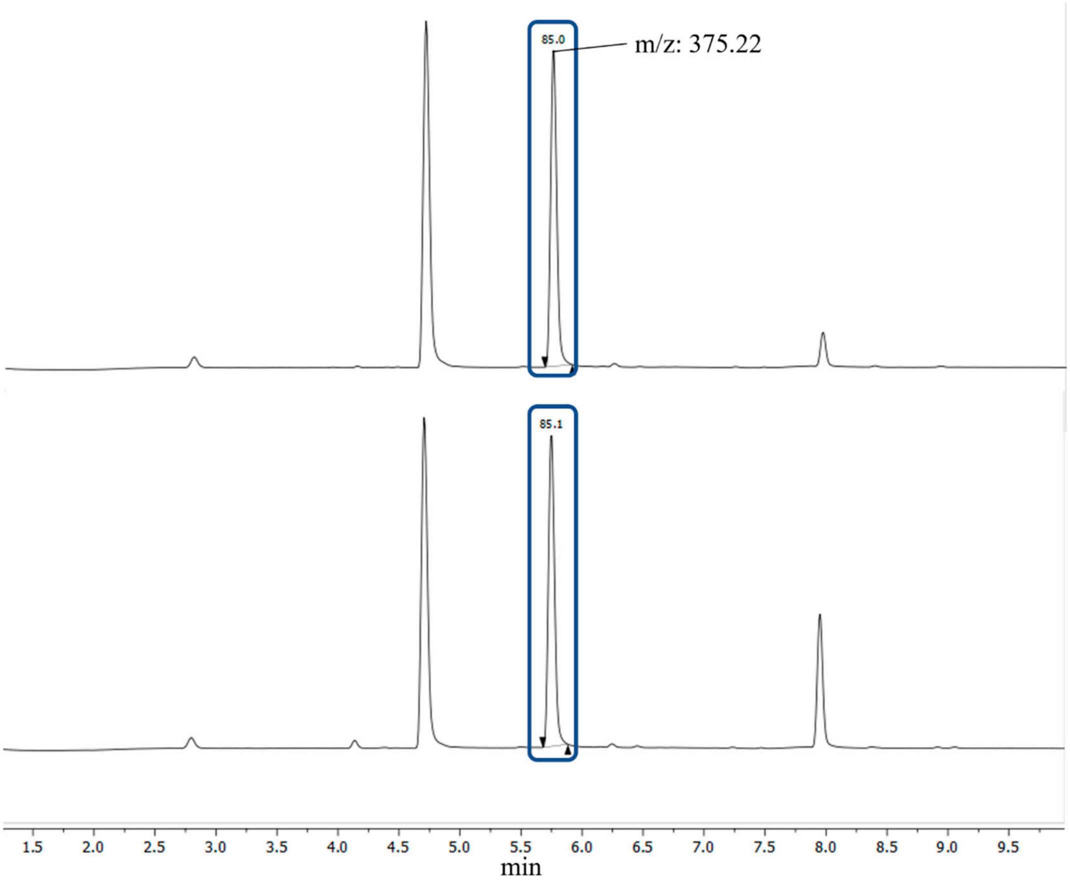

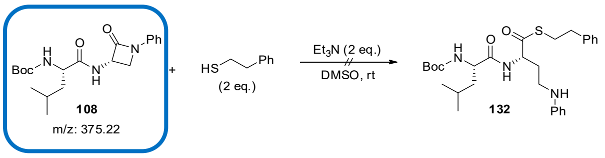

Figure S18. UV chromatogram of **108** after certain time intervals. Reaction of 2-phenylethanthiol with the *β*-lactam moiety in presence of Et_3N and without Et_3N.

Fluorometric inhibition assays

The inhibitory activities of the compounds against the proteases were determined with assays based on fluorogenic or FRET-based substrates. The fluorescence was measured in a white flat-bottom 96-well plate from Greiner Bio-One using a Tecan Infinite F2000 Pro plate reader. Measurements were performed as triplicates. The substrate and the compounds were prepared as stock solutions in DMSO. Each well contained a total volume of 200 µL, consisting of 185 µL buffer, 5 µL inhibitor in DMSO or pure DMSO as negative control, 5 µL substrate in DMSO and 5 µL enzyme solution in buffer. Dilution series between 200 µM and 50 pM were prepared for the determination of the inhibition constants. The fluorescence signal was measured every 30 s for 10 min at 25 °C with the corresponding excitation and emission wavelengths. IC_{50} values for the reversible inhibitors were calculated by fitting the remaining enzymatic activity to the four parameter IC_{50} equation with Y [ΔF/min] as the substrate hydrolysis rate, Y_{max} as the maximum value of the dose response curve at inhibitor concentrations [I] = 0 µM, Y_{min} as the minimum value at high inhibitor concentrations and *s* as the hill coefficient.[1] The sigmoidal dose-response curves of the compounds **30** and **94** against rhodesain are shown exemplary in Figure **S19**.

$$y = \frac{y_{\max} - y_{\min}}{1 + \left(\frac{[\mathrm{I}]}{\mathrm{IC}_{50}}\right)^{s}} + y_{\min}$$

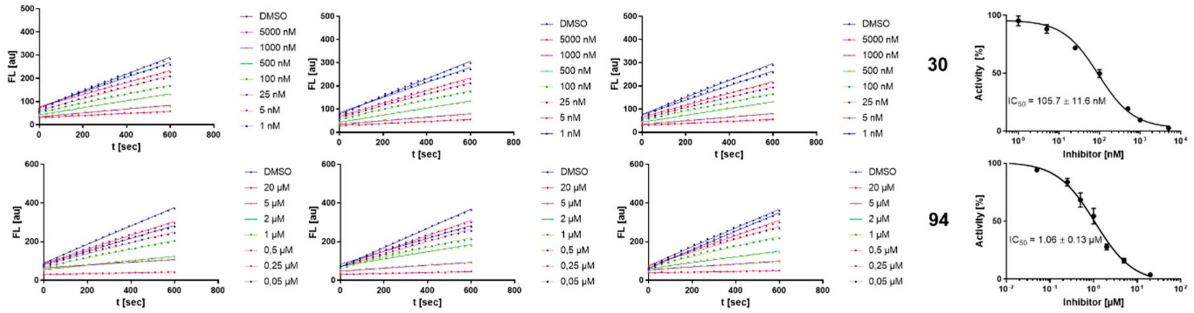

Figure S19. (left) Fluorescence progress curves for **30** and **94** against rhodesain. (right) Plots showing the respective IC_{50} values from sigmoidal fits.

Due to the dependence of the IC_{50} value on the substrate affinity and concentration, the K_i values were calculated with the Cheng-Prusoff equation (1) for appropriate comparison of the inhibitory activities to the other enzymes and inhibitors.[1]

$$K_I = \frac{\mathrm{IC}_{50}}{1 + \frac{[S]}{K_\mathrm{M}}} \qquad 1$$

To evaluate the inhibitory activities of the irreversible inhibitors, reporting IC_{50} values is less suitable, because the IC_{50} values are heavily depending on the incubation time of the enzyme and inhibitor. Therefore, the dissociation constant of the reversible enzyme-inhibitor complex K_I , the maximum inactivation rate k_{inact} and the second-order rate constants of inhibition k_{2nd} were determined. The substrate-conversion plots in presence of the compounds **16**, and **35** against rhodesain are indicating a time-dependent inhibition as shown exemplary in Figure **S20**. It is shown, that the apparent first-order rate constant k_{obs} changed hyperbolically with the inhibitor concentration. A limiting value was approached at higher inhibitor concentrations, indicating a two-step inhibition-mechanism.[1,2]

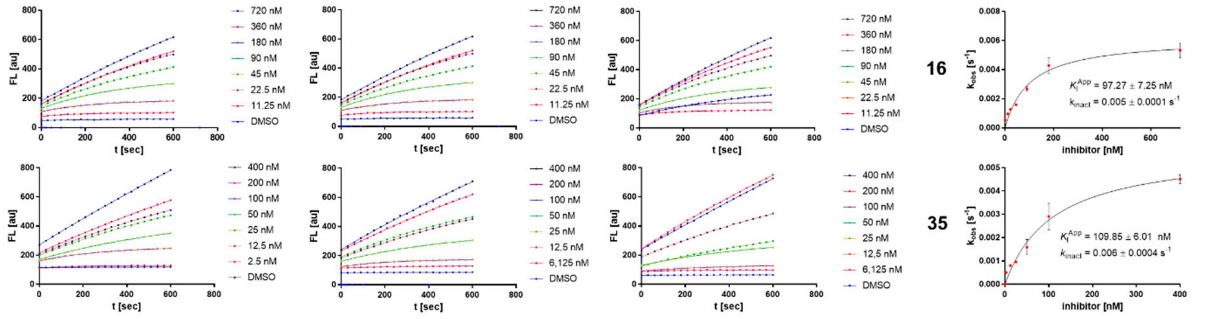

Figure S20. (left) Fluorescence progress curves for **16** and **35** against rhodesain. (right) k_{obs} against [I] plots showing the respective K_I^{App} and k_{inact} values from hyperbolic fits.

For determination of the k_{obs} values, progress curves were analyzed by non-linear regression analysis up to 10 min with the exponential equation (2). F represents the fluorescence intensity, which is proportional to the product concentration [P] and offset represents the background fluorescence.

$$F = [P]^{\propto}(1 - e^{-k_{\text{obs}} \cdot t}) + \text{offset} \qquad 2$$

The received k_{obs} values were fitted against the inhibitor concentrations using the hyperbolic equation (3) to get the k_{inact} and K_I^{app} values. For compound **93** no k_{obs} saturation was observed. A Lineweaver-Burk linearization (4) was used to receive K_I^{app} and k_{inact}.

$$k_{\text{obs}} = \frac{k_{\text{inact}}[I]}{K_{\text{I}}^{\text{App}} + [I]} \qquad 3$$

$$\frac{1}{k_{\text{obs}}} = \frac{K_{\text{I}}^{\text{App}}}{k_{\text{inact}}[I]} \frac{1}{[I]} + \frac{1}{k_{\text{inact}}} \qquad 4$$

The K_I^{app} values were corrected to the K_I values by the Cheng-Prussoff equation (5), for better comparison of the inhibitory constants to the other enzymes and compounds.

$$K_I = \frac{K_I^{App}}{1 + \frac{[S]}{K_M}} \qquad 5$$

The second-order rate constant of inhibition k_{2nd} were calculated from the equation (6).[1]

$$K_{2nd} = \frac{k_{inact}}{K_I} \qquad 6$$

Buffers and substrates

The following buffers and substrates were used for the respective assays: Rhodesain (50 mM Na-acetate pH 5.5, 5 mM EDTA, 200 mM NaCl, 50 µM DTT, 0.005% Brij 10 µM Cbz-Phe-Arg-AMC, 1 nM rhodesain), Cathepsin S (50 mM KH_2PO_4, pH 6.5, 50 mM K_2HPO_4, 2.5 mM DTT, 2.5 mM EDTA, Z-Val-Arg-Arg-AMC, 10 nM CatS), *β*-5-subunit of the proteasome (50 mM Tris HCl, 25 mM NaCl, 10 mM NaCl, $MgCl_2 \cdot 6\ H_2O$, 100 µM Succ-Leu-Leu-Val-Tyr-AMC, 0.02 mg/mL proteasome *β*5-subunit), Sars-Cov-2 M^{Pro} (20 mM TRIS, pH 7.5, 0.1 mM, NaCl 200 mM, DTT 1 mM, 50 nm M^{Pro}), an internally quenched 14-mer fluorogenic (FRET) peptide (DABCYL-KTSAVLQSGFRKME-EDANS)), uPA (50 mM Tris HCl, 150 mM pH 7.5, NaCl, 10 mM $CaCl_2$, 0.005% TX-100, 240µM Cbz-Gly-Gly-Arg-AMC, 2.5 U uPA). As positive controls, several well-known inhibitors have been used for the respective enzymes and their biological activity was reproduced.

The human uPa and recombinant CatS were purchased from Sigma Aldrich, and the proteasome *β*5-subunit from Enzo Life sciences. Rhodesain and SARS-CoV-2 M^{pro} were expressed under the conditions described below.

Protein Expression and purification

SARS-CoV-2 M^{pro}.

The expression of SARS-CoV-2 Mpro was performed as described previously.[3] Briefly, the pMal-c2 plasmid (New England Biolabs, Ipswich, MA, USA), harboring the DNA of the entire SARS-CoV-2 M^{pro} coding sequence framed by a short sequence specifying the 5 *C*-terminal residues of nonstructural protein 4 (nsp4/nsp5 cleavage site) at the 5' end and the sequence of a hexahistidine tag at the 3' end. The presence of the nsp4/nsp5 cleavage site between the plasmid's MBP and the M^{pro} sequence together with the native nsp5/nsp6 cleavage site between

M^{pro} and the hexahistidine tag, enables the generation of the native termini of M^{pro} by autocatalytic cleavage. The plasmid construct was transformed in competent Escherichia coli (E. coli) BL21-Gold (DE3) cells (Agilent Technologies, Santa Clara, CA, USA). Cells were grown in LB medium supplemented with 100 µg/mL ampicillin at 37 °C until they reached an optical density at λ=600 nm (OD_{600}) of ~0.7 and expression was induced with 0.3 mM isopropyl-D-thiogalactoside. Overexpression of M^{pro} was carried out for 16 h at 18 °C. To suppress early cleavage of the hexahistidine tag, all purification steps until IMAC were performed while cooling buffers and samples on ice. For purification, cells were harvested by centrifugation and resuspended in lysis buffer (20 mM Tris−HCl pH 7.8, 150 mM NaCl, 20 mM imidazole, Lysozyme, DNase, RNase) and lysed by several cycles of sonication (Sonoplus HD 2200; Bandelin, Berlin, Germany). The lysate was cleared by centrifugation (one hour at 40 krpm) and immediately subjected to immobilized metal affinity chromatography (IMAC) on a HisTrap HP 5 ml column (Cytiva Europe GmbH, Freiburg im Breisgau. Germany) on a ÄKTA start protein purification system (GE Healthcare, Chicago, IL, USA). After washing with 20 column volumes (CV) of IMAC buffer A (20 mM Tris−HCl pH 7.8, 200 mM NaCl, 20 mM imidazole), M^{pro} was eluted in a linear gradient of IMAC buffer B (20 mM Tris−HCl pH 7.8, 200 mM NaCl, 500 mM imidazole). The collected fractions containing M^{pro} were concentrated to a volume of 5 mL using Vivaspin 10 MWCO spin concentrators (Sartorius AG, Göttingen, Germany) and were subjected to a gel filtration step using a HiLoad 16/600 Superdex 75 pg column (GE Healthcare) in SEC buffer (20 mM Tris−HCl pH 7.8, 150 mM NaCl, 1 mM ethylenediaminetetraacetic acid (EDTA), 1 mM dithiothreitol (DTT)). After dilution to 10 µM and adjustment to 10% (v/v) glycerol as cryoprotectant, M^{pro} was shock frozen in liquid N_2 and stored at −80 °C. Throughout purification, protein concentrations were measured via absorbance at 280 nm using a NanoDrop 2000 Spectrophotometer (Thermo Scientific Waltham, MA, USA). Sample purity was assessed via Coomassie brilliant blue-stained sodium dodecyl sulfate− polyacrylamide gel electrophoresis (SDS-PAGE).

Rhodesain

Rhodesain was recombinantly expressed in *P. pastoris* according to a method adapted from literature[4,5]: A stored *P. pastoris* X-33 mutant, stably transformed with the rhodesain gene cloned into the pPICZα A vector, was cultured in buffered minimal glycerol supplemented with ampicillin (BMG_{Amp}) at 30 °C to an optical density (λ = 600 nm) of 2–3. To induce AOX1-controlled expression, BMG_{Amp} was exchanged for buffered minimal methanol with ampicillin (BMM_{Amp}). Incubation was continued for 72 h, and 0.5% (V/V) methanol was added every

12 h. After this period, cells were pelleted by centrifugation and discarded. The culture supernatant containing secreted rhodesain was filtered (0.2 µm filter, cellulose acetate), adjusted to 2 M sodium chloride and loaded overnight onto a Phenylsepharose FF (high sub) column (V = 20 mL), equilibrated to chromatography conditions described below, attached to an ÄKTA start system. Hydrophobic interaction chromatography was performed by gradient elution with falling concentrations of sodium chloride (2 M to 0 M) in 20 mM sodium citrate buffer (pH 5.5). Rhodesain was eluted afterwards with MQ-water. The eluate was concentrated with centrifugal filter units (10 kDa MWCO, regenerated cellulose) to a volume ≤ 5 mL. Subsequently, size exclusion chromatography was performed with 20 mM sodium citrate buffer with 200 mM sodium chloride (pH 5.5) on an ÄKTA start system equipped with a HiLoad 16/600 Superdex 75 pg column. Rhodesain-containing fractions were pooled and then dialyzed against MQ-water for 4 h (dialysis tubing, 6 kDa MWCO, regenerated cellulose). The desalted rhodesain solution was lyophilized overnight and stored at ≤5 °C.

BMG_{Amp} / BMM_{Amp}:

1% (V/V) glycerol OR 0.5% (V/V) methanol

100 mM potassium phosphate buffer pH 6

3.4 g/L yeast nitrogen base without amino acids, without ammonium sulphate

10 g/L Ammonium sulphate

0.4 mg/L Biotin

100 mg/L Ampicillin

All other enzymes were purchased from Sigma-Aldrich.

Computational studies

Molecular docking

Since the inhibitors were designed to react covalently with the cysteine or the serine-/ threonine-proteases two different docking approaches were followed. First, a conventional non-covalent docking was performed, to estimate affinity and geometry of the pre-organized enzyme-inhibitor complex, secondly a covalent docking was used to determine the final covalent enzyme-inhibitor complex.

In both docking setups crystallographic reference ligands were used for validation *via* redocking (Table S2), all inhibitors with the same peptidic recognition sequence with varying warheads were docked against their corresponding target enzyme.

Molecular docking experiments were performed using the following crystal structures freely available in the protein data bank (PDB)[6]: rhodesain covalently bound to K11777 (PDB entry 2P7U)[7]; cathepsin S covalently bound to morpholine-4-carboxylic acid [1s-(2-benzyloxy-1r-cyano-ethylcarbamoyl)-3-methyl-butyl]amide (PDB entry 1MS6)[8]; urokinase type plasimogen activator covalently bound to *N*-(Isobutoxycarbonyl)-D-seryl-N-((1s)-4-{[amino(imino)methyl]amino}-1-formylbutyl)-L-alanineamide (pdb entry 1W10)[9]; Main Protease of SARS-Cov-2 covalently bound to GRL-024-20 (pdb entry 6XR3)[10]; Chain K of the human 20 S proteasome covalently bound to bortezomib (pdb entry: 5LF3)[11]. All ligands were energetically minimized prior docking with Molecular operating environment (MOE 2020.09)[12] using the MMF94x force field.[13]

Docking approach A: non-covalent docking with LeadIT

The non-covalent docking was performed with LeadIT-2.3.2.[14] The receptors were prepared in MOE with the protonate3D functionality and the covalent bonds between the co-crystallized ligands and the corresponding proteases were untethered *via* the Builder tool in MOE. In case of the proteasome the *β*-5-subunit (chain K) was extracted with PyMOL-2.5.2[15] and used for all subsequent docking operations. For all receptors the binding site was defined as a 6.5 Å shell around the bound reference ligand. Water molecules that form at least three hydrogen bonds with the receptor and ligand were kept as part of the binding site. The docking was performed under default settings using the enthalpy-entropy hybrid approach with 2000 solutions *per* iteration and fragmentation. Only the top pose of the initial docking was kept and re-scored using the HYDE scoring function.[16] For the *β*-5 subunit of the proteasome, pharmacophore constraints needed to be included to obtain reasonable binding modes. The nitrogen atoms of the peptide backbone were therefore defined as h-bond donors with a 1 Å sphere radius.

Docking approach B: covalent docking with MOE

Covalent docking was performed with MOE. The receptors were prepared using the 3D protonation tool inside MOE. For the covalent reaction of the different warheads, the already existing template reactions were used or customized using the combinatorial library tool of MOE. Initial 30 poses from the triangle match placement with London ΔG scoring were re-scored using the Affinity ΔG scoring function and induced fit refinement implemented in MOE. 10 Poses were kept and visually inspected for binding geometry the interactions matching between the docked inhibitor pose and co-crystallized ligand with the enzyme. The poses best matching inspected interaction patterns are further discussed.

Table S2. Redocking.

Enzyme (pdb entry)	Reference ligand ID	Redocking FlexX (RMSD/ Å)	FlexX score (kJ/mol)
UPA (1W10)	SJ1	0.97	–41.8
β-5-subunit 20S-Proteasome (5LF3)	BO2	0.91	–21.7
Cathepsin S (1MS6)	BLN	0.94	–18.0
SARS-Cov-2 M^{pro} (6XR3)	V7G	0.53	–49.1
Rhodesain (2P7U)	D1R	1.48	–20.2

Table S3. Results of molecular docking analysis.

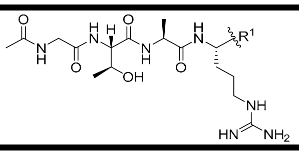		Target (pdb entry) uPA (1W10)		
**R^1**	Distance (electrophilic C-Ser195-O) / Å	FlexX score (kJ/mol)	Hyde score (kJ/ mol)[a)]	Covalent docking score (Affinity ΔG, MOE/ kcal/mol)
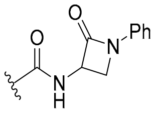 **103**	2.84	–51.59	17	–5.97
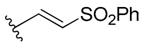 **106**[b)]	2.74	–48.96	3	–5.01
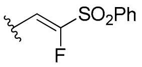 **107**[b)]	4.04	–40.11	5	–5.37

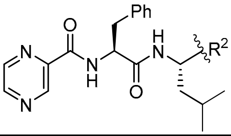		Target (pdb entry) *β*-5-subunit of 20S-Proteasome (5LF3)		
**R^2**	Distance (electrophilic C-Ser195-O) / Å	FlexX score (kJ/mol)	Hyde score (kJ/ mol)[a)]	Covalent docking score (Affinity ΔG, MOE/ kcal/mol)
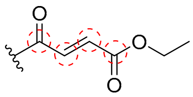 **68**[b),c)]	2.56	–16.64	–14	–4.32

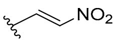 **59**	2.52	–21.67	–22	–2.93
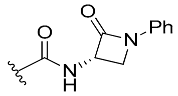 **72**[b)]	4.95	–21.48	–13	–3.08
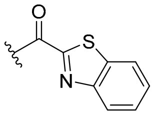 **62**	3.16	–23.97	–2	–3.40
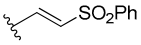 **58**[b)]	6.77	–12.33	–25	–2.73
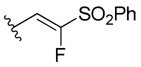 **55**[b)]	5.90	–9.76	–4	–2.44
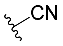 **74**	2.27	–22.96	–22	–3.17

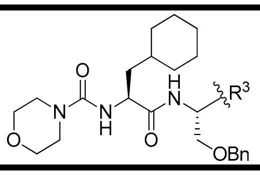

Target (pdb entry)
Cathepsin S (1MS6)

R^3	Distance (electrophilic C-Ser195-O) / Å	FlexX score (kJ/mol)	Hyde score (kJ/ mol)[a)]	Covalent docking score (Affinity ΔG, MOE/ kcal/mol)
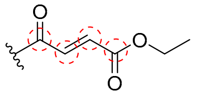 **40**[c)]	3.37	–21.56	–51	–5.44
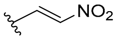 **49**	2.69	–22.88	–32	–4.38
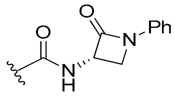 **45**	3.03	–30.80	–24	–4.61
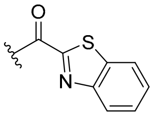 **42**	2.96	–30.67	–30	–6.29
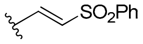 **35**	3.07	–27.35	–48	–5.32
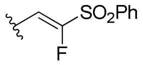 **36**	3.30	–25.17	–45	–5.19
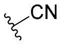 **30**	2.87	–26.22	–40	–3.00

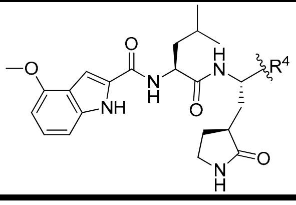

Target (pdb entry)
Sars-Cov-2 MPro (6XR3)

R^4	Distance (electrophilic C-Ser195-O) / Å	FlexX score (kJ/mol)	Hyde score (kJ/ mol)[a)]	Covalent docking score (Affinity ΔG, MOE/ kcal/mol)
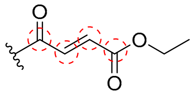	3.81	–44.50	–11	–5.38

**81**[c)]				
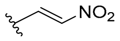 **88**	2.90	–42.80	–9	–3.56
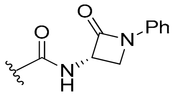 **96**	4.91	–43.37	–9	–5.89
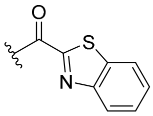 **90**	3.41	–46.96	–8	–4.88
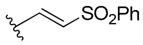 **93**	3.02	–36.04	–8	–4.66
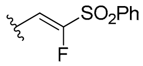 **94**	3.58	–37.95	–21	–5.50
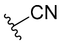 **84**	3.04	–44.38	–27	–4.42

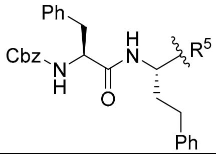			Target (pdb entry) Rhodesain (2P7U)	
**R**[5]	Distance (electrophilic C-Ser195-O) / Å	FlexX score (kJ/mol)	Hyde score (kJ/ mol)[a)]	Covalent docking score (Affinity ΔG, MOE/ kcal/mol)
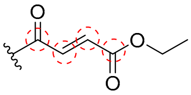 **22**[c)]	2.70	–23.18	–33	–4.53
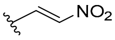 **13**	3.50	–24.03	–12	–2.66
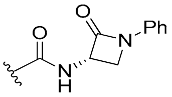 **24**	2.93	–28.88	–21	–4.22
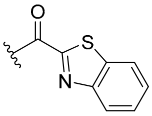 **18**	2.54	–25.62	–28	–3.91
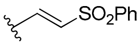 **16**	3.00	–27.11	–17	–2.96
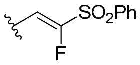 **17**	2.76	–26.92	–23	–3.58
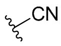 **25**	2.60	–26.35	–17	–3.04

a) The HYDE-scores were in the negative range for all inhibitors except the UPA inhibitors. This could be due to the high polarity of these inhibitors with calculated log P values below 0 and TPSA values above 200 Å2 which could hamper the desolvation term of the HYDE rescoring function.

b) A covalent reaction between the inhibitor and the active site is unlikely/ not expected.

c) The four possible reaction sites of the 4-oxo-enoate warhead are depicted in red dashed circles. Only one distance between the nucleophilic residue in the active site and the 4-oxo-enoate warhead was measured.

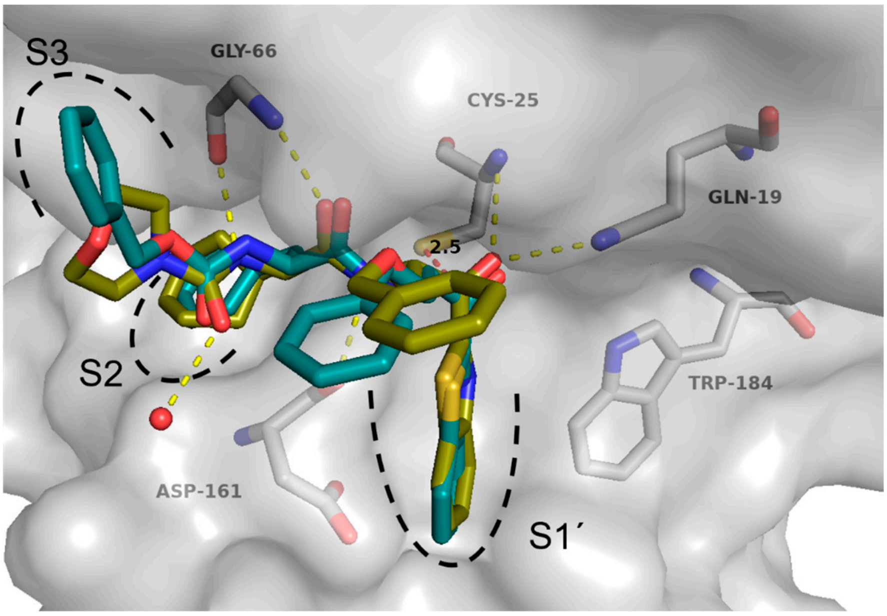

Figure S21. Superposition of the non-covalent docking poses of α-ketobenzothiazole **42** (deepolive C-atoms) and **18** (deepteal C-atoms) inside the active site of rhodesain (pdb entry: 2P7U). Polar interactions between **42** and the enzyme are depicted as yellow dashed lines. The distance between the sulfur atom of Cys25 and the electrophilic C-atom of **42** is depicted as red dashed line and the distance is given in Å.

Quantum mechanics simulations / kinetics

Computational Details

All calculations were performed with the Gaussian 16 program (Revision A.03)[17]. For all geometry optimizations, the range-separated, dispersion corrected hybrid functional ωB97X-D and the Pople basis set 6-31+G* were employed.[18] Implicit solvation in water was included with the SMD solvation method.[19] Free energies included a concentration correction resulting from the change in standard states going from gas phase to condensed phase.[20,21] For thermodynamic calculations additional Single-Point calculations with ωB97X-D/6-311++G** [4] were performed, leading to variations of about 2 kcal mol^{-1} when comparing thermodynamic and kinetic data. Figures S10-S13 illustrate the thermodynamic calculation scheme. As a model system, only the warhead of the inhibitor was considered. As sulfur-based nucleophilic systems, MeSH and MeSH + NEt_3 were chosen to mimic the reaction in absence and presence of a base, respectively. For EtONa, the MeO^- anion was chosen to represent the negatively charged nucleophile in solution. For the possibility of product protonation, the reactants consisted of the warhead, MeO^- anion with three water molecules and one separately computed water molecule to account for the change in entropy by an addition. Consequently, the products consisted of the MeO-warhead adduct and OH^- stabilized by three water molecules. Since the neutral adduct of α-ketobenzothiazole and MeO^- were computed to be strongly endergonic (11 kcal mol^{-1}), we concluded that the formation of the deprotonated hemiacetal is favored. Since an anionic compound as a product would be poorly stabilized, the free energy of reaction was computed as depicted in Figure S13, by adding two water molecules to stabilize the anionic product. For the evaluation of reaction mechanisms, separately optimized reactants (R), van-der-Waals complexes of reactants (R vdw), intermediates (Int), transition states (TS), van-der-Waals complexes of products (P vdw) and products (P) were optimized and confirmed by frequency analysis. As sulfur nucleophiles the inhibition reactions with MeSH and MeSH + NEt_3 were computed, the reaction with NaOEt was modeled as MeO^- + $3H_2O$. For the reaction of 4-oxyenoate and α-ketobenzothiazole with MeSH an additional reaction path including a water assisted proton transfer was calculated by adding three water molecules in various orientations. The performed cluster calculations lead to uncertainties because there is a plethora of local minima in the orientation of the water molecules, leading to a strong impact on the energy. Additionally, the addition of water molecules influences the reaction thermodynamics, because of varying reactant and product stabilization.

Additional reaction path calculations

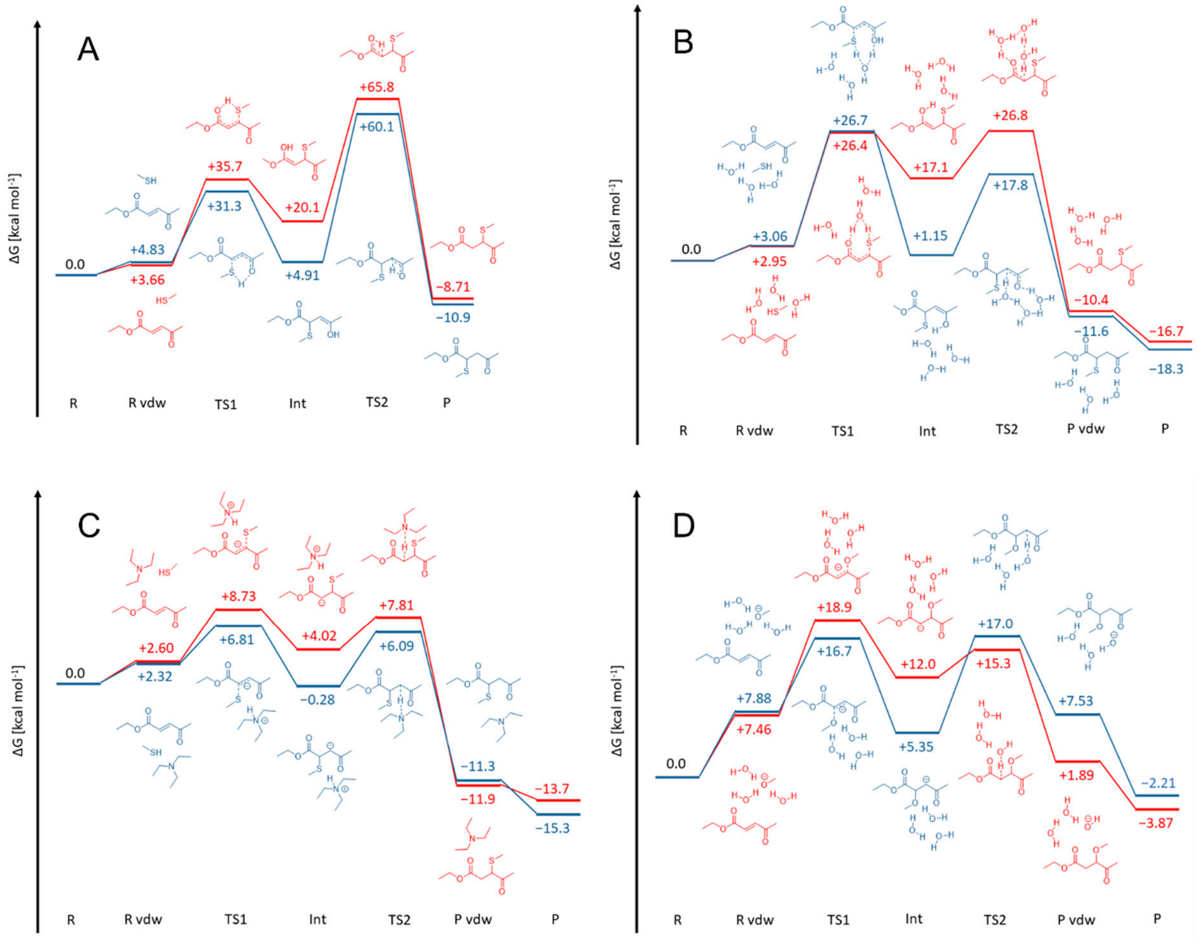

Figure S22. A: Free energy reaction paths for C_α (red) and C_β addition (blue) of the 4-oxoenaoate warhead with MeSH. B: Free energy reaction paths for C_α (red) and C_β addition (blue) of the 4-oxoenaoate warhead with MeSH in presence of three water molecules that facilitate the proton transfer. C: Free energy reaction paths for C_α (red) and C_β addition (blue) of the 4-oxoenaoate warhead with MeSH in presence of triethylamine as a base. D: Free energy reaction paths for C_α (red) and C_β addition (blue) of the 4-oxoenaoate warhead with MeO^- in presence of three water molecules, acting as a proton donor to yield

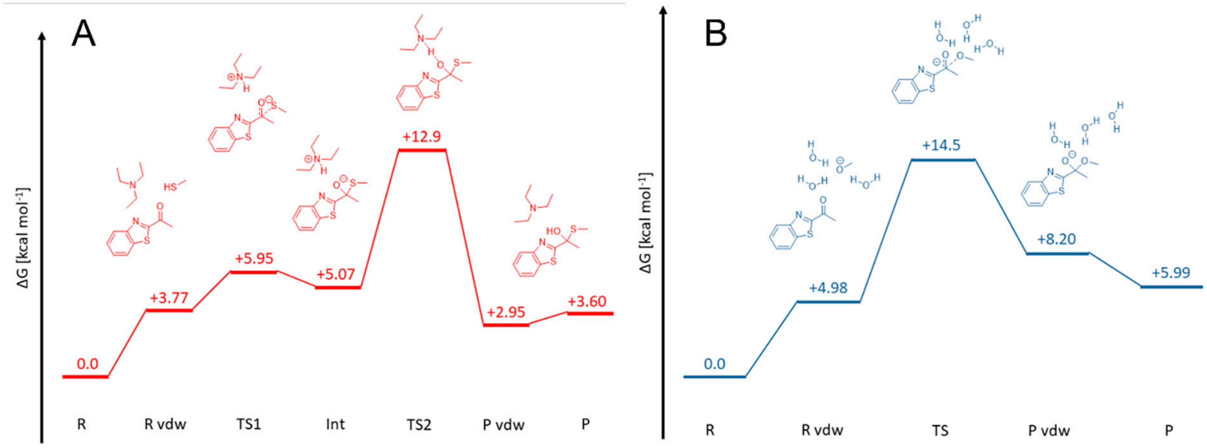

the product.

Figure S23. A: Free energy reaction path of α-ketobenzothiazole with MeSH in presence of Et_3N. B: Free energy reaction path of α-ketobenzothiazole with MeO^- in presence of three water molecules. Since the deprotonated hemiacetal is computed separately for P, it is more endergonic than in the thermodynamic calculations, where it was stabilized by two water molecules (3.09 kcal mol^{-1}).

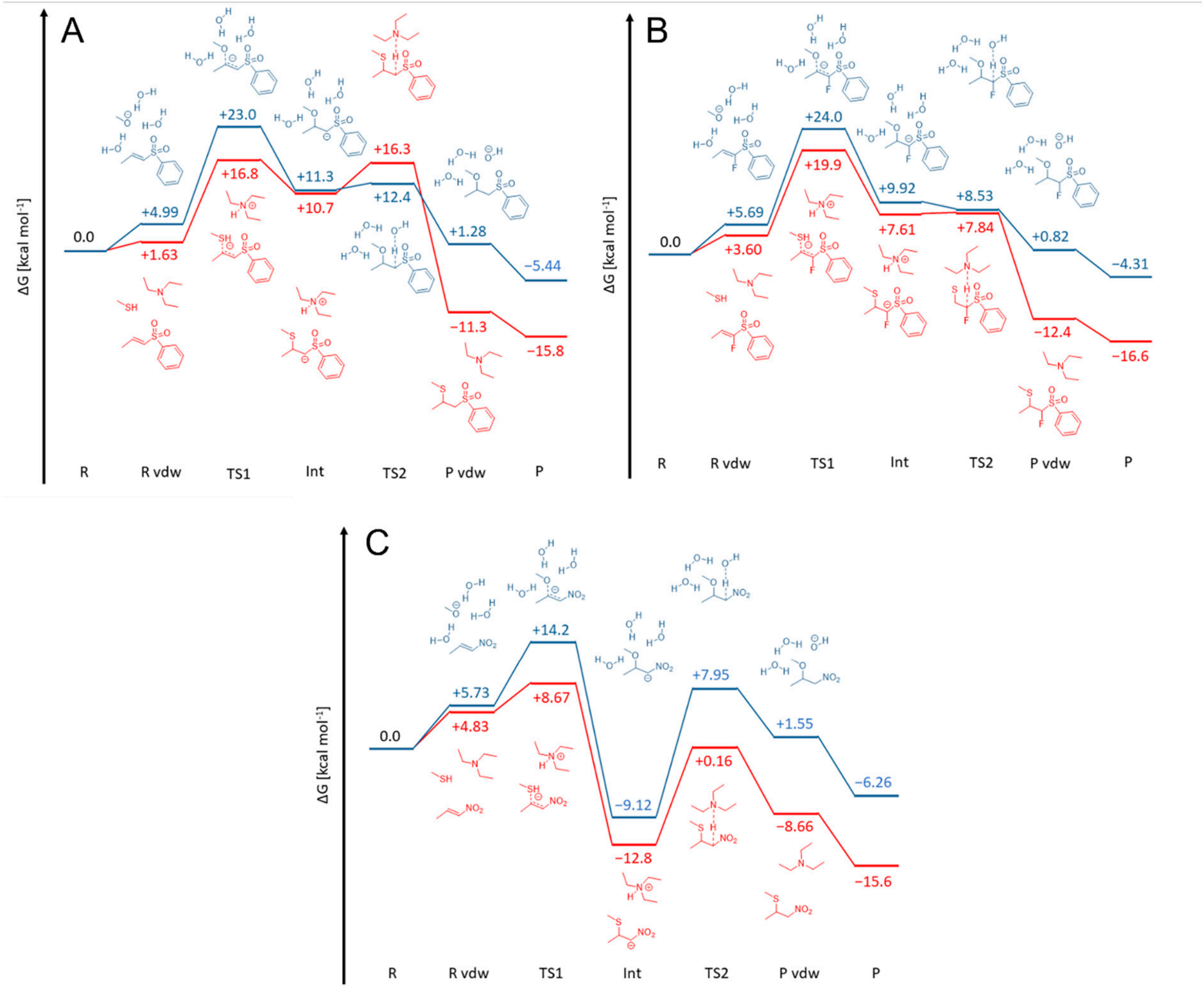

Figure S24. A: Free energy reaction path of vinyl sulfone with MeSH in presence of Et_3N (red) and with MeO^- + $3H_2O$. B: Free energy reaction path of F-vinyl sulfone with MeSH in presence of Et_3N (red) and with MeO^- + $3H_2O$. C: Free energy reaction path of nitroalkene with MeSH in presence of NEt_3 (red) and with MeO^- + $3H_2O$.

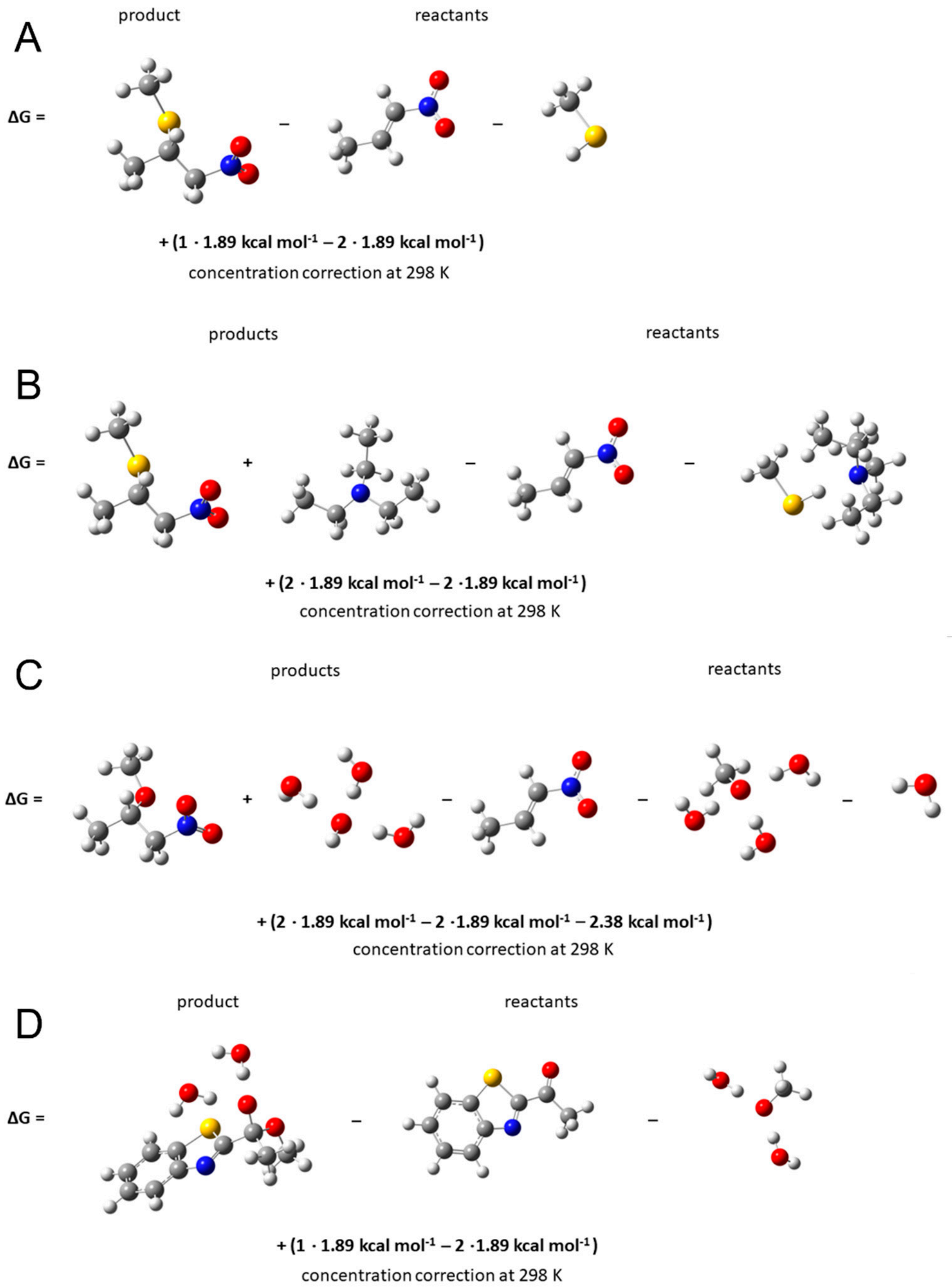

Figure S25. A: Calculation of free energies of reaction for MeSH as a nucleophile including a concentration correction resulting from the change in standard states going from gas phase to condensed phase. Exemplarily shown for the nitroalkene warhead. B: Calculation of free energies of reaction for MeSH + Et_3N as nucleophile and base including a concentration correction resulting from the change in standard states going from gas phase to condensed phase. Exemplarily shown for the nitroalkene warhead. C: Calculation of free energies of reaction for MeO^- in presence of three water molecules and a separate water molecule to account for the change in number of molecules. A concentration correction resulting from the change in standard states going from gas phase to condensed phase is included. Exemplarily shown for the nitroalkene warhead. D: Calculation of free energies of reaction for the *α*-ketobenzothiazole warhead with MeO^- in presence of two water molecules. Since the anionic product separately would be very unstable the product consists of the deprotonated hemiacetal stabilized by two water molecules. A concentration correction resulting from the change in standard states going from gas phase to condensed phase is included. Exemplarily shown for the nitroalkene warhead.

Synthesis

General Methods and Materials:

All reagents and solvents were purchased commercially and used as provided by the supplier without further purification. Solvents for synthesis, extraction, and chromatography were of analytical grade. Moisture-sensitive reactions were carried out under argon atmosphere, and anhydrous solvents were used as provided by the commercial supplier. Reaction progress was monitored by thin-layer chromatography using Alugram Xtra F254 silica plates from Macherey-Nagel and/or LC-MS. Therefore, an Agilent 1100 series HPLC system and an Agilent Poroshell 120 EC-C18, 150 x 2.10 mm, 4 μm column coupled to an Agilent 1100 series LC/MSD Trap with electron spray ionization (ESI), was used. The identities and purities of compounds were determined by the same LC-MS system with a gradient of acetonitrile and water (+0.1% formic acid). Signals were detected at 210/254 nm with quantitation by AUC and masses were determined in positive ionization mode (ESI). HPLC purification was performed with the Agilent 1290 II Infinity Preparative LC System using an InfinityLab Pursuit XRs C18, 30 x 250mm, 5 µm, preparative LC column. Flash chromatography was performed with the Biotage Isolera™ One system using prepacked columns from Biotage. Silica gel (0.040 – 0.063 mm) from Macherey-Nagel was used for column chromatography. Optical rotations $[\alpha]_D^{22}$ were measured on an P3000 polarimeter from Krüss at 22 °C and are reported in $ml \cdot dm^{-1} \cdot g^{-1}$ with the concentration *c* being g/100 ml. Fourier-transformed ATR-corrected IR spectra were measured on an Avatar 330 single crystal spectrometer from ThermoNicolet. Melting points (uncorrected) were measured with an MPM-H3 using semi-open capillaries. NMR spectra were recorded as stated individually on Bruker Fourier 300 MHz, Bruker Avance DSX 400 MHz and Bruker Avance III 600 MHz. Chemical shifts are indicated in parts per million (ppm), with the solvent resonance ($CDCl_3$, DMSO-d_6 or CD_3OD from Deutero GmbH) as internal standard. The purity of all compounds tested in biological assays was ≥95% as determined by LC-MS.

Synthesis of the vinyl sulfone precursors[4]

3, Diethyl ((phenylsulfonyl)methyl)phosphonate

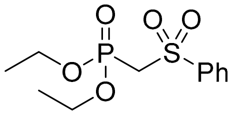

Methyl phenyl sulfone (1.5 g, 9.9 mmol, 1 eq) was dissolved in dry THF (30 mL) under argon atmosphere and cooled to 0 °C. Subsequently, *n*-BuLi (2.5 M in hexanes, 9.6 mL, 24 mmol, 2.5 eq) was added dropwise and the reaction mixture stirred for one hour at 0 °C. Afterwards, diethyl chlorophosphate (2.4 mL, 16 mmol 1.2 eq) was added and stirring was continued for one hour. The reaction was quenched with the addition of saturated NH_4Cl (10 mL) and diluted with DCM (30 mL). The aqueous phase was extracted with DCM (3x 20 mL) and the combined organic layers were dried over anhydrous Na_2SO_4. The solvent was removed under reduced pressure and the crude product was purified by column chromatography (CH/EA 4:1) to yield the desired product as a colorless solid (1.47 g, 5 mmol, 51%). ^{1}H NMR (300 MHz, $CDCl_3$): δ/ppm = 8.01 – 7.95 (m, 2H), 7.70 – 7.63 (m, 1H), 7.61 – 7.53 (m, 2H), 4.20 – 4.09 (m, 4H), 3.75 (d, J = 16.9 Hz, 2H), 1.28 (td, J = 7.1, 0.6 Hz, 6H). ^{13}C NMR (75 MHz, $CDCl_3$): δ/ppm = 140.0, 134.2, 129.2, 128.4, 63.5, 54.8, 52.9 (d, J = 6.5 Hz), 23.6 16.3. FT-IR: ν/cm^{-1} = 2988, 2898, 1324, 1257, 1155, 1018, 970, 796, 751, 683, MS (ESI) *m/z* calculated for $[C_{11}H_{18}O_5PS]^+$ ($[M+H]^+$): 293.1, found 293.0.

4, Diethyl (fluoro(phenylsulfonyl)methyl)phosphonate

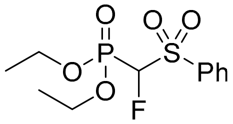

3 (1 g, 4.45 mmol, 1 eq) was dissolved in dry THF (15 mL) and cooled to −78 °C. LiHMDS (1 M in THF, 5.6 mL, 5.56 mmol, 1.25 eq) was added and stirring was continued for 30 min at −78 °C. Afterwards, Selectfluor® (2.4 g, 6.66 mmol, 1.5 eq) in DMF (10 mL) was added dropwise and the mixture stirred for two hours. The reaction was quenched with saturated NH_4Cl solution (15 mL) and diluted with EtOAc (30 mL). The aqueous phase was extracted with EtOAc (3x 20 mL) and the combined organic phases were dried over anhydrous Na_2SO_4 and the solvent was removed under reduced pressure. The crude product was purified by column chromatography (CH/EA 4:1) to yield the desired product as a colorless solid (0.81 g, 2.6 mmol, 58%). ^{1}H NMR (300 MHz, $CDCl_3$): δ/ppm = 8.03 – 7.96 (m, 1H), 7.75 – 7.68 (m, 1H), 7.60 (dd, J = 8.3, 6.9 Hz, 2H), 5.38 (dd, J = 45.5, 6.6 Hz, 1H), 4.35 – 4.19 (m, 4H), 1.34 (t, J = 7.1 Hz, 6H). ^{13}C NMR (75 MHz, $CDCl_3$): δ/ppm = 136.3, 135.0, 129.9, 129.3, 100.0, 97.9, 97.0, 94.9, 65.0 (t, J = 7.1 Hz), 16.41 (d, J = 5.9 Hz). mp: 61 – 65 °C. FT-IR: ν/cm^{-1} = 2990, 2907, 1449, 1331, 1263, 1221, 1159, 1061, 1006, 967, 852, 784, 755, 716, 699, 680. MS (ESI) *m/z* calculated for $[C_{11}H_{17}FO_5PS]^+$ ($[M+H]^+$): 311.0, found 311.0.

Synthesis of the *β*-lactam precursor[22]

6, (*S*)-2-(((Benzyloxy)carbonyl)amino)-3-hydroxypropanoic acid

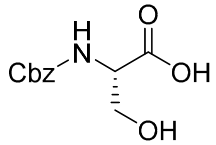

H-Ser-OH (1 g, 9.6 mol, 1 eq) and $NaHCO_3$ (2 g, 24 mmol, 2.5 eq) were dissolved in water (20 mL) and cooled to 0 °C. Benzyl chloroformate (2.01 mL, 14.4 mmol, 1.5 eq) was added dropwise. The reaction mixture was stirred for two hours at 0 °C and overnight at room temperature. The aqueous solution was acidified to pH = 2 with hydrochloric acid (1 M) and then extracted with EtOAc (3x, 10 mL). The combined organic layers were dried over Na_2SO_4, filtered and the solvent was removed under reduced pressure to yield the desired product as a colorless solid (2 g, 8.64 mmol, 90%). ^{1}H NMR (300 MHz, DMSO-d_6): δ/ppm = 7.40 – 7.25 (m, 5H), 5.04 (s, 2H), 4.06 (dt, J = 8.2, 5.1 Hz, 1H), 3.66 (d, J = 5.1 Hz, 2H). ^{13}C NMR (75 MHz, DMSO-d_6): δ/ppm = 172.2, 156.1, 137.0, 128.4, 127.8, 65.5, 61.3, 56.7. mp: 115 – 116 °C. $[\alpha]_D^{20}$ = +9 (c 1.00, DMSO). FT-IR: ν/cm^{-1} = 3320, 2935, 1710, 1690, 1567, 1477, 1285, 996, 821, 755. MS (ESI) *m/z* calculated for $[C_{11}H_{14}NO_5]^+$ ($[M+H]^+$): 240.1, found 262.1.

7, (*S*)-Benzyl (3-hydroxy-1-oxo-1-(phenylamino)propan-2-yl)carbamate

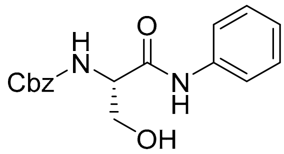

6 (3.26 g, 13.6 mmol, 1 eq) was dissolved in EtOAc (50 mL) under argon atmosphere and cooled to 0 °C. NMM (1.65 mL, 15 mmol, 1.1 eq) and TBTU (5.3 g, 16.3 mmol, 1.2 eq) were added and stirring was continued for 30 min at 0 °C. Afterwards, aniline (1.4 mL, 15 mmol, 1.1 eq) was added and the reaction mixture stirred for 12 h. The mixture was evaporated under reduced pressure and the crude product was purified by column chromatography (CH/EA 1:1) to yield the desired product as a colorless oil (3.32 g, 10.06 mmol, 74%). ^{1}H NMR (300 MHz, DMSO-d_6): δ/ppm = 7.65 – 7.58 (m, 2H), 7.39 – 7.25 (m, 7H), 7.09 – 7.01 (m, 1H), 5.07 – 5.03 (m, 2H), 4.94 (dt, J = 10.7, 5.8 Hz, 1H), 4.24 (dt, J = 7.9, 5.8 Hz, 1H), 3.66 (dq, J = 10.7, 5.5, 4.8 Hz, 2H). ^{13}C NMR (75 MHz, DMSO-d_6): δ/ppm = 169.1, 155.9, 138.9, 136.9, 128.6, 128.3, 127.8, 127.7, 123.3, 119.2, 65.5, 61.7, 57.7. $[\alpha]_D^{20}$ = +11 (c 1.00, DMSO). FT-IR: ν/cm^{-1} = 2241, 2108, 1740, 1689, 1510, 1341, 1049, 1015, 832, 754, MS (ESI) *m/z* calculated for $[C_{17}H_{19}N_2O_4]^+$ ($[M+H]^+$): 315.1, found 315.1.

8, (S)-Benzyl (2-oxo-1-phenylazetidin-3-yl)carbamate

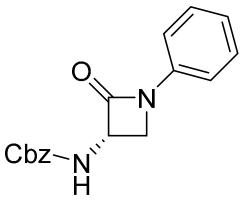

7 (1.5 g, 4.8 mmol, 1 eq) was dissolved in DMF (25 mL) under argon atmosphere and cooled to 0 °C. 1,1-sulfonyldiimidazole (1.42 g, 7.20 mmol, 1.5 eq) was added and stirred for 30 min at 0 °C. Afterwards, the reaction mixture was cooled to –20 °C and NaH (60%, 0.20 g, 7.20 mmol, 1.5 eq) was added portion wise. After additional 30 min of stirring, mixture of MeOH (0.04 mL) and water (20 mL) were added. The precipitate was filtered under reduced pressure and washed with cold water (15 mL) to yield the desired product as a white solid (1.1 g, 3.70 mmol, 77%). ^{1}H NMR (300 MHz, DMSO-d_6): δ/ppm = 8.09 (d, J = 8.4 Hz, 1H), 7.39 – 7.33 (m, 9H), 7.13 – 7.07 (m, 1H), 5.06 (s, 2H), 4.88 (ddd, J = 8.8, 5.8, 2.9 Hz, 1H), 3.94 (dt, J = 8.8, 5.8 Hz, 1H), 3.60 (dd, J = 5.8, 2.9 Hz, 2H). ^{13}C NMR (75 MHz, DMSO-d_6): δ/ppm = 164.7, 155, 138.0, 136.7, 129.2, 128.3, 127.9, 127.8, 123.6, 116.2, 65.7, 56.2, 46.4. $[\alpha]_D^{20}$ = –5 (*c* 1.00, DMSO). FT-IR: ν/cm^{-1} = 1754, 1512, 1501, 1380, 1341, 1254, 1023, 1004, 813, 756. MS (ESI) *m/z* calculated for $[C_{17}H_{17}N_2O_3]^+$ ($[M+H]^+$): 297.1, found 297.4.

9, (*S*)-3-Amino-1-phenylazetidin-2-one hydrochloride

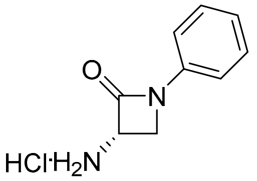

9 (0.5 g, 1.7 mmol, 1 eq) was dissolved in THF (5 mL). Subsequently, Pd/C (50 mg, 10%) was added, and the reaction mixture was stirred for two hours under a H_2 atmosphere (3 bar). Afterwards, the solution was filtered through Celite® 545 and acidified with hydrochloric acid (1 M, 7 mL). The desired product was obtained after removing the solvent under reduced pressure as a white solid (0.3 g, 1.5 mmol, 88%). ^{1}H NMR (300 MHz, DMSO-d_6): δ/ppm = 9.14 (s, 1H), 7.44 – 7.36 (m, 4H), 7.16 (ddt, J = 6.3, 5.2, 2.9 Hz, 1H), 4.67 (dd, J = 5.6, 2.5 Hz, 1H), 4.0 (dd, J = 6.7, 5.6 Hz, 1H), 3.74 (dd, J = 6.7, 2.6 Hz, 1H). ^{13}C NMR (75 MHz, DMSO-d_6): δ/ppm = 160.3, 137.4, 129.4, 124.5, 116.5, 53.5, 44.0. $[\alpha]_D^{20}$ = +17 (*c* 1.00, MeOH). FT-IR: ν/cm^{-1} = 1749, 1532, 1509, 1368, 1339, 1244, 1025, 1000, 813, 756. MS (ESI) *m/z* calculated for $[C_9H_{11}N_2O]^+$ ($[M+H]^+$): 163.1, found 163.0.

Synthesis of rhodesain targeting compounds

11, *tert*-Butyl (*S*)-(1-(methoxy(methyl)amino)-1-oxo-4-phenylbutan-2-yl) carbamate

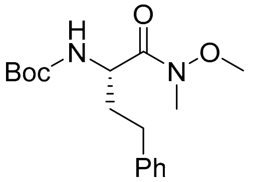

Boc-*h*Phe-OH **10** (8.07 g, 29.53 mmol, 1 eq) was dissolved in THF (165 mL) and the solution was cooled to 0 °C. DCC (7.20 g, 34.55 mmol, 1.17 eq), HOBt · H_2O(4.669 g, 34.557 mmol, 1.17 eq) and DIPEA (22 mL, 127.01 mmol, 4.3 eq) were added and the mixture was stirred for 15 min before *N*,*O*-dimethylhydroxylamine · HCl (3.439 g, 34.557 mmol, 1.17 eq) was added. The mixture was allowed to warm up to room temperature and was stirred overnight. Filtration of side product DCU was carried out several times (immersion of the solution in an ice bath to favour precipitation of DCU is recommended). The solvent was removed under reduced pressure, DCM was added (50 mL) and the organic phase was washed with hydrochloric acid (1 M, 3x 50 mL), saturated $NaHCO_3$ solution (3x 50 mL), H_2O (50 mL) and brine (50 mL). The organic phase was dried over anhydrous $MgSO_4$ and concentrated under vacuum. The crude product was purified by column chromatography (CH/EA 7:3) to obtain the pure product as a transparent oil (4.37 g, 13.58 mmol, 46%). ^{1}H NMR (300 MHz, $CDCl_3$): δ/ppm = 7.26 (m, 5H), 5.34 (m, 1H), 4.65 (m, 1H), 3.57 (s, 3H), 3.12 (s, 3H), 2.79 – 2.55 (m, 2H), 2.04 – 1.93 (m, 1H), 1.82 (m, 1H), 1.42 (s, 9H). ^{13}C NMR (101 MHz, $CDCl_3$): δ/ppm = 173.1, 155.7, 141.3, 128.6, 128.4, 126.1, 61.6, 53.5, 50.2, 34.7, 32.2, 31.8, 28.4. $[\alpha]_D^{20}$ =–7° (c = 10 mg/ mL, $CHCl_3$) . FT-IR: ν/cm^{-1} = 3321, 3050, 2974, 2933, 1707, 1655, 1495, 1454, 1445, 1390, 1245, 1163, 992, 701. MS (ESI) *m/z* calculated for $[C_{17}H_{26}N_2O_4Na]^+$ ($[M+Na]^+$): 345.2, found: 345.2.

12, *tert*-Butyl (*S*)-(1-oxo-4-phenylbutan-2-yl) carbamate

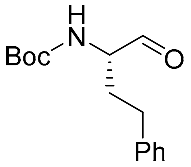

A solution of **11** (1.97 g, 6.13 mmol, 1 eq) in THF (40 mL) was placed in an ice bath. $LiAlH_4$ (0.73 g, 18.41 mmol, 3 eq) was slowly added. The reaction mixture was stirred for two hours and subsequently quenched by addition of aqueous saturated Rochelle salt solution (50 mL). The organic phases were extracted with EtOAc (3x 50 mL), washed with brine (50 mL), dried over anhydrous $MgSO_4$ and the solvent was removed under reduced pressure. Purification by column chromatography (CH/EA 8:2) was performed and the pure product was obtained as a transparent oil (1.08 g, 4.11 mmol, 67%). ^{1}H NMR (300 MHz, $CDCl_3$): δ/ppm = 9.45 (s, 1H), 7.17 (m, 5H), 5.33 – 4.80 (m, 1H), 4.35 – 2.59 (m, 1H), 2.62 (t, J = 7.9 Hz, 2H), 2.21 – 2.04 (m, 1H), 1.79 (m, 1H), 1.38 (s, 9H). ^{13}C NMR (101 MHz, $CDCl_3$): δ/ppm = 199.6, 155.6, 140.6, 128.6, 128.5, 126.4, 59.6, 31.5, 30.9, 28.3, FT-IR: ν/cm^{-1} = 3347, 3027, 2978, 2930, 2863,

1685, 1498, 1450, 1380, 1249, 746, 701. MS (ESI) *m/z* calculated for $[C_{15}H_{21}NO_3Na]^+$ ($[M+Na]^+$): 286.1, found: 286.1.

13, Benzyl ((*S*)-1-(((*S,E*)-1-nitro-5-phenylpent-1-en-3-yl)amino)-1-oxo-3-phenylpropan-2-yl)carbamate

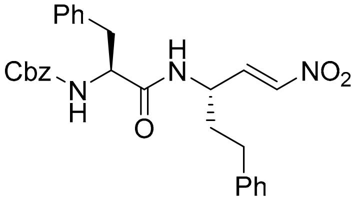

To an ice-bath cold solution of **12** (0.44 g, 1 mmol, 1 eq) in DCM (1 mL) was added nitromethane (0.36 g, 6 mmol, 6 eq) and Et_3N (42 mL, 0.3 mmol, 0.3 eq). The resulting mixture was stirred at 23 ºC for eight hours and then was quenched with saturated NH_4Cl solution (25 mL) and extracted with DCM (3x 15 mL), the organic layers were washed with hydrochloric acid (1 M, 15 mL), then with saturated $NaHCO_3$ solution (15 mL) and then with brine (15 mL), dried over anhydrous Na_2SO_4 and concentrated under reduced pressure. The crude oil was directly submitted to the next step without any further purification. The resulting mixture of nitroaldols was dissolved in DCM (1 mL), cooled with an ice-bath and then trifluoroacetic acid (1.5 mL, 20 mmol) was added. The resulting mixture was stirred at 23 ºC for 30 min and then it was directly concentrated under vacuum. The resulting crude was dissolved in DCM (10 mL). Then Cbz-Phe-OH **19** (1.1 mmol), HOBt · H_2O (168 mg, 1.1 mmol), Et_3N (558 mL, 4 mmol) and EDC · HCl (211 mg, 1.1 mmol) were sequentially added. The resulting mixture was stirred at 23 ºC for eight hours and then was quenched with saturated NH_4Cl solution (25 mL) and extracted with DCM (3x 15 mL), the organic layers were washed with hydrochloric acid (1 M, 15 mL), then with saturated $NaHCO_3$ solution (15 mL) and then with brine (15 mL), dried over anhydrous Na_2SO_4 and concentrated under reduced pressure. The crude oil was directly submitted to the next step without any further purification. The resulting diastereomeric mixture of dipeptidyl nitroaldols was dissolved in DCM (10 mL). The resulting mixture was treated with DIPEA (697 µL, 4 mmol) and MsCl (155 µL, 2 mmol) and then stirred at 23 ºC for two hours. It was quenched with saturated NH_4Cl solution (25 mL) and extracted with DCM (3x 15 mL), the organic layers were washed with hydrochloric acid (1 M, 15 mL), then with saturated $NaHCO_3$ solution (15 mL) and then with brine (15 mL), dried over Na_2SO_4 and concentrated. The crude oil was purified by column chromatography (CH/EA 9:1 and 7:3) to afford desired product as a colorless solid (0.37 g, 0.75 mmol, 75%). ^{1}H NMR (500 MHz, $CDCl_3$): δ/ppm = 7.41 – 7.13 (m, 15H), 6.87 (dd, J = 13.2, 5.7 Hz, 2H), 6.64 (d, J = 13.5 Hz, 1H), 6.21 (br s, 1H), 5.50 (br s, 1H), 5.08 (s, 2H), 4.59 (m, 1H), 4.41 (m, 1H), 3.22 – 2.92 (m, 2H), 2.62 – 2.39 (m, 2H), 1.92 – 1.64 (m, 2H). ^{13}C NMR (75 MHz, $CDCl_3$): δ/ppm = 170.7, 156.2, 141.0, 140.5, 140.1, 135.9, 129.2, 128.8, 128.6, 128.5, 128.3, 128.2, 127.9, 127.5, 126.4, 67.2, 56.7, 47.1, 38.4, 35.2, 35.2, 31.7. $[\alpha]_D^{20}$ = + 0.2 (*c* 0.50, EtOAc). FT-IR: ν/cm^{-1} = 3289, 3091, 3058, 3031, 2925, 2860, 1951, 1882, 1688, 1655, 1525, 1453, 1358, 1285, 1236, 1179, 1035, 955, 750, 700. MS (ESI) *m/z* calculated for $[C_{28}H_{30}N_3O_5]^+$ ($[M+H]^+$): 510.2, found: 510.1. Purity: 97%.

14, *tert*-Butyl (*S,E*)-(5-phenyl-1-(phenylsulfonyl)pent-1-en-3-yl)carbamate

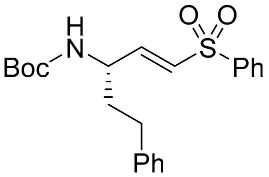

3 (0.25 g, 0.85 mmol, 1.5 eq) was dissolved in dry THF (3 mL) and cooled to −78 °C. Under nitrogen atmosphere, LiHMDS (1 M in hexanes, 0.85 mL, 0.85 mmol, 1.5 eq) was added dropwise and the mixture was stirred 30 min at −78 °C before a solution of **12** (0.15 g, 0.57 mmol, 1 eq) in dry THF (1.6 mL) was added. The mixture was stirred overnight and THF was removed under reduced pressure. The residue was dissolved in DCM (20 ml) and washed with water (10 ml), saturated $NaHCO_3$ solution (2x, 10 ml) and brine (10 ml). The organic phase was dried over anhydrous Na_2SO_4 and concentrated under reduced pressure. The crude was purified by column chromatography (CH/EA 7:3). The pure product was obtained as a colorless solid (0.14 g, 0.34 mmol, 59%). ^{1}H NMR (400 MHz, $CDCl_3$): δ/ppm = 7.89 – 7.84 (m, 2H), 7.63 – 7.58 (m, 1H), 7.53 (m, J = 8.2, 1.4 Hz, 2H), 7.30 – 7.26 (m, 2H), 7.19 (m, J = 8.3, 6.1 Hz, 1H), 7.15 – 7.11 (m, 2H), 6.89 (dd, J = 15.0, 4.7 Hz, 1H), 6.43 (d, J = 15.1 Hz, 1H), 4.61 (d, J = 7.9 Hz, 1H), 4.36 (s, 1H), 2.73 – 2.62 (m, 2H), 2.00 – 1.77 (m, 2H), 1.44 (dd, J = 13.3, 11.8 Hz, 8H). ^{13}C NMR (75 MHz, DMSO-d_6): δ/ppm = 155.0, 147.3, 141.1, 140.3, 133.6, 129.8, 129.6, 128.3, 128.3, 127.1, 125.8, 78.2, 50.4, 34.8, 31.5, 28.1. MS (ESI) *m/z* calculated for $[C_{22}H_{27}NO_4S]^+$ ($[M+Na]^+$): 424.2, found: 424.2.

15, *tert*-Butyl (*S,E*)-(1-fluoro-5-phenyl-1-(phenylsulfonyl)pent-1-en-3-yl)carbamate

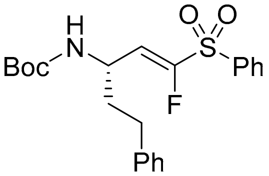

4 (0.265 g, 0.855 mmol, 1.5 eq) was dissolved in dry THF (3 mL) and cooled to −78 °C. Under nitrogen atmosphere, LiHMDS (1 M in hexanes, 0.85 mL, 0.85 mmol, 1.5 eq) was added dropwise and the mixture was stirred 30 min at −78 °C before a solution of **12** (0.150 g, 0.570 mmol, 1 eq) in dry THF (1.6 0mL) was added. The mixture was stirred overnight and THF was removed under reduced pressure. The residue was dissolved in DCM (20 ml) and washed with water (10 ml), saturated $NaHCO_3$ solution (2x, 10 ml) and brine (10 ml). The organic phase was dried with anhydrous Na_2SO_4 and concentrated under reduced pressure. The product was purified by column chromatography (CH/EA 7:3). The pure product was obtained as a colorless solid (0.19 g, 0.32 mmol, 57%). ^{1}H NMR (400 MHz, $CDCl_3$): δ/ppm = 8.02 – 7.91 (m, 2H), 7.76 – 7.66 (m, 1H), 7.65 – 7.55 (m, 2H), 7.29 – 7.09 (m, 5H), 6.19 (d, J = 30.7 Hz, 1H), 4.61 (s, 1H), 4.46 (s, 1H), 2.71 – 2.57 (m, 2H), 1.94 (m, 2H), 1.60 – 1.23 (m, 9H). ^{13}C NMR (75 MHz, $CDCl_3$): δ/ppm = 154.8, 140.3, 137.1, 134.8, 134.5, 129.4, 129.0, 128.6, 128.5, 128.3, 126.2, 118.3, 118.3, 77.4, 77.2, 77.0, 76.6, 46.3, 36.0, 31.8, 28.2. MS (ESI) *m/z* calculated for $[C_{22}H_{26}FNO_4S]^+$ ($[M+H]^+$): 442.1, found: 442.1.

16, Benzyl ((*S*)-1-oxo-3-phenyl-1-(((*S*,*E*)-5-phenyl-1-(phenylsulfonyl)pent-1-en-3-yl)amino)propan-2-yl)carbamate

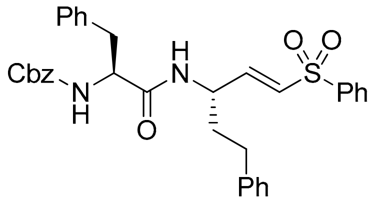

14 (0.13 g, 0.33 mmol, 1 eq) was dissolved in DCM (2 mL) and a mixture of TFA and DCM (1:1, 1 ml) was slowly added under cooling in an ice bath. The mixture was allowed to stir until for two hours. The solvent was removed under reduced pressure and the product was used in the following reaction without further purification. The deprotected vinylsulfone (0.33 mmol, 1 eq) and Cbz-Phe-OH **19** (0.15 g, 0.50 mmol, 1.5 eq) were dissolved in DMF (6.7 mL) and DIPEA (0.26 mL, 1.50 mmol, 4.5 eq) was added. The mixture was cooled in an ice bath and T3P (50% in DMF, 0.6 mL, 1.0 mmol, 3 eq) was added dropwise. The mixture was stirred overnight at room temperature. Water (20 mL) was added and the mixture was extracted with EtOAc (3x 10 ml). The combined organic layers were washed with a saturated $NaHCO_3$ solution (15 ml) and brine (15 ml), dried over anhydrous Na_2SO_4, concentrated under reduced pressure and purified by column chromatography (CH/EA 1:1). The pure product was obtained as a colorless solid (0.12 g, 0.21 mmol, 65%). ^{1}H NMR (400 MHz, $CDCl_3$): δ/ppm = 7.77 (d, J = 7.5 Hz, 2H), 7.55 (t, J = 7.4 Hz, 1H), 7.47 (t, J = 7.6 Hz, 2H), 7.25 – 6.95 (m, 15H), 6.69 (dd, J = 15.1, 4.8 Hz, 1H), 6.00 (dd, J = 15.1, 1.6 Hz, 1H), 5.76 (br s, 1H), 5.12 (br s, 1H), 4.99 (s, 2H), 4.57 (m, 1H), 4.21 (m, 1H), 2.95 (dd, J = 13.7, 6.4 Hz, 1H), 2.91 (dd, J = 13.7, 8.0 Hz, 1H), 2.47 (m, 2H), 1.80 (m, 1H), 1.67 (m, 1H) ppm; ^{13}C NMR (101 MHz, $CDCl_3$): δ/ppm = 170.5, 156.0, 145.1, 140.3, 140.1, 136.0, 135.9, 133.5, 130.7, 129.3, 129.2, 128.9, 128.7, 128.6, 128.4, 128.3, 128.2, 127.7, 127.4, 126.4, 67.3, 56.6, 49.3, 38.1, 35.4, 31.8. %). $[\alpha]_D^{20}$ = +0.06 (*c* 1.3, DCM). mp: 186 – 188 °C. FT-IR: *v*/cm^{-1} = 3279, 3061, 3030, 2948, 2923, 2852, 1685, 1653, 1520, 1283. MS (ESI) *m/z* calculated for $[C_{34}H_{34}N_2O_5SNa]^+$ ($[M+Na]^+$): 605.2, found: 605.2. Purity: 99%

17, Benzyl ((*S*)-1-(((*S*,*E*)-1-fluoro-5-phenyl-1-(phenylsulfonyl)pent-1-en-3-yl)amino)-1-oxo-3-phenylpropan-2-yl)carbamate

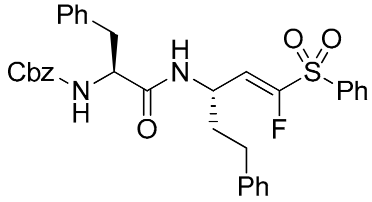

15 (0.13 g, 0.32 mmol, 1 eq) was dissolved in DCM (2 mL) and a mixture of TFA and DCM (1:1, 1 ml) was slowly added under cooling in an ice bath. The mixture was allowed to stir for two hours. The volatiles were removed under reduced pressure and the product was used in the following reaction without further purification. The deprotected vinylsulfone (0.32 mmol, 1 eq)

and Cbz-Phe-OH (0.14 g, 0.49 mmol, 1.5 eq) were dissolved in DMF (6.5 mL) and DIPEA (0.26 mL, 1.47 mmol, 4.5 eq) was added. The mixture was placed under an ice bath and T3P (50% in DMF, 0.58 mL, 0.98 mmol, 3 eq) was added dropwise. The mixture was stirred overnight at room temperature. Water (20 mL) was added and the mixture was extracted with EtOAc (3x 10 ml). The combined organic layers were washed with a saturated $NaHCO_3$ solution (15 ml) and brine (15 ml), dried over anhydrous Na_2SO_4, concentrated under reduced pressure and purified by column chromatography (CH/EA 7:3 to 1:1). The pure product was obtained as a colorless solid (0.08 g, 0.15 mmol, 48%). ^{1}H NMR (400 MHz, $CDCl_3$): δ/ppm = 7.99 – 7.87 (m, 2H), 7.76 – 7.65 (m, 1H), 7.65 – 7.53 (m, 2H), 7.38 – 6.95 (m, 15H), 5.97 (dd, J = 32.0, 8.6 Hz, 15H), 5.71 (broad s, 1H), 5.19 (broad s, 1H), 5.12 – 5.03 (m, 2H), 4.67 (m, 1H), 4.26 (m, 1H), 3.15 – 2.89 (m, 2H), 2.49 (t, J = 7.6 Hz, 2H), 1.91 – 1.68 (m, 2H). ^{13}C NMR (101 MHz, $CDCl_3$): δ/ppm = 170.3, 156.5, 153.5, 140.4, 137.1, 136.1, 134.8, 129.7, 129.4, 129.1, 128.8, 128.8, 128.8, 128.5, 128.4, 128.3, 128.3, 127.5, 126.5, 117.2, 67.4, 56.6, 53.6, 45.1, 35.7, 31.8. FT-IR: ν/cm^{-1} = 3290, 3061, 3029, 2925, 2858, 1655, 1525, 1496, 1332, 1158. MS (ESI) *m/z* calculated for $[C_{34}H_{34}FN2O_5S]^+$ ($[M+H]^+$): 601.2, found:601.1. Purity: 99%

18, Benzyl ((*S*)-1-(((*S*)-1-(benzo[*d*]thiazol-2-yl)-1-oxo-4-phenylbutan-2-yl) amino)-1-oxo-3-phenylpropan-2-yl)carbamate

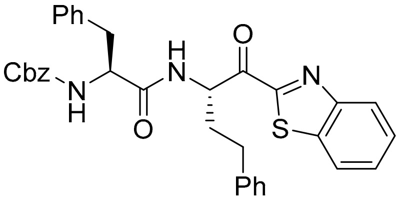

To a solution of benzothiazole (1.45 ml, 244.44 mmol, 18 eq) in dry THF (30 mL), *n*-BuLi (2.5 M in THF, 95 ml, 237.65 mmol, 17.5 equiv.) was added dropwise at −78 °C under argon atmosphere. After the mixture was stirred for additional 30 min at the same temperature, **11** (4.37 g, 13.58 mmol, 1 eq) was dissolved in THF (8 mL) and added dropwise. The mixture was stirred at −78 °C for 3 h. The reaction was quenched with saturated NH_4Cl (15 ml) and the aqueous layer was extracted with EtOAc (3x, 10 ml). The combined organic layers were dried over anhydrous Na_2SO_4, and then concentrated under reduced pressure. The resulting residue was dissolved in DCM (2 mL) and a mixture of TFA and DCM (1:1, 1 ml) was slowly added under cooling in an ice bath. The mixture was allowed to stir for two hours. The volatiles were removed under reduced pressure and the product was used in the following reaction without further purification. The resulting crude product was dissolved in dichloromethane (10 ml). Then Cbz-Phe-OH **19** (4.47 g, 14.94 mmol, 1.1 eq), HOBt · H_2O (2.28 g, 14.94 mmol, 1.1 eq) Et_3N (7.53 ml, 54.32 mmol, 4 eq) and EDC · HCl (2.86 g, 14.94 mmol, 1.1 eq) were sequentially added. The resulting mixture was stirred at 23 ºC for eight hours and then was quenched with saturated NH_4Cl solution (25 ml) and extracted with DCM (3x, 15 ml), the combined organic layers were washed with hydrochloric acid (1 M, 10 ml), then with saturated $NaHCO_3$ solution (10 ml) and then with brine, dried over anhydrous Na_2SO_4 and concentrated under reduced pressure. The crude oil was purified by column chromatography (CH/EA 9:1 to 2:8) to afford desired product as a colorless solid (4.39 g, 7.60 mmol, 56%). ^{1}H NMR (300

MHz, $CDCl_3$): *δ*/ppm = 8.10 - 8.05 (m, 1H), 7.92 – 7.87 (m, 1H), 7.54 – 7.44 (m, 2H), 7.24 – 6.98 (m, 14H), 6.76 – 6.70 (m, 1H), 5.75 – 5.63 (m, 1H), 5.27 (br s, 1H), 5.02 (s, 2H), 4.48 – 4.37 (m, 1H), 3.03 – 2.91 (m, 2H), 2.59 – 1.89 (m, 4H). ^{13}C NMR (75 MHz, CDCl3): *δ*/ppm = 192.5, 170.6, 163.6, 155.9, 153.4, 140.7, 140.5, 137.2, 136.3, 129.3, 128.8, 128.7, 128.6, 128.4, 128.2, 128.1, 127.2, 127.1, 126.2, 125.9, 122.3, 67.2, 56.0, 55.7, 38.6, 34.2, 31.5 mp: 172 – 175 ºC. $[\alpha]_D^{20}$ = –9 (*c* 1.1, DCM). FT-IR: *ν*/cm^{-1} = 3291, 3060, 3027, 2922, 1692, 1648, 1528, 1286, 1238. MS (ESI) *m/z* calculated for $[C_{34}H_{31}N_3O_4SNa]^+$ ($[M+H]^+$): 600.2, found: 600.2. Purity: 97%

20, Methyl (*S*)-2-((*S*)-2-(((benzyloxy)carbonyl)amino)-3-phenylpropanamido)-4-phenylbutanoate

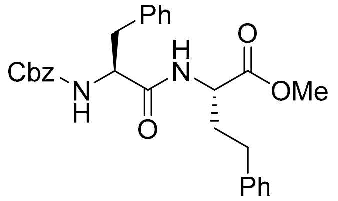

H-*h*Phe-OMe **10** (2.4 g, 10 mmol, 1.0 eq) was coupled with Cbz-Phe-OH **19** (3.0 g, 10 mmol, 1.0 eq), HATU (4.94 g, 13.0 mmol, 1.2 eq) and 2,4,6-collidine (3.9 mL, 30 mmol, 3.0 eq) in a mixture of DCM and DMF (1:1, 30 mL). After stirring for 16 h water (30 mL) was added and the aqueous phase was extracted with EtOAc (3x 25 mL). The combined organic layers were washed with brine (15 mL) and water (20 mL) dried over anhydrous $NaSO_4$ and concentrated under vacuum. The crude product was purified by column chromatography (CH/EA 3:1) to give the desired product as a colorless oil (5.3 g, 10.0 mmol, quant.). ^{1}H-NMR (300 MHz, $CDCl_3$): *δ*/ppm = 7.39 – 7.00 (m, 5H), 6.43 (d, *J* = 7.7 Hz, 1H), 5.28 (d, *J* = 8.0 Hz, 1H), 5.10 (s, 2H), 4.65 – 4.52 (m, 1H), 4.50 – 4.34 (m, 1H), 4.24 – 3.98 (m, 2H), 3.08 (d, *J* = 6.7 Hz, 2H), 2.65 – 2.42 (m, 2H), 2.32 – 1.80 (m, 2H), 1.27 (t, *J* = 7.1 Hz, 3H). ^{13}C-NMR (75.5 MHz, $CDCl_3$): *δ*/ppm = 170.6, 170.7, 156.0, 140.8, 136.3, 136.2, 129.5, 128.8, 128.7, 128.6, 128.5, 128.3, 128.2, 127.2, 126.3, 67.3, 61.7, 56.3, 52.4, 38.4, 33.9, 31.6, 14.3. $[\alpha]_D^{22}$ = +20 (*c* 1.00 , $CHCl_3$). FT-IR: *ν*/cm^{-1} = 3300, 2359, 1732, 1690, 1653, 1531, 1454, 1287, 1038, 747. MS (ESI) *m/z* calculated for $[C_{28}H_{30}N_2O_5]^+$ ($[M+H]^+$): 474.2, found.: 474.2.

21, Benzyl ((*S*)-1-(((*S*)-1-(dimethoxyphosphoryl)-2-oxo-5-phenylpentan-3-yl)amino)-1-oxo-3-phenylpropan-2-yl)carbamate

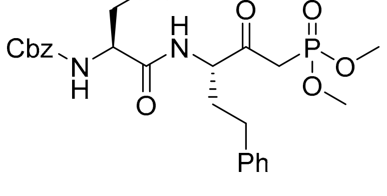

To a −78 °C cold solution of dimethyl methylphosphonate (2.08 mL, 19.17 mmol, 8 eq) in THF (20 mL) was added *n*-BuLi (1.6 M in hexanes ,1.98 mL, 19.17 mmol, 8 eq). The resulting mixture was stirred cooled at −78 °C for 15 min and then a solution of **20** (1.13 g, 2.40 mmol,

1 eq) in THF (20 mL) was added dropwise. The resulting mixture was stirred at −78 °C for two hour and then was quenched with diluted AcOH (10% in water, 20 mL) and extracted with EtOAc (3x 30 mL), the organic layers were washed with saturated $NaHCO_3$ solution (20 mL), dried over anhydrous Na_2SO_4 and concentrated under reduced pressure. The crude oil was directly submitted to the next step without any further purification (1.33 g, 2.35 mmol, 98%). ^{1}H NMR (300 MHz, $CDCl_3$): δ/ppm = 7.48 (d, J = 7.8 Hz, 1H), 7.27–7.08 (m, 15H), 5.68 (d, J = 7.2 Hz, 1H), 5.07 (d, J = 12.6 Hz, 1H), 5.01 (d, J = 12.3 Hz, 1H), 4.63–4.53 (m, 2H), 3.71 (s, 3H), 3.67 (s, 3H), 3.18–2.91 (m, 2H), 2.53 (t, J = 7.8 Hz, 2H), 2.24–2.13 (m, 1H), 1.89–1.80 (m, 1H). ^{13}C NMR (75 MHz, $CDCl_3$): δ/ppm = 200.3, 170.9, 156.1, 140.7, 136.2, 129.2, 128.6, 128.4, 128.0, 127.9, 126.9, 126.0, 66.9, 58.7, 56.2, 53.1, 53.0, 38.6, 36.9, 31.9, 31.5.

22, Ethyl (*S,E*)-5-((*S*)-2-(((benzyloxy)carbonyl)amino)-3-phenylpropanamido)-4-oxo-7-phenylhept-2-enoate

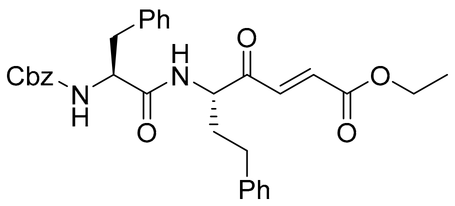

To a stirred solution of **21** (0.56 g, 1 mmol, 1 eq) in EtOH (7.5 mL) were added K_2CO_3 (0.14 g, 1 mmol, 1 eq) and over P_2O_5 freshly distilled ethyl glyoxylate (0.1 g, 1 mmol, 1 eq). The resulting mixture was stirred at room temperature for two hours and then was filtered off, neutralized using AcOH and concentrated under reduced pressure. The crude oil was purified by column chromatography (CH/EA 7:3) to afford the desired product as a colorless solid (0.41 g ,0.76 mmol ,76%). ^{1}H NMR (400 MHz, $CDCl_3$): δ/ppm = 7.35–7.06 (m, 16 H), 6.72 (d, *J*=16.0 Hz, 1 H), 6.50 (br s, 1 H), 5.21 (br s, 1 H), 5.13 (d, *J*=12.5 Hz, 2 H), 5.09 (d, *J*=12.0 Hz, 2 H), 4.84 (q, *J*=7.0 Hz, 1 H), 4.44 (m, 1 H), 4.28 (q, *J*=7.5 Hz, 2 H), 3.07 (m, 2 H), 2.45–2.58 (m, 2 H), 2.18–2.25 (m, 1 H), 1.83–1.90 (m, 1 H), 1.34 (t, *J*=7.5 Hz, 3 H). ^{13}C NMR (125 MHz, $CDCl_3$): δ/ppm = 196.3, 170.7, 165.0, 155.9, 140.3, 136.2, 135.8, 132.7, 129.3, 128.7, 128.6, 128.5, 128.4, 128.2, 128.0, 127.1, 126.4, 67.2, 61.7, 56.9, 56.2, 38.4, 32.7, 31.2, 14.1. mp: 115 – 119 °C. $[\alpha]_D^{22}$ = −12 (*c* 0.10, $CHCl_3$). FT-IR: ν/cm^{-1} = 3619, 3019, 2896, 2399, 1716, 1518, 1385, 1213, 1046, 928, 746, 734, 669, 627. MS (ESI) *m*/*z* calculated for $[C_{32}H_{35}O_6N_2]^+$ ($[M+H]^+$): 543.2, found: 543.2. Purity: 99%.

23, (*S*)-2-((*S*)-2-(((benzyloxy)carbonyl)amino)-3-phenylpropanamido)-4-phenylbutanoic acid

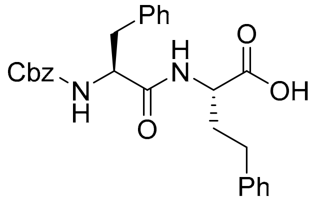

20 (5.0 g, 10 mmol, 1.0 eq) with LiOH (1.26 g, 30.0 mmol, 3.00 eq) in a mixture of water and THF (1:1, 30 mL). was stirred for 18 h at room temperature. After the reaction was complete as indicated by TLC, the solvent was evaporated under reduced pressure and to the resulting crude product was added hydrochloric acid (1.0 M) until pH = 3 was reached. The mixture was then extracted with EtOAc (3x 20 mL) and after phase separation, the combined organic layers were dried over anhydrous Na_2SO_4. The solvent was evaporated under reduced pressure yielding the target carboxylic acid as a colorless solid (4.57 g, 9.92 mmol, quant.). ^{1}H-NMR (300 MHz, $CDCl_3$): δ/ppm = 8.37 (d, J = 7.9 Hz, 1H), 7.60 – 7.39 (m, 1H), 7.41 – 6.95 (m, 15H), 5.09 – 4.81 (m, 2H), 4.42 – 4.31 (m, 1H), 4.26 – 4.14 (m, 1H), 3.05 (dd, J = 13.9, 3.8 Hz, 1H), 2.84 – 2.72 (m, 1H), 2.71 – 2.56 (m, 2H), 2.13 – 1.76 (m, 2H). ^{13}C-NMR (75 MHz, $CDCl_3$): δ/ppm = 173.5, 171.8, 55.9, 141.0, 138.1, 137.0, 129.3, 128.4, 2 × 128.3, 128.2, 128.1, 127.7, 127.4, 126.3, 125.9, 65.2, 56.0, 51.3, 37.3, 32.9, 31.3. $[\alpha]_D^{22}$ = +16 (c 1.00, ACN). FT-IR: ν/cm^{-1} = 3299, 3029, 2359, 1699, 1643, 1497, 1243, 1028, 744, 698. MS (ESI) *m/z* calculated for $[C_{27}H_{29}N_2O_5]^+$ ($[M+H]^+$): 461.2, found: 461.2.

24, Benzyl ((*S*)-1-oxo-1-(((*S*)-1-oxo-1-(((*S*)-2-oxo-1-phenylazetidin-3-yl)amino)-4-phenylbutan-2-yl)amino)-3-phenylpropan-2-yl)carbamate

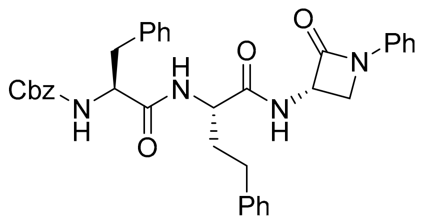

23 (138 mg, 0.30 mmol, 1.0 eq) was coupled with **9** (60 mg, 0.3 mmol, 1.0 eq), HATU (136.9 mg, 0.360 mmol, 1.2 eq) and 2,4,6-collidine (0.12 mL; 0.91 mmol; 3.0 eq). After stirring overnight, water (30 mL) was added, and the aqueous phase was extracted with EtOAc (3x 25 mL). The combined organic extracts were washed with brine (15 mL) and water (20 mL), dried over anhydrous $NaSO_4$ and concentrated under reduced pressure. The crude product was purified by preparative HPLC yielding the desired product as a colorless solid (120 mg, 0.20 mmol, 67%). ^{1}H-NMR (300 MHz, DMSO-d_6): δ/ppm =8.65 (d, J = 8.4 Hz, 1H), 8.25 (d, J = 7.8 Hz, 1H), 7.56 (d, J = 8.5 Hz, 1H), 7.44 – 6.99 (m, 20H), 5.15 – 5.01 (m, 1H), 4.96 (s, 2H), 4.49 – 4.26 (m, 2H), 3.95 (t, J = 5.8 Hz,1H), 3.65 – 3.53 (m, 1H), 3.15 – 3.00 (m, 1H), 2.89 – 2.74 (m, 1H), 2.73 – 2.57 (m, 2H), 2.10 – 1.83 (m, 2H). ^{13}C-NMR, HSQC, HMBC (75.5 MHz, DMSO-d_6): δ/ppm = 164.5, 155.9, 141.3, 138.1, 137.0, 126.3, 125.9, 123.7, 116.2, 65.3, 56.1, 54.9, 52.2, 46.2, 37.2, 33.9, 31.2. mp: 240°C (decomposition). $[\alpha]_D^{22}$ = +13 (c 1.00, DMSO). FT-IR: ν/cm^{-1} = 3305, 2925, 1744, 1685, 1650, 1528, 1389, 1285, 1226, 1037. MS (ESI) *m/z* calculated for $[C_{36}H_{36}N_4O_5Na]^+$ ($[M+Na]^+$): 627.3 , found: 627.3. Purity: 98%.

25, Benzyl ((*S*)-1-(((*S*)-1-cyano-3-phenylpropyl)amino)-1-oxo-3-phenylpropan-2-yl)carbamate

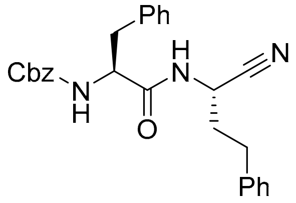

23 (0.15 g, 0.32 mmol, 1 eq) was dissolved in DMF (3 mL) and the solution was cooled to 0 °C. EDC · HCl (0.07 g, 0.39 mmol, 1.2 eq) and HOBt · H_2O (0.05 g, 0.39 mmol, 1.2 eq) were added and the mixture was stirred for 30 min before NH_4OH solution (25% in water, 3 mL) was added. The mixture was allowed to warm up to room temperature and was stirred overnight. The solvent was removed under reduced pressure and EtOAc (5 mL) was added. The organic phase was washed with saturated $NaHCO_3$ solution (5 mL) and hydrochloric acid (1 M, 5 mL), dried over anhydrous Na_2SO_4 and evaporated under reduced pressure. The crude product was used without further purification in the next step and solved in dry DMF (3 mL) under argon atmosphere. The solution cooled to 0 °C, pyridine (0.08 mL, 0.98 mmol, 3 eq) and TFA anhydride (0.14 mL, 0.98 mmol, 3 eq) were added and the reaction mixture stirred for 60 min. Subsequently, the reaction was quenched with saturated NH_4Cl solution (5 mL) and evaporated under reduced pressure. The crude product was purified by preparative HPLC to yield the desired product as a colorless solid (83.7 mg, 0.19 mmol, 60%). ^1H NMR (600 MHz, $CDCl_3$): δ/ppm = 7.35 – 7.03 (m, 15H), 6.35 (s, 1H), 5.32 – 5.22 (m, 1H), 5.10 – 5.01 (m, 2H), 4.77 – 4.66 (m, 1H), 4.41 – 4.29 (m, 1H), 3.10 – 2.98 (m, 2H), 2.66 (dt, *J* = 29.3, 7.1 Hz, 2H), 1.98 (h, *J* = 7.1, 6.6 Hz, 2H) ppm. ^{13}C NMR (600 MHz, $CDCl_3$): δ/ppm = 170.6, 156.2, 139.1, 135.8, 129.4, 129.1, 128.9, 128.7, 128.5, 128.2, 127.5, 126.8, 117.8, 67.5, 56.2, 40.1, 38.3, 34.3, 31.4. FT-IR: ν/cm^{-1} = 3294, 3024, 1667, 1529, 1445, 1254, 1035, 894, 748, 689. MS (ESI) *m/z* calculated. for $[C_{27}H_{28}N_3O_3]^+$ ($[M+H]^+$): 442.2, found: 442.0. Purity: 97%.

Synthesis of Cathepsin S targeting compounds

General procedures

A Boc deprotection

The Boc-protected amino acid or dipeptide (1.0 eq) was dissolved in 2 mL 1,4-Dioxan and 3 mL of a 4.0 M HCl-solution in 1,4-Dioxan was added dropwise. The mixture was stirred at room temperature until completion of the deprotection could be observed via TLC monitoring. The solvent was then removed under reduced pressure and the deprotected target compound could be obtained and was used in the next step without further purification and characterization.

B Ester cleavage with LiOH

The ester (1.0 eq) was dissolved in THF (20 mL) and mixed with a solution of LiOH (3.0 eq) in water (20 mL). The resulting mixture was stirred for 18 h at room temperature. After the reaction was complete as indicated by TLC, the solvent was evaporated under reduced pressure and to the resulting crude product was added hydrochloric acid (1.0 M) until pH = 3 was reached. The mixture was then extracted with EtOAc (3x 20 mL) and after phase separation, the combined organic layers were dried over anhydrous Na_2SO_4. The solvent was evaporated under reduced pressure yielding the target carboxylic acid as a colorless oil or a colorless solid.

C: Peptide coupling with HATU

The carboxylic acid (1.0 eq) was dissolved in a mixture of DCM/DMF (9:1) at 0 °C. Under stirring HATU (1.2 eq) was added in portions. Afterwards 2,4,6- collidine (3.0 eq) was added and stirred for an additional 10 min at 0 °C. The amine (1.0 eq) was added in portions or dropwise diluted in DCM and the reaction mixture was allowed to reach room temperature and stirred overnight. H_2O (30 mL) was added, and the organic phase separated. The aqueous phase was extracted with EtOAc (3x 20 mL). The combined organic extracts were washed with brine (40 mL) and dried over anhydrous $NaSO_4$. The solvent was evaporated under reduced pressure to give a crude product that was purified using either column chromatography or via preparative HPLC.

27, Cyclohexylalanine methylester hydrochloride

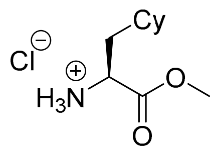

H-Cha-OH **26** (0.51 g, 2.92 mmol, 1.0 eq) was dissolved in dry MeOH (20 mL) and cooled to 10 °C. $SOCl_2$ was added dropwise, and the mixture was allowed to reach room temperature and was stirred overnight. After completion of the reaction, indicated by TLC monitoring, the solvent was evaporated under reduced pressure. EtOAc (20 mL) was added to the residue and

after filtration the methyl ester was obtained as a colorless solid (0.59 g, 2.65 mmol, 91%). ^{1}H NMR (300 MHz, $CDCl_3$): δ/ppm = 8.81 (s, 3H), 4.10 (s, 1H), 3.79 (s, 3zH), 1.84 (dd, J = 33.5, 16.6 Hz, 4H), 1.72 – 1.55 (m, 4H), 1.42 – 1.03 (m, 3H), 0.92 (p, J = 11.7 Hz, 2H). ^{13}C NMR (75 MHz, $CDCl_3$): δ/ppm = 170.3, 53.2, 51.2, 38.2, 33.4, 32.9, 32.6, 26.4, 26.0, 25.8. mp: 152–153 °C. $[\alpha]_D^{22}$ = +20 (c 1.00, $CHCl_3$). FT-IR: ν/cm^{-1} = 2929, 2855, 1730, 1483, 1227, 1209, 1156, 1046, 844, 753. MS (ESI) m/z calculated for $[C_{10}H_{21}NO_2]^+$ ($[M+H]^+$): 187.15, found: 187.21.

28, (4-Morpholine-1-carbonyl)-L-cyclohexyl alanine-methyl ester

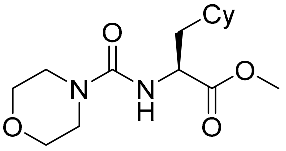

27 (0.55 g, 2.48 mmol, 1.0 eq) was dissolved in DCM and saturated $NaHCO_3$ solution (40 mL) and cooled to 0°C. Triphosgene (0.25 g, 0.83 mmol, 0.33 eq) was added and the mixture was stirred for 30 min. The mixture was extracted with DCM (2x 40 mL) and the combined organic layers were washed with saturated $NaHCO_3$ solution (2x 30 mL), and brine (2x 30 mL), then dried over anhydrous Na_2SO_4 and concentrated under reduced pressure, resulting in a crude product that was dissolved in THF (30 mL) and cooled to 0 °C. Morpholine (1.0 eq, 2.48 mmol, 0.22 g) was added and the mixture stirred for one hour. The solvent was removed under reduced pressure. Water (30 ml) to the crude residue, which was then extracted with EtOAc (3x 20 mL). The combined organic layers were washed with brine (2x 20 mL), dried over anhydrous Na_2SO_4, and concentrated under reduced pressure to yield a colorless oil (0.75 g, 2.46 mmol, 98%). ^{1}H NMR (300 MHz, $CDCl_3$): δ/ppm = 4.96 – 4.83 (m, 1H), 4.58 – 4.40 (m, 1H), 4.09 (q, J = 7.1 Hz, 1H), 3.70 (s, 3H), 3.69 – 3.61 (m, 4H), 3.41 – 3.28 (m, 4H), 2.01 (s, 1H), 1.76 (d, J = 12.8 Hz, 1H), 1.70 – 1.55 (m, 6H), 1.53 – 1.43 (m, 1H), 1.38 – 1.28 (m, 1H), 1.28 – 1.10 (m, 4H), 1.02 – 0.75 (m, 2H). ^{13}C NMR (75 MHz, $CDCl_3$): δ/ppm = 175.1, 157.4, 66.5, 52.3, 51.6, 44.1, 40.4, 34.2, 33.6, 32.7, 26.4, 26.2, 26.1, 21.1, 14.3. $[\alpha]_D^{22}$ = –9 (c 1.00, $CHCl_3$). FT-IR: ν/cm^{-1} = 3317, 3015, 2929, 1707, 1655, 1460, 1260, 1159, 910, 700. MS (ESI) m/z calculated for $[C_{15}H_{26}N_2O_4Na]^+$ ($[M+Na]^+$): 321.18, found: 321.18.

29, (4-Morpholin-1-carbonyl)-L-cyclohexyl alanine

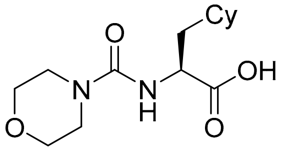

29 was prepared according to procedure B using **28** (1.0 eq, 0.7 g, 2.35 mmol), resulting in a colorless solid (0.65 g, 2.29 mmol, 97%).^{1}H NMR (300 MHz, $CDCl_3$): δ/ppm = 9.37 (s, 1H), 5.27 – 5.07 (m, 1H), 4.41 (s, 1H), 3.75 – 3.55 (m, 4H), 3.46 – 3.28 (m, 4H), 1.83 – 1.47 (m, 6H), 1.44 – 1.30 (m, 1H), 1.26 – 1.04 (m, 4H), 1.04 – 0.79 (m, 2H). ^{13}C NMR (75 MHz, $CDCl_3$): δ/ppm = 176.9, 158.1, 66.5, 51.9, 44.2, 39.6, 34.3, 33.6, 32.6, 26.6, 26.2, 26.1. 176.9, 158.1, 66.5, 51.9, 44.2, 39.6, 34.3, 33.6, 32.6, 26.5, 26.2, 26.1. mp: 96–97 °C. $[\alpha]_D^{22}$ = –22 (c 1.00,

$CHCl_3$). FT-IR: ν/cm^{-1} = 3320, 2978, 1707, 1655, 1460, 1368, 126, 1051, 910, 700. MS (ESI) *m/z* calculated for $[C_{14}H_{24}N_2O_4Na]^+$ ($[M+Na]^+$): 307.2, found: 307.2.

30, *N*-((*S*)-1-((*R*-2-(Benzyloxy)-1-cyanoethyl) amino)-3-cyclohexyl-1-oxopropan-2-yl) morpholine-4-carboxamideL-Cyclohexylalanine-methylester-hydrochloride

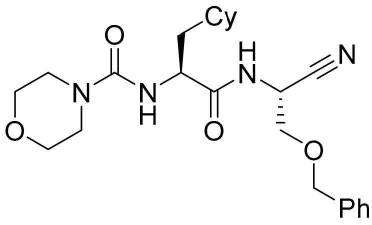

To a solution of NaCN (0.45 g, 9.11 mmol, 1.0 eq) and NH_4Cl (0.58 g, 10.87 mmol, 1.48 eq) in 35% aq. NH_4OH at 0°C, 2-(benzyloxy)acetaldehyde (1.0 eq, 7.35 mmol, 1.0 g) was added dropwise. The mixture was stirred for 48 h at room temperature after which a brown precipitate was formed. DCM (20 mL) and water (20 mL) were added and extracted with DCM (2x 30 mL). The organic extracts were washed with brine (1x 20 mL) and dried over anhydrous $NaSO_4$. The solvent was removed under reduced pressure yielding the intermediate aminonitrile as a pale-brown oil that was used in the next step without further purification. Coupling of the aminonitrile (31 mg, 0.18 mmol, 1.0 eq) with **29** (50 mg, 0.18 mmol, 1.0 eq) according to general procedure C followed by preparative HPLC purification yielded **30** as a colorless solid (20 mg, 0.05 mmol, 26%). ^{1}H NMR (300 MHz, $CDCl_3$): δ/ppm = 7.34 – 7.20 (m, 5H), 5.00 – 4.83 (m, 1H), 4.49 (d, *J* = 4.1 Hz, 2H), 4.39 (d, *J* = 7.2 Hz, 1H), 3.71 – 3.44 (m, 4H), 3.34 – 3.15 (m, 4H), 1.73 – 1.38 (m, 8H), 1.33 – 1.02 (m, 6H), 0.96 – 0.70 (m, 3H).^{13}C NMR (75 MHz, $CDCl_3$): δ/ppm = 173.8, 157.5, 136.9, 128.7, 128.3, 127.9, 117.3, 73.7, 68.9, 66.4, 52.3, 44.2, 40.9, 40.1, 34.3, 33.6, 32.9, 26.4, 26.3, 26.2. mp: 140–141 °C. $[\alpha]_D^{22}$ = –20 (*c* 1.00, $CHCl_3$). FT-IR: ν/cm^{-1} = 2920, 2850, 1666, 1619, 1521, 1447, 1247, 1114, 999, 698. MS (ESI) *m/z* calculated for $[C_{24}H_{34}N_4O_4Na]^+$ ($[M+Na]^+$): 465.3, found: 465.2. Purity: 99%.

32, *tert*-Butyl (*S*)-(3-(benzyloxy)-1-(methoxy(methyl)amino)-1-oxopropan-2-yl) carbamate

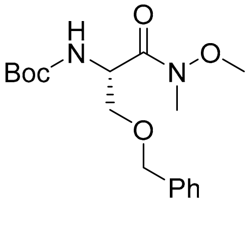

Boc-Ser(Bzl)-OH **31** (3.0 g, 10.2 mmol, 1.0 eq) was dissolved in DCM (20 mL) and cooled to –15 °C. *N*,*O*-dimethylhydroxyalmine · HCl (1.0 g, 10.26 mmol, 1.01 eq) and NMM (1.1 mL, 10.26 mmol, 1.01 eq) were added and after stirring for 5 min at this temperature EDC · HCl (1.97 g, 10.26 mmol, 1.01 eq) was added. The mixture was stirred overnight at room temperature and after consumption of the starting material, the solvent was removed under reduced pressure. Water (20 mL) and DCM (20 mL) were added, and the aqueous phase was extracted with DCM (3x 25 mL). The combined organic extracts were washed with brine (15 mL) and water (20 mL) dried over anhydrous $NaSO_4$ and concentrated under reduced

pressure. The crude product was purified by column chromatography (CH/EA 3:1), giving a colorless oil (3.4 g, 10.15 mmol, 98%). ^{1}H NMR (300 MHz, $CDCl_3$): δ/ppm = 7.37 – 7.11 (m, 5H), 5.48 – 5.25 (m, 1H), 4.84 (s, 1H), 4.58 – 4.40 (m, 2H), 3.66 (s, 3H), 3.65 – 3.57 (m, 2H), 3.16 (s, 3H), 1.39 (s, 9H). ^{13}C NMR (75 MHz, $CDCl_3$): δ/ppm = 170.8, 155.5, 137.9, 128.4, 127.7, 79.8, 73.2, 69.9, 61.5, 50.9, 32.4, 28.4. $[\alpha]_D^{22}$ = +5° (*c* 1.00, $CHCl_3$). FT-IR: ν/cm^{-1} = 2976, 1710, 1662, 1496, 1459, 1390, 1248, 1104, 1022, 986. MS (ESI) *m/z* calculated for $[C_{17}H_{26}N_2O_5Na]^+$ ($[M+Na]^+$): 361.2, found: 361.1.

33, *tert*-Butyl (*S*,*E*)-(1-(benzyloxy)-4-(phenylsulfonyl)but-3-en-2-yl) carbamate

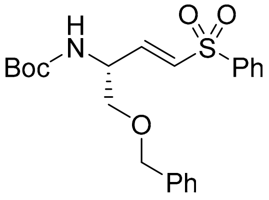

32 (0.5 g, 1.48 mmol, 1.0 eq) was dissolved in dry Et_2O (20 mL) at 0 °C. $LiAlH_4$ (72.9 mg, 1.92 mmol, 1.2 eq) was added and the mixture was stirred for 2 h at this temperature. The reaction was then quenched by adding hydrochloric acid (1 M, 15 mL). After phase separation, the organic phase was washed with hydrochloric acid (1 M, 15 mL) and brine (15 mL), dried over $NaSO_4$ and concentrated under reduced pressure yielding a colorless oil as crude product that was used in the next step without further characterization. To a solution of **3** (430 mg, 1.47 mmol, 1.0 eq) in dry THF at −78 °C, LiHMDS (1.0 M in THF, 1.62 mL) was added dropwise. After stirring for 30 min at this temperature the crude aldehyde (410 mg, 1.47 mmol, 1.0 eq) in THF (5 mL) was added. The mixture was stirred for 2.5 h at −78 °C. Saturated NH_4Cl-solution (15 mL) and EtOAc (5 mL) were added. After separating the phases, the aqueous phase was extracted with EtOAc (2x 15 mL) and the combined organic extracts were washed with water (15 mL) and brine (15 mL). The organic extract was dried over anhydrous Na_2SO_4 and concentrated under reduced pressure. The crude product was purified via column chromatography (CH/EA 3:1), yielding the desired product as a colorless oil (463 mg, 1.11 mmol, 75%). ^{1}H NMR (300 MHz, $CDCl_3$): δ/ppm = 7.90 – 7.81 (m, 2H), 7.65 – 7.46 (m, 3H), 7.39 – 7.21 (m, 4H), 6.98 (dd, *J* = 15.1, 4.8 Hz, 1H), 6.48 (dd, *J* = 15.1, 1.7 Hz, 1H), 5.01 (s, 1H), 4.64 – 4.42 (m, 2H), 3.58 (d, *J* = 4.1 Hz, 2H), 1.41 (s, 9H). ^{13}C NMR (75 MHz, $CDCl_3$): δ/ppm = 154.9, 144.5, 140.3, 137.3, 133.4, 131.5, 129.3, 128.5, 128.0, 127.7, 127.6, 80.3, 73.4, 70.7, 28.2. $[\alpha]_D^{22}$ = +10 (*c* 1.00, $CHCl_3$). FT-IR: ν/cm^{-1} = 2978, 1711, 1497, 1447, 1307, 1249, 1086, 1024, 751, 688. MS (ESI) *m/z* calculated for $[C_{22}H_{27}NO_5SNa]^+$ ($[M+Na]^+$): 440.2, found: 440.1.

34, *tert*-Butyl (*R*,*E*)-(1-(benzyloxy)-4-fluoro-4-(phenylsulfonyl) but-3-en-2-yl) carbamate

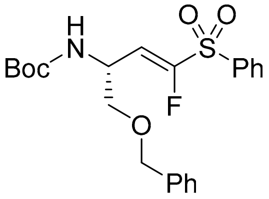

To a solution of **4** (170 mg, 0.55 mmol, 1.0 eq) in dry THF at −78 °C, KHMDS (1.0 M in THF, 0.55 mL) was added dropwise. After stirring for 30 min at this temperature the crude aldehyde (155 mg, 0.55 mmol, 1.0 Äq.) dissolved in THF (5 mL) was added. The mixture was stirred for 2.5 h at −78 °C. Saturated NH_4Cl-solution (15 mL) and EtOAc (5 mL) were added. Saturated NH_4Cl solution (15 mL) and EtOAc (5 mL) were added. After separating the phases, the aqueous phase was extracted with EtOAc (2x 15 mL) and the combined organic extracts were washed with water (15 mL) and brine (15 mL). The combined organic layers were dried over anhydrous Na_2SO_4 and concentrated in under reduced pressure. The crude product was purified via column chromatography (CH/EA 5:1), yielding the desired product as a colorless oil (105 mg, 0.24 mmol, 44%). ^{1}H NMR (300 MHz, $CDCl_3$): *δ*/ppm = 8.03 (s, 2H), 7.62 – 7.52 (m, 1H), 7.49 – 7.39 (m, 2H), 7.32 – 7.14 (m, 5H), 5.84 (dd, *J* = 21.2, 9.7 Hz, 1H), 5.48 (s, 1H), 5.15 (d, *J* = 8.2 Hz, 1H), 4.45 (s, 2H), 3.72 – 3.51 (m, 2H), 1.36 (s, 9H). ^{13}C NMR (75 MHz, $CDCl_3$): *δ*/ppm = 155.0, 137.5, 134.6, 129.4, 128.5, 127.9, 119.9, 119.8, 73.4, 72.8, 46.3, 28.4. $[\alpha]_D^{22}$ = –64 (*c* 1.00, $CHCl_3$). FT-IR: *ν*/cm^{-1} = 3390, 2978, 2929, 1711, 1497, 1366, 1335, 1166, 1081, 735. MS (ESI) *m/z* calculated for $[C_{22}H_{26}FNO_5SNa]^+$ ($[M+Na]^+$): 458.1, found: 458.1.

35, *N*-((*S*)-1-(((*S*,*E*)-1-(benzyloxy)-4-(phenylsulfonyl) but-3-en-2-yl) amino)-3-cyclohexyl-1-oxopropan-2-yl) morpholin-4-carboxamide

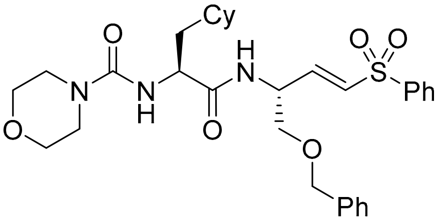

Deprotection of **33** (88 mg, 0.21 mmol, 1.0 eq) was performed following general procedure A. The deprotected amino acid was then coupled with **29** (60 mg, 0.21 mmol, 1.0 eq) following general procedure C. After purification via column chromatography ($CHCl_3$/MeOH 75:1) the desired product was obtained as a colorless solid (74 mg, 0.13 mmol, 62%). ^{1}H NMR (300 MHz, $CDCl_3$): *δ*/ppm = 7.86 – 7.74 (m, 2H), 7.64 – 7.52 (m, 1H), 7.51 – 7.41 (m, 2H), 7.36 – 7.15 (m, 6H), 6.96 (dd, *J* = 15.1, 4.6 Hz, 1H), 6.48 (dd, *J* = 15.1, 1.8 Hz, 1H), 5.03 (d, *J* = 7.7 Hz, 1H), 4.89 – 4.74 (m, 1H), 4.53 – 4.21 (m, 3H), 3.65 – 3.40 (m, 5H), 3.35 – 3.16 (m, 4H), 1.72 – 1.36 (m, 8H), 1.31 – 0.73 (m, 6H). ^{13}C NMR (75 MHz, $CDCl_3$): *δ*/ppm = 173.4, 157.4, 144.0, 140.3, 137.4, 133.5, 131.7, 129.3, 128.6, 127.9, 127.7, 127.7, 73.4, 70.6, 66.4, 52.6, 49.4, 44.1, 40.1, 34.3, 33.5, 32.9, 26.4, 26.2, 26.1. mp: 74–75 °C. $[\alpha]_D^{22}$ = +5 (*c* 1.00, $CHCl_3$). FT-IR: *ν*/cm^{-1} = 3756, 2921, 2851, 1659, 1537, 1446, 1261, 1147, 1117, 999. MS (ESI) *m/z* calculated for $[C_{31}H_{42}N_3O_6S]^+$ ($[M+H]^+$): 584.1, found: 584.2. Purity: 98%.

36, *N*-((*S*)-1-(((*S*,*E*)-1-(Benzyloxy)-4-fluoro-4-(phenylsulfonyl) but-3-en-2-yl) amino)-3-cyclohexyl-1-oxopropan-2-yl) morpholine-4-carboxamide

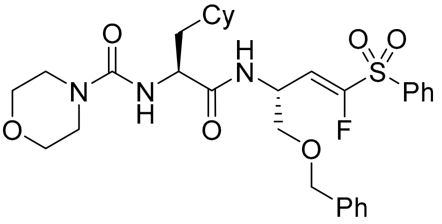

Deprotection of **38** (90 mg, 0.21 mmol, 1.0 eq) was performed following the general procedure A. The deprotected amino acid was then coupled with **29** (60 mg, 0.21 mmol, 1.0 eq) following general procedure C. After purification via preparative HPLC using a **36** was obtained as an amorphous glass (80 mg, 0.13 mmol, 63%). ^{1}H NMR (300 MHz, $CDCl_3$): δ/ppm = 8.08 – 8.02 (m, 2H), 7.71 – 7.62 (m, 1H), 7.60 – 7.46 (m, 2H), 7.38 – 7.21 (m, 5H), 5.97 – 5.82 (m, 1H), 5.81 – 5.66 (m, 1H), 5.28 (d, *J* = 7.9 Hz, 1H), 4.66 – 4.44 (m, 2H), 4.37 (td, *J* = 8.3, 5.9 Hz, 1H), 3.79 – 3.54 (m, 5H), 3.39 – 3.23 (m, 4H), 1.74 – 1.40 (m, 6H), 1.34 – 0.74 (m, 9H). ^{13}C NMR (75 MHz, $CDCl_3$): δ/ppm = 173.62, 170.03, 157.56, 154.85, 150.95, 137.51 (d, *J* = 6.3 Hz), 134.7, 129.5, 128.9, 128.6, 127.9, 127.8, 119.2, 119.0, 73.4, 72.3, 66.5, 52.4, 45.2, 44.1, 39.9, 34.3, 33.6, 32.8, 29.7, 26.4, 26.2, 23.2. mp: 78–79 °C. $[\alpha]_D^{22}$ = –22 (*c* 1.00, $CHCl_3$). FT-IR: ν/cm^{-1} = 3274, 3064, 2922, 2852, 1661, 1541, 1448, 1334, 1116, 735. MS (ESI) *m/z* calculated for $[C_{31}H_{41}FN_3O_6S]^+$ ($[M+H]^+$): 602.3, found: 602.2. Purity: 97%.

37, Methyl *O*-benzyl-*N*- (tert-butoxy carbonyl)-L-serinate

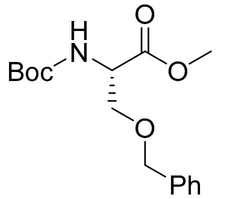

To a solution of Boc-Ser(Bzl)-OH **31** (3.0 g, 10.16 mmol, 1.0 eq) in DMF (40 mL) at 0°C, K_2CO_3 (2.8 g, 20.3 mmol, 2.0 eq) and MeI (1.26 mL, 20.3 mmol, 2.0 eq) were added. The mixture was stirred overnight at room temperature and subsequently diluted with saturated NH_4Cl solution (30 mL). The aqueous phase was extracted with EtOAc (3x 25 mL). The combined organic layers were dried over anhydrous Na_2SO_4 and concentrated under reduced pressure to yield **37** as a pale-orange oil (3.05 g, 9.85 mmol, 97%). ^{1}H NMR (300 MHz, $CDCl_3$): δ/ppm = 7.38 – 7.09 (m, 5H), 5.38 (d, *J* = 8.8 Hz, 1H), 4.59 – 4.35 (m, 3H), 3.84 (dd, *J* = 9.4, 3.3 Hz, 1H), 3.72 (s, 3H), 3.66 (dd, *J* = 9.4, 3.4 Hz, 1H), 1.43 (s, 9H). ^{13}C NMR (75 MHz, $CDCl_3$): δ/ppm = 171.2, 155.6, 137.7, 128.5, 127.9, 127.7, 80.1, 73.3, 70.1, 54.1, 52.5, 28.4. $[\alpha]_D^{22}$ = +33 (*c* 1.00, $CHCl_3$). FT-IR: ν/cm^{-1} = 2978, 2360, 1750, 1714, 1497, 1365, 1296, 1208, 1062, 739. MS (ESI) *m/z* calculated for $[C_{11}H_{16}NO_3]^+$ ($[M-Boc+H]^+$): 210.1, found: 210.0.

38, *tert*-Butyl (*S*)-(1-(benzyloxy)-4-(dimethoxy phosphoryl)-3-oxobutan-2-yl) carbamate

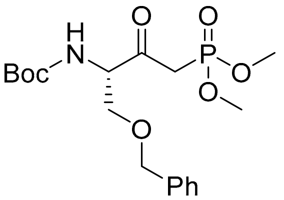

To a solution of dimethyl methylphosphonate (2.2 g, 18.4 mmol, 4.0 eq) in dry THF (12 mL) at −78 °C under an argon-atmosphere, *n*-BuLi (2.5 M in hexanes, 7.36 mL) was added dropwise. After stirring for one hour, **37** (1.5 g, 4.6 mmol, 1.0 eq) was added and the mixture stirred for an additional 2.5 h at this temperature. The reaction was then stopped by adding water (30 mL) and EtOAc (30 mL). After extraction with EtOAc (3x 20 mL) the organic extracts were dried over anhydrous Na_2SO_4 and the mixture concentrated under reduced pressure to give a crude product that was purified via column chromatography (CH/EA 1:3) yielding a pale-yellow oil (1.46 g, 3.64 mmol, 79%). ^{1}H NMR (300 MHz, $CDCl_3$): δ/ppm = 7.36 – 7.20 (m, 5H), 5.58 (d, J = 7.7 Hz, 1H), 4.55 – 4.45 (m, 3H), 3.93 – 3.85 (m, 1H), 3.79 – 3.61 (m, 7H), 3.31 (dd, J = 22.3, 14.5 Hz, 1H), 3.10 (dd, J = 21.9, 14.5 Hz, 1H), 1.41 (s, 9H). ^{13}C NMR (75 MHz, $CDCl_3$): δ/ppm = 199.7, 155.4, 137.4, 128.5, 127.9, 127.8, 80.2, 73.4, 69.3, 60.4, 53.2, 39.2, 37.4, 28.3. $[\alpha]_D^{22}$ = +3 (*c* 1.00, $CHCl_3$). FT-IR: ν/cm^{-1} = 2976, 1708, 1496, 1454, 1366, 1251, 1166, 1068, 866, 809. MS (ESI) *m/z* calculated for $[C_{13}H_{21}NO_5P]^+$ ($[M-Boc+H]^+$): 302.1, found: 302.1.

39, Dimethyl ((*S*)-4-(benzyloxy)-3-((*S*)-3-cyclohexyl-2-(morpholine-4-carboxamido) propanamido)-2-oxobutyl) phosphonate

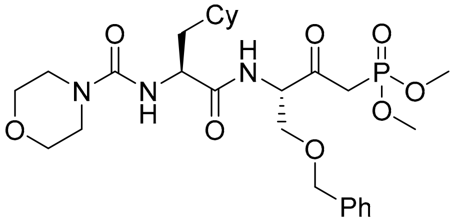

Deprotection of **38** (0.83 g, 2.07 mmol, 1.0 eq) was performed following the general procedure A. The deprotected amino acid was then coupled with **29** (0.59 g, 2.07 mmol, 1.0 eq) following general procedure C. After Purification via preparative HPLC **39** was obtained as a colorless oil (0.68 g, 1.2 mmol, 58%). ^{1}H NMR (300 MHz, $CDCl_3$): δ/ppm = 7.66 (d, J = 8.3 Hz, 1H), 7.34 – 7.20 (m, 5H), 4.72 (dt, J = 8.0, 3.8 Hz, 1H), 4.54 – 4.38 (m, 3H), 3.97 (dd, J = 9.7, 3.8 Hz, 1H), 3.78 – 3.53 (m, 11H), 3.49 – 3.26 (m, 1H), 3.04 (dd, J = 22.5, 14.0 Hz, 4H), 1.84 – 1.56 (m, 6H), 1.55 – 1.41 (m, 1H), 1.40 – 1.26 (m, 1H), 1.24 – 0.78 (m, 6H). ^{13}C NMR (75 MHz, $CDCl_3$): δ/ppm = 199.7, 199.6, 173.8, 157.6, 137.5, 128.5, 127.9, 127.7, 73.5, 68.7, 66.5, 59.1, 59.1, 53.4, 53.3, 53.2, 52.5, 44.2, 40.1, 39.7, 37.9, 34.3, 33.8, 32.7, 26.5, 26.3, 26.1. $[\alpha]_D^{22}$ = –21 (*c* 1.00, $CHCl_3$). FT-IR: ν/cm^{-1} = 3278, 2920, 2850, 2360, 1627, 1533, 1449, 1249, 1115, 999. MS (ESI) *m/z* calculated for $[C_{27}H_{43}N_3O_8P]^+$ ($[M+H]^+$): 568.3, found: 568.2.

Ethyl (*S*,*E*)-6-(benzyloxy)-5-((*S*)-3-cyclohexyl-2-(morpholine-4-carboxamido) propanamido)-4-oxohex-2-enoate

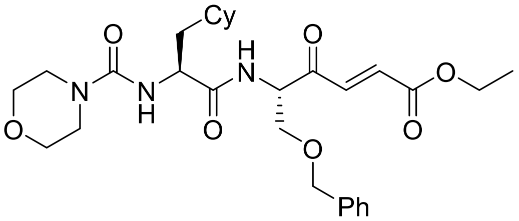

In a schlenk tube containing LiCl (8 mg, 0.19 mmol, 1.2 eq), a solution of **39** (90 mg, 0.16 mmol, 1.0 eq) in MeCN was added. At 0 °C, over P_2O_{10}, freshly distilled ethyl glyoxylate (33 µL, 0.32 mmol, 2.0 eq) and DIPEA (36 µL, 0.21 mmol, 1.3 eq) were added and the mixture was stirred for 2 h. After adding a saturated NH_4Cl solution (30 mL) and extracting with EtOAc (3x 20 mL), the organic extracts were washed with brine (20 mL), dried over anhydrous Na_2SO_4 and concentrated under reduced pressure to yield a crude which was purified via preparative HPLC. **40** could be obtained as a colorless solid (34 mg, 0.06 mmol, 10%). ^{1}H NMR (300 MHz, $CDCl_3$): *δ*/ppm = 7.48 – 7.14 (m, 5H), 6.96 – 6.69 (m, 1H), 4.56 – 3.88 (m, 9H), 3.71 – 3.57 (m, 4H), 3.38 – 3.25 (m, 4H), 1.80 – 1.55 (m, 6H), 1.36 – 1.23 (m, 3H), 1.20 – 1.07 (m, 6H), 1.01 – 0.79 (m, 3H). ^{13}C NMR (75 MHz, $CDCl_3$): *δ*/ppm = 192.6, 192.2, 165.4, 165.1, 157.2, 137.0, 133.8, 133.3, 131.7, 128.6, 128.0, 73.9, 70.7, 66.5, 62.2, 61.6, 52.5, 44.2, 34.2, 33.7 32.9, 32.7, 14.3, 14.0. mp: 62–63 °C. $[\alpha]_D^{22}$ = –23 (*c* 1.00, $CHCl_3$). FT-IR: *ν*/cm^{-1} = 2923, 1735, 1632, 1526, 1449, 1370, 1250, 1187, 1115, 752 cm^{-1}. MS (ESI) *m/z* calculated for $[C_{29}H_{42}N_3O_7]^+$ ($[M+H]^+$): 544.3, found: 544.2. Purity: 99%.

41, *tert*-Butyl (*S*)-(1-(benzo[*d*]thiazol-2-yl)-3-(benzyloxy)-1-oxopropan-2-yl) carbamate

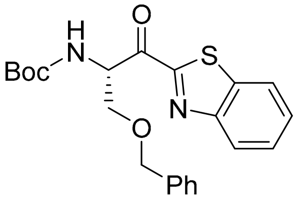

To a solution of benzothiazole (0.87 mL, 8.08 mmol, 10.0 eq) in THF (25 mL) at −78 °C under argon, *n*-BuLi (2.5 M in hexanes, 3.23 mL, 8.08 mmol, 10.0 eq) was added dropwise. The mixture was stirred for 30 min at this temperature and subsequently **37** (250 mg, 0.81 mmol, 1.0 eq) was added and stirred for an additional three hours. After consumption of the starting material, the mixture was diluted with a saturated NH_4Cl solution (30 mL). The aqueous phase was extracted with EtOAc (3x 25 mL). The organic extracts were dried over anhydrous Na_2SO_4 and concentrated to give a crude product that was purified via column chromatography (CH/EA 10:1) which gave access to **41** as a pale-yellow oil (215 mg, 0.52 mmol, 64%). ^{1}H NMR (300 MHz, $CDCl_3$): *δ*/ppm = 8.15 – 8.04 (m, 1H), 8.03 – 7.92 (m, 1H), 7.62 – 7.49 (m, 2H), 7.20 – 7.05 (m, 5H), 5.78 – 5.60 (m, 2H), 4.58 – 4.35 (m, 2H), 4.35 – 4.28 (m, 1H), 4.01 – 3.84 (m, 1H), 1.47 (s, 9H). ^{13}C NMR (75 MHz, $CDCl_3$): *δ*/ppm = 191.4, 163.9, 155.5, 153.5, 137.5, 137.3, 128.3, 128.0, 127.7, 127.6, 127.1, 125.8, 122.5, 80.1, 73.3, 70.5, 57.9, 28.5. $[\alpha]_D^{22}$ = +1 (*c* 1.00, $CHCl_3$). FT-IR: *ν*/cm^{-1} = 2978, 2359, 1698, 1485, 1454, 1367, 1216, 1165, 1102, 744. MS (ESI) *m/z* calculated for $[C_{22}H_{24}N_2O_4SNa]^+$ ($[M+H]^+$): 435.1, found: 435.1.

42, *N*-((*S*)-1-(((*S*)-1-(benzo[*d*]thiazol-2-yl)-3-(benzyloxy)-1-oxopropan-2-yl) amino)-3-cyclohexyl-1-oxopropan-2-yl) morpholine-4-carboxamide

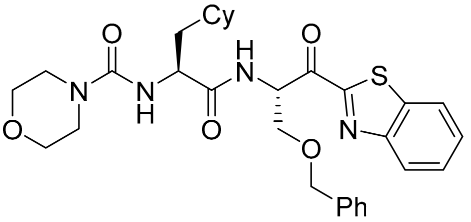

Deprotection of **41** (56 mg, 0.14 mmol, 1.0 eq) was performed following general procedure A. The deprotected amino acid was then coupled with **29** (40 mg, 0.14 mmol, 1.0 eq) following general procedure C. After purification via preparative HPLC **42** could be obtained as a colorless solid (33 mg, 0.06 mmol, 44%). ^{1}H NMR (300 MHz, $CDCl_3$): δ/ppm = 8.15 – 8.05 (m, 1H), 8.03 – 7.92 (m, 1H), 7.63 – 7.47 (m, 2H), 7.10 (d, J = 6.9 Hz, 5H), 5.95 – 5.78 (m, 1H), 4.64 – 4.44 (m, 2H), 4.41 – 4.25 (m, 2H), 4.02 – 3.86 (m, 1H), 3.65 (q, J = 4.7 Hz, 4H), 3.45 – 3.28 (m, 4H), 1.89 – 1.34 (m, 9H), 1.30 – 0.80 (m, 5H). ^{13}C NMR (75 MHz, $CDCl_3$): δ/ppm = 190.4, 173.5, 163.7, 157.6, 153.5, 137.4, 137.3, 137.2, 128.3, 128.1, 127.7, 127.6, 127.6, 127.2, 125.8, 122.4, 73.2, 70.0, 66.6, 56.7, 52.5, 52.3, 44.3, 40.8, 40.2, 34.4, 33.8, 32.9, 26.5, 26.3, 26.2. mp: 95–96 °C. $[\alpha]_D^{22}$ = –12 (c 1.00, $CHCl_3$). FT-IR: ν/cm^{-1} = 3275, 2920, 2850, 1648, 1541, 1485, 1249, 1116, 998, 761. MS (ESI) *m/z* calculated for $[C_{31}H_{39}N_4O_5S]^+$ ($[M+H]^+$): 579.3, found: 580.1. Purity: 97%

43, Methyl *O*-benzyl-*N*-((*S*)-3-cyclohexyl-2-(morpholine-4-carboxamido) propanoyl)-L-serinate

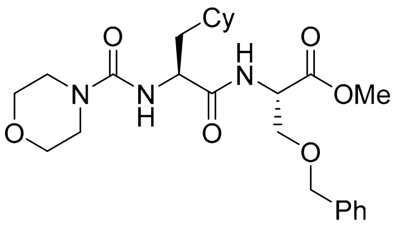

Deprotection of **37** (217 mg, 0.7 mmol, 1.0 eq) was performed following general procedure A. The deprotected amino acid was then coupled with **29** (200 mg, 0.7 mmol, 1.0 eq) following general procedure C. After purification via column chromatography (CH/EA 1:3), **43** could be obtained as a colorless oil (253 mg, 0.53 mmol, 76%). ^{1}H NMR (300 MHz, $CDCl_3$): δ/ppm = 7.36 – 7.19 (m, 5H), 4.69 (dt, J = 8.2, 3.4 Hz, 1H), 4.58 – 4.37 (m, 3H), 3.86 (dd, J = 9.6, 3.4 Hz, 1H), 3.71 (s, 3H), 3.68 – 3.52 (m, 5H), 3.41 – 3.24 (m, 4H), 1.86 – 1.58 (m, 6H), 1.57 – 1.31 (m, 3H), 1.29 – 0.80 (m, 6H). ^{13}C NMR (75 MHz, $CDCl_3$): δ/ppm = 173.7, 170.5, 157.5, 137.6, 128.5, 127.9, 127.7, 73.4, 69.6, 66.5, 52.8, 52.6, 52.4, 44.2, 40.6, 34.2, 33.7, 32.9, 26.5, 26.3, 26.2. $[\alpha]_D^{22}$ = +13 (c 1.00, $CHCl_3$). FT-IR: ν/cm^{-1} = 3276, 2920, 2850, 2360, 1748, 1662, 1538, 1449, 1261, 1116. MS (ESI) *m/z* calculated for $[C_{25}H_{37}N_3O_6Na]^+$ ($[M+Na]^+$): 498.3, found: 498.2.

44, *O*-benzyl-*N*-((*S*)-3-cyclohexyl-2-(morpholine-4-carboxamido) propanoyl)-L-serine

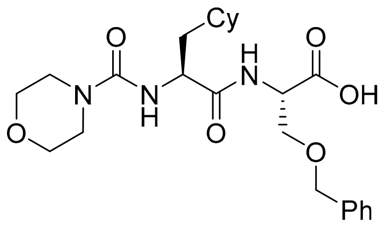

The methyl ester **43** (252 mg, 0.53 mmol, 1.0 eq) was cleaved according to the general procedure B. **44** was obtained as a colorless solid (250 mg, 0.52 mmol, 98%). ^{1}H NMR (300 MHz, $CDCl_3$): δ/ppm = 7.43 – 7.18 (m, 5H), 4.75 – 4.24 (m, 2H), 3.98 – 3.64 (m, 2H), 3.63 – 3.44 (m, 4H), 3.44 – 3.19 (m, 4H), 2.60 (s, 2H), 2.46 (s, 1H), 1.77 – 1.48 (m, 7H), 1.44 – 0.68 (m, 8H). ^{13}C NMR (75 MHz, $CDCl_3$): δ/ppm = 174.4, 172.9, 157.7, 153.0, 137.7, 128.5, 127.9, 127.8, 127.7, 125.1, 73.4, 69.6, 66.5, 53.5, 52.9, 44.2, 39.9, 34.2, 33.7, 32.7, 26.5, 26.3, 26.2, 21.9, 19.5. mp: 75–76 °C. $[\alpha]_D^{22}$ = +19 (*c* 1.00, $CHCl_3$). FT-IR: ν/cm^{-1} = 3304, 2922, 2852, 2359, 1725, 1622, 1532, 1450, 1260, 1116. MS (ESI) *m/z* calculated for $[C_{24}H_{36}N_3O_6]^+$ ($[M+H]^+$): 462.3 found: 462.4.

45, *N*-((2*S*)-1-(((2*S*)-3-(benzyloxy)-1-oxo-1-((2-oxo-1-phenylazetidin-3-yl) amino) propan-2-yl) amino)-3-cyclohexyl-1-oxopropan-2-yl) morpholine-4-carboxamide

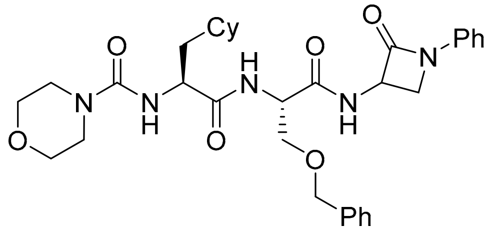

Coupling of **44** (185 mg, 0.4 mmol, 1.0 eq) with **9** (80 mg, 0.4 mmol, 1.0 eq) was performed following general procedure C. After purification via preparative HPLC **45** could be obtained as a colorless solid (169 mg, 0.28 mmol, 70%). ^{1}H NMR (300 MHz, $CDCl_3$): δ/ppm = 8.15 (d, *J* = 7.3 Hz, 1H), 7.35 – 7.12 (m, 10H), 7.08 – 6.95 (m, 2H), 5.10 (s, 2H), 5.00 – 4.80 (m, 1H), 4.61 – 4.51 (m, 1H), 4.43 (q, *J* = 11.8 Hz, 2H), 4.14 – 4.03 (m, 1H), 3.94 (dd, *J* = 9.6, 4.1 Hz, 1H), 3.81 (t, *J* = 5.6 Hz, 1H), 3.63 – 3.49 (m, 2H), 3.48 – 3.31 (m, 4H), 3.25 – 2.99 (m, 4H), 1.70 – 1.37 (m, 6H), 1.35 – 0.68 (m, 9H).^{13}C NMR (75 MHz, $CDCl_3$): δ/ppm = 173.5, 170.6, 163.7, 157.9, 138.0, 137.8, 129.3, 128.6, 128.0, 127.9, 124.4, 166.7, 73.5, 69.4, 66.3, 55.8, 53.8, 53.2, 47.1, 44.1, 39.3, 34.5, 33.8, 32.5, 26.4, 26.2, 26.1. mp: 91–92 °C. $[\alpha]_D^{22}$ = +11 (*c* 1.00, $CHCl_3$). FT-IR: ν/cm^{-1} = 3271, 2922, 2850, 2340, 1754, 1626, 1502, 1386, 115, 866. MS (ESI) *m/z* calculated for $[C_{33}H_{43}N_5O_6Na]^+$ ($[M+Na]^+$): 628.3, found: 628.4. Purity: 99%.

46, *N*-((*S*)-1-(((*R*)-1-(benzyloxy)-3-hydroxypropan-2-yl) amino)-3-cyclohexyl-1-oxopropan-2-yl) morpholine-4-carboxamide

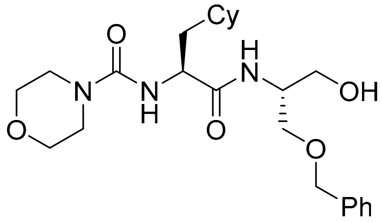

To a solution of **43** (132 mg, 0.28 mmol, 1.0 eq) in THF (15 mL) at 0 °C, $NaBH_4$ (42 mg, 1.11 mmol, 4.0 eq) and MeOH (1.2 mL) were added. The mixture was stirred for 16 h at room temperature and upon completion, diluted with saturated NH_4Cl solution (30 mL). After extraction with EtOAc (3x 25 mL) the combined organic layers were washed with saturated NH_4Cl solution (20 mL) and brine (20 mL), dried over anhydrous Na_2SO_4 and concentrated under reduced pressure to yield **46** as a colorless solid (114 mg, 0.25 mmol, 91%). ^{1}H NMR (300 MHz, $CDCl_3$): δ/ppm = 7.34 – 7.18 (m, 5H), 6.98 (d, *J* = 7.5 Hz, 1H), 5.26 (d, *J* = 7.7 Hz, 1H), 4.55 – 4.40 (m, 2H), 4.33 (td, *J* = 8.4, 6.3 Hz, 1H), 4.08 – 3.91 (m, 2H), 3.67 – 3.41 (m, 7H), 3.33 – 3.07 (m, 4H), 1.78 – 1.43 (m, 6H), 1.32 – 0.71 (m, 9H). ^{13}C NMR (75 MHz, $CDCl_3$): δ/ppm = 174.1, 171.2, 157.5, 137.8, 135.9, 128.5, 127.9, 127.7, 125.6, 73.5, 69.6, 66.5, 62.9, 60.5, 52.7, 51.2, 44.1, 40.4, 34.3, 33.6, 32.9, 26.3, 21.1, 14.3. mp: 89–90 °C. $[\alpha]_D^{22}$ = +14 (*c* 1.00, $CHCl_3$). FT-IR: ν/cm^{-1} = 33280, 2916, 2850, 2265, 2339, 1615, 1540, 1447, 1250, 1111. MS (ESI) *m/z* calculated for $[C_{24}H_{37}N_3O_5Na]^+$ ($[M+Na]^+$): 470.3, found: 470.3.

47, *N*-((*S*)-1-(((*S*)-1-(benzyloxy)-3-oxopropan-2-yl) amino)-3-cyclohexyl-1-oxopropan-2-yl) morpholine-4-carboxamide

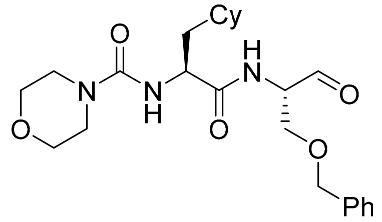

To a solution of **46** (45 mg, 0.1 mmol, 1.0 eq) in DCM (10 mL) Dess-Martin-Periodinane (51 mg, 0.12 mmol, 1.2 eq) was added and the mixture was stirred at room temperature for 12 h. After completion, indicated by TLC, the mixture was filtrated, washed a saturated $Na_2S_2O_3$ solution (15 mL), a saturated $NaHCO_3$ solution (15 mL) and brine (20 mL). The mixture was dried over anhydrous Na_2SO_4, concentrated under reduced pressure and subsequently purified via column chromatography ($CHCl_3$/MeOH 30:1), to yield **47** as a colorless solid (31 mg, 0.07 mmol, 70%). ^{1}H NMR (300 MHz, $CDCl_3$): δ/ppm = 9.56 (s, 1H), 7.43 – 7.17 (m, 5H), 5.15 – 4.96 (m, 1H), 4.76 – 4.34 (m, 3H), 4.25 (s, 1H), 3.99 (dd, *J* = 9.7, 3.6 Hz, 1H), 3.75 – 3.53 (m, 4H), 3.48 – 3.20 (m, 4H), 1.84 – 1.47 (m, 6H), 1.40 – 0.77 (m, 8H). ^{13}C NMR (75 MHz, $CDCl_3$): δ/ppm = 198.3, 174.1, 157.5, 137.3, 128.6 127.8, 127.7, 73.7, 67.5, 66.5, 59.0,52.4, 44.2, 40.6, 34.3, 33.7, 32.9, 26.5, 26.3. mp: 85–86 °C. $[\alpha]_D^{22}$ = +20 (*c* 1.00, MeOH).

FT-IR: ν/cm^{-1} = 3291, 2921, 2851, 2363, 2341, 1624, 1541, 1448, 1261, 1117. MS (ESI) *m/z* calculated for $[C_{24}H_{25}N_3O_5Na]^+$ ($[M+Na]^+$): 468.3, found: 468.3.

48, *N*-((2*S*)-1-(((2*S*)-1-(benzyloxy)-3-hydroxy-4-nitrobutan-2-yl) amino)-3-cyclohexyl-1-oxopropan-2-yl) morpholine-4-carboxamide

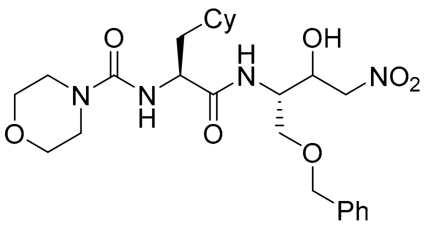

To a suspension of NaH (60% in paraffin, 23 mg, 0.56 mmol, 1.25 eq), and $MeNO_2$ (24 µL, 0.45 mmol, 1.0 eq) in THF (20 mL) at 0 °C, **48** (200 mg, 0.45 mmol, 1.0 eq) was added and the mixture was stirred for one hour. After completion, indicated by TLC, the mixture was diluted with a saturated NH_4Cl solution (20 mL). After separation of the aqueous phase, the organic phase was washed with brine (15 mL), dried over anhydrous Na_2SO_4 and concentrated to yield a crude yellowish-oil **48** (132 mg), which was used in the next step without further purification.

49, *N*-((*S*)-1-(((*R*,*E*)-1-(benzyloxy)-4-nitrobut-3-en-2-yl) amino)-3-cyclohexyl-1-oxopropan-2-yl) morpholine-4-carboxamide

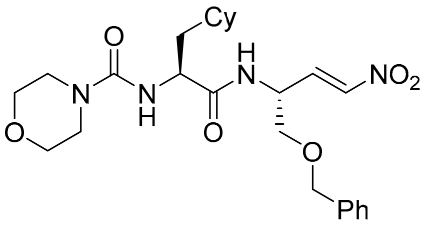

To a solution of **48** (72 mg, 0.14 mmol, 1.0 eq) and Et_3N (59 µL, 0.43 mmol, 3.0 eq) in DCM (10 mL) at 0 °C, MsCl (16 µL, 0.21 mmol, 1.5 eq) was added and the mixture was stirred for one hour at room temperature. The mixture was diluted and washed with a saturated $NaHCO_3$ solution (15 mL) and extracted with EtOAc (3x 25 mL). The mixture was dried over anhydrous Na_2SO_4, concentrated under reduced pressure and subsequently purified via preparative HPLC yielding **49** as a colorless solid (30 mg, 0.06 mmol, 44%). ^{1}H NMR (300 MHz, $CDCl_3$): δ/ppm = 7.43 – 7.27 (m, 5H), 7.25 – 7.15 (m, 1H), 7.12 – 6.99 (m, 1H), 4.95 – 4.79 (m, 1H), 4.63 – 4.45 (m, 2H), 4.44 – 4.29 (m, 1H), 3.73 – 3.52 (m, 4H), 3.44 – 3.19 (m, 4H), 1.91 – 1.45 (m, 8H), 1.40 – 0.69 (m, 9H). ^{13}C NMR (75 MHz, $CDCl_3$): δ/ppm = 173.5, 157.6, 141.2, 139.2, 137.2, 128.8, 128.3, 127.9, 73.7, 70.3, 66.5, 52.6, 47.8, 44.2, 39.9, 34.5, 33.7, 32.9, 26.5, 26.3, 26.2. mp: 71–72 °C. $[\alpha]_D^{22}$ = –4 (*c* 1.00, $CHCl_3$). FT-IR: ν/cm^{-1} = 3286, 2923, 2852, 2360, 2341, 1653, 1624, 1449, 1351, 117. MS (ESI) *m/z* calculated for $[C_{25}H_{36}N_4O_6Na]^+$ ($[M+Na]^+$): 511.3, found: 511.2. Purity: 99%.

Synthesis of proteasome targeting compounds

51, *tert*-Butyl (*S*)-(1-(methoxy(methyl)amino)-4-methyl-1-oxopentan-2-yl)carbamate

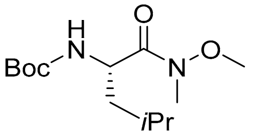

To a 0 °C cold solution of Boc-Leu-OH **50** (2.04 g, 8.81 mmol, 1 eq) in DCM (80 mL) were added HOBt · H_2O (1.35 g, 8.81 mmol, 1 eq) and 2,4,6-collidine (2.34 mL, 17.62 mmol, 2 eq). After stirring at 0 °C for 30 min, TBTU (2.83 g, 8.81 mmol, 1 eq) was added. The solution was stirred for further 30 min at 0 °C and *N*,*O*-dimethylhydroxylamine · HCl (0.89 g, 8.81 mmol, 1 eq) was added. After stirring at room temperature overnight, the solvent was removed under reduced pressure and the residue was taken up in EtOAc (100 mL). The mixture was washed with saturated $NaHCO_3$ solution (3x 80 mL) and hydrochloric acid (1 M, 3x 80 mL) and filtered over a small silica column. The filtrate was concentrated under reduced pressure to yield the desired product as a colorless oil (2.24 g, 8.18 mmol, 93%). ^1H NMR (300 MHz, $CDCl_3$): δ/ppm = 5.05 (d, J = 9.5 Hz, 1H), 4.70 (s, 1H), 3.77 (s, 3H), 3.18 (s, 3H), 1.78–1.62 (m, 1H), 1.47–1.38 (m, 11H), 0.99–0.88 (m, 6H). ^{13}C NMR (75.5 MHz, $CDCl_3$): δ/ppm = 174.0, 155.8, 79.6, 61.7, 49.1, 42.2, 32.3, 28.5, 24.8, 23.5, 21.7. $[\alpha]_D^{22}$ = –9 (*c* 1.00, $CHCl_3$). FT-IR: ν/cm^{-1} = 3324, 2958, 2936, 2870, 1709, 1658, 1501, 1389, 1366, 1250, 1165, 1045, 1016, 989, 876. MS (ESI) *m*/*z* calculated for $[C_{13}H_{26}N_2O_4+Na]^+$ ($[M+Na]^+$): 297.2, found: 297.1.

52, *tert*-Butyl (4-methyl-1-oxopentan-2-yl)carbamate

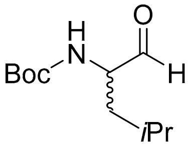

To a solution of **51** (1.51 g, 5.50 mmol, 1 eq) in dry THF (20 mL) was added $LiAlH_4$ (271 mg, 7.15 mmol, 1.3 eq) portion wise at 0 °C under argon atmosphere. After stirring at 0 °C for 30 min, diethyl ether (50 mL) and $KHSO_4$ solution (0.33 M, 80 mL,) were added. The suspension was filtered, and the filtrate was extracted with diethyl ether (2x 50 mL). The combined organic extracts were washed with hydrochloric acid (1 M, 2x 40 mL) and saturated $NaHCO_3$ solution (2x 40 mL) and dried over anhydrous Na_2SO_4. After removing the solvent under reduced pressure, the desired product was obtained as a colorless oil (345 mg, 1.60 mmol, 29%). ^1H NMR (300 MHz, $CDCl_3$): δ/ppm = 9.57 (s, 1H), 4.95 (d, J = 7.3 Hz, 1H), 4.23 (s, 1H), 1.81–1.70 (m, 1H), 1.67–1.58 (m, 1H), 1.44 (s, 9H), 1.39–1.32 (m, 1H), 0.98–0.94 (m, 6H). ^{13}C NMR (75.5 MHz, $CDCl_3$): δ/ppm = 200.5, 155.8, 80.2, 58.5, 38.3, 28.4, 24.8, 23.2. $[\alpha]_D^{22}$ = –9 (*c* 1.00, $CHCl_3$). FT-IR: ν/cm^{-1} = 3352, 2958, 2932, 2871, 1689, 1507, 1455, 1391, 1366, 1249, 1164, 1045, 1010, 873, 779.

53, *tert*-Butyl (*E*)-(1-fluoro-5-methyl-1-(phenylsulfonyl)hex-1-en-3-yl)carbamate

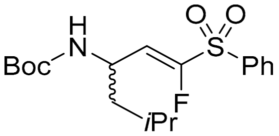

To a solution of **4** (451 mg, 1.45 mmol, 1 eq) and DBU (217 µL, 1.45 mmol, 1 eq) in dry MeCN (20 mL) was added anhydrous LiCl (74 mg, 1.74 mmol, 1.2 eq) at 0 °C under argon atmosphere. The solution was stirred for 20 min and a solution of **52** (313 mg, 1.45 mmol, 1 eq) in dry MeCN (6 mL) was added. After stirring for one hour at 0 °C under an argon atmosphere, citric acid solution (10% in water, 60 mL) was added. The mixture was extracted with EtOAc (3x 30 mL) and the combined organic layers were filtered over a short silica column. The filtrate was concentrated under reduced pressure and the residue was purified by column chromatography (CH/EA 10:1) to yield the desired product as a colorless oil (249 mg *E*-isomer + 183 mg *E*/*Z* mixture, ∑ 432 mg, 1.16 mmol, 80%). *E*-isomer: ^{1}H NMR (300 MHz, $CDCl_3$): *δ*/ppm = 7.97–7.90 (m, 2H), 7.73–7.63 (m, 1H), 7.62–7.51 (m, 2H), 6.26–5.96 (m, 1H), 4.64–4.35 (m, 2H), 1.66–1.25 (m, 12H), 0.92–0.85 (m, 6H). ^{13}C NMR (75.5 MHz, $CDCl_3$): *δ*/ppm = 155.0, 154.5 (d, *J* = 298.7 Hz), 137.3, 134.6, 129.6, 128.7, 119.2 (d, *J* = 5.3 Hz), 80.1, 45.1, 43.5, 28.3, 24.8, 22.5, 22.4. $[\alpha]_D^{22}$ = +21 (*c* 1.00, $CHCl_3$). FT-IR: ν/cm^{-1} = 3390, 2959, 2933, 2871, 1699, 1699, 1448, 1332, 1247, 1158, 1093, 1013, 754, 720, 686. MS (ESI) *m*/*z* calculated for $[C_{18}H_{26}FNO_4S+Na]^+$ ($[M+Na]^+$): 394.2, found: 394.1.

54, *tert*-Butyl ((2*S*)-1-(((*E*)-1-fluoro-5-methyl-1-(phenylsulfonyl)hex-1-en-3-yl)amino)-1-oxo-3-phenylpropan-2-yl)carbamate

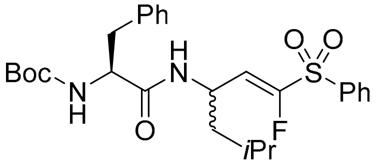

(1) **53** (223 mg, 0.60 mmol) was treated with HCl in dioxane (4 M, 3 mL) at room temperature for one hour. The solvent was removed under reduced pressure to yield the deprotected amine hydrochloride as a colorless solid (184 mg, 0.60 mmol, quantitative), which was directly used in the next step.

(2) To a 0 °C cold solution of Boc-Phe-OH (160 mg, 0.60 mmol, 1 eq) in DCM (5 mL) were added HOBt · H_2O (92 mg, 0.60 mmol, 1 eq) and 2,4,6-collidine (160 µL, 1.20 mmol, 2 eq). After stirring at 0 °C for 30 min, TBTU (194 g, 0.60 mmol, 1 eq) was added. The solution was stirred for further 30 min at 0 °C and deprotected **53** (184 mg, 0.60 mmol, 1 eq) was added. After stirring at room temperature overnight, the solvent was removed under reduced pressure and the residue was taken up in EtOAc (30 mL). The mixture was washed with saturated $NaHCO_3$ solution (3x 25 0mL) and hydrochloric acid (1 M, 3x 25 mL) and filtered over a small silica column. The filtrate was concentrated under reduced pressure to yield the desired product as a colorless foam (302 mg, 0.58 mmol, 97%). ^{1}H NMR (300 MHz, $CDCl_3$): *δ*/ppm = 7.96–7.89 (m, 2H), 7.73–7.65 (m, 1H), 7.62–7.54 (m, 2H), 7.33–7.22 (m, 3H), 7.17–7.12 (m, 2H), 6.16–5.86 (m, 2H), 5.17–5.01 (m, 1H), 4.81–

4.67 (m, 1H), 4.30–4.18 (m, 1H), 3.08–2.89 (m, 2H), 1.52–1.44 (m, 1H), 1.41–1.38 (m, 9H), 1.34–1.22 (m, 1H), 0.85–0.77 (m, 6H). ^{13}C NMR (75.5 MHz, $CDCl_3$): δ/ppm = 170.9, 155.6, 154.9 (d, J = 299.7 Hz), 137.2, 136.6, 134.7, 129.6, 129.4, 128.9, 128.7, 127.2, 118.1 (d, J = 5.1 Hz), 80.5, 56.1, 43.5, 43.2, 38.5, 28.4, 24.7, 22.6, 22.1. mp: 47–49 °C. $[\alpha]_D^{22}$ = +11 (c 1.00, $CHCl_3$). FT-IR: ν/cm^{-1} = 3302, 3064, 3031, 2959, 2931, 2871, 1655, 1521, 1366, 1333, 1165, 1092, 753, 720, 686. MS (ESI) m/z calculated for $[C_{27}H_{35}FN_2O_5S+Na]^+$ ($[M+Na]^+$): 541.2, found: 541.2.

55, *N*-((*S*)-1-(((*S*,*E*)-1-fluoro-5-methyl-1-(phenylsulfonyl)hex-1-en-3-yl)amino)-1-oxo-3-phenylpropan-2-yl)pyrazine-2-carboxamide

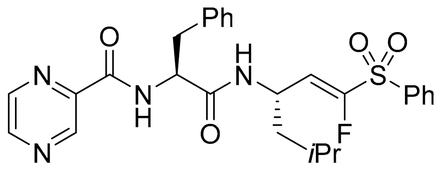

(1) **54** (254 mg, 0.49 mmol) was treated with HCl in dioxane (4 M, 3 mL) at room temperature for 1.5 h. The solvent was removed under reduced pressure to yield the amine hydrochloride as a colorless solid (224 mg, 0.49 mmol, quantitative), which was directly used in the next step.

(2) To a 0 °C cold solution of pyrazinecarboxylic acid (61 mg, 0.49 mmol, 1 eq) in DCM (6 mL) were added HOBt · H_2O (75 mg, 0.49 mmol, 1 eq) and 2,4,6-collidine (130 µL, 0.98 mmol, 2 eq). After stirring at 0 °C for 30 min, TBTU (158 g, 0.49 mmol, 1 eq) was added. The solution was stirred for further 30 min at 0 °C and the amine hydrochloride (224 mg, 0.49 mmol, 1 eq) was added. After stirring at room temperature overnight, the solvent was removed under reduced pressure and the residue was taken up in EtOAc (30 mL). The mixture was washed with saturated $NaHCO_3$ solution (3x 25 mL) and hydrochloric acid (1 M, 3x 25 mL) and dried over anhydrous Na_2SO_4. After the solvent was removed under reduced pressure, the residue was purified by column chromatography (CH/EA 1:1) to yield the product as a mixture of two diastereomers (colorless resin, 174 mg, 0.33 mmol, 67%). 40 mg diastereomeric mixture were separated by preparative HPLC (MeCN/H_2O 25:75) to give 20 mg pure (*S*/*S*) and 10 mg pure (*S*/*R*) diastereomer. (*S*/*S*) diastereomer: ^{1}H NMR (300 MHz, $CDCl_3$): δ/ppm = 9.34–9.25 (m, 1H), 8.75 (d, J = 2.5 Hz, 1H), 8.57–8.49 (m, 1H), 8.42 (d, J = 8.2 Hz, 1H), 7.98–7.91 (m, 2H), 7.73–7.66 (m, 1H), 7.63–7.55 (m, 2H), 7.32–7.16 (m, 5H), 6.19–6.14 (m, 1H), 5.93 (dd, J = 31.9, 8.9 Hz, 1H), 4.83–4.71 (m, 2H), 3.23–3.05 (m, 2H), 1.44–1.36 (m, 1H), 1.34–1.21 (m, 2H), 0.79–0.74 (m, 6H). ^{13}C NMR (75.5 MHz, $CDCl_3$): δ/ppm = 169.8, 163.0, 154.6 (d, J = 300.1 Hz), 147.7, 144.2, 144.0, 143.0, 137.2, 136.3, 134.7, 129.6, 129.4, 128.0, 129.7, 127.4, 117.9 (d, J = 5.3 Hz), 55.0, 43.6, 43.1, 38.8, 24.7, 22.4, 22.3. mp: 68 – 70 °C. $[\alpha]_D^{22}$ = +1 (c 1.00, $CHCl_3$). FT-IR: ν/cm^{-1} = 3368, 3302, 3063, 3030, 2957, 2927, 2870, 1652, 1519, 1332, 1167, 1019, 752, 720, 685. MS (ESI) m/z calculated for $[C_{27}H_{29}FN_4O_4S+H]^+$ ($[M+H]^+$): 525.2, found: 525.1. Purity: 99%.

56, *tert*-Butyl ((S)-1-(((S)-1-(methoxy(methyl)amino)-4-methyl-1-oxopentan-2-yl)amino)-1-oxo-3-phenylpropan-2-yl)carbamate

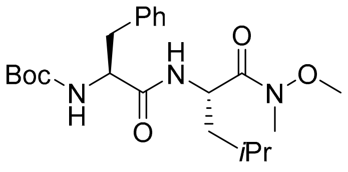

51 (15.74 g, 57.37 mmol) was treated with HCl in dioxane (4 M, 50 mL) at room temperature for four hours. The solvent was removed under reduced pressure to yield the deprotected amine hydrochloride as a colorless solid (12.05 g, 57.17 mmol, quantitative). To a solution of Boc-Phe-OH (14.92 g, 56.24 mmol, 1 eq) in DCM (500 mL) at 0 °C were added HOBt · H_2O (8.50 g, 56.24 mmol, 1 eq) and 2,4,6-collidine (14.9 mL, 112.48 mmol, 2 eq) and stirred at 0 °C for 15 min. TBTU (18.06 g, 56.24 mmol, 1 eq) was then added, stirred for another 30 min at 0 °C, and the deprotected amine hydrochloride (11.85 g, 56.24 mmol, 1 eq) was added. Stirring was carried out for three hours at 0 °C and overnight at room temperature. The solvent was then removed by distillation under reduced pressure and the residue was suspended in EtOAc (300 mL). It was filtered, the filter cake was washed with EtOAc, and the filtrate was washed successively with brine (2x 100 mL), saturated $NaHCO_3$ solution (2x 100 mL), saturated NH_4Cl solution (2x 100 mL), and again with brine (2x 100 mL). The organic phase was dried over anhydrous Na_2SO_4, the solvent was removed by distillation under reduced pressure, and the residue was dried under fine vacuum, whereupon the title compound was obtained as a colorless solid (22.95 g, 54.45 mmol, 97%). ^{1}H NMR (300 MHz, $CDCl_3$): δ/ppm = 7.32–7.12 (m, 5H), 6.55 (d, J = 8.9 Hz, 1H), 5.15–4.87 (m, 2H), 4.45–4.28 (m, 1H), 3.76 (s, 3H), 3.18 (s, 3H), 3.14–2.92 (m, 2H), 1.71–1.40 (m, 3H), 1.39 (s, 9H), 0.94 (d, J = 6.4 Hz, 3H), 0.84 (d, J = 6.4 Hz, 3H). ^{13}C NMR (75.5 MHz, $CDCl_3$): δ/ppm = 172.8, 171.1, 155.4, 136.8, 129.5, 128.6, 126.9, 80.1, 61.7, 55.7, 47.6, 42.0, 38.3, 32.2, 28.3, 24.7, 23.4, 23.8. mp: 165 – 167 °C. $[\alpha]_D^{22}$ = −21 (c 1.00, $CHCl_3$). FT-IR: ν/cm^{-1} = 2954, 1662, 1499, 1466, 1438, 1365, 1174, 1133, 986, 701. MS (ESI) m/z calculated for $[C_{22}H_{35}N_3O_5+H]^+$ ($[M+H]^+$): 422.3, found: 422.3.

57, *N*-((*S*)-1-(((*S*)-1-(Methoxy(methyl)amino)-4-methyl-1-oxopentan-2-yl)amino)-1-oxo-3-phenylpropan-2-yl)pyrazine-2-carboxamide

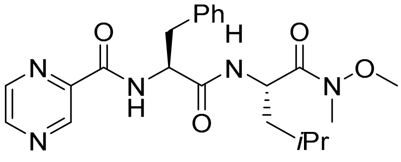

56 (22.80 g, 54.09 mmol) was treated with HCl in dioxane (4 M, 70 mL) at room temperature for 2 h. The solvent was removed under reduced pressure to yield the deprotected amine hydrochloride as a colorless oil (19.33 g, 54.09 mmol, quantitative). To a solution of pyrazinecarboxylic acid (6.60 g, 53.20 mmol, 1 eq) in DCM (300 mL) at 0 °C were added HOBt · H_2O (8.04 g, 53.20 mmol, 1 eq) and 2,4,6-collidine (14.1 mL, 106.40 mmol, 2 eq) and stirred at 0 °C for 15 min. TBTU (17.08 g, 53.20 mmol, 1 eq) was then added, stirred for another 45 min at 0 °C, and the deprotected amine hydrochloride (19.04 g, 53.20 mmol, 1 eq)

was added. Stirring was carried out for three hours at 0 °C and overnight at room temperature. The solvent was then removed by distillation under reduced pressure and the residue was suspended in EtOAc (300 mL). It was filtered, the filter cake was washed with EtOAc, and the filtrate was successively washed with brine (2x 100 mL), saturated $NaHCO_3$ solution (2x 100 mL), saturated NH_4Cl solution (2x 100 mL), and again with brine (2x 100 mL). The organic phase was dried with anhydrous Na_2SO_4, the solvent was removed by distillation under reduced pressure, and the residue was dried under fine vacuum, whereupon the title compound was obtained as a yellowish solid (20.04 g, 46.88 mmol, 88%). ^1H NMR (300 MHz, $CDCl_3$): δ/ppm = 9.36–9.24 (m, 1H), 8.66 (d, J = 2.5 Hz, 1H), 8.47–8.43 (m, 1H), 8.41 (d, J = 8.6 Hz), 7.35 (d, J = 8.8 Hz, 1H), 7.25–7.01 (m, 5H), 5.18–4.91 (m, 2H), 3.73 (s, 3H), 3.27–3.01 (m, 5H), 1.68–1.35 (m, 3H), 0.84 (d, J = 6.3 Hz, 3H), 0.79 (d, J = 6.3 Hz, 3H). ^{13}C NMR (75.5 MHz, $CDCl_3$): δ/ppm = 172.8, 170.4, 162.7, 147.3, 144.3, 144.1, 142.7, 136.3, 129.3, 128.3, 126.8, 61.6, 53.9, 47.5, 41.6, 38.8, 32.1, 24.7, 23.1, 21.7. mp: 62 – 64 °C. $[\alpha]_D^{22}$ = −26 (c 1.00, $CHCl_3$). FT-IR: ν/cm^{-1} = 3307, 2956, 1643, 1518, 1465, 1387, 1241, 1168, 1019, 983, 745, 699. MS (ESI) m/z calculated for $[C_{22}H_{29}N_5O_4+H]^+$ ($[M+H]^+$): 428.2, found: 428.2.

58, *N*-((*S*)-1-(((*S,E*)-5-Methyl-1-(phenylsulfonyl)hex-1-en-3-yl)amino)-1-oxo-3-phenylpropan-2-yl)pyrazine-2-carboxamide

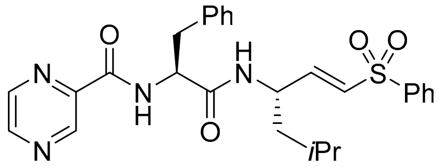

(1) Under argon atmosphere, $LiAlH_4$ (89 mg, 2.34 mmol, 2 eq) was added portionwise to a solution of **57** (500 mg, 1.17 mmol, 1 eq) in dry THF (20 mL) at 0 °C and stirred for one hour at 0 °C. The reaction mixture was quenched by addition of $KHSO_4$ solution (0.3 M, 50 mL), filtered, and extracted with EtOAc (3x 50 mL). The combined organic extracts were washed with saturated $NaHCO_3$ solution (2x 100 mL), dried over anhydrous Na_2SO_4, and the solvent was removed by distillation under reduced pressure. The peptidylaldehyde (ca. 200 mg) obtained in the form of an orange oil was directly used in the next step.

(2) To a solution of **3** (155 mg, 0.53 mmol, 1 eq) and DBU (79 µL, 0.53 mmol, 1 eq) in dry MeCN (20 mL) was added dried LiCl (27 mg, 0.64 mmol, 1.2 eq) at 0 °C under argon atmosphere. The solution was stirred for 20 min and a solution of the peptidylaldehyde in dry MeCN (6 mL) was added. After stirring for one hour at 0 °C and argon atmosphere, citric acid solution (10% in water, 60 mL) was added. The mixture was extracted with EtOAc (3x 30 mL) and the combined organic extracts were filtered over a short silica column. The filtrate was concentrated under reduced pressure and the residue was purified preparative HPLC to yield the desired product ((*S*/*S*)-isomer) as a colorless solid (29 mg, 0.06 mmol, 11%). ^1H NMR (600 MHz, $CDCl_3$): δ/ppm = 9.33 (s, 1H), 8.77 (s, 1H), 8.54 (s, 1H), 8.32 (d, J = 8.0 Hz, 1H), 7.87–7.84 (m, 2H), 7.67–7.63 (m, 1H), 7.59–7.55 (m, 2H), 7.25–7.18 (m, 5H), 6.74 (dd, J = 15.1, 5.0 Hz, 1H), 6.07 (dd, J = 15.1, 1.6 Hz, 1H), 5.93–5.86 (m, 1H), 4.77–4.71 (m, 1H), 4.70–4.63 (m, 1H), 3.20–3.10 (m, 2H), 1.53–1.46 (m, 1H), 1.35–1.31 (m, 2H), 0.83–0.80 (m, 6H). ^{13}C NMR (150 MHz, $CDCl_3$): δ/ppm = 169.9, 163.3, 147.8, 145.8, 144.3, 143.9, 143.0, 140.2, 136.1, 133.7, 130.3, 129.5, 129.3, 129.1,

127.8, 127.6, 55.2, 48.2, 43.1, 38.3, 24.7, 22.8, 22.0. mp: 78 – 81 °C. $[\alpha]_D^{22}$ = −22 (*c* 1.00, $CHCl_3$). FT-IR: *v*/cm^{-1} = 3299, 3062, 2957, 2929, 2870, 1656, 1517, 1446, 1306, 1144, 1085, 1020, 751, 719, 687. MS (ESI) *m*/*z* calculated for $[C_{27}H_{30}N_4O_4S+H]^+$ ($[M+H]^+$): 507.2, found: 507.1. Purity: 99%.

59, *N*-((*S*)-1-(((*S*)-5-Methyl-1-nitrohex-1-en-3-yl)amino)-1-oxo-3-phenylpropan-2-yl)pyrazine-2-carboxamide

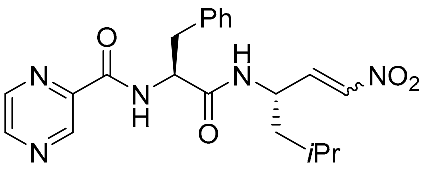

(1) Under argon atmosphere, $LiAlH_4$ (178 mg, 4.68 mmol, 2 eq) was added portionwise to a solution of **57** (1.00 g, 2.34 mmol, 1 eq) in dry THF (40 mL) at 0 °C and stirred for one hour at 0 °C. The reaction mixture was quenched by addition of a citric acid solution (10% in water, 20 mL) and extracted with EtOAc (3x 50 mL). The combined organic phases were washed with saturated $NaHCO_3$ solution (2x 50 mL), with brine (100 mL), dried over anhydrous Na_2SO_4, and the solvent was removed by distillation under reduced pressure. The peptidylaldehyde obtained in the form of an orange oil was directly used in the next step.
(2) To a solution of peptidylaldehyde (718 mg, 1.95 mmol, 1 eq) in DCM (5 mL) was added $MeNO_2$ (626 µL, 11.70 mmol, 6 eq) and Et_3N (81 µL, 0.59 mmol, 0.3 eq) at 0 °C and stirred for one hour at 0 °C and overnight at room temperature. Saturated NH_4Cl solution (10 mL) was then added, and the reaction mixture was extracted with DCM (3x 50 mL). The combined organic extracts were washed with saturated $NaHCO_3$ solution (50 mL) and with brine (50 mL), dried over anhydrous Na_2SO_4, and the solvent was removed by distillation under reduced pressure. The obtained nitroaldol product was used in the next step without further purification.
(3) To a solution of the nitroaldol (2, 837 g, 1.95 mmol, 1 eq) in dry DCM (10 mL) was added under argon atmosphere DIPEA (1.3 mL, 7.80 mmol, 4 eq) and MsCl (302 µL, 3.90 mmol, 2 eq) and stirred for three hours at room temperature. The reaction mixture was quenched by addition of saturated NH_4Cl solution (10 mL) and extracted with DCM (3x 50 mL). The combined organic phases were successively washed with 1 M HCl (1x 50 mL), with saturated $NaHCO_3$ solution (1x 50 mL), and with brine (1x 50 mL), dried over anhydrous Na_2SO_4, and concentrated under reduced pressure. After purification by column chromatography ($CHCl_3$/MeOH 200:1), the title compound was obtained as a colorless oil (149 mg, 0.36 mmol, 19% over three steps). The product was obtained as a mixture of two *E*/*Z* isomers (*E*/*Z* = 1:1). ^1H NMR (600 MHz, $CDCl_3$): *δ*/ppm = 9.34 (s, 1H), 8.78 (d, *J* = 2.5 Hz, 1H), 8.60–8.51 (m, 1H), 8.42–8.30 (m, 1H), 7.37–7.15 (m, 5H), 7.04–6.97 (m, 2H, (*Z*)), 6.85 (dd, *J* = 13.4 Hz, *J* = 6.3 Hz, 1H, (*E*)), 6.69 (dd, *J* = 13.4 Hz, *J* = 1.3 Hz, 1H, (*E*)), 6.16 and 6.07 (2 × d, *J* = 8.2 Hz, 2 × 1H, (*E*) and (*Z*)), 4.88–4.73 (m, 1H), 4.68–4.57 (m, 2H), 3.31–3.08 (m, 2H), 1.41–1.20 (m, 3H,), 0.97–0.72 (m, 6H). ^{13}C NMR (150 MHz, $CDCl_3$): *δ*/ppm = 170.2, 170.1, 163.6, 163.3, 148.0, 147.9, 144.5, 144.4, 143.8, 143.7, 143.01, 142.98, 141.4, 140.9, 140.1, 140.0, 136.2, 136.0, 129.4, 129.1, 129.0, 127.7, 127.4,

55.4, 55.2, 46.3, 46.1, 42.9, 42.8, 38.5, 38.1, 24.7, 24.5, 22.7, 22.5, 22.1, 22.0. $[\alpha]_D^{22} = -18$ (*c* 1.00, $CHCl_3$). FT-IR: ν/cm^{-1} = 3288, 2957, 1650, 1518, 1466, 1350, 1240, 1152, 1020, 748, 699. MS (ESI) *m*/*z* calculated for $[C_{21}H_{25}N_5O_4+H]^+$ ($[M+H]^+$): 412.2, found: 412.2. Purity: 97%.

60, *tert*-Butyl (1-(benzo[*d*]thiazol-2-yl)-4-methyl-1-oxopentan-2-yl)carbamate

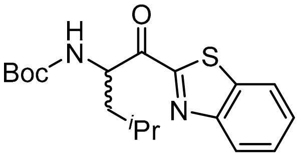

To a solution of benzothiazole (2.66 g, 19.68 mmol, 10 eq) in dry THF (50 mL) at −75 °C was added *n*-BuLi (2.5 M in *n*-hexane, 5.5 mL, 13.78 mmol, 7 eq) dropwise over 15 min. The mixture was stirred at −75 °C for one hour and a solution of **51** (540 mg, 1.97 mmol, 1 eq) in dry THF (10 mL) was added. After stirring was continued at −75 °C for five hours, saturated NH_4Cl solution (80 mL) was added. The mixture was extracted with EtOAc (3x 80 mL) and the combined organic extracts were dried over anhydrous Na_2SO_4. The solvent was removed by distillation under reduced pressure and the residue was purified by column chromatography (CH/EA 20:1) to yield the desired product as a yellowish solid (448 mg, 1.29 mmol, 65%). ^{1}H NMR (300 MHz, $CDCl_3$): δ/ppm = 8.22–8.17 (m, 1H), 8.01–7.95 (m, 1H), 7.62–7.49 (m, 2H), 5.71–5.54 (m, 1H), 5.33–5.18 (m, 1H), 1.90–1.77 (m, 2H), 1.60–1.50 (m, 1H), 1.43 (s, 9H), 1.09 (d, *J* = 6.0 Hz, 3H), 0.96 (d, *J* = 6.3 Hz, 3H). ^{13}C NMR (75.5 MHz, $CDCl_3$): δ/ppm = 194.9, 164.2, 155.6, 153.7, 137.4, 128.0, 127.1, 125.95, 122.5, 80.0, 55.4, 42.2, 28.4, 25.4, 23.4, 21.9. mp: 101–103 °C. $[\alpha]_D^{22} = +33$ (*c* 1.00, $CHCl_3$). FT-IR: ν/cm^{-1} = 3362, 2965, 2930, 2871, 1702, 1679, 1518, 1481, 1366, 1230, 1162, 878, 828, 759, 731. MS (ESI) *m*/*z* calculated for $[C_{18}H_{24}N_2O_3S+Na]^+$ ($[M+Na]^+$): 371.1, found: 371.1.

61, *tert*-Butyl ((2*S*)-1-((1-(benzo[d]thiazol-2-yl)-4-methyl-1-oxopentan-2-yl)amino)-1-oxo-3-phenylpropan-2-yl)carbamate

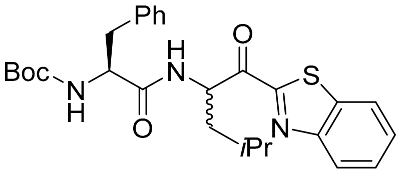

(1) **60** (274 mg, 0.79 mmol) was treated with HCl in dioxane (4 M, 4 mL) at room temperature for one hour. The solvent was removed under reduced pressure to yield the deprotected amine hydrochloride as a colorless solid (223 mg, 0.79 mmol, quantitative), which was directly used in the next step.
(2) To a 0 °C cold solution of Boc-Phe-OH (208 mg, 0.78 mmol, 1 eq) in DCM (6 mL) were added HOBt · H_2O (120 mg, 0.78 mmol, 1 eq) and 2,4,6-collidine (208 µL, 1.57 mmol, 2 eq). After stirring at 0 °C for 30 min, TBTU (252 g, 0.78 mmol, 1 eq) was added. The solution was stirred for further 30 min at 0 °C and the deprotected amine hydrochloride (223 mg, 0.78 mmol, 1 eq) was added. After stirring at rt overnight, the solvent was

removed under reduced pressure and the residue was taken up in EtOAc (30 mL). The mixture was washed with saturated $NaHCO_3$ solution (3x 25 mL) and hydrochloric acid (1 M, 3x 25 mL) and filtered over a small silica column. The filtrate was concentrated under reduced pressure to yield the desired product as a yellowish solid (264 mg, 0.53 mmol, 68%). ^{1}H NMR (300 MHz, $CDCl_3$): δ/ppm = 8.20–8.13 (m, 1H), 8.00–7.94 (m, 1H), 7.62–7.50 (m, 2H), 7.24–6.95 (m, 5H), 6.79 (d, J = 8.5 Hz, 1H), 5.79–5.65 (m, 1H), 5.23–5.02 (m, 1H), 4.48–4.31 (m, 1H), 3.14–2.95 (m, 2H), 1.84–1.51 (m, 3H), 1.4 –1.39 (m, 9H), 1.03 (d, J = 6.2 Hz, 3H), 0.90 (d, J = 6.3 Hz, 3H). ^{13}C NMR (75.5 MHz, $CDCl_3$): δ/ppm = 193.4 (diastereomer B), 192.9 (diastereomer A), 171.1 (diastereomer B), 171.0 (diastereomer A), 164.0, 155.5, 153.6 (diastereomer B), 153.5 (diastereomer A), 137.3 (diastereomer B), 137.2 (diastereomer A), 136.7, 129.3, 128.7 (diastereomer B), 128.6 (diastereomer A), 128.1, 127.2, 127.0 (diastereomer B), 126.8 (diastereomer A), 125.9, 122.4, 80.3, 56.3, 54.6 (diastereomer A), 54.3 (diastereomer B), 42.2 (diastereomer A), 41.8 (diastereomer B), 38.7 (diastereomer A), 38.6 (diastereomer B), 28.4, 25.2, 23.3 (diastereomer B), 23.2 (diastereomer A), 21.9 (diastereomer A), 21.7 (diastereomer B). mp: 133–135 °C. $[\alpha]_D^{22}$ = −13 (c 1.00, $CHCl_3$). FT-IR: ν/cm^{-1} = 3275, 3065, 2962, 2930, 2870, 1686, 1639, 1518, 1366, 1248, 1169, 885, 758, 726, 699. MS (ESI) m/z calculated for $[C_{27}H_{33}N_3O_4S+Na]^+$ ($[M+Na]^+$): 518.2, found: 518.0.

62, *N*-((*S*)-1-(((*S*)-1-(Benzo[*d*]thiazol-2-yl)-4-methyl-1-oxopentan-2-yl)amino)-1-oxo-3-phenylpropan-2-yl)pyrazine-2-carboxamide

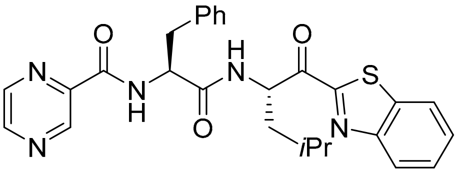

(1) **61** (223 mg, 0.45 mmol) was treated with HCl in dioxane (4 M, 4 mL) at room temperature for one hour. The solvent was removed under reduced pressure to yield the deprotected amine hydrochloride as a colorless solid (194 mg, 0.45 mmol, quantitative), which was directly used in the next step.

(2) To a 0 °C cold solution of pyrazinecarboxylic acid (56 mg, 0.45 mmol, 1 eq) in DCM (6 mL) were added HOBt · H_2O (69 mg, 0.45 mmol, 1 eq) and 2,4,6-collidine (120 µL, 0.90 mmol, 2 eq). After stirring at 0 °C for 30 min, TBTU (145 g, 0.45 mmol, 1 eq) was added. The solution was stirred for further 30 min at 0 °C and the deprotected amine hydrochloride (194 mg, 0.45 mmol, 1 eq) was added. After stirring at room temperature overnight, the solvent was removed under reduced pressure and the residue was taken up in EtOAc (30 mL). The mixture was washed with saturated $NaHCO_3$ solution (3x 25 mL) and hydrochloric acid (1 M, 3x 25 mL) and filtered over a small silica column. The filtrate was concentrated under reduced pressure and the residue was purified by column chromatography (CH/EA 2:1) to yield the desired product as a colorless solid (mixture of two diastereomers, ∑ 119 mg, 0.24 mmol, 53%). Separation of the diastereomers was achieved by preparative HPLC. (*S*/*S*)-diastereomer: ^{1}H NMR (300 MHz, $CDCl_3$): δ/ppm = 9.35 (d, J = 1.5 Hz, 1H), 8.75 (d, J = 2.5 Hz, 1H), 8.53 (dd, J = 2.5, 1.5 Hz, 1H), 8.44 (d, J

= 8.2 Hz, 1H), 8.13–8.08 (m, 1H), 8.01–7.96 (m, 1H), 7.61–7.50 (m, 2H), 7.25–7.19 (m, 2H), 7.14–7.07 (m, 2H), 7.03–6.96 (m, 1H), 6.77 (d, *J* = 8.3 Hz, 1H), 5.72–5.63 (m, 1H), 4.96–4.87 (m, 1H), 3.28–3.08 (m, 2H), 1.82–1.75 (m, 1H), 1.67–1.53 (m, 2H), 0.97 (d, *J* = 6.3 Hz, 3H), 0.87 (d, *J* = 6.3 Hz, 3H). ^{13}C NMR (75.5 MHz, $CDCl_3$): δ/ppm = 192.7, 170.1, 164.0, 163.0, 153.5, 147.6, 144.4, 144.2, 142.9, 137.2, 136.4, 129.3, 128.7, 128.1, 127.2, 127.0, 125.9, 122.5, 55.1, 54.9, 42.1, 38.9, 25.3, 23.1, 22.0. mp: 65 – 67 °C. $[\alpha]_D^{22} = -12$ (*c* 1.00, $CHCl_3$). FT-IR: ν/cm^{-1} = 3307, 3061, 2956, 2925, 2869, 1651, 1519, 1370, 1207,1155, 1019, 882, 760, 730, 699. MS (ESI) *m*/*z* calculated for $[C_{27}H_{27}N_5O_3S+H]^+$ ($[M+H]^+$): 502.2, found: 502.1. Purity: 99%.

64, Methyl (*tert*-butoxycarbonyl)-L-leucinate

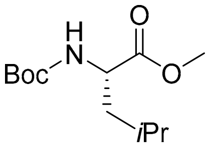

(Adapted from R. Spina, *et al.*)[23]

H_2N-Leu-OMe · HCl **63** (10.00 g, 55.05 mmol, 1 eq) was added to a solution of $NaHCO_3$ (9.25 g, 110.10 mmol, 2 eq) in water (100 mL) and a solution of Boc_2O (13.22 g, 60.56 mmol, 1.1 eq) in 1,4-dioxane (100 mL) was added dropwise at room temperature. Stirring was carried out for three hours at room temperature and the organic solvent was removed by distillation under reduced pressure. The aqueous phase was extracted with EtOAc (3x 100 mL), and the combined organic extracts were dried over anhydrous Na_2SO_4 and concentrated to yield the title compound as a colorless oil (13.50 g, 55.03 mmol, 99%). ^{1}H NMR (300 MHz, $CDCl_3$): δ/ppm = 4.96 (d, *J* = 8.5 Hz, 1H), 4.33–4.15 (m, 1H), 3.66 (s, 3H), 1.77–1.40 (m, 3H), 1.37 (s, 9H), 0.88 (d, *J* = 6.5 Hz, 3H), 0.87 (d, *J* = 6.5 Hz, 3H). ^{13}C NMR (75.5 MHz, $CDCl_3$): δ/ppm =174.0, 155.5, 79.7, 52.1, 41.8, 28.3, 24.8, 22.8. $[\alpha]_D^{22} = -4$ (*c* 1.00, $CHCl_3$). FT-IR: ν/cm^{-1} = 3362, 2958, 2359, 1714, 1510, 1437, 1366, 1249, 1160, 1047, 1019, 780. MS (ESI) *m*/*z* calculated for $[C_{12}H_{23}NO_4+H]^+$ ($[M+H]^+$): 246.2, found: 146.3 ($[M-Boc+H]^+$).

65, *tert*-Butyl (1-(dimethoxyphosphoryl)-5-methyl-2-oxohexan-3-yl)carbamate

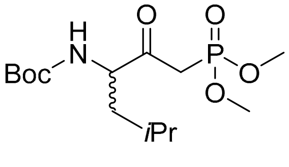

(Adapted from A. Ho *et al.*)[24]

A solution of *n*-BuLi (2.5 M in hexane, 55.4 mL, 138.60 mmol, 4 eq) was slowly added to a solution of dimethyl methyl phosphonate (14.8 mL, 138.60 mmol, 4 eq) in dry THF (200 mL) at −78 °C under argon atmosphere. Stirring was continued for one hour at −78 °C and then a solution of **64** (8.50 g, 34.65 mmol, 1 eq) in dry THF (25 mL) was added dropwise and stirred for an additional five hours at −78 °C. The reaction mixture was quenched by addition of water (50 mL) and extracted with EtOAc (3x 200 mL). The combined organic phases were dried over

anhydrous Na_2SO_4, and the solvent was removed by distillation under reduced pressure. After purification by column chromatography (CH/EA 1:1 to 1:2), the title compound was obtained as a colorless oil (10.24 g, 30.35 mmol, 88%). ^{1}H NMR (300 MHz, $CDCl_3$): δ/ppm = 5.25 (d, J = 8.5 Hz, 1H), 4.35–4.21 (m, 1H), 3.75 (d, J = 11.2 Hz, 3H), 3.74 (d, J = 11.2 Hz, 3H, 3.28 (dd, J = 22.5 Hz, J = 14.2 Hz, 1H), 3.06 (dd, J = 22.0 Hz, J = 14.2 Hz, 1H), 1.76–1.47 (m, 2H), 1.39 (s, 9H), 1.37–1.30 (m, 1H), 0.90 (d, J = 6.3 Hz, 3H), 0.89 (d, J = 6.3 Hz, 3H). ^{13}C NMR (75.5 MHz, $CDCl_3$): δ/ppm = 202.5 (d, J = 6.7 Hz), 155.7, 80.0, 58.9, 53.1 (d, J = 6.5 Hz), 39.8, 38.0 (d, J = 130.4 Hz), 28.3, 24.8, 23.7, 21.6. $[\alpha]_D^{22}$ = −41 (c 1.00, MeOH). FT-IR: ν/cm^{-1} = 3236, 2955, 1724, 1698, 1536, 1364, 1298, 1276, 1238, 1166, 1059, 1032, 854, 824. MS (ESI) m/z calculated for $[C_{14}H_{28}NO_6P+H]^+$ ($[M+H]^+$): 338.2, found: 238.2 ($[M–Boc+H]^+$).

66, *tert*-Butyl ((2*S*)-1-((1-(dimethoxyphosphoryl)-5-methyl-2-oxohexan-3-yl)amino)-1-oxo-3-phenylpropan-2-yl)carbamate

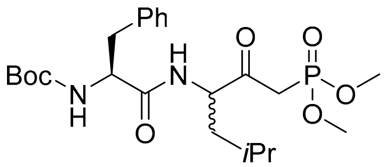

65 (6.00 g, 17.79 mmol) was treated with HCl in dioxane (4 M, 35 mL) at room temperature for one hour. The solvent was removed under reduced pressure to yield the deprotected amine hydrochloride as a colorless oil (4.86 g, 17.76 mmol, quantitative). To a solution of Boc-Phe-OH (1.32 g, 4.98 mmol, 1 eq) in DCM/DMF (1:1, 50 mL) at 0 °C were added HOBt · H_2O (753 mg, 4.98 mmol, 1 eq), TBTU (1.60 g, 4.98 mmol, 1 eq), and 2,4,6-collidine (1.32 mL, 9.96 mmol, 2 eq) were added and stirred at 0 °C for 5 min. A solution of the deprotected amine hydrochloride (1.50 g, 5.48 mmol, 1.1 eq) in DCM (10 mL) was then added dropwise and stirred for three hours at 0 °C and overnight at room temperature. Brine (50 mL) was added to the reaction mixture and extracted with DCM (3x 100 mL). The combined organic extracts were successively washed with saturated NH_4Cl solution (2x 50 mL), saturated $NaHCO_3$ solution (50 mL), and brine (100 mL), dried with anhydrous Na_2SO_4, and the solvent was removed by distillation under reduced pressure. After purification by column chromatography (DCM/MeOH 50:1), the title compound was obtained as a colorless solid (1.73 g, 3.58 mmol, 72%). The product was present as a mixture of two diastereomers (A/B = 5:1). $[\alpha]_D^{20}$ = −22 (c=0.1, $CHCl_3$). mp: 106–108 °C. ^{1}H NMR (300 MHz, $CDCl_3$): δ/ppm =7.28–7.08 (m, 5H), 6.86 (d, J = 8.1 Hz, 1H, diastereomer B), 6.78 (d, J = 8.5 Hz, diastereomer A), 5.08 (d, J = 7.9 Hz, 1H, diastereomer B), 4.93 (d, J = 8.1 Hz, 1H, diastereomer A), 4.63–4.43 (m, 1H), 4.40–4.21 (m, 1H), 3.69 (d, J = 11.2 Hz, 3H), 3.68 (d, J = 11.2 Hz, 3H), 3.13–2.79 (m, 4H), 1.63–1.10 (m, 3H), 1.34 (s, 9H, diastereomer A), 1.32 (s, 9H, diastereomer B), 0.88–0.79 (m, 6H, diastereomer A), 0.79–0.71 (m, 6H, Diastereomer B). ^{13}C NMR (75.5 MHz, $CDCl_3$): δ/ppm = 201.4 (d, J = 6.8 Hz), 171.8 (diastereomer B), 171.5 (diastereomer A), 154.8, 136.6, 129.5 (diastereomer A), 129.4 (diastereomer B), 128.8, 127.1, 80.4, 57.5 (d, J = 1.9 Hz), 55.9, 53.3 (d, J = 6.7 Hz, one diastereomer), 53.2 (d, J = 6.0 Hz (other diastereomer), 39.8, 38.3 (d, J = 129.4 Hz), 38.0, 28.3, 24.7, 23.3, 21.5. $[\alpha]_D^{22}$ = −22 (c 1.00, $CHCl_3$). FT-IR: ν/cm^{-1} = 3289, 2957, 1716, 1662, 1521, 1455, 1366, 1246, 1170, 1029, 816, 750, 700. MS (ESI) m/z calculated for $[C_{23}H_{37}N_2O_7P+Na]^+$ ($[M+Na]^+$): 507.2, found: 507.2.

67, Dimethyl ((*S*)-5-methyl-2-oxo-3-((*S*)-3-phenyl-2-(pyrazine-2-carboxamido)propanamido)hexyl)phosphonate

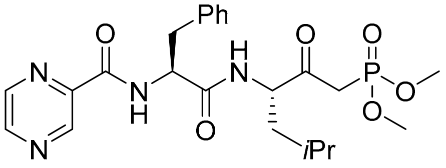

66 (1.56 g, 3.22 mmol) was treated with HCl in dioxane (4 M,10 mL) at room temperature for one hour. The solvent was removed under reduced pressure to yield the deprotected amine hydrochloride as a yellowish oil (1.35 g, 3.21 mmol, quantitative). To a solution of pyrazinecarboxylic acid (341 mg, 2.75 mmol, 1 eq) in DCM/DMF (1:1, 30 mL) at 0 °C were added HOBt · H_2O (416 mg, 2.75 mmol, 1 eq), TBTU (883 mg, 2.75 mmol, 1 eq), and 2,4,6-collidine (730 µL, 5.50 mmol, 2 eq) were added and stirred at 0 °C for 30 min. A solution of the deprotected amine hydrochloride (1.27 g, 3.03 mmol, 1.1 eq) in DCM (5 mL) was then added dropwise and stirred for six hours at 0 °C and overnight at room temperature. Water (100 mL) was added to the reaction mixture and extracted with EtOAc (3x 100 mL). The combined organic extracts were successively washed with saturated NH_4Cl solution (1x 100 mL), water (2x 100 mL), and brine (1x 100 mL), dried over anhydrous Na_2SO_4, and the solvent was removed by distillation under reduced pressure. After purification by column chromatography ($CHCl_3$/MeOH 100:1), the separated diastereomers were each obtained as colorless to slightly yellowish oils (∑ 888 mg, 1.81 mmol, 66%) were obtained. (*S*/*S*)-diastereomer: ^1H NMR (300 MHz, $CDCl_3$): δ/ppm = 9.11 (d, J = 1.5 Hz, 1H), 8.87 (d, J = 2.5 Hz, 1H), 8.76 (d, J = 8.4 Hz, 1H), 8.73 (dd, J = 2.5 Hz, J = 1.5 Hz, 1H), 8.57 (d, J = 7.9 Hz, 1H), 7.32–7.10 (m, 5H), 4.83 (td, J = 8.6, 5.0 Hz, 1H), 4.40 (ddd, J = 9.9, 7.7, 3.6 Hz, 1H), 3.65 (d, J = 11.2 Hz, 3H), 3.64 (d, J = 11.2 Hz, 3H), 3.27 (d, J = 21.4 Hz, 2H), 3.23–3.06 (m, 2H), 1.69–1.34 (m, 3H), 0.88 (d, J = 5.9 Hz, 3H), 0.83 (d, J = 5.9 Hz, 3H). ^{13}C NMR (75.5 MHz, $CDCl_3$): δ/ppm = 201.4 (d, J = 6.4 Hz), 170.7, 162.6, 147.8, 144.1, 143.5, 143.4, 137.4, 129.2, 128.2, 126.5, 57.2 (d, J = 3.6 Hz), 54.1, 52.61 and 52.58 (2 × d, J = 6.3 Hz), 37.7, 37.3, 36.3 (d, J = 129.7 Hz), 24.2, 23.1, 21.2. $[\alpha]_D^{22}$ = −27 (*c* 1.00, $CHCl_3$). FT-IR: ν/cm^{-1} = 3290, 2957, 1723, 1660, 1519, 1467, 1401, 1243, 1184, 1030, 1020, 815, 749. MS (ESI) *m*/*z* calculated for $[C_{23}H_{31}N_4O_6P+H]^+$ ($[M+H]^+$): 491.2, found: 491.3.

68, Ethyl (*S,E*)-7-methyl-4-oxo-5-((*S*)-3-phenyl-2-(pyrazine-2-carboxamido)propanamido)oct-2-enoate

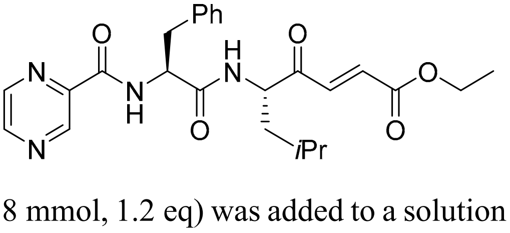

Anhydrous LiCl (25 mg, 0.58 mmol, 1.2 eq) was added to a solution of **67** (235 mg, 0.48 mmol, 1 eq) in dry MeCN (10 mL) under argon atmosphere at 0 °C. DIPEA (82 µL, 0.48 mmol, 1 eq) was added and the mixture was stirred at 0 °C for 15 min. Then, a solution of ethyl glyoxylate (freshly distilled over P_2O_5, 980 µL, 0.96 mmol, 2 eq) in dry MeCN (10 mL) was slowly added and stirred for one hour at 0 °C. The reaction was quenched by addition of a citric acid solution (10% in water, 2 mL), brine (15 mL) was added to the mixture and extracted with EtOAc (3x

50 mL). The combined organic extracts were dried over anhydrous Na_2SO_4, and the solvent was removed by distillation under reduced pressure. After purification by column chromatography ($CHCl_3$/MeOH 100:1), the product was obtained as a mixture of *E*/*Z*-isomers (*E*/*Z* = 5:1)1 (∑ 177 mg, 0.38 mmol, 79%). The corresponding isomers could be separated from each other by preparative HPLC. *E*-isomer: colorless oil, ^{1}H NMR (400 MHz, $CDCl_3$): δ/ppm = 9.34 (d, J = 1.5 Hz, 1H), 8.74 (d, J = 2.5 Hz, 1H), 8.52 (dd, J = 2.5 Hz, J = 1.5 Hz, 1H), 8.37 (d, J = 8.2 Hz, 1H), 7.27–7.17 (m, 5H), 7.13 (d, J = 15.8 Hz, 1H), 6.79 (d, J = 15.8 Hz, 1H), 6.65–6.56 (m, 1H), 4.98–4.88 (m, 1H), 4.85–4.75 (m, 1H), 4.27 (q, J = 7.1 Hz, 2H), 3.26–3.12 (m, 2H), 1.60–1.45 (m, 2H), 1.42–1.20 (m, 1H), 1.32 (t, J = 7.1 Hz, 3H), 0.88 (d, J = 6.2 Hz, 3H), 0.81 (d, J = 6.2 Hz, 3H). ^{13}C NMR (100.6 MHz, $CDCl_3$): δ/ppm =197.2, 169.7, 165.2, 163.1, 147.7, 144.5, 144.0, 142.9, 136.4, 136.3, 132.7, 129.4, 128.8, 127.3, 61.7, 56.1, 54.6, 40.3, 38.5, 25.0, 23.2, 21.9, 14.2. $[\alpha]_D^{22}$ = −27 (*c* 0.50, $CHCl_3$). FT-IR: ν/cm^{-1} = 2960, 2365, 2160, 1722, 1704, 1658, 1521, 1466, 1368, 1301, 1187, 1020, 875, 698. MS (ESI) *m*/*z* calculated for $[C_{25}H_{30}N_4O_5+H]^+$ ($[M+H]^+$): 467.2, found: 467.3. Purity: 99%.

69, Methyl (*tert*-butoxycarbonyl)-L-phenylalanyl-L-leucinate

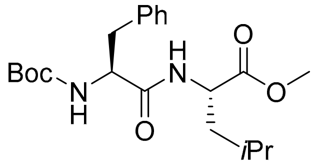

To a 0 °C cold solution of Boc-Phe-OH (1.34 mg, 5.04 mmol, 1 eq) in DCM (20 mL) were added HOBt · H_2O (0.77 g, 5.04 mmol, 1 eq) and 2,4,6-collidine (1.34 mL, 1.20 mmol, 2 eq). After stirring at 0 °C for 30 min, TBTU (1.62 g, 5.04 mmol, 1 eq) was added. The solution was stirred for further 30 min at 0 °C and H_2N-Leu-OMe · HCl **63** (0.92 g, 5.04 mmol, 1 eq) was added. After stirring at room temperature overnight, the solvent was removed under reduced pressure and the residue was taken up in EtOAc (100 mL). The mixture was washed with saturated $NaHCO_3$ solution (3x 80 mL) and hydrochloric acid (1 M, 3x 80 mL) and filtered over a small silica column. The filtrate was concentrated under reduced pressure to yield the desired product as a colorless solid (1.63 mg, 4.15 mmol, 82%). ^{1}H NMR (300 MHz $CDCl_3$): δ/ppm = 7.32–7.14 (m, 5H), 6.33 (d, J = 8.2 Hz, 1H), 5.12–4.95 (m, 1H), 4.62–4.48 (m, 1H), 4.42–4.27 (m, 1H), 3.68 (s, 3H), 3.06 (d, J = 6.8 Hz, 2H), 1.64 – 1.43 (m, 3H), 1.40 (s, 9H), 0.89 (t, J = 5.6 Hz, 6H). ^{13}C NMR (75.5 MHz, $CDCl_3$): δ/ppm = 173.0, 171.1, 155.5, 136.7, 129.5, 128.8, 127.1, 80.4, 55.9, 52.4, 50.9, 41.7, 38.2, 28.4, 24.8, 22.9, 22.0. mp: 98 – 100 °C. $[\alpha]_D^{22}$ = −14 (*c* 1.00, $CHCl_3$). FT-IR: ν/cm^{-1} = 3289, 3067, 2956, 2871, 1747, 1682, 1647, 1532, 1440, 1366, 1251, 1169, 1048, 1027, 702. MS (ESI) *m*/*z* calculated for $[C_{21}H_{32}N_2O_5+Na]^+$ ($[M+Na]^+$): 415.2, found: 415.1.

70, Methyl (pyrazine-2-carbonyl)-L-phenylalanyl-L-leucinate

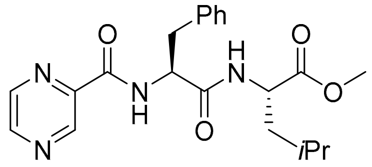

(1) **69** (1.44 g, 3.67 mmol) was treated with HCl in dioxane (4 M, 8 mL) at room temperature for 1.5 h. The solvent was removed under reduced pressure to yield the deprotected amine hydrochloride as a colorless foam (1.21 g, 3.67 mmol, quantitative), which was directly used in the next step.

(2) To a 0 °C cold solution of pyrazinecarboxylic acid (0.44 g, 3.55 mmol, 1 eq) in DCM (20 mL) were added HOBt · H_2O (0.54 g, 3.55 mmol, 1 eq) and 2,4,6-collidine (940 µL, 7.09 mmol, 2 eq). After stirring at 0 °C for 30 min, TBTU (1.14 g, 3.55 mmol, 1 eq) was added. The solution was stirred for further 30 min at 0 °C and the deprotected amine hydrochloride (1.17 g, 3.55 mmol, 1 eq) was added. After stirring at room temperature overnight, the solvent was removed under reduced pressure and the residue was taken up in EtOAc (70 mL). The mixture was washed with saturated $NaHCO_3$ solution (3x 50 mL) and hydrochloric acid (1 M, 3x 50 mL) and filtered over a small silica column. The filtrate was concentrated under reduced pressure to yield the desired product as a colorless solid (1.15 mg, 2.89 mmol, 82%). ^{1}H NMR (300 MHz, $CDCl_3$): δ/ppm = 9.35 (d, J = 1.5 Hz, 1H), 8.75 (d, J = 2.5 Hz, 1H), 8.55–8.51 (m, 1H), 8.39 (d, J = 8.3 Hz, 1H), 7.28–7.19 (m, 5H), 6.44 (d, J = 8.0 Hz, 1H), 4.98–4.88 (m, 1H), 4.61–4.50 (m, 1H), 3.71 (s, 3H), 3.21 (d, J = 7.0 Hz, 2H), 1.62–1.40 (m, 3H), 0.88–0.82 (m, 6H). ^{13}C NMR (75.5 MHz, $CDCl_3$): δ/ppm = 172.8, 170.2, 163.0, 147.5, 144.3, 144.1, 143.0, 136.4, 129.5, 128.8, 127.2, 54.6, 52.4, 51.1, 41.6, 38.5, 24.9, 22.7, 22.1. mp: 50–53 °C. $[\alpha]_D^{22} = -29$ (c 1.00, $CHCl_3$). FT-IR: ν/cm^{-1} = 3294, 3063, 3030, 2955, 2870, 1742, 1651, 1519, 1438, 1370, 1200, 1152, 1019, 746, 699. MS (ESI) m/z calculated for $[C_{21}H_{26}N_4O_4+Na]^+$ ($[M+Na]^+$): 421.2, found: 421.1.

71, (Pyrazine-2-carbonyl)-L-phenylalanyl-L-leucine

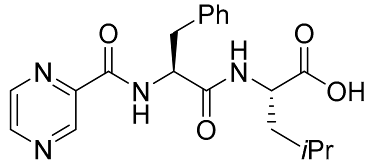

To a solution of **70** (1.09 g, 2.72 mmol, 1 eq) in THF (15 mL) and water (15 mL) was added LiOH (0.34 g, 8.17 mmol, 3 eq). After stirring at room temperature for 17 h, THF was removed by distillation under reduced pressure and the aqueous solution was acidified to pH = 1 with hydrochloric acid (1 M). The resulting suspension was extracted with $CHCl_3$, and the combined organic extracts were dried over anhydrous Na_2SO_4. After removing the solvent by distillation under reduced pressure, the title compound was obtained as a colorless foam (1.05 g, 2.72 mmol, quantitative). ^{1}H NMR (300 MHz, $CDCl_3$): δ/ppm = 9.31 (d, J = 1.4 Hz, 1H), 8.74 (d, J = 2.5 Hz, 1H), 8.56–8.54 (m, 1H), 8.51 (d, J = 8.5 Hz, 1H), 7.25–7.07 (m, 6H), 6.98 (d, J = 8.0 Hz, 1H), 5.10–5.00 (m, 1H), 4.64–4.53 (m, 1H), 3.29–3.13 (m, 2H), 1.71–1.47 (m, 3H),

0.88–0.82 (m, 6H). ^{13}C NMR (75.5 MHz, $CDCl_3$): δ/ppm = 175.5, 170.8, 163.1, 147.2, 144.3, 144.0, 143.3, 136.3, 129.5, 128.7, 127.2, 54.6, 51.2, 41.3, 38.6, 24.9, 22.8, 22.0. mp: 62 – 64 °C. $[\alpha]_D^{22}$ = −3 (*c* 1.00, $CHCl_3$). FT-IR: ν/cm^{-1} = 3297, 3064, 3031, 2956, 2870, 1724, 1648, 1522, 1467, 1199, 1151, 1020, 866, 746, 698. MS (ESI) *m*/*z* calculated for $[C_{20}H_{24}N_4O_4+H]^+$ ($[M+H]^+$): 385.2, found: 385.1.

72, *N*-((*S*)-1-(((*S*)-4-Methyl-1-oxo-1-(((*S*)-2-oxo-1-phenylazetidin-3-yl)amino)pentan-2-yl)amino)-1-oxo-3-phenylpropan-2-yl)pyrazine-2-carboxamide

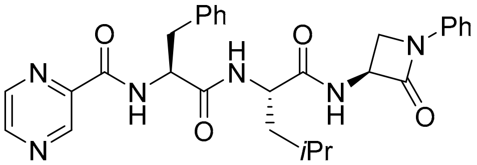

To a 0 °C cold solution of **72** (131 mg, 0.34 mmol, 1 eq), HOBt · H_2O (52 mg, 0.34 mmol, 1 eq), and TBTU (109 mg, 0.34 mmol, 1 eq) in DCM (20 mL) were added **9** (68 mg, 0.34 mmol, 1 eq) and 2,4,6-collidine (90 µL, 0.68 mmol, 2 eq) and the mixture was stirred at room temperature overnight. The solvent was removed under reduced pressure and the residue was purified by column chromatography (DCM/MeOH 30:1) to yield the desired product as a colorless solid (117 mg, 0.22 mmol, 65%). ^{1}H NMR (300 MHz, $CDCl_3$): δ/ppm = 9.27 (d, *J* = 1.4 Hz, 1H), 8.69 (d, *J* = 2.5 Hz, 1H), 8.46–8.40 (m, 1H), 8.35 (d, *J* = 7.5 Hz, 1H), 7.51 (d, *J* = 7.5 Hz, 1H), 7.33–7.27 (m, 4H), 7.25–7.13 (m, 5H), 7.12–7.05 (m, 1H), 7.00 (d, *J* = 8.1 Hz, 1H), 4.97–4.85 (m, 2H), 4.55–4.45 (m, 1H), 3.89–3.82 (m, 1H), 3.59 (dd, *J* = 5.8, 2.7 Hz, 1H), 3.24–3.18 (m, 2H), 1.73–1.43 (m, 3H), 0.84–0.76 (m, 6H). ^{13}C NMR (75.5 MHz, $CDCl_3$): δ/ppm = 172.4, 170.9, 163.8, 163.6, 147.7, 144.3, 143.8, 142.9, 137.8, 136.2, 129.4, 129.3, 128.9, 127.3, 124.5, 116.8, 55.62, 55.0, 52.0, 47.1, 40.5, 38.0, 24.8, 23.0, 21.9. mp: 187–189 °C. $[\alpha]_D^{22}$ = −15 (*c* 1.00, $CHCl_3$). FT-IR: ν/cm^{-1} = 3390, 3280, 3066, 2957, 2870, 1757, 1653, 1598, 1498, 1464, 1387, 1147, 1020, 750, 693. MS (ESI) *m*/*z* calculated for $[C_{29}H_{32}N_6O_4+H]^+$ ($[M+H]^+$): 529.3, found: 529.2. Purity: 95%.

73, *N*-((*S*)-1-(((*S*)-1-Amino-4-methyl-1-oxopentan-2-yl)amino)-1-oxo-3-phenylpropan-2-yl)pyrazine-2-carboxamide

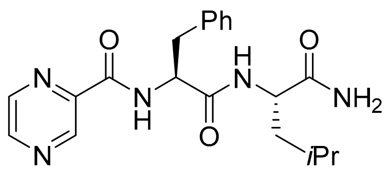

To a 0 °C cold solution of **70** (365 mg, 0.92 mmol) in MeOH (5 mL) was added NH_3 solution (7 N in MeOH, 12 mL). The solution was stirred at 0 °C for 15 min, and at room temperature for 24 h. Again, NH_3 solution (7 N in MeOH, 12 mL) was added, and the mixture was stirred at room temperature for an additional 24 h. The solvent was removed by distillation to yield the title compound as a colorless solid (315 mg, 0.82 mmol, 89%). ^{1}H NMR (300 MHz, $CDCl_3$): δ/ppm = 9.16 – 9.07 (m, 1H), 8.87 (d, *J* = 2.6 Hz, 1H), 8.77–8.62 (m, 2H), 8.27 (d, *J* = 8.3 Hz, 1H), 7.37–7.29 (m, 1H), 7.28–7.09 (m, 5H), 7.07–6.93 (m, 1H), 4.89–4.76 (m, 1H), 4.36–4.24 (m, 1H), 3.23–3.02 (m, 2H), 1.67–1.40 (m, 3H), 0.92–0.78 (m, 6H). ^{13}C NMR (75.5 MHz,

CDCl3): δ/ppm = 173.9, 170.0, 162.3, 147.8, 144.1, 143.4, 137.3, 129.3, 128.1, 126.4, 53.9, 50.9, 41.1, 37.5, 24.3, 23.0, 21.7. mp: 201–203 °C. $[\alpha]_D^{22}$ = −4 (*c* 1.00, $CHCl_3$). FT-IR: *ν*/cm^{-1} = 3368, 3308, 3195, 3054, 2959, 1686, 1645, 1530, 1449, 1398, 1227, 1020, 774, 746, 693. MS (ESI) *m*/*z* calculated for $[C_{20}H_{25}N_5O_3+Na]^+$ ($[M+Na]^+$): 406.2, found: 406.1.

74, *N*-((*S*)-1-(((*S*)-1-Cyano-3-methylbutyl)amino)-1-oxo-3-phenylpropan-2-yl)pyrazine-2-carboxamide

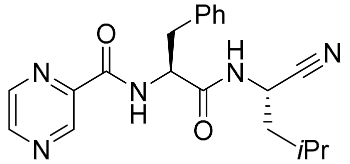

To a 0 °C cold solution of **73** (211 mg, 0.55 mmol, 1 eq) in dry DMF (4 mL) was added cyanuric chloride (112 mg, 0.61 mmol, 1.1 eq) in portions. The solution was stirred at 0 °C for one hour and at room temperature overnight. Again, cyanuric chloride (112 mg, 0.61 mmol, 1.1 eq) was added, and the mixture was stirred at room temperature for an additional 24 h. The solvent was removed by distillation under reduced pressure and the residue was purified by reversed phase flash chromatography (H_2O/MeCN, gradient) yield the desired product as a slightly yellowish solid (95 mg, 0.26 mmol, 47%). ^1H NMR (300 MHz, $CDCl_3$): δ/ppm = 9.33 (d, *J* = 1.5 Hz, 1H), 8.78 (d, *J* = 2.5 Hz, 1H), 8.55 (dd, *J* = 2.5, 1.5 Hz, 1H), 8.38 (d, *J* = 8.1 Hz, 1H), 7.34–7.19 (m, 5H), 6.54 (d, *J* = 8.2 Hz, 1H), 4.86–4.74 (m, 2H), 3.22 (d, *J* = 7. 1 Hz, 2H), 1.77–1.45 (m, 3H), 0.93–0.85 (m, 6H). ^{13}C NMR (75.5 MHz, $CDCl_3$): δ/ppm = 170.1, 163.4, 147.9, 144.4, 143.7, 143.0, 135.9, 129.4, 129.1, 127.5, 118.2, 54.9, 41.7, 39.2, 38.4, 24.9, 22.3, 21.8. mp: 46 °C (decomposition). $[\alpha]_D^{22}$ = −32 (*c* 1.00, $CHCl_3$). FT-IR: *ν*/cm^{-1} = 3288, 3061, 2959, 2872, 2247, 1657, 1519, 1467, 1402, 1236, 1154, 1020, 910, 729, 700. MS (ESI) *m*/*z* calculated for $[C_{20}H_{23}N_5O_2+H]^+$ ($[M+H]^+$): 366.2, found: 366.0. Purity: 95%.

Synthesis of SARS-Cov-2 M^{pro} targeting compounds

76, Benzyl (4-methoxy-1*H*-indole-2-carbonyl)-L-leucinate

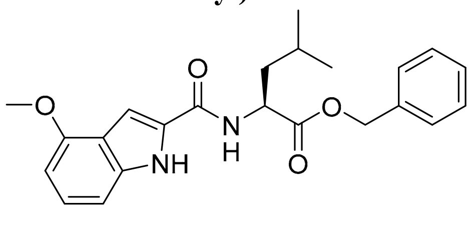

4-methoxy-1H-indole-2-carboxylic acid **75** (500 mg, 2.62 mmol, 1 eq) was dissolved in DCM (20 ml). DIPEA (1.3 ml, 7.85 mmol, 3 eq) and TBTU (840 mg, 2.62 mmol, 1 eq) were added and the mixture was stirred for 15 min. H-Leu-OBzl · *p*-TsOH (1.029 g, 2,62 mmol, 1eq) was added and the mixture was stirred for 24 h. The solvent was evaporated under reduced pressure and the residue was dissolved in EtOAc (30 ml) and washed with hydrochloric acid (2x, 1 M, 20 ml), saturated $NaHCO_3$ solution (2x, 20 ml) and brine (2x, 20 ml). The combined organic layers were dried over anhydrous sodium sulfate and the solvent was evaporated under reduced pressure. The resulting residue was purified by chromatography (CH/EA, gradient) yielding the desired product as a colorless solid (963 mg, 2.44 mmol, 93%). ^{1}H NMR (300 MHz, $CDCl_3$): δ/ppm = 9.75 (s), 7.39 – 7.30 (m, 5H), 7.22 – 7.15 (m, 1H), 7.08 – 7.07 (m, 1H), 7.12 – 7.00 (m, 1H), 6.73 (d, *J* = 8.4 Hz, 1H), 6.49 (d, *J* = 8.4 Hz, 1H), 5.22 (s, 2H), 5.01 – 4.87 (m, 1H), 3.95 (s, 3H), 1.84 – 1.65 (m, 3H), 0.97 (dd, *J* = 6.2, 4.3 Hz, 6H). 13C NMR (75 MHz, $CDCl_3$): δ/ppm = 173.02, 161.59, 154.31, 138.10, 135.44, 128.90, 128.75, 128.56, 128.38, 125.70, 119.00, 105.32, 100.68, 99.75, 67.36, 55.41, 51.19, 41.83, 25.10, 22.97, 22.15. $[\alpha]_D^{22}$ = +28 (*c* 1.00, $CHCl_3$). mp: 104 – 106 °C. FT-IR: ν/cm^{-1} = 3274, 2957, 1729, 1636, 1583, 1541, 1515, 1429, 1360, 1253, 1191, 1133, 1100, 977, 824, 753, 696. MS (ESI) *m/z* calculated for $[C_{23}H_{27}N_2O_4]^+$ ($[M+H]^+$): 395.2, found: 395.4.

77, (4-Methoxy-1*H*-indole-2-carbonyl)-L-leucine

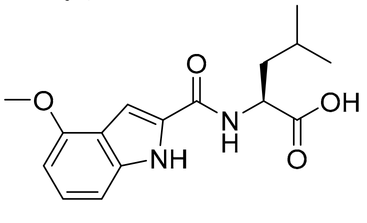

Into a solution of **76** (750 mg, 1,90 mmol, 1 eq) in ethanol Pd/C (10%, 15 mg) was suspended and the mixture was stirred for 18 h under a hydrogen atmosphere. After filtration over Celite® 545 the solvent was evaporated under reduced pressure yielding the desired product as a colorless solid (552 mg, 1.81 mmol, 95%). ^{1}H NMR (300 MHz, DMSO-d_6): δ/ppm = 11.55 (s, 1H), 8.50 (d, *J* = 8.0 Hz, 1H), 7.39 – 7.32 (m, 1H), 7.16 – 6.98 (m, 2H), 6.50 (d, *J* = 7.6 Hz, 1H), 4.55 – 4.39 (m, 1H), 3.88 (s, 3H), 1.88 – 1.66 (m, 2H), 1.67 – 1.51 (m, 1H), 0.91 (dd, *J* = 13.5, 6.2 Hz, 3H). ^{13}C NMR (75 MHz, DMSO-d_6): δ/ppm = 173.02, 161.59, 154.31, 138.10, 135.44, 128.90, 128.75, 128.56, 128.38, 125.70, 119.00, 105.32, 100.68, 99.75, 67.36, 55.41, 51.19, 41.83, 25.10, 22.97, 22.15. $[\alpha]_D^{22}$ = +7 (*c* 1.00, MeOH). mp: 191 – 193 °C. FT-IR: ν/cm^{-1} = 3276, 2957, 1715, 1620, 1584, 1542, 1516, 1429, 1359, 1307, 1253, 1165, 1132, 1099, 977, 822, 754, 705. MS (ESI) *m/z* calculated for $[C_{16}H_{21}N_2O_4]^+$ ($[M+H]^+$): 305.2, found: 305.0.

79, *Bis*-(*tert*-butyloxycarbonyl)-((*S*)-4-(dimethoxyphosphoryl)-3-oxo-1-((*S*)-2-oxopyrrolidin-3-yl)butan-2-yl)amine

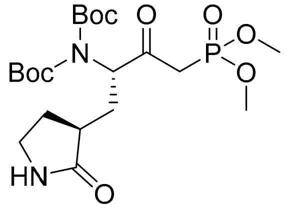

A solution of dimethyl methylphosphonate (2.035 ml, 19.03 mmol, 6 eq) in dry THF (15 ml) was cooled to −78 °C while stirring. *n*-BuLi (2.5 M in hexanes, 6.34 ml, 15.85 mmol, 5 eq) was added and stirring was continued at −78 °C for 30 min. **78**[25] (1 g, 3.17 mmol, 1 eq) was added and the mixture was stirred for six hours at −78 °C. Hydrochloric acid (20 ml, 1 M) and saturated ammonium chloride solution (30 ml) were added and the mixture was allowed to warm up to room temperature over 30 min while stirring. After Extraction with a mixture of chloroform and isopropyl alcohol (3:1, 4x 20 ml) the combined organic layers were dried over sodium sulfate and the solvent was removed under reduced pressure. The residue was purified by reversed phase flash chromatography (H_2O/MeCN, gradient) yielding the desired product as a colorless resin (357 mg, 0.78 mmol, 25%). ^{1}H NMR (300 MHz, $CDCl_3$): δ/ppm = 6.65 (s, 1H), 4.82 (dd, J = 8.8, 5.0 Hz, 1H), 3.75 (dd, J = 11.2, 3.2 Hz, 7H), 3.45 – 3.21 (m, 3H), 3.07 – 2.82 (m, 1H), 2.43 – 2.26 (m, 2H), 2.24 – 2.04 (m, 2H), 1.84 – 1.71 (m, 1H), 1.47 (s, 18H). ^{13}C NMR (75 MHz, $CDCl_3$): δ/ppm = 198.20, 198.11, 179.81, 152.12, 84.22, 63.19, 63.16, 53.19, 53.11, 53.03, 40.48, 38.34, 37.45, 35.71, 30.19, 28.02, 27.97. FT-IR: ν/cm^{-1} = 3277, 2976, 1693, 1522, 1458, 1392, 1367, 1251, 1167, 1030, 868, 811. MS (ESI) *m/z* calculated for $[C_{20}H_{35}N_2O_9PNa]^+$ ($[M+Na]^+$): 501.2, found: 501.1.

80, Dimethyl ((*S*)-3-((*S*)-2-(4-methoxy-1*H*-indole-2-carboxamido)-4-methylpentanamido)-2-oxo-4-((*S*)-2-oxopyrrolidin-3-yl)butyl)phosphonate

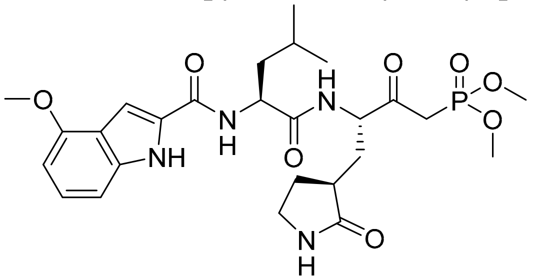

79 (233 mg, 0.62 mmol, 1 eq) was dissolved in DCM (8 ml) and cooled to 0 °C. TFA (2 ml) was added dropwise to the solution while stirring. After stirring for one hour at 0 °C the solvent was removed under reduced pressure and the residue was taken up in a mixture of acetonitrile and water (1:1, 10 ml) and lyophilized overnight. The resulting residue was taken up in dry THF (5 ml) and cooled to −20 °C (amine solution). A solution of **77** (234 mg, 0.77 mmol, 1.25 eq) in dry THF (10 ml) was cooled to −20 °C. NMM (205 µL, 1.85 mmol, 3 eq) was added to the mixture followed by the addition of ethyl chloroformate (70 µL, 0.74 mmol, 1.2 eq) while stirring at −20 °C. After stirring for 15 min at −20 °C the amine solution was added and the mixture was stirred at −20 °C for 15 min. The reaction was quenched by the addition of acetic acid (70 µL, 1.23 mmol, 2 eq) and allowed to warm up to room temperature while stirring for 30 min. The solvent was removed under reduced pressure and the residue was purified by reversed phase flash chromatography (H_2O/MeCN, gradient). Additional purification using preparative HPLC yielded the desired product as a colorless solid (140 mg, 0.25 mmol, 40%).

^{1}H NMR (600 MHz, $CDCl_3$): δ/ppm = 10.12 (s, 1H), 8.61 (s, 1H), 7.37 (s, 1H), 7.23 – 7.10 (m, 2H), 7.03 (d, J = 8.2 Hz, 1H), 6.58 (s, 1H), 6.46 (d, J = 7.7 Hz, 1H), 4.73 (s, 1H), 4.61 – 4.46 (m, 1H), 3.92 (s, 3H), 3.72 (dd, J = 11.2, 8.4 Hz, 7H), 3.53 – 3.43 (m, 1H), 3.21 – 3.11 (m, 2H), 3.03 (s, 1H), 2.32 (s, 1H), 2.19 – 2.12 (m, 1H), 2.04 – 1.93 (m, 1H), 1.92 – 1.69 (m, 6H), 1.64 (s, 1H), 0.96 (dd, J = 8.7, 5.6 Hz, 6H). ^{13}C NMR (151 MHz, $CDCl_3$): δ/ppm = 201.47, 180.41, 173.93, 173.60, 162.04, 154.22, 138.14, 129.27, 125.62, 118.83, 105.37, 101.75, 99.61, 58.25, 55.40, 53.40, 53.35, 53.31, 53.27, 52.63, 41.30, 40.80, 38.22, 37.85, 36.98, 31.52, 28.26, 25.14, 23.23, 21.87. $[\alpha]_D^{22}$ = +22 (c 1.00, $CHCl_3$). mp: 134 °C decomposition. FT-IR: ν/cm^{-1} = 3268, 2956, 1722, 1678, 1641, 1548, 1514, 1502, 1462, 1432, 1367, 1254, 1100, 1033, 765. MS (ESI) m/z calculated for $[C_{26}H_{38}N_4O_8P]^+$ ($[M+H]^+$): 565.2, found: 565.3.

81, Ethyl (*S,E*)-5-((*S*)-2-(4-methoxy-1*H*-indole-2-carboxamido)-4-methylpentanamido)-4-oxo-6-((S)-2-oxopyrrolidin-3-yl)hex-2-enoate

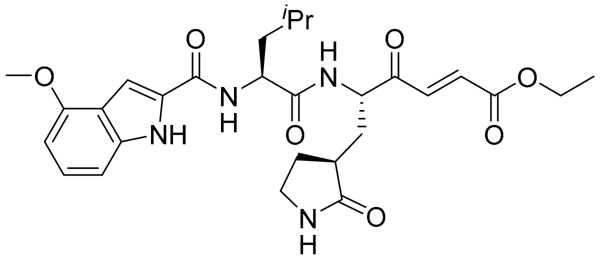

A solution of **80** (140 mg, 0.25 mmol, 1 eq) and LiCl (21 mg, 0.50 mmol, 2 eq) in dry MeCN (8 ml) was cooled to 0 °C. DIPEA (42 µL, 0.25 mmol, 1 eq) was added, followed by the dropwise addition of a solution of freshly distilled ethyl glyoxylate (51 µL, 0.5 mmol, 2 eq) in dry MeCN (2 ml), while stirring at 0 °C. After stirring for two hours at 0 °C, hydrochloric acid (0.1 M, 5 ml) and saturated NH_4Cl solution (20 ml) were added and stirring was continued at room temperature for 15 min, followed by extraction with a mixture of chloroform an isopropyl alcohol (3:1, 4x 15ml). The combined organic layers were dried over $NaSO_4$ and the solvent was removed under reduced pressure. The resulting residue was purified by preparative HPLC yielding the desired product as a yellow solid (64 mg, 0.12 mmol, 47%). ^{1}H NMR (300 MHz, $CDCl_3$): δ/ppm = 10.98 (s, 1H), 9.98 (s, 1H), 8.59 (d, J = 6.3 Hz, 1H), 7.27 (d, J = 15.8 Hz, 1H), 7.21 – 7.07 (m, 2H), 7.01 (d, J = 8.3 Hz, 1H), 6.80 (d, J = 15.8 Hz, 1H), 6.47 (d, J = 7.7 Hz, 1H), 4.91 – 4.76 (m, 1H), 4.73 – 4.61 (m, 1H), 4.32 – 4.17 (m, 2H), 3.91 (s, 3H), 3.32 – 3.07 (m, 2H), 2.68 – 2.20 (m, 2H), 2.15 – 1.87 (m, 1H), 1.86 – 1.64 (m, 5H), 1.30 (t, J = 7.1 Hz, 3H), 1.07 – 0.87 (m, 6H). ^{13}C NMR (75 MHz, DMSO): δ/ppm = 197.53, 178.33, 172.79, 164.74, 160.98, 153.60, 137.78, 137.13, 130.29, 129.79, 124.30, 118.05, 105.34, 101.16, 99.11, 60.86, 55.16, 54.99, 51.37, 40.22, 39.52, 37.31, 30.36, 27.25, 24.33, 22.83, 21.45, 13.91. $[\alpha]_D^{22}$ = +35 (c 1.00, $CHCl_3$). mp: 132 – 134 °C. FT-IR: ν/cm^{-1} = 3268, 2956, 1723, 1703, 1691, 1679, 1659, 1649, 1640, 1630, 1583, 1563, 1547, 1536, 1514, 1502, 1432, 1367, 1301, 1256, 1099, 760. MS (ESI) m/z calculated for $[C_{28}H_{37}N_4O_7]^+$ ($[M+H]^+$): 541.3, found: 541.4. Purity: 99%.

82, Methyl (S)-2-((S)-2-(4-methoxy-1H-indole-2-carboxamido)-4-methylpentanamido)-3-((S)-2-oxopyrrolidin-3-yl)propanoate

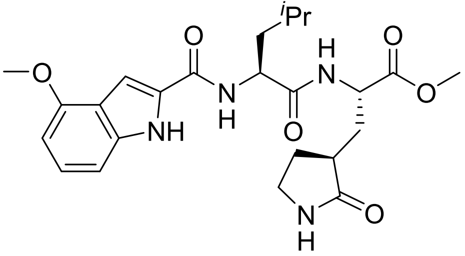

78[25] (300 mg, 0.78 mmol, 1 eq) was dissolved in DCM (8 ml) and cooled to 0 °C. TFA (2 ml) was added dropwise to the solution while stirring. After stirring for one hour at 0 °C the solvent was removed under reduced pressure and the residue was taken up in a mixture of acetonitrile and water (1:1, 10 ml) and lyophilized overnight. The resulting residue was taken up in DMF (5 ml) (amine solution). A solution of **77** (285 mg, 0,93 mmol, 1.2 eq) in DMF (10 ml) was cooled to 0 °C. 2,4,6-collidine (308 µL, 2.33 mmol, 3 eq) and HATU (354 mg, 0.93 mmol, 1.2 eq) were added. After stirring for 20 min at 0 °C the amine solution was added and stirring was continued over night at room temperature. The solvent was removed under reduced pressure and the residue was purified by reversed phase flash chromatography (H_2O/MeCN, gradient) yielding the desired product as a colorless solid (303 mg, 0.64 mmol, 83%). ^{1}H NMR (300 MHz, $CDCl_3$): δ/ppm = 10.13 (s, 1H), 8.29 (d, J = 7.0 Hz, 1H), 7.19 – 7.10 (m, 2H), 7.00 (d, J = 8.3 Hz, 1H), 6.81 (s, 1H), 6.46 (d, J = 7.7 Hz, 1H), 5.01 – 4.84 (m, 1H), 4.61 – 4.44 (m, 1H), 3.91 (s, 3H), 3.72 (s, 3H), 3.28 – 3.03 (m, 2H), 2.43 (s, 1H), 2.33 – 2.18 (m, 2H), 1.93 – 1.61 (m, 6H), 1.02 – 0.91 (m, 6H). ^{13}C NMR (75 MHz, $CDCl_3$): δ/ppm = 180.02, 172.88, 172.32, 161.67, 154.16, 138.09, 128.97, 125.45, 118.84, 105.22, 101.16, 99.58, 55.30, 52.42, 51.78, 51.43, 42.00, 40.54, 38.41, 32.81, 28.06, 24.80, 22.83, 22.33. $[\alpha]_D^{22}$ = +25 (c 1.00, $CHCl_3$). mp: 131 – 133 °C. FT-IR: ν/cm^{-1} = 3267, 2953, 1738, 1666, 1630, 1582, 1562, 1547, 1514, 1502, 1462, 1432, 1366, 1253, 1211, 1132, 1099, 828, 755, 666. MS (ESI) m/z calculated for $[C_{24}H_{33}N_4O_6]^+$ ($[M+H]^+$): 473.2, found: 473.3.

83, *N*-((*S*)-1-(((*S*)-1-Amino-1-oxo-3-((*S*)-2-oxopyrrolidin-3-yl)propan-2-yl)amino)-4-methyl-1-oxopentan-2-yl)-4-methoxy-1*H*-indole-2-carboxamide

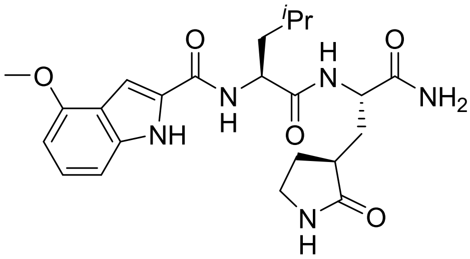

To a cooled solution of **82** (125 mg, 0.26 mmol, 1 eq) in THF (8 ml) was added a solution of LiOH (19 mg, 0.79 mmol, 3 eq) in water (2 ml) while stirring at 0 °C. After stirring over night at 4 °C hydrochloric acid (0.1 M, 10 ml) followed by extraction with a mixture of chloroform and isopropyl alcohol (3:1, 4x 10 ml). The combined organic layers were dried over anhydrous sodium sulfate and the solvents were removed under reduced pressure. The residue was taken up in DMF (8 ml) and the solution was cooled to 0°C. While stirring 2,4,6-collidine (105 µL, 0.79 mmol, 3 eq), OxymaPure® (38 mg, 026 mmol, 1 eq) and HATU (151 mg, 0.40 mmol, 1.5 eq) were added and the mixture was kept stirring at 0 °C for one hour. Ammonia solution (25%, 100 µL) was added and the mixture was at room temperature over night. The solvent was removed under reduced pressure and the residue was purified by reversed phase flash

chromatography (H_2O/MeCN, gradient). Additional purification using preparative HPLC yielded the desired product as a colorless solid (77 mg, 0.17 mmol, 65%). ^{1}H NMR (300 MHz, DMSO-d_6): δ/ppm = 11.57 (d, J = 2.3 Hz, 1H), 8.41 (d, J = 7.9 Hz, 1H), 8.03 (d, J = 8.4 Hz, 1H), 7.59 (s, 1H), 7.35 (dd, J = 2.3, 0.8 Hz, 1H), 7.31 – 7.27 (m, 1H), 7.14 – 6.98 (m, 3H), 6.51 (dd, J = 7.7, 0.8 Hz, 1H), 4.56 – 4.38 (m, 1H), 4.39 – 4.19 (m, 1H), 3.88 (s, 3H), 3.15 – 2.98 (m, 2H), 2.33 – 2.20 (m, 1H), 2.20 – 2.10 (m, 1H), 2.08 – 1.96 (m, 1H), 1.81 – 1.44 (m, 5H), 0.91 (dd, J = 13.3, 6.2 Hz, 6H). ^{13}C NMR (75 MHz, DMSO-d_6): δ/ppm = 178.39, 173.42, 172.17, 161.12, 153.63, 137.84, 129.87, 124.44, 118.06, 105.40, 101.13, 99.24, 55.07, 51.74, 50.70, 40.19, 39.43, 37.69, 33.74, 27.41, 24.44, 23.10, 21.36. $[\alpha]_D^{22}$ = +16 (*c* 1.00, MeOH). mp: 109 – 111 °C. FT-IR: ν/cm^{-1} = 3267, 2955, 1673, 1632, 1582, 1548, 1515, 1462, 1429, 1366, 1302, 1255, 1100, 1026, 764. MS (ESI) *m/z* calculated for $[C_{28}H_{32}N_5O_5]^+$ ($[M+H]^+$): 458.2, found: 458.1.

84, *N*-((*S*)-1-(((*S*)-1-Cyano-2-((*S*)-2-oxopyrrolidin-3-yl)ethyl)amino)-4-methyl-1-oxopentan-2-yl)-4-methoxy-1*H*-indole-2-carboxamide

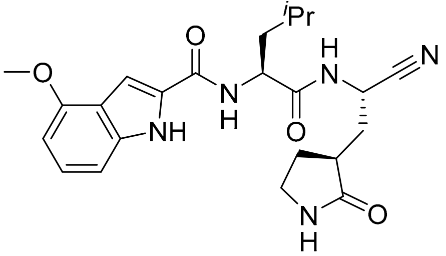

To a stirred solution of **83** (60 mg, 0.13 mmol, 1 eq) in DCM was added Burgess reagent (63 mg, 0.26 mmol, 2 eq) and stirring was continued for two hours at room temperature. The solvent was removed under reduced pressure and the residue was purified by preparative HPLC yielding the desired product as a colorless solid (38 mg, 0.09 mmol, 67%). ^{1}H NMR (400 MHz, DMSO-d_6): δ/ppm = 11.58 (d, J = 2.4 Hz, 1H), 8.91 (d, J = 8.0 Hz, 1H), 8.47 (d, J = 7.7 Hz, 1H), 7.71 (s, 1H), 7.37 (d, J = 2.2 Hz, 1H), 7.09 (t, J = 7.9 Hz, 1H), 7.00 (d, J = 8.2 Hz, 1H), 6.50 (d, J = 7.6 Hz, 1H), 4.97 (dt, J = 9.4, 7.5 Hz, 1H), 4.50 – 4.39 (m, 1H), 3.88 (s, 3H), 3.21 – 3.04 (m, 2H), 2.44 – 2.28 (m, 1H), 2.22 – 2.05 (m, 2H), 1.86 – 1.77 (m, 1H), 1.77 – 1.66 (m, 2H), 1.57 – 1.48 (m, 1H), 0.94 (d, J = 6.1 Hz, 3H), 0.89 (d, J = 6.2 Hz, 3H). ^{13}C NMR (101 MHz, DMSO-d_6): δ/ppm = 177.55, 177.47, 172.52, 161.19, 153.66, 137.84, 129.75, 124.49, 119.67, 118.07, 105.41, 101.31, 99.23, 55.09, 51.40, 40.40, 39.80, 38.30, 37.12, 33.44, 27.00, 24.42, 23.02, 21.34. $[\alpha]_D^{22}$ = +13 (*c* 1.00, MeOH). mp: 102 – 104 °C. FT-IR: ν/cm^{-1} = 3257, 2960, 1680, 1642, 1583, 1544, 1514, 1461, 1429, 1357, 1300, 1255, 1166, 1131, 1099, 1046, 1025, 976, 832, 762, 713, 683. MS (ESI) *m/z* calculated for $[C_{23}H_{30}N_5O_4]^+$ ($[M+H]^+$): 440.23, found: 440.51. Purity: 99%.

87, *tert*-Butyl ((*S,E*)-4-nitro-1-((*S*)-2-oxopyrrolidin-3-yl)but-3-en-2-yl)carbamate

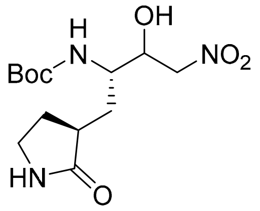

86 (1.26 g, 4.00 mmol, 1 eq) was dissolved in dry THF (30 ml) and cooled to −20 °C followed by the addition of $LiAlH_4$ (182.16 mg, 4.80 mmol, 1.2 eq). The mixture was stirred at −20 °C for one hour after which the reaction was quenched by the addition of hydrochloric acid (1 M, 5 ml). The pH of the mixture was adjusted to neutral using hydrochloric acid (1 M) and saturated $NaHCO_3$ solution, while stirring at room temperature. The neutralized solution was extracted with a mixture of chloroform and isopropyl alcohol (3:1, 4x 20 ml) and the combined organic layers were dried over anhydrous Na_2SO_4 and the solvent was removed under reduced pressure. The resulting residue contained the respective aldehyde and was taken up in DCM (30 ml). The solution was cooled to 0 °C and Et_3N (0.17 ml, 1.21 mmol, 0.3 eq) followed by nitromethane (1.29 ml, 24.00 mmol, 6 eq) was added. After stirring for 15 h at room temperature, saturated NH_4Cl solution (10 ml) was added. The mixture was extracted with DCM (3x 15 ml) and the combined organic layers were washed with hydrochloric acid (1 M, 1x 15 ml) and brine (1x 15 ml) and then dried over Na_2SO_4. The solvent was removed under reduced pressure and the resulting residue was purified by column chromatography (DCM/MeOH 99:1 to 85:15) to yield the desired product as a yellow oil (901 mg, 2.84 mmol, 71%). ^{1}H NMR (400 MHz, CD_3OD): δ/ppm = 4.67–4.53 (m, 1H), 4.43–4.28 (m, 2H), 3.85–3.76 (m, 1H), 2.50 (ddd, J = 18.2, 8.6, 4.8 Hz, 1H), 2.34 (tdd, J = 12.6, 5.7, 3.9 Hz, 1H), 2.09 (dt, J = 14.1, 5.2 Hz, 1H), 1.91 (dq, J = 12.5, 9.0 Hz, 1H), 1.66–1.53 (m, 1H), 1.47 (s, 9H). ^{13}C NMR (101 MHz, CD_3OD): δ/ppm = 180.8, 156.5, 79.1, 78.7, 69.7, 51.6, 40.1, 39.0, 32.1, 27.4, 27.3. MS (ESI) m/z calculated for $[C_{13}H_{22}N_3O_6Na]^+$ ($[M+Na]^+$): 340.2 found: 340.2.

88, 4-Methoxy-*N*-((*S*)-4-methyl-1-(((*S,E*)-4-nitro-1-((*S*)-2-oxopyrrolidin-3-yl)but-3-en-2-yl)amino)-1-oxopentan-2-yl)-1*H*-indole-2-carboxamide

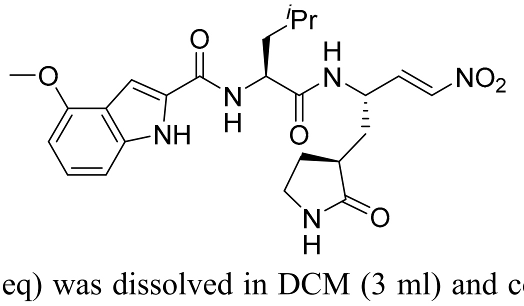

87 (231 mg, 0.73 mmol, 1 eq) was dissolved in DCM (3 ml) and cooled to 0 °C. TFA (1 ml) was added dropwise ant the mixture was stirred at room temperature for three hours. The solvent was removed under reduced pressure and the resulting residue was taken up in DCM (8 ml). **77** (268 mg, 0.88 mmol, 1.2 eq) and HOBt · H_2O (121 mg, 0.89 mmol, 1.2 eq) were added and the mixture was cooled to 0 °C. DIPEA (0.56 ml, 3.23 mmol, 4.4 eq) and EDC · HCl (186 mg, 0.97 mmol, 1.3 eq) were added an d the mixture war stirred at room temperature for 16 hours. Saturated NH_4Cl solution (10 ml) was added and the mixture was extracted with DCM (3x 20 ml). The combined organic layers were washed with hydrochloric acid (1 M, 2x 20 ml) and saturated $NaHCO_3$ solution (2x 20 ml) and dried over Na_2SO_4. The solvent was removed under reduced pressure and the residue was purified by column chromatography (DCM/MeOH 100:0 to 85:15). The resulting dipeptidyl nitroaldol product (332 mg, 0.66 mmol) was dissolved in DCM (7 ml) and cooled to 0 °C. DIPEA (0.24 ml, 1.39 mmol, 2.0 eq) followed

by MsCl (56 µL, 0.73 mmol, 1 eq) were added and the mixture was stirred overnight at room temperature. The addition of saturated NH_4Cl solution (10 ml) was followed by extraction with DCM (3x 20 ml). The combined organic layers were washed with hydrochloric acid (1 M, 2x 20 ml) and saturated $NaHCO_3$ solution (2x 20 ml) and dried over Na_2SO_4. The solvent was removed under reduced pressure and the resulting residue was purified by column chromatography (DCM/MeOH 99:1 to 9:1) to afford the desired product as a colorless solid (177 mg, 0.37 mmol, 50%). ^1H NMR (400 MHz, CD_3OD): δ/ppm = 7.03–7.19 (m, 4H), 6.93 (d, J = 8.3 Hz, 1H), 6.41 (d, J = 7.6 Hz, 1H), 4.65–4.68 (m, 1H), 4.48 (dd, J = 9.7, 5.2 Hz, 1H), 3.83 (s, 3H), 3.09–3.19 (m, 2H), 2.50 (ddd, J = 18.6, 9.9, 4.4 Hz, 1H), 2.18 (dddd, J = 12.4, 8.6, 6.8, 2.6 Hz, 1H), 2.00 (ddd, J = 14.0, 11.5, 4.4 Hz, 1H), 1.55–1.74 (m, 5H), 0.93 (d, J = 6.2 Hz, 3H), 0.89 (d, J = 6.3 Hz, 3H). ^{13}C NMR (101 MHz, CD3OD): δ/ppm = 180.49, 173.91, 162.78, 154.28, 141.26, 139.85, 138.44, 128.85, 124.96, 118.72, 104.78, 101.73, 98.97, 54.32, 52.48, 45.93, 40.25, 40.05, 38.21, 34.18, 27.32, 24.83, 21.95, 20.63. $[\alpha]_D^{22} = -7$ (*c* 1.00, MeOH). mp: 140 – 142 °C. FT-IR: ν/cm^{-1} = 3293, 2957, 2941, 2878, 2360, 1672, 1662, 1634, 1430, 1357, 1257, 1101, 1099, 764. MS (ESI) *m/z* calculated for $[C_{24}H_{32}N_5O_6]^+$ ($[M+H]^+$): 486.2, found: 486.2. Purity: 99%

86, *tert*-Butyl ((*S*)-1-(methoxy(methyl)amino)-1-oxo-3-((*S*)-2-oxopyrrolidin-3-yl)propan-2-yl)carbamate

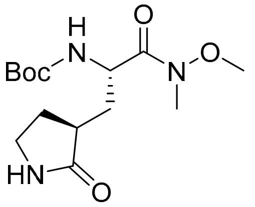

85[26] (1000 mg, 3.49 mmol, 1 eq) was dissolved in a mixture of water/MeOH/THF (1:1:1, 15 ml) and cooled to 0 °C. LiOH (251 mg, 10.48 mmol, 3 eq) was added and the solution was stirred at 4 °C overnight. The addition of hydrochloric acid (1 M, 15 ml) was followed by extraction with a mixture of chloroform and isopropyl alcohol (3:1, 4x 15 ml). The combined organic layers were dried over sodium sulfate and the solvent was removed under reduced pressure. The resulting residue was taken up in DMF (15 ml) and cooled to 0 °C. 2,4,6-collidine (1.39 ml, 10.48 mmol, 3 eq) and HATU (1.46 g, 3.84 mmol, 1.1eq) were added and stirring was continued at 0 °C for 30 min followed by the addition of *N*,*O*-dimethylhydroxalamine hydrochloride and the mixture was stirred over night at room temperature. After removing the solvent under reduced pressure, the resulting residue was purified by reversed phase flash chromatography (H_2O/MeCN, gradient) yielding the desired product as a colorless resin (839 mg, 2.66 mmol, 76%). ^1H NMR (300 MHz, $CDCl_3$): δ/ppm = 6.51 (s, 1H), 5.45 (s, 1H), 4.65 (d, J = 9.9 Hz, 1H), 3.76 (s, 3H), 3.38 – 3.29 (m, 2H), 3.19 (s, 3H), 2.56 – 2.38 (m, 2H), 2.22 – 2.00 (m, 1H), 1.91 – 1.75 (m, 1H), 1.72 – 1.59 (m, 1H), 1.41 (s, 9H). ^{13}C NMR (75 MHz, $CDCl_3$): δ/ppm = 180.08, 173.03, 155.90, 79.77, 61.74, 49.42, 40.61, 38.24, 34.45, 32.39, 28.46, 28.02. $[\alpha]_D^{22}$ = +9 (*c* 1.00, $CHCl_3$). FT-IR: ν/cm^{-1} = 3295, 2976, 1687, 1519, 1503, 1440, 1390, 1365, 1252, 1163, 1114, 1047, 1017, 992, 952, 864, 759, 697. MS (ESI) *m/z* calculated for $[C_{14}H_{25}N_3O_5Na]^+$ ($[M+Na]^+$): 338.2, found: 338.1.

89, *tert*-Butyl ((*S*)-1-(benzo[*d*]thiazol-2-yl)-1-oxo-3-((*S*)-2-oxopyrrolidin-3-yl)propan-2-yl)carbamate

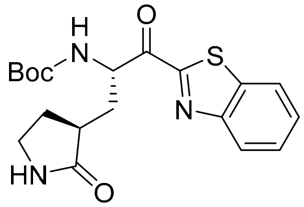

Benzothiazole (2.01 ml, 18.39 mmol, 20 eq) was dissolved in dry THF (10 ml) and cooled to −78 °C. While stirring at −78 °C a solution of *n*-BuLi (2.5 M in hexanes, 1.9 ml, 4.60 mmol, 5 eq) was added dropwise and stirring was continued for one hour at −78 °C. A solution of **86** (290 mg, 0.92 mmol, 1 eq) in dry THF (5 ml) was added dropwise and stirring was continued for four hours at −78 °C. Saturated NH_4Cl solution (20 ml) was added and the mixture was allowed to warm up to room temperature for 30 min. The mixture was extracted with EtOAc (4x 15 ml) and the combined organic layers were washed with water (2x 15 ml) and brine (2x 15 ml) an subsequently dried over $NaSO_4$ and the solvent was removed under reduced pressure. The resulting residue was purified by column chromatography (DCM/MeOH, 97:3) yielding the desired product as a colorless solid (211 mg, 0.54 mmol, 59%). ^{1}H NMR (300 MHz, $CDCl_3$): δ/ppm = 8.24 – 8.11 (m, 1H), 8.02 – 7.94 (m, 1H), 7.65 – 7.48 (m, 2H), 6.30 (s, 1H), 5.86 (s, 2H), 5.58 (s, 2H), 3.45 – 3.34 (m, 2H), 2.73 – 2.50 (m, 2H), 2.19 – 2.03 (m, 3H), 1.43 (s, 9H). ^{13}C NMR (75 MHz, $CDCl_3$): δ/ppm = 193.25, 179.76, 163.75, 153.47, 137.25, 127.99, 127.10, 125.70, 122.42, 79.99, 55.37, 40.49, 38.54, 34.57, 28.31, 27.99. $[\alpha]_D^{22}$ = +36 (*c* 1.00, $CHCl_3$). mp: 88 – 90 °C. FT-IR: ν/cm^{-1} = 3280, 2977, 1693, 1483, 1457, 1391, 1366, 1317, 1253, 1167, 1047, 1022, 904, 879, 761, 731. MS (ESI) *m/z* calculated for $[C_{19}H_{23}N_3O_4Na]^+$ ($[M+Na]^+$): 412.1, found: 412.2.

90, *N*-((*S*)-1-(((*S*)-1-(Benzo[*d*]thiazol-2-yl)-1-oxo-3-((*S*)-2-oxopyrrolidin-3-yl)propan-2-yl)amino)-4-methyl-1-oxopentan-2-yl)-4-methoxy-1*H*-indole-2-carboxamide

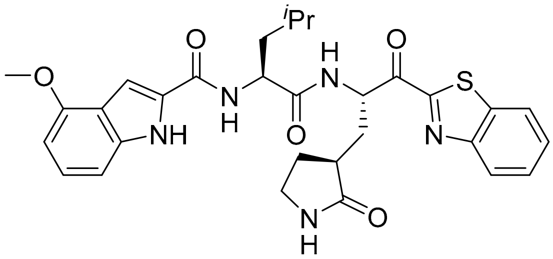

89 (90 mg, 0.23 mmol, 1 eq) was dissolved in DCM (8 ml) and cooled to 0 °C. TFA (2 ml) was added dropwise to the solution while stirring. After stirring for one hour at 0 °C the solvent was removed under reduced pressure and the residue was taken up in a mixture of acetonitrile and water (1:1, 10 ml) and lyophilized overnight. The resulting residue was taken up in dry THF (5 ml) and cooled to −20 °C (amine solution). A solution of **77** (77 mg, 0.25 mmol, 1.1 eq) in dry THF (10 ml) was cooled to −20 °C. NMM (76 µL, 0.69 mmol, 3 eq) was added to the mixture followed by the addition of ethyl chloroformate (24 µL, 0.25 mmol, 1.1 eq) while stirring at −20 °C. After stirring for 15 min at −20 °C the amine solution was added and the mixture was stirred at −20 °C for 15 min. The reaction allowed to warm up to room temperature while stirring for 30 min. The solvent was removed under reduced pressure and the residue was purified preparative HPLC yielding the desired product as a colorless solid (72 mg, 0.125 mmol, 54%). ^{1}H NMR (300 MHz, $CDCl_3$): δ/ppm = 10.36 – 10.05 (m, 1H), 8.93 – 8.57 (m, 1H), 8.19 – 8.02 (m, 1H), 8.00 – 7.89 (m, 1H), 7.59 – 7.45 (m, 2H), 7.19 – 6.93 (m, 4H), 6.42 (t, J = 7.8 Hz, 1H), 5.91 – 5.69 (m, 1H), 5.03 – 4.79 (m, 1H), 3.89 (s, 3H), 3.37 – 3.17 (m,

2H), 3.16 – 2.97 (m, 1H), 2.78 – 2.60 (m, 1H), 2.59 – 1.61 (m, 6H), 0.98 – 0.87 (m, 6H). ^{13}C NMR (75 MHz, $CDCl_3$): δ/ppm = 192.33, 180.42, 173.02, 163.98, 162.12, 154.24, 153.53, 138.20, 137.35, 129.24, 129.05, 128.07, 127.17, 125.81, 125.58, 122.48, 118.94, 105.61, 105.39, 99.67, 55.41, 54.94, 42.01, 40.91, 39.19, 33.18, 28.13, 25.00, 22.35, 21.96. $[\alpha]_D^{22}$ = +21 (*c* 1.00, $CHCl_3$). mp: 142 – 144 °C. FT-IR: ν/cm^{-1} = 3261, 2954, 1678, 1633, 1542, 1514, 1484, 1429, 1359, 1254, 1215. MS (ESI) *m/z* calculated for $[C_{30}H_{34}N_5O_5S]^+$ ($[M+H]^+$): 576.2, found: 576.1. Purity: 97%.

91, *tert*-Butyl ((*S,E*)-1-((*S*)-2-oxopyrrolidin-3-yl)-4-(phenylsulfonyl)but-3-en-2-yl)carbamate

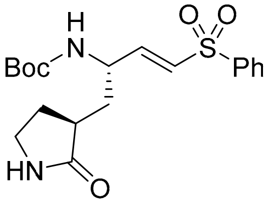

86 (308 mg, 0.98 mmol, 1 eq) was dissolved in dry THF (15 ml) and cooled to −20 °C followed by the addition of $LiAlH_4$ (44 mg, 1.17 mmol, 1.2 eq). The mixture was stirred at −20 °C for one hour after which the reaction was quenched by the addition of hydrochloric acid (0.1 M, 10 ml). The pH of the mixture was adjusted to neutral using hydrochloric acid (0.1 M) and saturated $NaHCO_3$ solution, while stirring at room temperature. The neutralized solution was extracted with a mixture of chloroform and isopropyl alcohol (3:1, 4x 15 ml) and the combined organic layers were dried over Na_2SO_4 and the solvent was removed under reduced pressure. The resulting residue contained the respective aldehyde and was taken up in dry MeCN (2 ml). A solution of LiCl (50 mg, 1.17 mmol, 1.2 eq) and **3** (285 mg, 0.98 mmol, 1 eq) in dry MeCN (15 ml) was cooled to 0 °C. DBU (146 µL, 0.98 mmol, 1 eq) was added and the mixture was stirred at 0 °C for 20 min followed by the dropwise addition of the aldehyde solution. Stirring was continued at 0 °C for one hour after which the solvent was removed under reduced pressure and the resulting residue was purified by column chromatography (DCM/MeOH, 98:2) yielding the desired product as a colorless solid (251 mg, 0.64 mmol, 65%). ^{1}H NMR (300 MHz, $CDCl_3$): δ/ppm = 7.92 – 7.82 (m, 2H), 7.67 – 7.47 (m, 3H), 6.89 (dd, *J* = 15.0, 5.1 Hz, 1H), 6.47 (dd, *J* = 15.0, 1.5 Hz, 1H), 6.21 – 6.07 (m, 1H), 5.67 – 5.41 (m, 1H), 4.38 (s, 1H), (3.42 – 3.25 (m, 2H), (2.53 – 2.34 (m, 2H), 2.06 – 1.90 (m, 1H), 1.90 – 1.73 (m, 1H), 1.70 – 1.55 (m, 1H), 1.36 (s, 9H). ^{13}C NMR (75 MHz, $CDCl_3$): δ/ppm = 179.74, 155.65, 155.35, 146.22, 140.19, 133.46, 130.61, 129.31, 127.66, 80.07, 62.46, 53.42, 40.60, 38.26, 35.20, 28.42, 28.22. $[\alpha]_D^{22}$ = −2 (*c* 1.00, $CHCl_3$). mp: 84 – 86 °C. FT-IR: ν/cm^{-1} = 3268, 2979, 1692, 1514, 1502, 1446, 1391, 1366, 1306, 1251, 1166, 1146, 1086, 1048, 1024, 971, 843, 753, 720, 688. MS (ESI) *m/z* calculated for $[C_{19}H_{26}N_2O_5SNa]^+$ ($[M+Na]^+$): 417.2, found: 417.1.

92, *tert*-Butyl ((*S*,*E*)-4-fluoro-1-((*S*)-2-oxopyrrolidin-3-yl)-4-(phenylsulfonyl)but-3-en-2-yl)carbamate

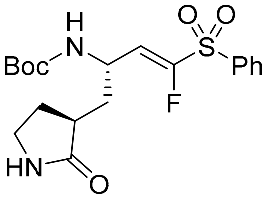

86 (308 mg, 0.98 mmol, 1 eq) was dissolved in dry THF (15 ml) and cooled to −20 °C followed by the addition of $LiAlH_4$ (44 mg, 1.17 mmol, 1.2 eq). The mixture was stirred at −20 °C for one hour after which the reaction was quenched by the addition of hydrochloric acid (0.1 M, 10 ml). The pH of the mixture was adjusted to neutral using hydrochloric acid (0.1 M) and saturated $NaHCO_3$ solution, while stirring at room temperature. The neutralized solution was extracted with a mixture of chloroform and isopropyl alcohol (3:1, 4x 15 ml) and the combined organic layers were dried over $NaSO_4$ and the solvent was removed under reduced pressure. The resulting residue contained the respective aldehyde and was taken up in dry MeCN (2 ml). A solution of LiCl (50 mg, 1.17 mmol, 1.2 eq) and **4** (302 mg, 0.98 mmol, 1 eq) in dry MeCN (15 ml) was cooled to 0 °C. DBU (146 µL, 0.98 mmol, 1 eq) was added and the mixture was stirred at 0 °C for 20 min followed by the dropwise addition of the aldehyde solution. Stirring was continued at 0 °C for one hour after which the solvent was removed under reduced pressure and the resulting residue was purified by column chromatography (DCM/MeOH, 98:2) yielding the desired product as a colorless solid (143 mg, 0.35 mmol, 35%). ^{1}H NMR (400 MHz, $CDCl_3$): δ/ppm = 8.20 – 8.09 (m, 2H), 7.73 – 7.64 (m, 1H), 7.62 – 7.52 (m, 2H), 6.38 (s, 1H), 5.80 (dd, J = 20.8, 9.5 Hz, 1H), 5.44 – 5.29 (m, 1H), 3.46 – 3.26 (m, 2H), 2.63 – 2.53 (m, 1H), 2.52 – 2.41 (m, 1H), 2.21 – 2.07 (m, 1H), 2.07 – 1.95 (m, 1H), 1.80 – 1.66 (m, 1H), 1.43 (s, 9H). ^{13}C NMR (101 MHz, $CDCl_3$): δ/ppm = 180.42, 155.63, 153.54, 150.63, 137.60, 134.72, 129.47, 129.17, 122.65, 122.54, 79.75, 45.64, 40.78, 38.54, 36.97, 28.48, 28.06. $[\alpha]_D^{22}$ = –31 (*c* 1.00, $CHCl_3$). mp: 178 – 180 °C. FT-IR: ν/cm^{-1} = 1689, 1508, 1449, 1438, 1391, 1365, 1335, 1291, 1267, 1160, 1121, 1097, 1078, 1042, 1026, 1003, 862, 761, 733, 685. MS (ESI) *m/z* calculated for $[C_{19}H_{25}FN_2O_5SNa]^+$ ($[M+Na]^+$): 435.1, found: 435.1.

93, 4-Methoxy-*N*-((*S*)-4-methyl-1-oxo-1-(((*S*,*E*)-1-((S)-2-oxopyrrolidin-3-yl)-4-(phenylsulfonyl)but-3-en-2-yl)amino)pentan-2-yl)-1*H*-indole-2-carboxamide

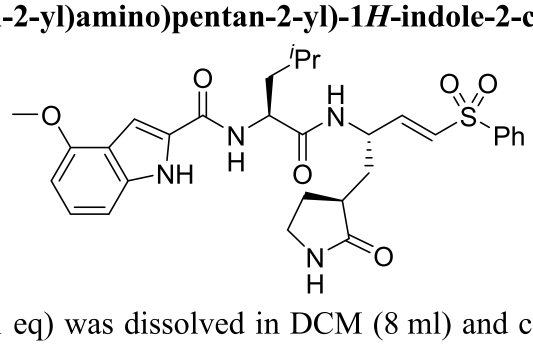

91 (103 mg, 0.26 mmol, 1 eq) was dissolved in DCM (8 ml) and cooled to 0 °C. TFA (2 ml) was added dropwise to the solution while stirring. After stirring for one hour at 0 °C the solvent was removed under reduced pressure and the residue was taken up in a mixture of acetonitrile and water (1:1, 10 ml) and lyophilized over night. The resulting residue was taken up in dry THF (5 ml) and cooled to −20 °C (amine solution). A solution of **77** (95 mg, 0.31 mmol, 1.2 eq) in dry THF (10 ml) was cooled to −20 °C. NMM (96 µL, 0.86 mmol, 3.3 eq) was added to the mixture followed by the addition of ethyl chloroformate (30 µL, 0.31 mmol, 1.2 eq) while stirring at −20 °C. After stirring for 15 min at −20 °C the amine solution was added and the mixture was stirred at −20 °C for 15 min. The mixture was allowed to warm up to room

temperature while stirring for 30 min. The solvent was removed under reduced pressure and the residue was purified by preparative HPLC yielding the desired product as a colorless solid (28 mg, 0.048 mmol, 18%). ^{1}H NMR (300 MHz, DMSO-d_6): δ/ppm = 11.55 (s, 1H), 8.55 – 8.30 (m, 1H), 7.88 – 7.77 (m, 2H), 7.74 – 7.54 (m, 3H), 7.34 (s, 1H), 7.14 – 7.07 (m, 1H), 7.06 – 6.99 (m, 1H), 6.91 (dd, J = 15.1, 4.9 Hz, 1H), 6.66 (dd, J = 15.1, 1.5 Hz, 1H), 6.51 (d, J = 7.5 Hz, 1H), 4.72 – 4.56 (m, 1H), 4.52 – 4.33 (m, 2H), 3.88 (s, 3H), 3.18 – 2.97 (m, 2H), 2.42 – 1.42 (m, 8H), 0.97 – 0.80 (m, 6H). ^{13}C NMR (75 MHz, DMSO): δ/ppm = 178.70, 174.66, 172.65, 172.57, 161.59, 154.11, 147.83, 140.70, 138.29, 134.11, 130.29, 130.08, 127.52, 124.91, 118.54, 105.87, 101.71, 99.71, 55.54, 52.34, 50.90, 47.53, 38.09, 34.92, 27.65, 24.99, 24.89, 23.41, 23.25, 22.04, 21.60. mp: 172 – 174 °C. FT-IR: ν/cm^{-1} = 3266, 2958, 1658, 1623, 1583, 1547, 1515, 1463, 1429, 1359, 1306, 1254, 1145, 1099, 1086, 975, 828, 756, 722, 686. MS (ESI) *m/z* calculated for $[C_{30}H_{37}N_4O_6S]^+$ ($[M+H]^+$): 518.2, found: 581.4. Purity: 99%.

94, *N*-((*S*)-1-(((*S,E*)-4-Fluoro-1-((*S*)-2-oxopyrrolidin-3-yl)-4-(phenylsulfonyl)but-3-en-2-yl)amino)-4-methyl-1-oxopentan-2-yl)-4-methoxy-1*H*-indole-2-carboxamide

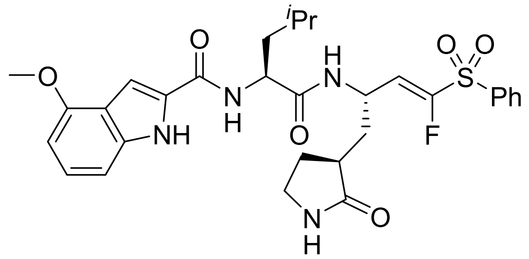

94 (131 mg, 0.32 mmol, 1 eq) was dissolved in DCM (8 ml) and cooled to 0 °C. TFA (2 ml) was added dropwise to the solution while stirring. After stirring for one hour at 0 °C the solvent was removed under reduced pressure and the residue was taken up in a mixture of acetonitrile and water (1:1, 10 ml) and lyophilized overnight. The resulting residue was taken up in dry THF (5 ml) and cooled to −20 °C (amine solution). A solution of **77** (106 mg, 0.35 mmol, 1.1 eq) in dry THF (10 ml) was cooled to −20 °C. NMM (106 µL, 0.96 mmol, 3 eq) was added to the mixture followed by the addition of ethyl chloroformate (30 µL, 0.31 mmol, 1.2 eq) while stirring at −20 °C. After stirring for 15 min at −20 °C the amine solution was added and the mixture was stirred at −20 °C for 15 min. The mixture was allowed to warm up to room temperature while stirring for 30 min. The solvent was removed under reduced pressure and the residue was purified by preparative HPLC yielding the desired product as a colorless solid (28 mg, 0.05 mmol, 15%). ^{1}H NMR (600 MHz, DMSO-d_6): δ/ppm = 11.59 (d, J = 2.3 Hz, 1H), 8.45 – 8.38 (m, 2H), 7.94 – 7.89 (m, 2H), 7.87 – 7.82 (m, 1H), 7.74 – 7.69 (m, 2H), 7.67 – 7.60 (m, 1H), 7.38 – 7.34 (m, 1H), 7.09 (t, J = 7.9 Hz, 1H), 7.00 (d, J = 8.2 Hz, 1H), 6.50 (d, J = 7.6 Hz, 1H), 6.36 (dd, J = 33.9, 8.7 Hz, 1H), 4.76 – 4.69 (m, 1H), 4.46 – 4.35 (m, 1H), 3.88 (s, 3H), 3.10 – 2.99 (m, 2H), 2.32 – 2.24 (m, 1H), 2.13 – 1.98 (m, 2H), 1.67 – 1.53 (m, 3H), 1.49 – 1.43 (m, 1H), 1.42 – 1.35 (m, 1H), 0.94 – 0.80 (m, 6H). ^{13}C NMR (151 MHz, DMSO-d_6): δ/ppm = 178.03, 171.99, 161.09, 153.66, 137.83, 136.43, 129.88, 128.19, 124.45, 119.47, 118.08, 105.42, 55.10, 51.60, 42.46, 40.19, 40.06, 37.43, 34.96, 27.31, 24.41, 22.99, 21.47. $[\alpha]_D^{22}$ = +4 (c 1.00, MeOH). mp: 194 – 196 °C. FT-IR: ν/cm^{-1} = 3266, 2957, 1687, 1659, 1622, 1583, 1548, 1514, 1462, 1447, 1428, 1334, 1252, 1214, 1165, 1131, 1099, 834, 755, 723, 685. MS (ESI) *m/z* calculated for $[C_{30}H_{36}FN_4O_6S]^+$ ($[M+H]^+$): 599.2, found: 599.1. Purity: 99%.

95, tert-Butyl ((2*S*)-1-oxo-1-((2-oxo-1-phenylazetidin-3-yl)amino)-3-((*S*)-2-oxopyrrolidin-3-yl)propan-2-yl)carbamate

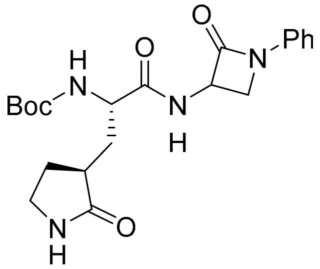

85[26] (88 mg, 0.31 mmol, 1 eq) was dissolved in a mixture of water/MeOH/THF (1:1:1, 15 ml) and cooled to 0 °C. LiOH (22 mg, 0.93 mmol, 3 eq) was added and the solution was stirred at 4 °C over night. The addition of hydrochloric acid (1 M, 10 ml) was followed by extraction with a mixture of chloroform and isopropyl alcohol (3:1, 4x 15 ml). The combined organic layers were dried over sodium sulfate and the solvent was removed under reduced pressure. The resulting residue was taken up in DMF (10 ml) and cooled to 0 °C. DIPEA (161 µL, 0.93 mmol, 3 eq) and TBTU (109, 0.34 mmol, 1.1 eq) were added and stirring was continued at 0 °C for 30 min followed by the addition of **108** (67 mg, 0.34 mmol, 1.1 eq) and the mixture was stirred over night at room temperature. After removing the solvent under reduced pressure, the resulting residue was purified by preparative HPLC yielding the desired product as a colorless solid (50 mg, 0.12 mmol, 39%). ^{1}H NMR (300 MHz, CD_3OD): δ/ppm = 7.45 – 7.28 (m, 4H), 7.17 – 7.06 (m, 1H), 5.12 – 5.04 (m, 1H), 4.29 – 4.08 (m, 1H), 4.01 (t, J = 5.7 Hz, 1H), 3.78 – 3.65 (m, 1H), 3.37 – 3.34 (m, 2H), 2.59 – 2.30 (m, 2H), 2.13 – 1.98 (m, 1H), 1.94 – 1.68 (m, 2H), 1.45 (s, 9H). ^{13}C NMR (75 MHz, CD_3OD): δ/ppm = 181.90, 175.24, 166.09, 157.88, 139.41, 130.22, 125.39, 117.78, 80.81, 56.30, 54.66, 49.28, 47.79, 41.52, 39.77, 34.93, 28.67. mp: 193 – 195 °C. FT-IR: ν/cm^{-1} = 3441, 2977, 2468, 1750, 1668, 1599, 1503, 1456, 1391, 1367, 1291, 1159, 1049, 862, 756, 692. MS (ESI) *m/z* calculated for $[C_{21}H_{28}N_4O_6Na]^+$ ($[M+Na]^+$): 439.2, found: 439.4. Purity: 99%.

96, 4-Methoxy-*N*-((2*S*)-4-methyl-1-oxo-1-(((2*S*)-1-oxo-1-((2-oxo-1-phenylazetidin-3-yl)amino)-3-((*S*)-2-oxopyrrolidin-3-yl)propan-2-yl)amino)pentan-2-yl)-1*H*-indole-2-carboxamide

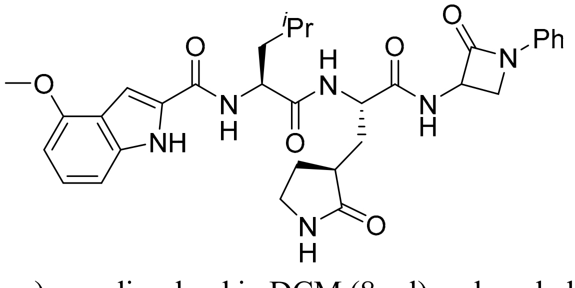

95 (50 mg, 0.12 mmol, 1 eq) was dissolved in DCM (8 ml) and cooled to 0 °C. TFA (2 ml) was added dropwise to the solution while stirring. After stirring for one hour at 0 °C the solvent was removed under reduced pressure and the residue was taken up in a mixture of acetonitrile and water (1:1, 10 ml) and lyophilized overnight. The resulting residue was taken up in dry THF (5 ml) and cooled to −20 °C (amine solution). A solution of **77** (40 mg, 0.13 mmol, 1.1 eq) in dry THF (10 ml) was cooled to −20 °C. NMM (40 µL, 0.36 mmol, 3 eq) was added to the mixture followed by the addition of ethyl chloroformate (13 µL, 0.13 mmol, 1.1 eq) while stirring at −20 °C. After stirring for 15 min at −20 °C the amine solution was added and the mixture was stirred at −20 °C for 15 min. The mixture was allowed to warm up to room temperature while stirring for 30 min. The solvent was removed under reduced pressure and the

residue was purified by preparative HPLC yielding the desired product as a colorless solid (58 mg, 0.10 mmol, 80%). ^{1}H NMR (300 MHz, DMSO-d_6): δ/ppm = 11.56 (s, 1H), 8.73 (d, J = 8.3 Hz, 1H), 8.41 (d, J = 7.9 Hz, 1H), 8.19 (d, J = 8.1 Hz, 1H), 7.69 – 7.55 (m, 1H), 7.49 – 7.25 (m, 5H), 7.18 – 6.93 (m, 3H), 6.50 (d, J = 7.5 Hz, 1H), 5.12 – 4.95 (m, 1H), 4.63 – 4.46 (m, 1H), 4.44 – 4.31 (m, 1H), 4.04 – 3.90 (m, 2H), 3.88 (s, 3H), 3.20 – 3.00 (m, 2H), 2.41 – 1.99 (m, 3H), 1.81 – 1.45 (m, 5H), 0.91 (dd, J = 12.1, 5.9 Hz, 6H). ^{13}C NMR (75 MHz, DMSO-d_6): δ/ppm = 178.38, 172.42, 171.85, 164.51, 161.17, 153.68, 138.12, 137.88, 129.93, 129.27, 124.52, 123.75, 118.12, 116.21, 105.44, 101.21, 99.29, 55.11, 54.93, 51.65, 50.87, 46.23, 40.21, 39.80, 37.70, 33.79, 27.38, 24.48, 23.19, 21.38. $[\alpha]_D^{22}$ = +33 (c 1.00, $CHCl_3$). mp: 160 °C decomposition. FT-IR: ν/cm^{-1} = 3283, 2976, 1745, 1691, 1563, 1547, 1514, 1502, 1462, 1441, 1390, 1366, 1254, 1166, 1100, 1049, 755. MS (ESI) m/z calculated for $[C_{32}H_{33}N_6O_6]^+$ ($[M+H]^+$): 603.3, found: 603.4. Purity: 95%.

Synthesis of uPA targeting compounds

General procedure of Fmoc-Solid phase peptide synthesis (SPPS)

A resin Loading

Solid phase peptide synthesis was prepared in a fritted 12 mL polypropylene syringe. 1 g of 2-Chlorotritylchloride resin (2-CTC resin, 1.2 mmol loading capacity) was prewetted in 8 mL DCM for 15 min and drained. The first amino acid (3.6 mmol, 3 eq relative to resin loading capacity) was added in 1.8 M NMM/DCM (6 mL) and the mixture was mixed on a rocker for 12 h. After draining the solution, the resin was washed with DMF (3x 6 mL, 1 min each) and DCM (3 x 6 mL, 1 min each).

B resin capping

The remaining free 2-CTC resin linkers were capped with methanol in a solution of DCM/MeOH/DIPEA (9:2:1). The resin was mixed on a rocker for one hour, drained and washed with DCM (3x 6 mL).

C Fmoc deprotection

The resin was treated with a 20% piperidine in DMF solution (2x 6 mL, 10 min each) and subsequently washed with DMF (3x 6 mL).

D peptide coupling with HATU

A coupling cocktail was prepared consisting of the specific Fmoc-protected amino acid (3.6 mmol, 3 eq) with HATU (3.6 mmol, 3 eq) and DIEA (10.8 mmol, 3 eq) in DMF (4.8 mL). The solution was added to the resin and mixed on a rocker for 3 h with exception of the amino acid Fmoc-Arg(Pbf)-OH which coupled overnight. After the reaction, the resin was drained and washed with DMF (3x 6 mL, 1 min each) and DCM (3x 6 mL, 1 min each).

E resin cleavage under preservation of protecting groups

After the last coupling, the resin washed with DMF (3x 6 mL, 1 min each) and DCM (3x 6 mL, 1 min each). Then the cleavage cocktail containing AcOH/Trifluoroisopropanol/DCM (1:1:4, 6 mL) was added and mixed on a rocker for 1.5 h. The resin was drained and washed with DCM (5 mL). The combined organic solvents were concentrated under reduced pressure to yield the crude linear peptide. The purification of the crude product was done by reversed phase flash chromatography (H_2O/MeCN, gradient).

99, *N*-(Acetylglycyl)-*O*-(tert-butyl)-L-allothreonyl-L-alanine

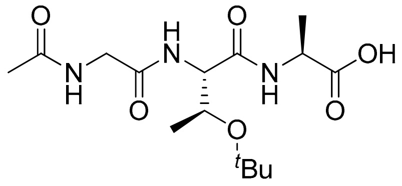

The title compound was prepared according to general procedures A, B, C, D, and E on 1.2 mmol scale. The product was purified by reversed phase flash chromatography (H_2O/MeCN, gradient) and obtained as a colorless solid after lyophilization (414 mg, 0.6 mmol, 50%) ^{1}H NMR (300 MHz, $CDCl_3$): δ/ppm = 8.19 (t, *J* = 5.8 Hz, 1H), 7.95 (d, *J* = 7.0 Hz, 1H), 7.53 (d, *J* = 8.5 Hz, 1H), 4.26 – 4.16 (m, 2H), 3.92 (qd, *J* = 6.2, 3.9 Hz, 2H), 3.74 (dd, *J* = 7.2, 5.8 Hz, 2H), 1.86 (s, 3H), 1.28 (d, *J* = 7.2 Hz, 3H), 1.12 (s, 9H), 1.01 (d, *J* = 6.2 Hz, 3H). ^{13}C NMR (75 MHz, CDCl3): δ/ppm = 173.8, 169.9, 169.3, 168.9, 73.8, 67.2, 57.3, 47.7, 42.3, 28.1, 22.4, 19.3, 17.5. mp: 87 – 95 °C. $[\alpha]_D^{20}$ = +12 (*c* 1.00, $CHCl_3$). FT-IR: ν/cm^{-1} = 668, 701, 1081, 1121, 1159, 1192, 1214, 1371, 1518, 1636. MS (ESI) *m/z* calculated for $[C_{15}H_{28}N_3O_6]^+$ ($[M+H]^+$): 346.2, found 368.1. Purity: 97.8%.

101, *tert*-Butyl (*S*)-(1-(methoxy(methyl)amino)-1-oxo-5-(3-((2,2,4,6,7-pentamethyl-2,3-dihydrobenzofuran-5-yl)sulfonyl)guanidino)pentan-2-yl)carbamate

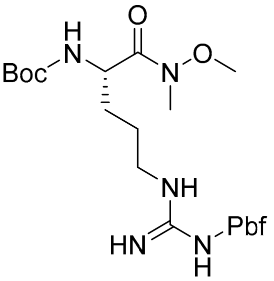

(Adapted from St-Georges *et al.*)[27]

Boc-Arg(Pbf)-OH **100** (6.00 g, 11.39 mmol, 1 eq) was dissolved in DCM (40 mL) under argon atmosphere. At 0 °C DIPEA (15.9 mL, 31.12 mmol, 8 eq) and TBTU (4.38 g, 13.67 mmol, 1.2 eq) were added. The reaction mixture stirred for 30 min at 0 °C, *N*,*O*-dimethyl hydroxylamine · HCl was added and stirred for 12 h. The solution was diluted with DCM (20 mL) and washed with saturated $NaHCO_3$ solution (3x 20 mL) and saturated NaCl solution (3x 20 mL). The organic solution dried over anhydrous Na_2SO_4 and evaporated under reduced pressure. The crude product was purified by column chromatography (EA) to yield the desired product as a colorless solid (6.16 g, 10.82 mmol, 95%). ^{1}H NMR (300 MHz, CDCl3): δ/ppm = 6.38 (s, 1H), 5.49 (d, *J* = 8.8 Hz, 1H), 4.61 (s, 1H), 3.73 (s, 3H), 3.18 (s, 3H), 2.95 (s, 3H), 2.56 (s, 3H), 2.50 (s, 3H), 2.08 (s, 3H), 1.74 – 1.52 (m, 4H), 1.45 (s, 6H), 1.41 (s, 9H) ppm, ^{13}C NMR (75 MHz, CDCl3): δ/ppm = 156.4, 156.0, 138.8, 132.7, 124.8, 117.7, 86.6, 80.3, 61.7, 43.3, 41.0, 30.9, 28.7, 28.4, 24.9, 19.4, 18.0, 12.5. mp: 76 – 84 °C. $[\alpha]_D^{20}$ = −7 (*c* 1.00, $CHCl_3$). FT-IR: ν/cm^{-1} = 668, 756, 1106, 1165, 1456, 1556, 1652, 2362, 2974, 2981. MS (ESI) *m/z* calculated for $[C_{26}H_{44}N_5O_7S]^+$ ($[M+H]^+$): 570.3, found 570.3.

102, *tert*-Butyl (1-(benzo[*d*]thiazol-2-yl)-1-oxo-5-(3-((2,2,4,6,7-pentamethyl-2,3-dihydrobenzofuran-5-yl)sulfonyl)guanidino)pentan-2-yl)carbamate

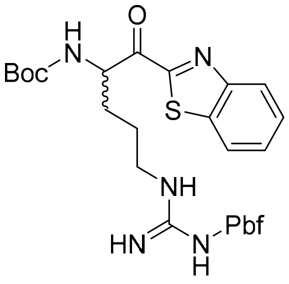

(Adapted from Costanzo *et al.*)[28]

Benzothiazole (3.47 g, 25.69 mmol, 10.2 eq) was dissolved in dry THF (70 mL) under argon atmosphere and cooled to −78 °C. *n*-BuLi (2.5 in hexanes, 2,97 mL, 25.19 mmol, 10 eq) was added dropwise and the reaction stirred for 1.5 h at the same temperature. Afterwards, **101** (1.43 g, 2.51 mmol, 1 eq) dissolved in dry THF (15 mL) was added slowly to the reaction mixture and stirred for two hours at −78 °C. The reaction mixture was quenched with saturated NH_4Cl solution (20 mL). The organic phase was separated and washed three times each with saturated $NaHCO_3$ solution (25 mL) and saturated NaCl solution (25 mL). The organic phase was dried over anhydrous $NaSO_4$ and concentrated under reduced pressure. The crude product was purified by column chromatography (CH/EA, 1:1 to 0:1) to yield the product as a colorless solid (1.11 g, 1.90 mmol, 76%). ^{1}H NMR (300 MHz, CDCl3): δ/ppm = 8.23 – 8.15 (m, 1H), 8.00 – 7.91 (m, 1H), 7.60 – 7.49 (m, 2H), 6.31 (s, 1H), 6.21 (s, 2H), 5.65 (d, J = 8.6 Hz, 1H), 5.61 – 5.49 (m, 1H), 3.57 – 3.18 (m, 2H), 2.92 (s, 3H), 2.54 (s, 3H), 2.49 (s, 3H), 2.06 (s, 3H), 1.72 (s, 4H), 1.45 (s, 6H), 1.41 (s, 9H). ppm, ^{13}C NMR (75 MHz, CDCl3): δ/ppm = 158.8, 156.2, 153.5, 138.5, 137.3, 133.0, 132.5, 128.3, 127.4, 126.0, 124.7, 122.5, 117.5, 86.4, 80.6, 43.3, 40.8, 28.7, 28.4, 25.5, 19.3, 18.0, 12.5. mp: 105 – 115 °C. $[\alpha]_D^{20}$ = +2 (*c* 1.00, $CHCl_3$). FT-IR: ν/cm^{-1} = 668, 756, 1102, 1164, 1216, 1369, 1483, 1552, 1622, 1694. MS (ESI) *m/z* calculated for $[C_{31}H_{42}N_5O_6S_2]^+$ ($[M+H]^+$): 644.3, found 644.3. Purity: 98%.

104, *tert*-Butyl (*S,E*)-(6-(3-((2,2,4,6,7-pentamethyl-2,3-dihydrobenzofuran-5-yl)sulfonyl)guanidino)-1-(phenylsulfonyl)hex-1-en-3-yl)carbamate

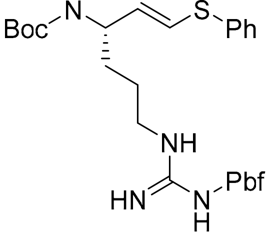

(Adapted from Andreasen *et al.*)[29]

101 (1 g, 1.76 mmol, 1 eq) was dissolved in dry THF (20 mL) under argon atmosphere and cooled to 0 °C. $LiAlH_4$ (1 M in THF, 2.2 mL, 1.25 eq) was added dropwise to the solution at 0 °C and stirred one hour at the same temperature. The reaction mixture was quenched with $KHSO_4$ solution (5%, 10 mL) and diluted with EtOAc (20 mL). The organic phase was washed with saturated $NaHCO_3$ solution (25 mL) and saturated NaCl solution (25 mL), dried over

anhydrous Na_2SO_4 and evaporated under reduced pressure, to yield the crude aldehyde as a colorless oil (0.67 g). The crude aldehyde (0.39 g, 0.76 mmol, 1 eq) and LiCl (0.04 g, 0.91 mmol, 1.2 eq) were dissolved in dry MeCN (15 mL) under argon atmosphere and cooled to 0 °C. Subsequently DBU (0.12 mL, 0.76 mmol, 1 eq) and 10 min later **3** (0.22 g, 0.76 mmol, 1 eq) in MeCN (2 mL) were added dropwise to the solution and stirred one hour at 0 °C and one hour at room temperature. The reaction mixture was quenched with citric acid solution (10%, 8 mL). The organic phase was separated and the aqueous phase extracted with EtOAc (3x 20 mL). The combined organic extracts were dried over anhydrous Na_2SO_4 and evaporated under reduced pressure. The crude product was purified by column chromatography (CH/EA 1:2) to yield the desired product colorless solid (0.36 g, 0.55 mmol, 72%). ^{1}H NMR (300 MHz, CDCl3): δ/ppm = 7.89 – 7.80 (m, 2H), 7.63 – 7.47 (m, 3H), 6.90 (dd, J = 15.0, 4.6 Hz, 2H), 6.51 – 6.33 (m, 3H), 5.26 (d, J = 8.7 Hz, 1H), 4.31 (s, 1H), 3.30 – 3.16 (m, 2H), 2.55 (s, 3H), 2.08 (s, 3H), 1.68 – 1.54 (m, 4H), 1.45 (s, 6H), 1.34 (s, 9H) ppm, ^{13}C NMR (75 MHz, CDCl3): δ/ppm = 156.1, 155.6, 146.9, 140.1, 138.6, 133.6, 132.6, 130.3, 129.5, 127.7, 124.9, 117.7, 86.6, 43.3, 28.7, 28.3, 19.4, 18.1, 12.6. mp: 73 – 77 °C, $[\alpha]_D^{20}$ = –9 (c 1.00, $CHCl_3$), FT-IR: ν/cm^{-1} = 2957, 1706, 1512, 1358, 1302, 1141, 1091, 908, 748, 731. MS (ESI) m/z calculated for $[C_{31}H_{45}N_4O_7S_2]^+$ ($[M+H]^+$): 649.3, found 649.3.

105, *tert*-Butyl (*S*,*E*)-(1-fluoro-6-(3-phenylguanidino)-1-(phenylsulfonyl)hex-1-en-3-yl)carbamate

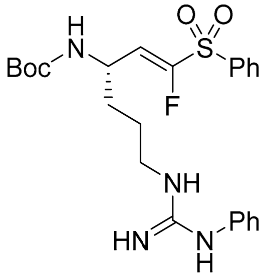

101 (1 g, 1.76 mmol, 1 eq) was dissolved in dry THF (20 mL) under argon atmosphere and cooled to 0 °C. $LiAlH_4$ (1 M in THF, 2.2 mL, 1.25 eq) was added dropwise to the solution at 0 °C and stirred one hour at the same temperature. The reaction mixture was quenched with $KHSO_4$ solution (5%, 10 mL) and diluted with EtOAc (20 mL). The organic phase was washed with saturated $NaHCO_3$ solution (25 mL) and saturated NaCl solution (25 mL), dried over anhydrous Na_2SO_4 and evaporated under reduced pressure, to yield the crude aldehyde as a colorless oil (0.67 g). The crude aldehyde (0.25 g, 0.49 mmol, 1 eq) and LiCl (0.03 g, 0.59 mmol, 1.2 eq) were dissolved in dry MeCN (8 mL) under argon atmosphere and cooled to 0 °C. Subsequently DBU (0.08 mL, 0.49 mmol, 1 eq) and 10 min later **4** (0.15 g, 0.49 mmol, 1 eq) in MeCN (1 mL) were added dropwise to the solution and stirred one hour at 0 °C and one hour at room temperature. The reaction mixture was quenched with citric acid solution (10%, 6 mL). The organic phase was separated, and the aqueous phase extracted with EA (3x 20 mL). The combined organic extracts were dried over Na_2SO_4 and evaporated under reduced pressure. The crude product was purified with column chromatography (CH/EA, 1:1) to yield the desired product as a colorless oil (0.1 g, 0.15 mmol, 31%). ^{1}H NMR (300 MHz, CDCl3): δ/ppm = 7.92 (dd, J = 7.2, 1.8 Hz, 2H), 7.72 – 7.64 (m, 1H), 7.57 (dd, J = 8.3, 6.9 Hz, 2H), 6.37 (s, 2H), 6.19 (d, J = 35.7 Hz, 2H), 5.21 (s, 1H), 4.41 (s, 1H), 3.20 (s, 2H), 2.96 (s, 2H), 2.56 (s, 3H), 2.50 (s, 3H), 2.09 (s, 3H), 1.67 – 1.50 (m, 4H), 1.46 (s, 6H), 1.30 (s, 9H) ppm, ^{13}C NMR

(75 MHz, CDCl3): δ/ppm = 156.1, 155.5, 152.4, 134.7, 129.6, 128.8, 124.9, 117.8, 86.7, 43.3, 40.9, 28.7, 28.3, 25.4, 19.4, 18.0, 12.6. $[\alpha]_D^{20}$ = +9 (*c* 1.00, $CHCl_3$). FT-IR: ν/cm^{-1} = 2974, 1692, 1628, 1563, 1369, 1245, 1161, 1094, 737, 658. MS (ESI) *m/z* calculated for $[C_{31}H_{44}FN_5O_7S_2]^+$ ($[M+H]^+$): 666.3, found 666.8.

General procedure for the amide bond formation between the H_2N-Arg-warhead moiety and 99

The respective H_2N-Arg-warhead moieties **102**, **104**, **105** were deprotected by using a deprotection mix of 16% TFA in DCM (4 ml) and stirred for 30 min at rt. The solvent was removed under reduced pressure to yield the deprotected amines as trifluoroacetate salts.

99 (1.0 eq) was dissolved in DCM and cooled to 0 °C with an ice-water bath. DIPEA (3 eq) and HATU (1.2 eq) were added and stirring was continued for 30 min at 0 °C. Then, the respective deprotected amine (1 eq) as a solution in DMF were added, stirred for 30 min at 0 °C and 16 h at room temperature. DCM and water were added, and the aqueous phase was extracted three times with DCM. The combined organic phases were dried over Na_2SO_4 and the solvent was removed under reduced pressure.

103, (2*S*,3*S*)-2-(2-Acetamidoacetamido)-*N*-((*S*)-1-(((*S*)-1-(benzo[*d*]thiazol-2-yl)-5-guanidino-1-oxopentan-2-yl)amino)-1-oxopropan-2-yl)-3-hydroxybutanamide

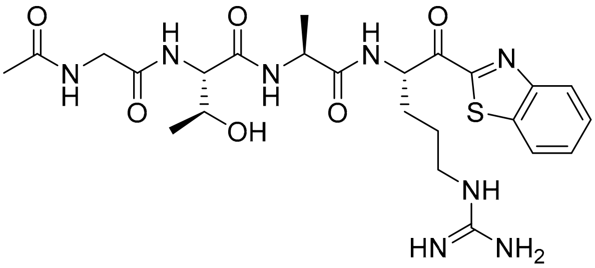

The crude product was used without further purification for the following deprotection of the Pbf- and O^{t}Bu-protecting group. It was dissolved in TFA/DCM (50%, 2 mL) and stirred for 2 h at room temperature. The solution was evaporated under reduced pressure and purified by preparative HPLC (12.8 mg, 0.017 mmol, 10%). ^{1}H NMR (400 MHz, DMSO-d_6): δ/ppm = 8.57 – 8.39 (m, 1H), 8.31 – 8.24 (m, 2H), 8.23 – 8.16 (m, 1H), 8.08 – 7.96 (m, 1H), 7.83 – 7.72 (m, 1H), 7.71 – 7.63 (m, 2H), 7.60 – 7.54 (m, 1H), 5.54 – 5.41 (m, 1H), 5.00 (d, *J* = 35.3 Hz, 1H), 4.36 (ddd, *J* = 10.0, 7.2, 2.7 Hz, 1H), 4.17 (tdd, *J* = 12.1, 8.0, 4.2 Hz, 1H), 3.97 (ddt, *J* = 16.9, 11.2, 5.2 Hz, 1H), 3.83 – 3.69 (m, 2H), 3.14 (p, *J* = 6.6 Hz, 2H), 1.97 (td, *J* = 13.7, 11.4, 6.7 Hz, 2H), 1.90 – 1.82 (m, 2H), 1.79 – 1.69 (m, 0H), 1.65 – 1.54 (m, 2H), 1.27 – 1.17 (m, 3H), 1.06 – 0.98 (m, 3H). ^{13}C NMR (400 MHz, DMSO-d_6): δ/ppm = 193.1, 172.6, 169.9, 169.7, 169.5, 164.5, 156.8, 153.0, 136.5, 128.3, 127.6, 125.3, 123.3, 66.7, 58.2, 54.3, 47.9, 42.2, 42.2, 27.8, 25.2, 22.4, 19.7, 18. $[\alpha]_D^{20}$ = +34 (*c* 1.00, DMSO). FT-IR: ν/cm^{-1} = 660, 668, 756, 1136, 1215, 1673, 1684, 2922, 2964, 3327, MS (ESI) *m/z* calculated for $[C_{24}H_{34}N_8O_6S+H]^+$ ($[M+H]^+$): 563.2, found 563.2. Purity: 98%.

106, (2*S*,3*S*)-2-(2-acetamidoacetamido)-N-((*S*)-1-(((*S,E*)-6-guanidino-1-(phenylsulfonyl)hex-1-en-3-yl)amino)-1-oxopropan-2-yl)-3-hydroxybutanamide

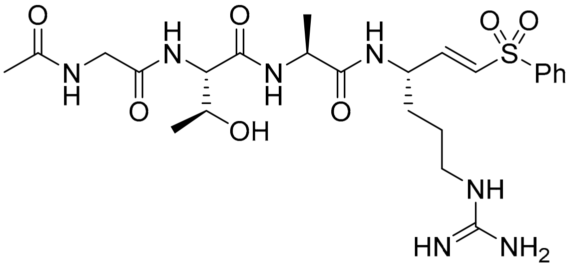

The crude product was used without further purification for the following deprotection of the Pbf- and O^{t}Bu-protecting group. It was solved in TFA/DCM (50%, 6 mL) and stirred for 2 h at room temperature. The solution was evaporated under reduced pressure and purified preparative HPLC (18.0 mg, 0.031 mmol, 16%). ^{1}H NMR (300 MHz, DMSO-*d*$_6$): δ/ppm = 8.27 – 8.15 (m, 1H), 8.00 (dd, *J* = 7.8, 3.3 Hz, 2H), 7.86 – 7.60 (m, 7H), 6.84 (d, *J* = 3.3 Hz, 1H), 6.81 – 6.65 (m, 1H), 4.57 – 4.45 (m, 1H), 4.23 – 3.66 (m, 5H), 3.13 – 2.99 (m, 2H), 1.64 – 1.37 (m, 4H), 1.22 (dd, *J* = 7.2, 4.9 Hz, 2H), 1.01 (dd, *J* = 6.3, 1.3 Hz, 3H). ^{13}C NMR (75 MHz, DMSO-*d*$_6$): δ/ppm = 172.1, 170.2, 170.1, 169.9, 169.6, 156.8, 146.5, 140.2, 133.8, 130.1, 129.7, 127.1, 66.6, 59.0, 58.3, 48.6, 42.4, 42.2, 30.1, 25.0, 22.4, 19.7, 17.8, 17.6. $[\alpha]_D^{20}$ = +13 (*c* 1.00, DMSO). FT-IR: ν/cm^{-1} = 675, 709, 734, 796, 1130, 1341, 1445, 1540, 1636, 3283, MS (ESI) *m/z* calculated for $[C_{24}H_{38}N_7O_7S]^+$ ($[M+H]^+$): 568.7, found 568.2. Purity: 100%.

107, (2*S*,3*S*)-2-(2-acetamidoacetamido)-*N*-((*S*)-1-(((*S,E*)-1-fluoro-6-guanidino-1-(phenylsulfonyl)hex-1-en-3-yl)amino)-1-oxopropan-2-yl)-3-hydroxybutanamide

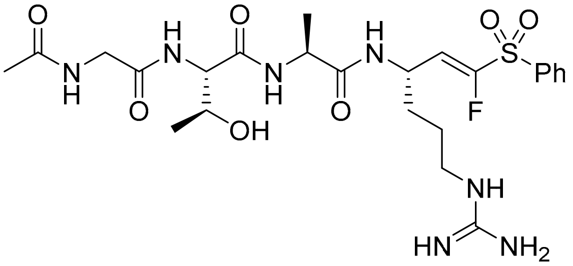

The crude product was used without further purification for the following deprotection of the Pbf- and O^{t}Bu-protecting group. It was solved in TFA/DCM (50%, 2 mL) and stirred for 2 h at rt. The solution was evaporated under reduced pressure and purified by preparative HPLC (5.6 mg, 0.021 mmol, 6%). ^{1}H NMR (600 MHz, DMSO-*d*$_6$): δ/ppm = 8.25 – 8.19 (m, 1H), 8.09 – 8.01 (m, 1H), 7.94 – 7.90 (m, 2H), 7.86 (td, *J* = 7.6, 3.8 Hz, 1H), 7.76 – 7.71 (m, 2H), 7.54 (t, *J* = 5.8 Hz, 1H), 7.34 (s, 1H), 6.83 (s, 2H), 6.32 (ddd, *J* = 36.4, 33.6, 9.1 Hz, 1H), 5.11 (s, 1H), 4.63 – 4.51 (m, 1H), 4.21 – 4.05 (m, 2H), 4.02 – 3.88 (m, 1H), 3.86 – 3.68 (m, 2H), 3.07 – 3.00 (m, 2H), 1.86 (d, *J* = 11.5 Hz, 2H), 1.58 – 1.29 (m, 4H), 1.23 – 1.17 (m, 3H), 1.05 (t, *J* = 6.3 Hz, 3H). ^{13}C NMR (151 MHz, DMSO-*d*$_6$): δ/ppm = 171.9, 171.7, 170.12, 170.0, 169.9, 169.8, 169.7, 169.6, 158.3, 158.1, 156.6, 136.4, 136.3, 135.4, 135.3, 130.3, 130.2, 128.2, 128.2, 118.7, 66.5, 66.4, 59.2, 58.1, 48.5, 43.8, 43.9, 43.8, 42.3, 42.0, 30.6, 30.5, 24.8, 24.8, 22.4, 22.4, 19.7, 19.6, 17.7, 17.6. $[\alpha]_D^{20}$ = +27 (*c* 1.00, DMSO). FT-IR: ν/cm^{-1} = 680, 713, 740, 781, 1145, 1335, 1444, 1561, 1640, 3260. MS (ESI) *m/z* calculated for $[C_{24}H_{36}FN_7O_7SNa]^+$ ($[M+Na]^+$): 586.7, found 586.2. Purity: 98%.

Synthesis of reactivity test compounds

108, *tert*-Butyl ((2S)-4-methyl-1-oxo-1-((2-oxo-1-phenylazetidin-3-yl) amino) pentan-2-yl) carbamate

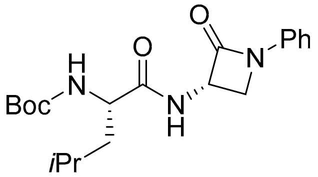

Boc-Leu-OH **50** (0.13 g, 0.67 mmol, 1.0 eq) was dissolved in a mixture of DCM/DMF (9:1) at 0 °C. Under stirring HATU (0.31 g, 0.80 mmol, 1.2 eq) was added in portions. Afterwards 2,4,6- collidine (0.24 g, 2.0 mmol, 3.0 eq) was added and stirred for an additional 10 min at 0 °C. A solution of **9** (0.14 g, 0.67 mmol, 1 eq) in DCM (5 ml) was added dropwise and the reaction mixture was allowed to reach room temperature and was stirred overnight. H_2O (30 mL) was added, and the organic phase separated. The aqueous phase was extracted with EtOAc (3 × 20 mL). The combined organic extracts were washed with aq. sat. NaCl-solution (40 mL) and dried over Na_2SO_4. The solvent was evaporated under reduced pressure to give a crude product that was purified with column chromatography (EA:Cy = 7:4) yielding the title compound as an off-white solid (0.2 g, 0.54 mmol, 81%). ^{1}H NMR (300 MHz, $CDCl_3$): δ/ppm = 7.65 – 7.45 (m, 1H), 7.38 – 7.20 (m, 4H), 7.06 (tdd, J = 5.9, 4.6, 3.0 Hz, 1H), 5.18 – 4.99 (m, 2H), 3.88 (t, J = 5.7 Hz, 1H), 3.51 (dd, J = 5.9, 2.7 Hz, 1H), 1.75 – 1.44 (m, 3H), 1.39 (s, 10H), 0.90 (dd, J = 6.3, 3.8 Hz, 6H). ^{13}C NMR (75 MHz, $CDCl_3$): δ/ppm = 173.6, 163.8, 156.0, 137.8, 129.3, 124.5, 116.8, 80.3, 65.4, 55.4, 53.1, 47.4, 41.6, 28.4, 24.8, 23.1, 21.9, 14.2. mp: 84 – 85 °C. $[\alpha]_D^{20} = -16$ (c 1.00, $CHCl_3$). MS (ESI) m/z calculated for $[C_{20}H_{28}N_3O_4Na]^+$ ($[M+Na]^+$): 389.2, found: 398.2. Purity: 99%.

109, *tert*-Butyl (*S,E*)-(5-methyl-1-(phenylsulfonyl)hex-1-en-3-yl)carbamate

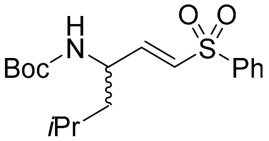

3 (0.52 g, 1.77 mmol, 1 eq) and LiCl (0.09 g, 2.21 mol, 1.25 eq) were dissolved in dry MeCN (18 mL) under argon atmosphere and cooled to 0 °C. Subsequently DBU (0.27 mL, 1.77 mmol, 1 eq) and 10 min later **52** (0.38 g, 1.77 mmol, 1 eq) in MeCN (5 mL) were added dropwise to the solution and stirred one hour at 0 °C and one hour at room temperature. The reaction mixture was quenched with citric acid solution (10%, 10 mL). The organic phase was separated, and the aqueous phase extracted with EtOAc (3x 20 mL). The combined organic extracts were dried over anhydrous Na_2SO_4 and evaporated under reduced pressure. The crude product was purified with column chromatography (CH/EA 1:6) to yield the product as colorless oil (0.18 g, 0.52 mmol, 30%). ^{1}H NMR (300 MHz, CDCl3): δ/ppm = 7.89 – 7.83 (m, 2H), 7.64 – 7.48 (m, 3H), 6.87 (dd, J = 15.0, 4.9 Hz, 1H), 6.42 (dd, J = 15.0, 1.3 Hz, 1H), 4.48 – 4.34 (m, 1H), 1.74 – 1.59 (m, 2H), 1.40 – 1.32 (m, 9H), 0.92 (d, J = 6.6 Hz, 6H). ^{13}C NMR (75 MHz, CDCl3): δ/ppm = 155.0, 147.2, 140.4, 133.5, 130.1, 129.4, 127.7, 43.4, 28.3, 24.8, 22.8, 22.1. mp: 72 –

76 °C. $[\alpha]_D^{20}$ = −7 (*c* 1.00, $CHCl_3$). FT-IR: *ν*/cm^{-1} = 731, 748, 908, 1091, 1141, 1302, 1358, 1512, 1706, 2957. MS (ESI) *m/z* calculated for $[C_{13}H_{20}NO_2S]^+$ ($[M-Boc+H]^+$): 254.12, found 254.01. Purity: 99%.

110, *tert*-Butyl (*S,E*)-(1-fluoro-5-methyl-1-(phenylsulfonyl)hex-1-en-3-yl)carbamate

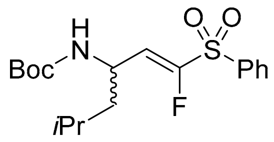

4 (0.22 g, 0.72 mmol, 1 eq) and LiCl (0.04 g, 0.90 mmol, 1.25 eq) were dissolved in dry MeCN (10 mL) under argon atmosphere and cooled to 0 °C. Subsequently DBU (0.11 mL, 0.72 mmol, 1 eq) and 10 min later **52** (0.16 g, 0.72 mmol, 1 eq) in MeCN (3 mL) were added dropwise to the solution and stirred one hour at 0 °C and one hour at room temperature. The reaction mixture was quenched with citric acid solution (10%, 10 mL). The organic phase was separated, and the aqueous phase extracted with EtOAc (3x 20 mL). The combined organic extracts were dried over anhydrous Na_2SO_4 and evaporated under reduced pressure. The crude product was purified with column chromatography (CH/EA 1:7) to desired product colorless oil (0.08 g, 0.21 mmol, 30%). ^{1}H NMR (300 MHz, $CDCl_3$): *δ*/ppm = 7.99 – 7.91 (m, 2H), 7.73 – 7.53 (m, 3H), 6.12 (d, *J* = 31.9 Hz, 1H), 4.53 (s, 1H), 1.65 – 1.45 (m, 2H), 1.35 (s, 9H), 0.90 (dd, *J* = 6.5, 2.5 Hz, 6H). ^{13}C NMR (75 MHz, $CDCl_3$): *δ*/ppm = 156.3, 154.9, 152.3, 137.3, 134.5, 129.5, 128.7, 119.1, 43.4, 28.3, 24.8, 22.5, 22.3. $[\alpha]_D^{20}$ = +26 (*c* 1.00, $CHCl_3$). FT-IR: *ν*/cm^{-1} = 683, 717, 756, 1080, 1153, 1335, 1448, 1507, 1703, 2965. MS (ESI) *m/z* calculated for $[C_{18}H_{26}FNO_4SNa]^+$ ($[M+Na]^+$): 394.1, found 394.1. Purity: 98%.

111, *tert*-Butyl ((3*S*)-2-hydroxy-5-methyl-1-nitrohexan-3-yl) carbamate

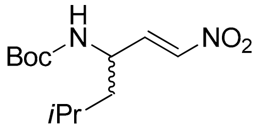

52 (0.39 g, 1.84 mmol, 1 eq) was dissolved in DCM (4 ml) and cooled to 0 °C. $MeNO_2$ (0.67 g, 11.06 mmol, 6 eq) and DBU (0.27 mL, 1.84 mmol, 1 eq) were added and the reaction mixture stirred for three hours at room temperature. The reaction got quenched with saturated NH_4Cl solution (6 mL) and extracted with DCM (15 mL). The organic solution was washed with brine (13 mL) and dried over anhydrous Na_2SO_4. The solvent was evaporated under reduced pressure and the crude product was used without further purification. The crude nitroaldol intermediate (0.44 g, 1.59 mmol, 1 eq) was dissolved in DCM (9 ml) and cooled to 0 °C. MsCl (0.36 g, 3.18 mmol, 2 eq) and DIPEA (1.11 mL, 6.36 mmol, 4 eq) were added and the reaction mixture stirred for 90 min at 0 °C. The reaction was quenched with saturated NH_4Cl solution (14 mL) and extracted with DCM (15 mL). The organic solution was washed with brine (20 mL) and dried over anhydrous Na_2SO_4. The solvent was evaporated under reduced pressure and the crude product was purified by reversed phase flash chromatography (H_2O/MeCN, gradient) to yield the desired product colorless solid (0.04 g, 0.16 mmol, 10%). ^{1}H NMR (300 MHz, $CDCl_3$): *δ*/ppm = 7.12 (dd, *J* = 13.3, 5.3 Hz, 1H), 7.04 (dd, *J* = 13.3, 0.8 Hz, 1H), 4.47 – 4.36

(m, 0H), 1.77 – 1.62 (m, 1H), 1.46 – 1.41 (m, 11H), 0.95 (dd, J = 6.6, 1.0 Hz, 6H). ^{13}C NMR (75 MHz, CDCl3): δ/ppm = 155.0, 142.7, 139.7, 80.5, 43.4, 28.4, 24.8, 22.7, 22.1. $[\alpha]_D^{20}$ = +11 (*c* 1.00, $CHCl_3$). FT-IR: ν/cm^{-1} = 2963, 1698, 1560, 1507, 1358, 1231, 1161, 1049, 849, 756. Purity: 99%.

112, Ethyl (*S,E*)-5-((*tert*-butoxycarbonyl)amino)-7-methyl-4-oxooct-2-enoate

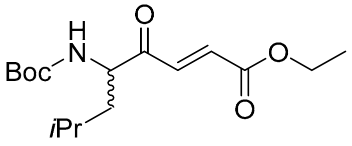

A solution of **65** (926 mg, 2.74 mmol, 1 eq) and LiCl (140 mg, 3.29 mmol, 1.2 eq) in dry MeCN (20 ml) was cooled to 0 °C. DBU (410 µL, 2.74 mmol, 1 eq) was added, followed by the dropwise addition of a solution of freshly distilled ethyl glyoxylate (559 µL, 5.49 mmol, 2 eq) in dry MeCN (5 ml), while stirring at 0 °C. After stirring for two hours at 0 °C, hydrochloric acid (0.1 M, 30 ml) was added and stirring was continued at room temperature for 15 min, followed by extraction with a mixture of chloroform an isopropyl alcohol (3:1, 4x 15ml). The combined organic layers were dried over anhydrous Na_2SO_4 and the solvent was removed under reduced pressure. The resulting residue was purified by reversed phase flash chromatography (H_2O/MeCN, gradient) yielding the desired product as a colorless resin (368 mg, 1.23 mmol, 45%). ^{1}H NMR (300 MHz, Chloroform-*d*): δ/ppm = 7.18 (d, J = 15.8 Hz, 1H), 6.78 (d, J = 15.8 Hz, 1H), 5.06 (d, J = 8.2 Hz, 1H), 4.65 – 4.47 (m, 1H), 4.24 (q, J = 7.1 Hz, 2H), 1.81 – 1.64 (m, 1H), 1.60 – 1.48 (m, 1H), 1.41 (s, 9H), 1.40 – 1.33 (m, 1H), 1.30 (t, J = 7.1 Hz, 3H), 0.94 (dd, J = 16.2, 6.5 Hz, 6H). ^{13}C NMR (75 MHz, $CDCl_3$): δ/ppm = 198.64, 165.20, 155.48, 136.31, 132.15, 80.00, 61.40, 57.22, 40.36, 28.23, 24.86, 23.17, 21.69, 14.06. $[\alpha]_D^{22}$ = +13 (*c* 1.00, $CHCl_3$). FT-IR: ν/cm^{-1} = 3367, 2960, 2872, 1700, 1507, 1470, 1391, 1367, 1253, 1165, 1023, 980, 871, 780. MS (ESI) *m/z* calculated for $[C_{16}H_{27}NO_5Na]^+$ ($[M+Na]^+$): 336.2, found: 336.3. Purity: 96%.

113, (*tert*-Butoxycarbonyl)-L-leucine-1-^{13}*C*

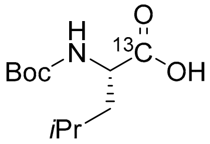

(Adapted from F. Dutton *et al.*)[30]

To a 0 °C cold solution of H-Leu-1-^{13}C-OH (0.98 g, 7.42 mmol, 1 eq) in 1,4-dioxane (5 mL) and water (3 mL) were added NaOH solution (1 M, 15 mL) and Boc_2O (3.66 g, 16.77 mmol, 2.26 eq). The solution was stirred at room temperature overnight and water (30 mL) was added. The mixture was washed with *n*-pentane (4x 80 mL) and acidified to pH = 1 with hydrochloric acid (1 M). The resulting suspension was extracted with EtOAc (4x 50 mL) and the combined organic extract were dried over anhydrous Na_2SO_4. After removing the solvent by distillation under reduced pressure, the desired product was obtained as a colorless oil (1.72 g, 7.42 mmol, quantitative). ^{1}H NMR (300 MHz, $CDCl_3$): δ/ppm = 4.89 (d, J = 8.5 Hz, 1H), 4.41–4.05 (m, 1H), 1.82–1.50 (m, 3H), 1.45 (s, 9H), 0.95 (d, J = 6.2 Hz, 6H). ^{13}C NMR (75.5 MHz, $CDCl_3$): δ/ppm = 178.3, 155.9, 80.4, 52.16 (d, J = 58.6 Hz), 41.6, 28.4, 24.9, 23.0, 21.9. $[\alpha]_D^{22}$ = −5 (*c*

1.00, $CHCl_3$). FT-IR: ν/cm^{-1} = 3446, 3333, 2959, 2940, 2870, 2561, 1684, 1533, 1392, 1366, 1237, 1161, 1048, 872, 757. MS (ESI) *m/z* calculated for $[C_{10}{}^{13}CH_{21}NO_4Na]^+$ ($[M+Na]^+$): 255.1, found: 255.0.

114, *tert*-Butyl (*S*)-(1-(methoxy(methyl)amino)-4-methyl-1-oxopentan-2-yl-1-^{13}*C*)carbamate

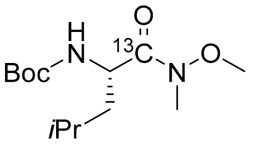

To a 0 °C cold solution of Boc-Leu-1-^{13}C-OH **113** (1.70 g, 7.32 mmol, 1 eq) in DCM (80 mL) were added HOBt · H_2O (1.12 g, 7.32 mmol, 1 eq) and 2,4,6-collidine (1.94 mL, 14.64 mmol, 2 eq). After stirring at 0 °C for 30 min, TBTU (2.35 g, 7.32 mmol, 1 eq) was added. The solution was stirred for further 30 min at 0 °C and *N*,*O*-dimethyl hydroxylamine · HCl (0.71 g, 7.32 mmol, 1 eq) was added. After stirring at room temperature overnight, the solvent was removed under reduced pressure and the residue was taken up in EtOAc (100 mL). The mixture was washed with saturated $NaHCO_3$ solution (3x 80 mL) and hydrochloric acid (1 M, 3x 80 mL) and filtered over a small silica column. The filtrate was concentrated under reduced pressure to yield the desired product as a colorless oil (1.91 g, 6.92 mmol, 95%). ^{1}H NMR (300 MHz, $CDCl_3$): δ/ppm = 5.14–4.95 (m, 1H), 4.79–4.60 (m, 1H), 3.77 (s, 3H), 3.22–3.15 (m, 3H), 1.78–1.61 (m, 1H), 1.49–1.37 (m, 11H), 0.97–0.88 (m, 6H). ^{13}C NMR (75.5 MHz, $CDCl_3$): δ/ppm = 174.0, 155.8, 79.6, 61.7, 49.10 (d, J = 54.6 Hz), 42.2, 32.3, 28.5, 24.9, 23.5, 21.0. $[\alpha]_D^{22}$ = −10 (c 1.00, $CHCl_3$). FT-IR: ν/cm^{-1} = 3321, 2957, 2935, 2870, 1708, 1619, 1499, 1365, 1250, 1165, 1045, 1017, 986, 875, 758. MS (ESI) *m/z* calculated for $[C_{12}{}^{13}CH_{26}N_2O_4Na]^+$ ($[M+Na]^+$): 298.2, found: 298.1.

115, *tert*-Butyl (1-(benzo[*d*]thiazol-2-yl)-4-methyl-1-oxopentan-2-yl-1-^{13}*C*)carbamate

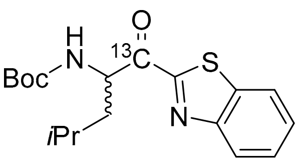

To a solution of benzothiazole (2.65 g, 19.61 mmol, 10 eq) in dry THF (50 mL) at −75 °C was added *n*-BuLi (2.5 M in hexanes, 5.5 mL, 13.73 mmol, 7 eq) dropwise over 15 min. The mixture was stirred at −75 °C for one hour and a solution of **114** (540 mg, 1.96 mmol, 1 eq) in dry THF (10 mL) was added. After stirring was continued at −75 °C for 2.5 h, saturated NH_4Cl solution (30 mL) was added. The mixture was extracted with EtOAc (3x 80 mL) and the combined organic extracts were filtered over a small silica column. The filtrate was concentrated by distillation under reduced pressure and the residue was purified by column chromatography (CH/EA 20:1) to yield the desired product as a yellowish solid (380 mg, 1.09 mmol, 56%). ^{1}H NMR (300 MHz, $CDCl_3$): δ/ppm = 8.22–8.16 (m, 1H), 8.00–7.94 (m, 1H), 7.61–7.49 (m, 2H), 5.76–5.52 (m, 1H), 5.40–5.18 (m, 1H), 1.88–1.77 (m, 2H), 1.60–1.50 (m, 1H), 1.43 (s, 9H), 1.09 (d, J = 6.0 Hz, 3H), 0.96 (d, J = 6.2 Hz, 3H). ^{13}C NMR (75.5 MHz, $CDCl_3$): δ/ppm =194.9, 164.2 (d, J = 64.8 Hz), 155.6, 153.7 (d, J = 7.8 Hz), 137.4, 128.0, 127.1,

125.9, 122.5, 80.0, 55.4 (d, J = 46.0 Hz), 42.2, 28.4, 25.4, 23.4, 21.8. mp: 108–110 °C. $[\alpha]_D^{22}$ = +40 (c 1.00, $CHCl_3$). FT-IR: ν/cm^{-1} = 3364, 3066, 2965, 2929, 2871, 1682, 1659, 1518, 1480, 1366, 1161, 876, 824, 760, 731. MS (ESI) m/z calculated for $[C_{17}{}^{13}CH_{24}N_2O_3SNa]^+$ ($[M+Na]^+$): 372.1, found: 372.0. Purity: 98%.

116, *tert*-Butyl (*S*)-(1-amino-4-methyl-1-oxopentan-2-yl-1-^{13}*C*)carbamate

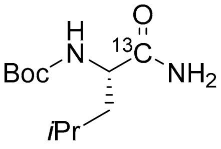

Boc-Leu-1-^{13}C-OH **113** (0.3 g, 1.5 mmol, 1.0 eq) was dissolved in DMF (10 mL) at 0 °C. Under stirring HOBt · H_2O (0.3 g, 1.9 mmol, 1.2 eq) was added in portions. After 20 min, NH_4OH solution (25% in water, 5 mL) was added, and reactions stirred overnight. After adding DCM (15 mL), a white precipitate formed, which was collected via filtration. The filtrate was washed with brine (15 mL), saturated $NaHCO_3$ solution (15 mL), dried over anhydrous N_2SO_4 and the solvent removed under reduced pressure to give a crude product that was purified with column chromatography (CH/EA 1:2) yielding the title compound as a colorless oil (51 mg, 0.22 mmol, 15%). ^{1}H NMR (300 MHz, CDCl3): δ/ppm = 6.85 – 6.50 (m, 1H), 5.42 – 5.05 (m, 1H), 4.18 (s, 1H), 1.82 – 1.57 (m, 2H), 1.42 (d, J = 4.5 Hz, 10H), 0.91 (dd, J = 6.3, 4.0 Hz, 6H). ^{13}C NMR (75.5 MHz, $CDCl_3$): δ/ppm =175.9, 155.9, 80.2, 52.4, 41.5, 28.4, 24.9, 23.1, 22.0. MS (ESI) m/z calculated for $[C_{10}{}^{13}CH_{23}N_2O_3]^+$ ($[M+H]^+$): 231.17, found: 232.22.

117, *tert*-Butyl (*S*)-(1-(cyano-^{13}*C*)-3-methylbutyl)carbamate

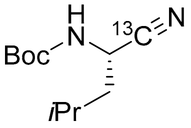

116 (50 mg, 1.5 mmol, 1.0 eq) was dissolved in dry THF (10 mL) at −78 °C and trifluoroacetic anhydride (45 µL, 0.33 mmol, 1.5 eq) and pyridine (53 µL, 0.65 mmol, 3.0 eq) were added subsequently. After stirring for 2 h, the solvent was removed in vacuum and the crude residue was diluted with EtOAc (20 mL) and washed with 1 m $KHSO_4$-solution (15 mL) and brine (15 mL). The combined organic extracts were dried over Na_2SO_4 and the solvent removed in vacuum yielding the title compound as an colorless oil (34 mg, 0.16 mmol, 74%). ^{1}H NMR (300 MHz, $CDCl_3$): δ/ppm = 1.91 – 1.75 (m, 1H), 1.74 – 1.60 (m, 2H), 1.55 – 1.43 (m, 9H), 1.04 – 0.90 (m, 6H).^{13}C NMR (75.5 MHz, $CDCl_3$): δ/ppm =172.9, 119.2, 81.5, 52.4, 42.2, 28.3, 24.9, 23.1, 22.0. Purity: 95%.

Spectra and chromatograms

13, Benzyl ((*S*)-1-(((*S,E*)-1-nitro-5-phenylpent-1-en-3-yl)amino)-1-oxo-3-phenylpropan-2-yl)carbamate

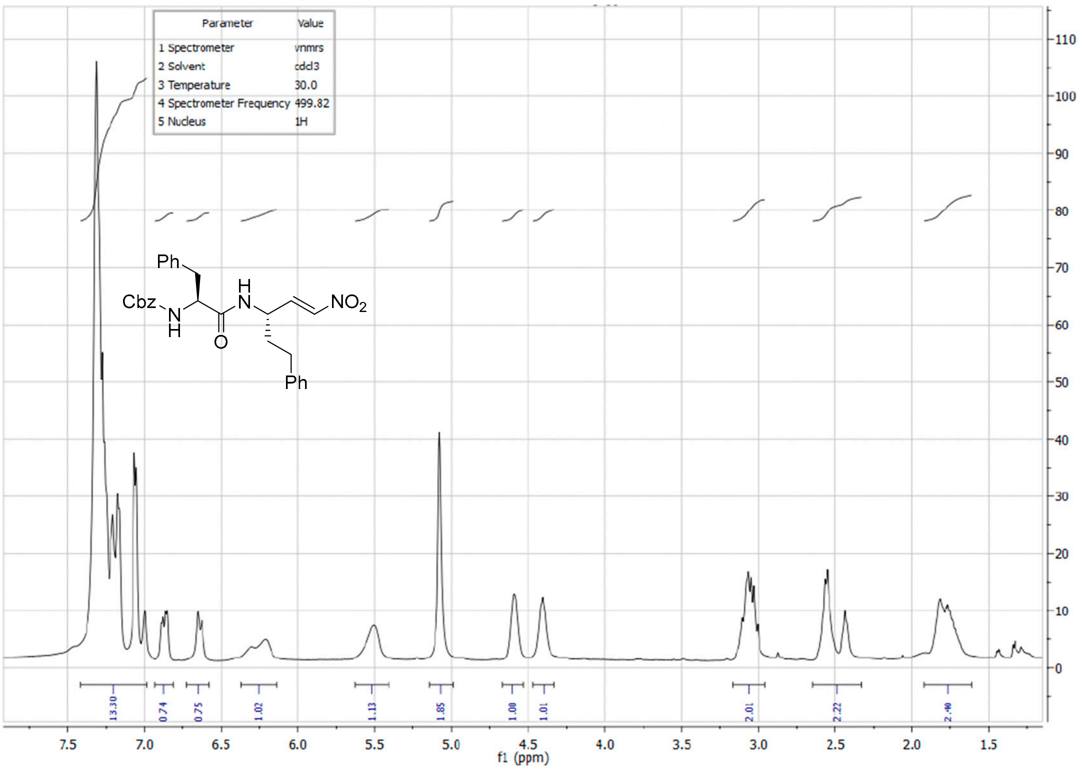

Figure S26. ^{1}H NMR of 13.

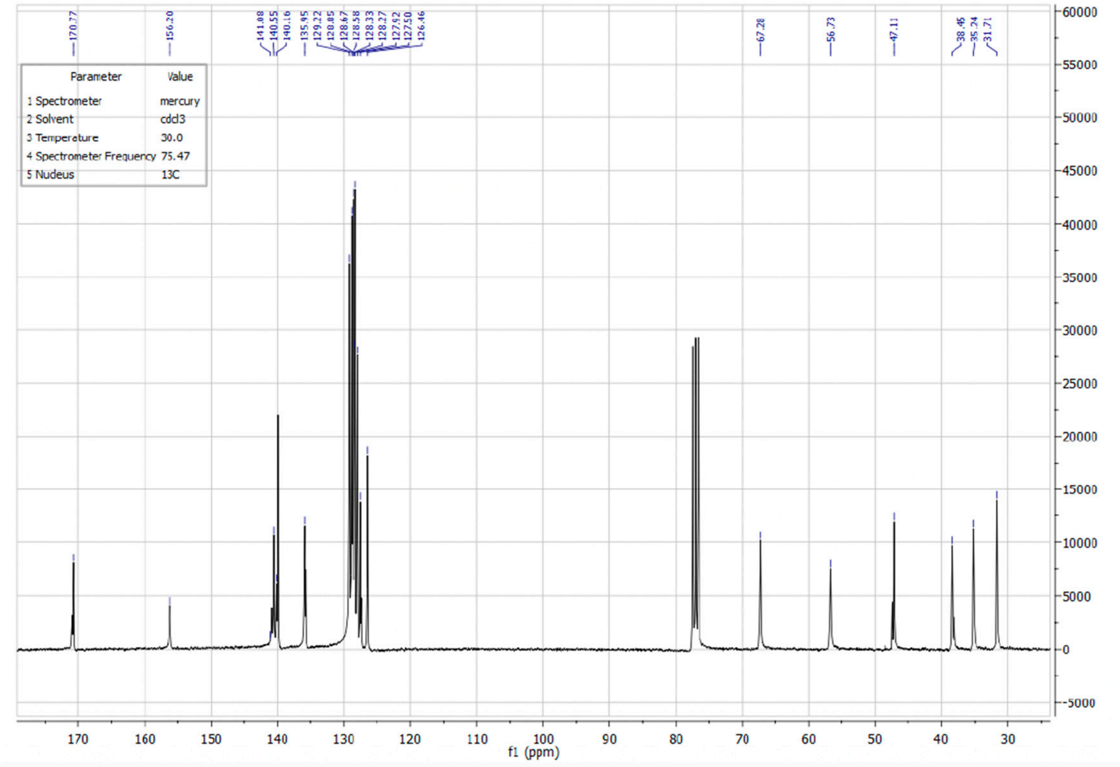

Figure S27. ^{13}C NMR of 13.

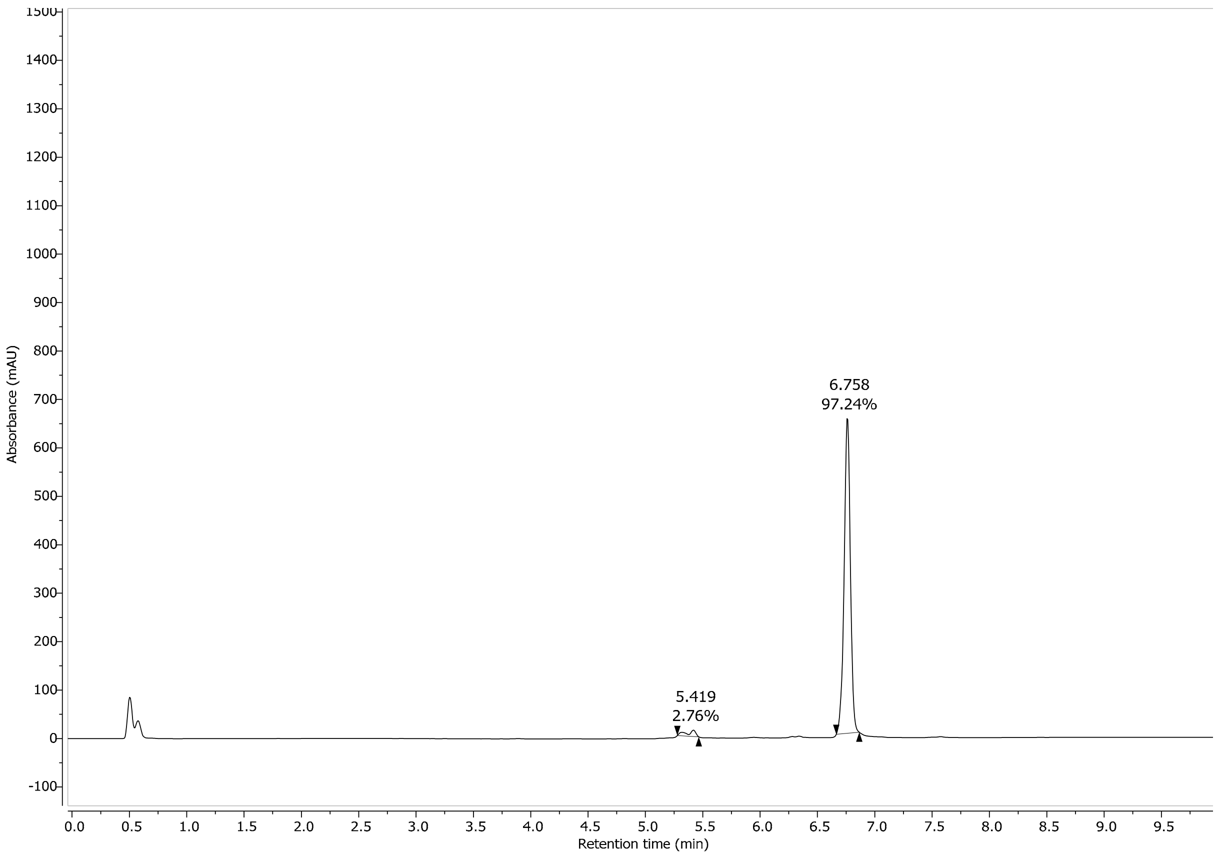

Figure S28. HPLC Chromatogram of 13 at 254 nm.

16, Benzyl ((*S*)-1-oxo-3-phenyl-1-(((*S*,*E*)-5-phenyl-1-(phenylsulfonyl)pent-1-en-3-yl)amino)propan-2-yl)carbamate

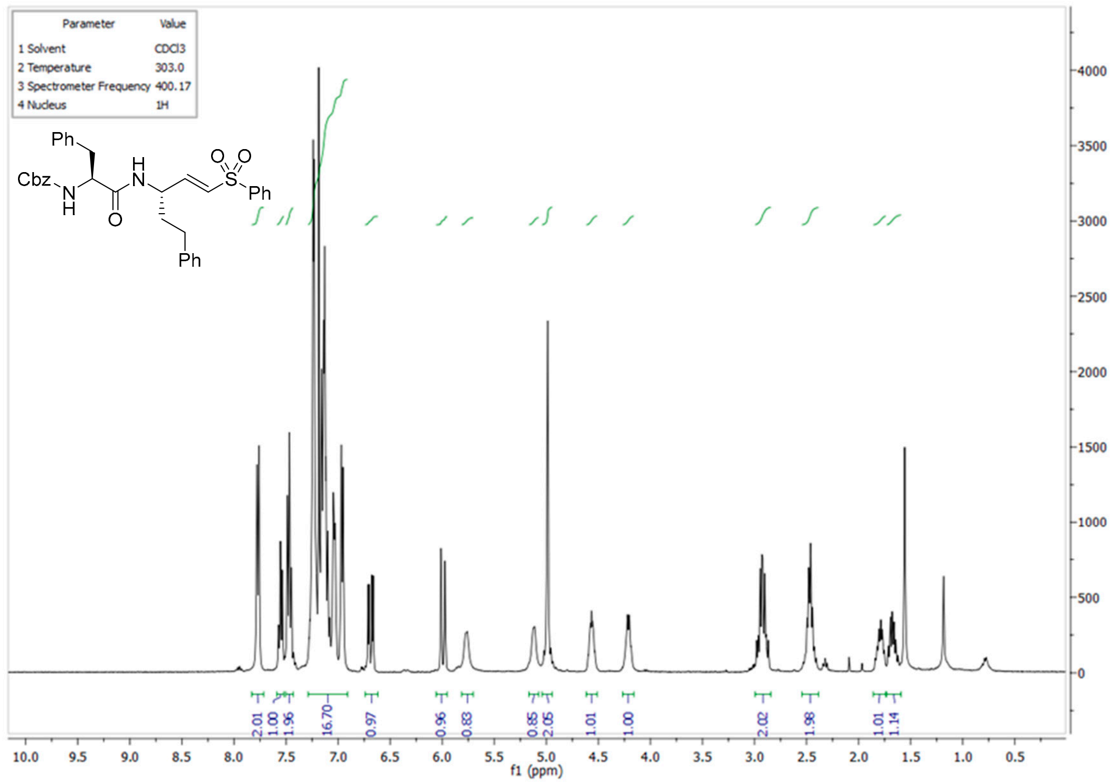

Figure S29. ^{1}H NMR of 16.

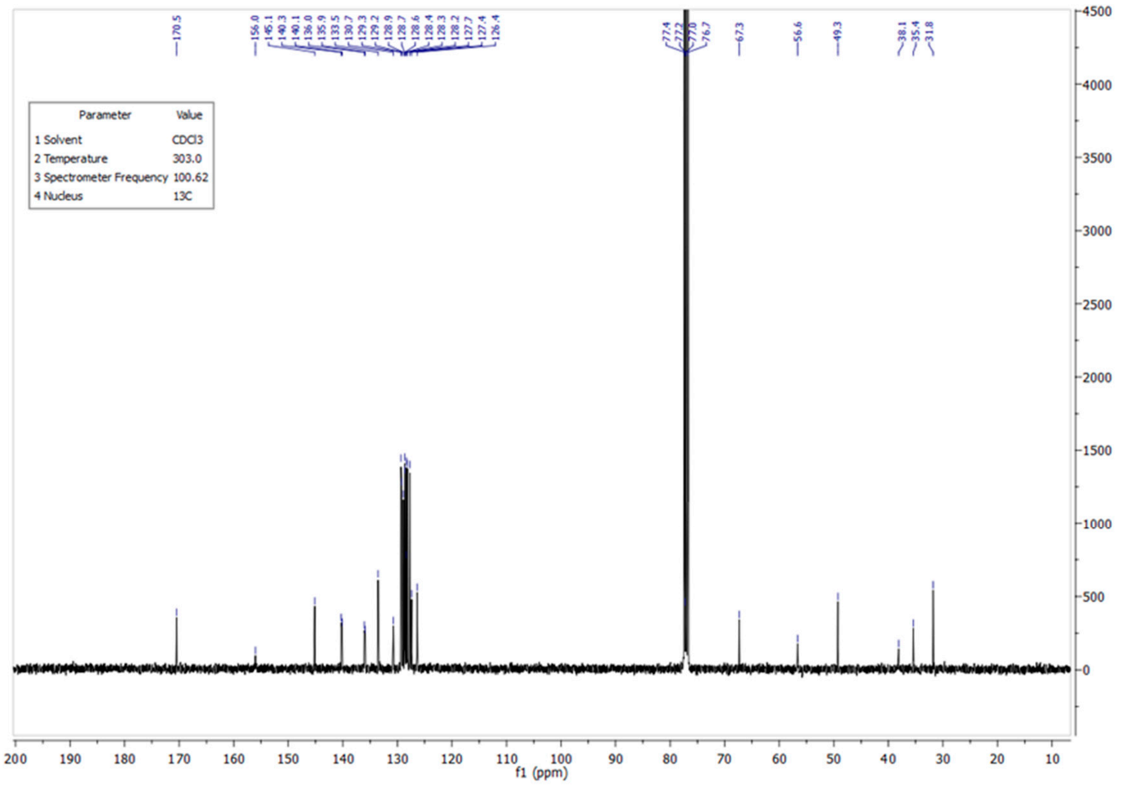

Figure S30. ^{13}C NMR of 16.

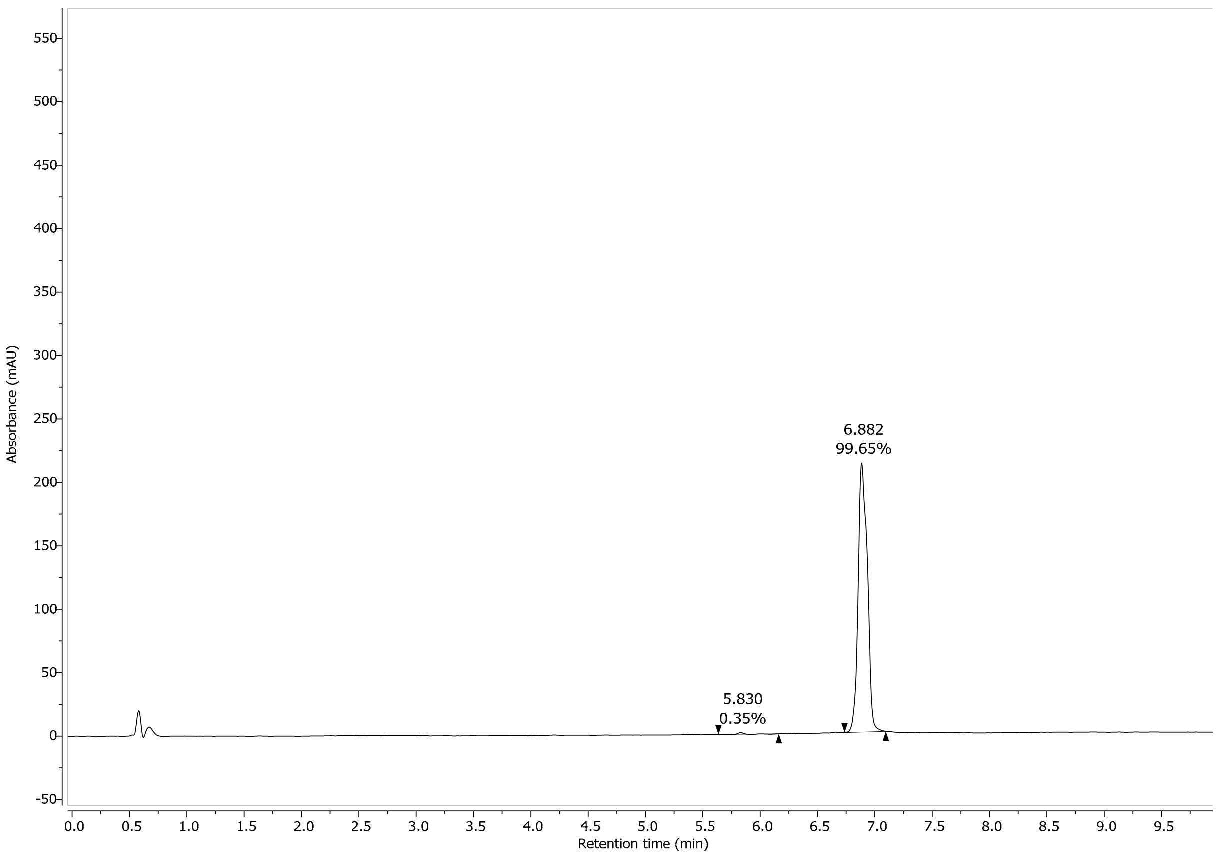

Figure S31. HPLC Chromatogram of 16 at 254 nm.

17, Benzyl ((*S*)-1-(((*S*,*E*)-1-fluoro-5-phenyl-1-(phenylsulfonyl)pent-1-en-3-yl)amino)-1-oxo-3-phenylpropan-2-yl)carbamate

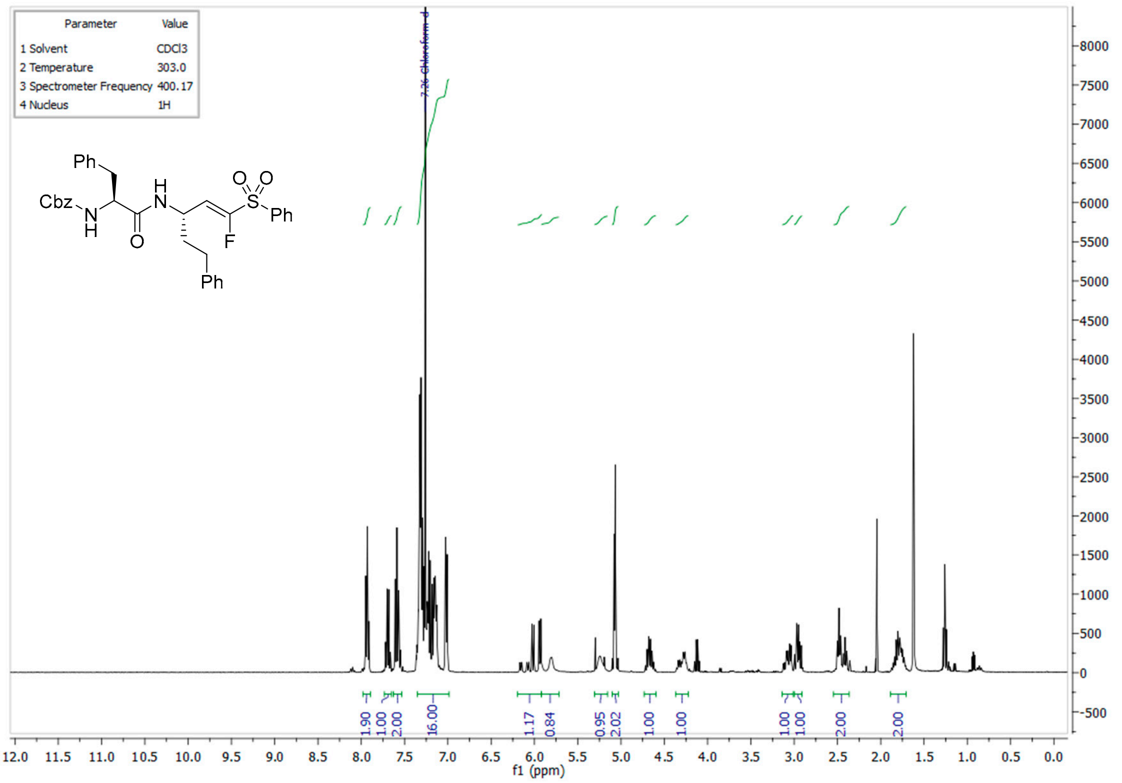

Figure S32. ^{1}H NMR of 17.

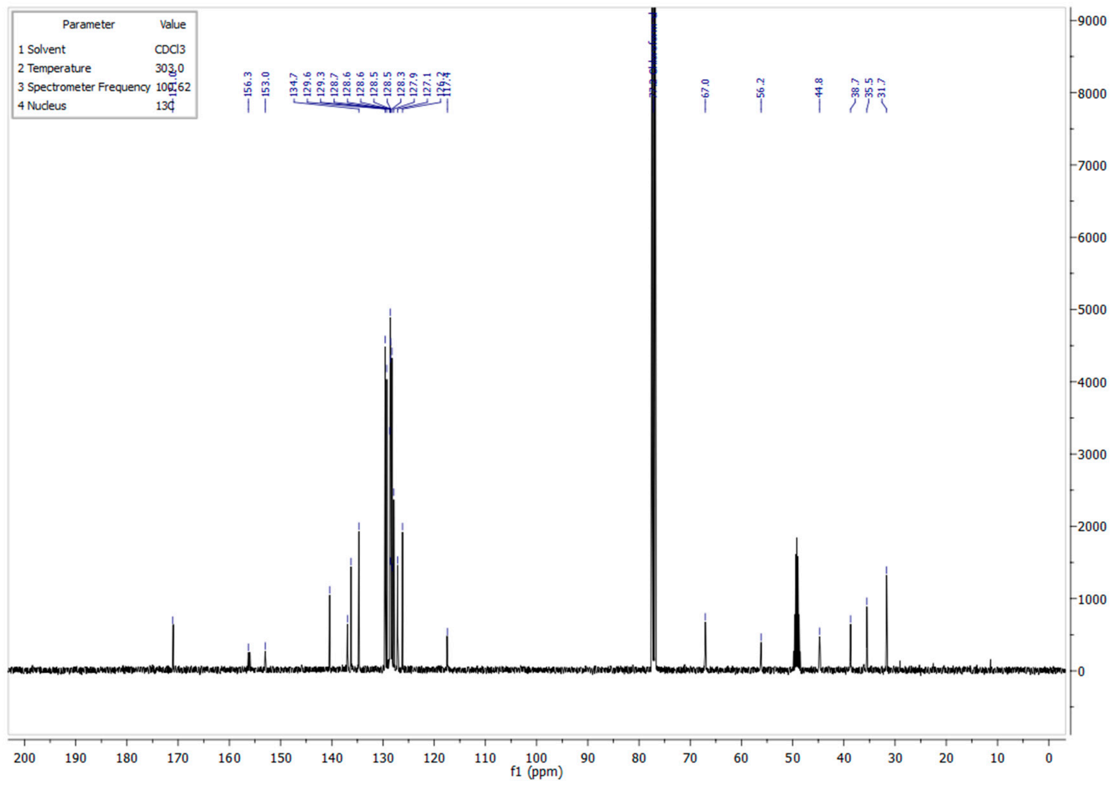

Figure S33. ^{13}C NMR of 17.

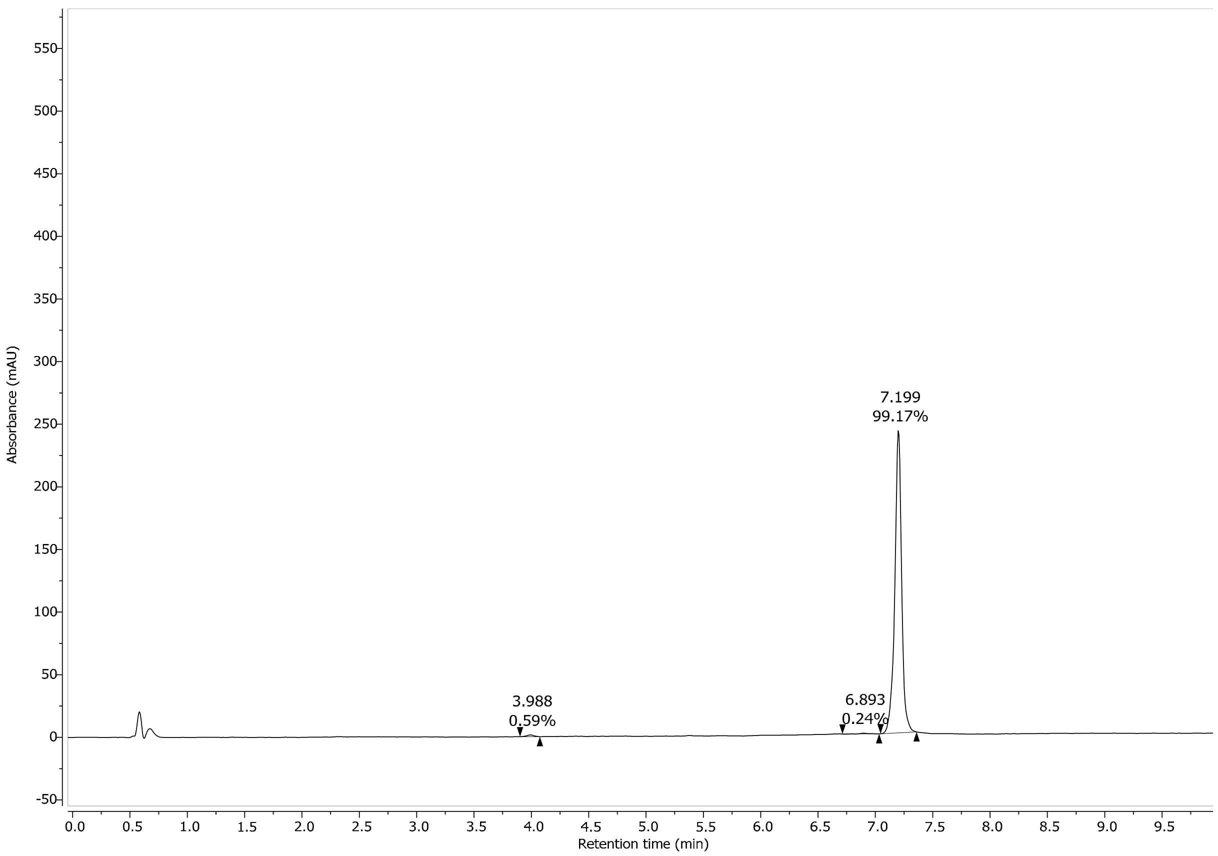

Figure S34. HPLC Chromatogram of 17 at 254 nm.

18, Benzyl ((*S*)-1-(((*S*)-1-(benzo[*d*]thiazol-2-yl)-1-oxo-4-phenylbutan-2-yl) amino)-1-oxo-3-phenylpropan-2-yl)carbamate

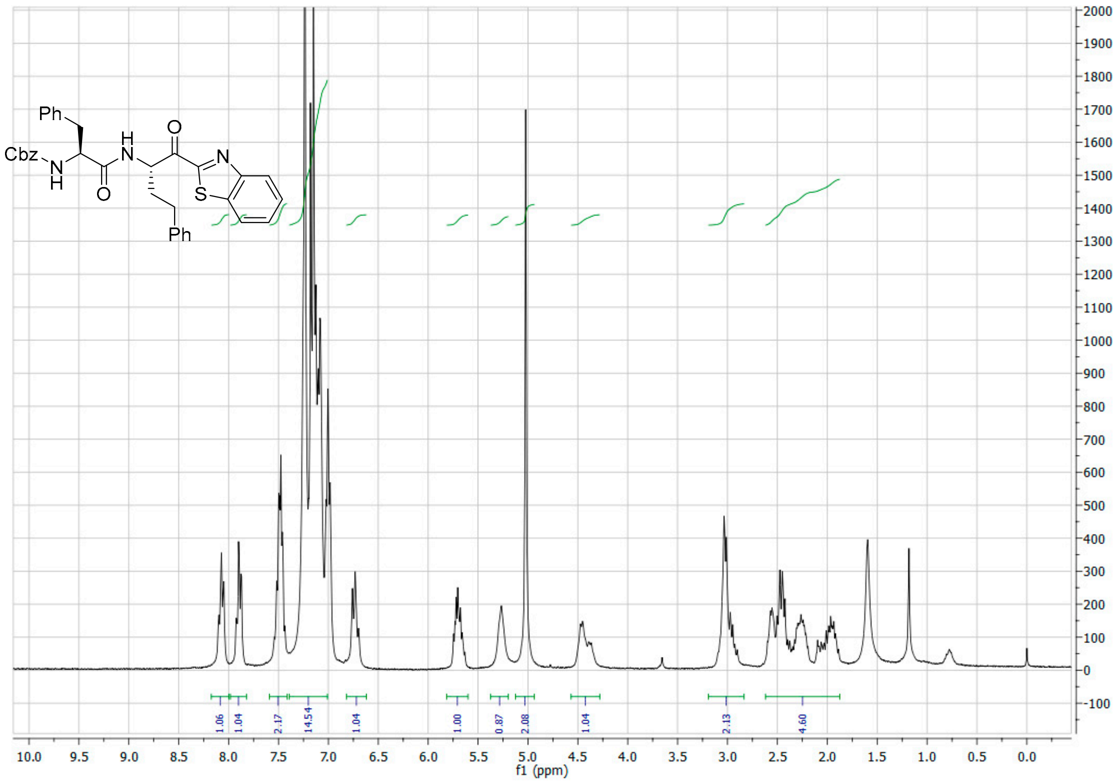

Figure S35. ^{1}H NMR of 18.

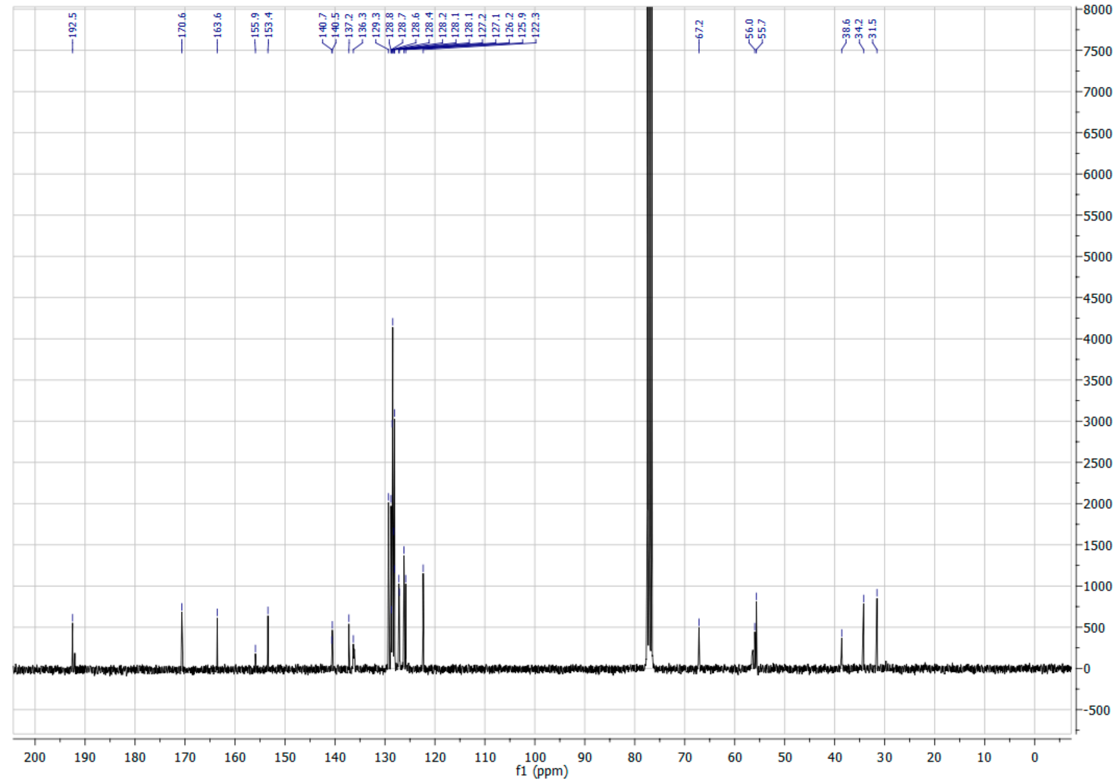

Figure S36. ^{13}C NMR of 18.

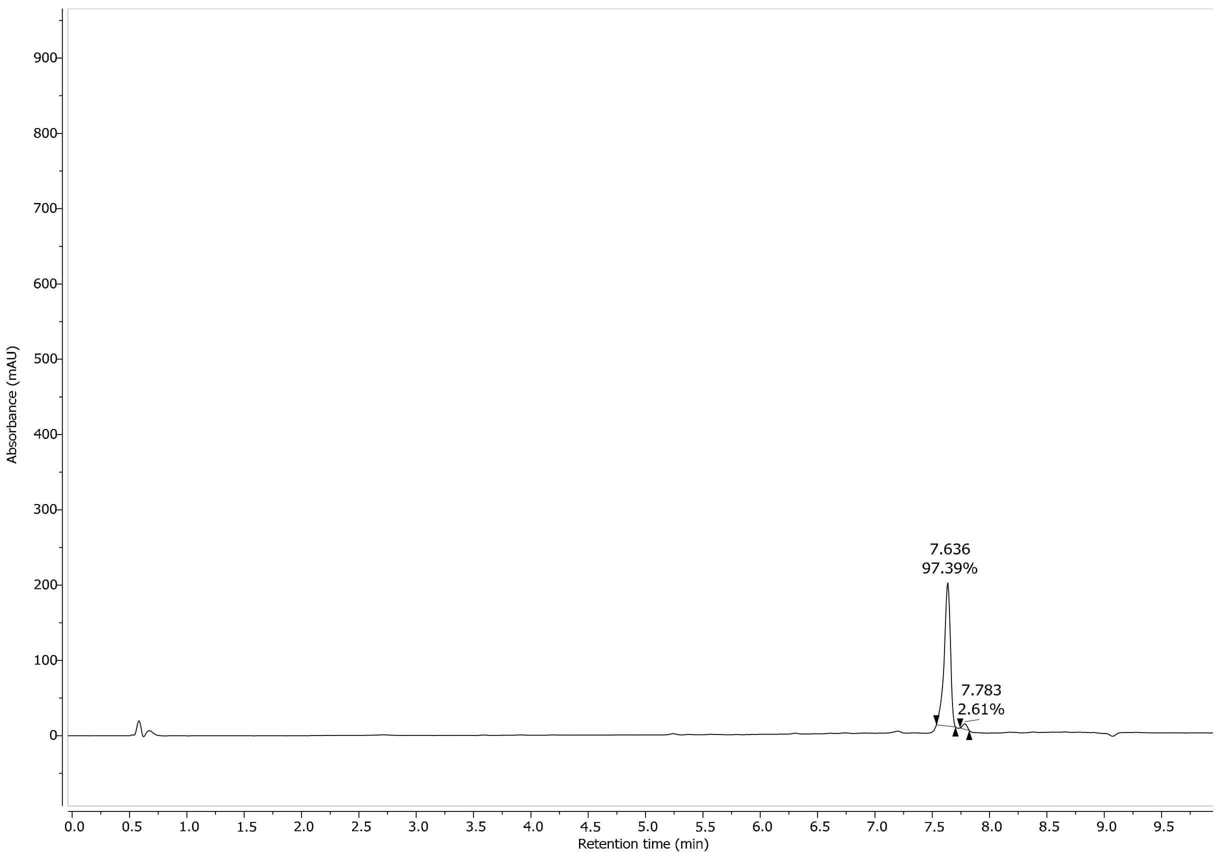

Figure S37. HPLC Chromatogram of 18 at 254 nm.

22, Ethyl (*S,E*)-5-((*S*)-2-(((benzyloxy)carbonyl)amino)-3-phenylpropanamido)-4-oxo-7-phenylhept-2-enoate

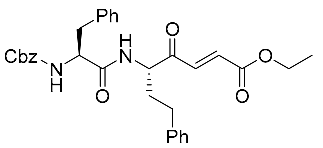

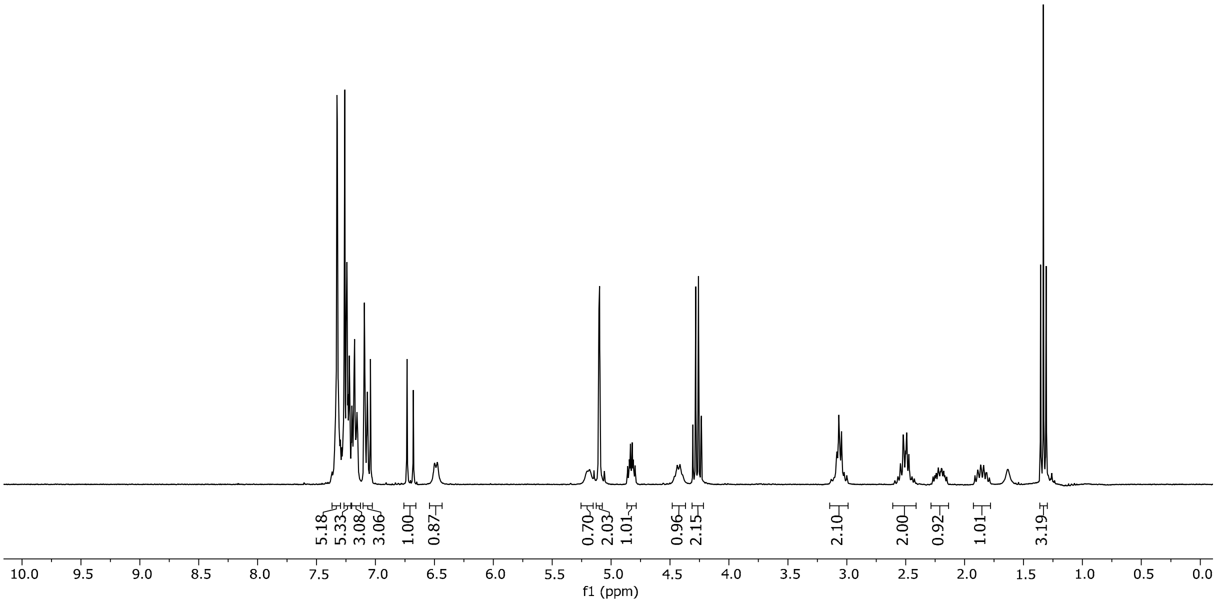

Figure S38. ^{1}H NMR of 22.

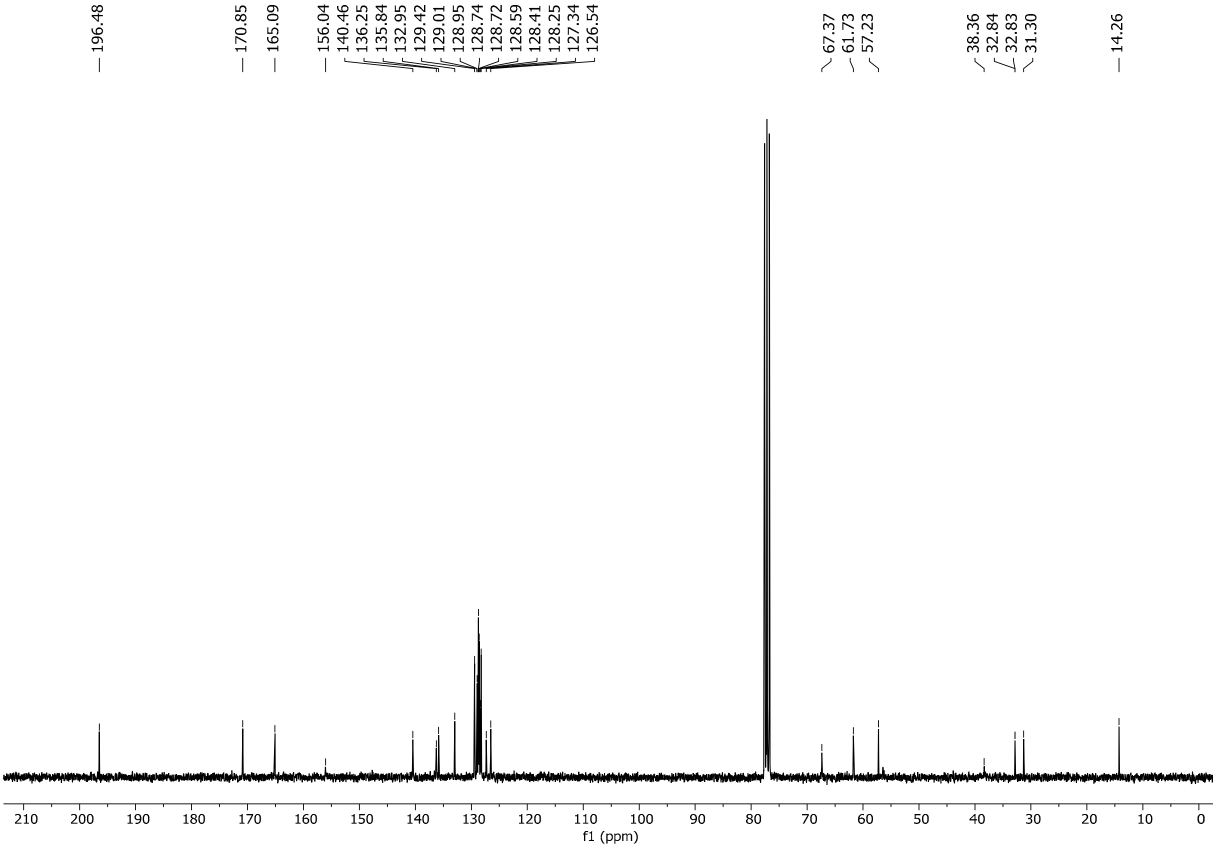

Figure S39. ^{13}C NMR of 22.

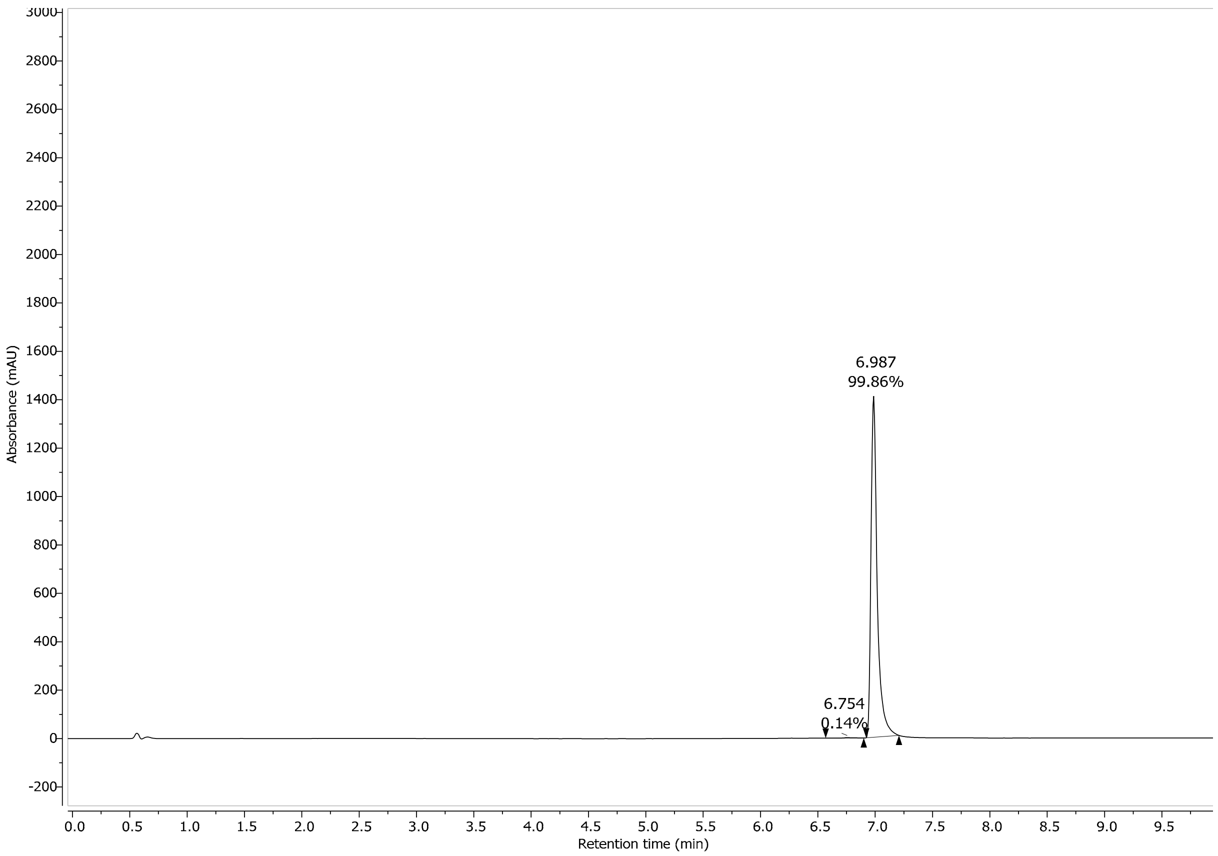

Figure S40. HPLC Chromatogram of 22 at 254 nm.

24, Benzyl ((*S*)-1-oxo-1-(((*S*)-1-oxo-1-(((*S*)-2-oxo-1-phenylazetidin-3-yl)amino)-4-phenylbutan-2-yl)amino)-3-phenylpropan-2-yl)carbamate

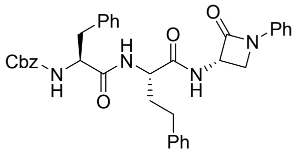

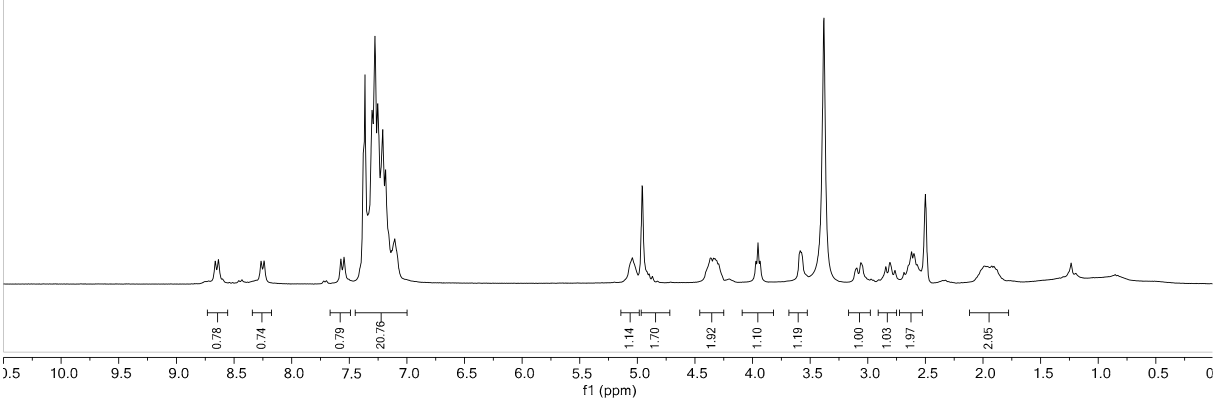

Figure S41. ^{1}H NMR of 24.

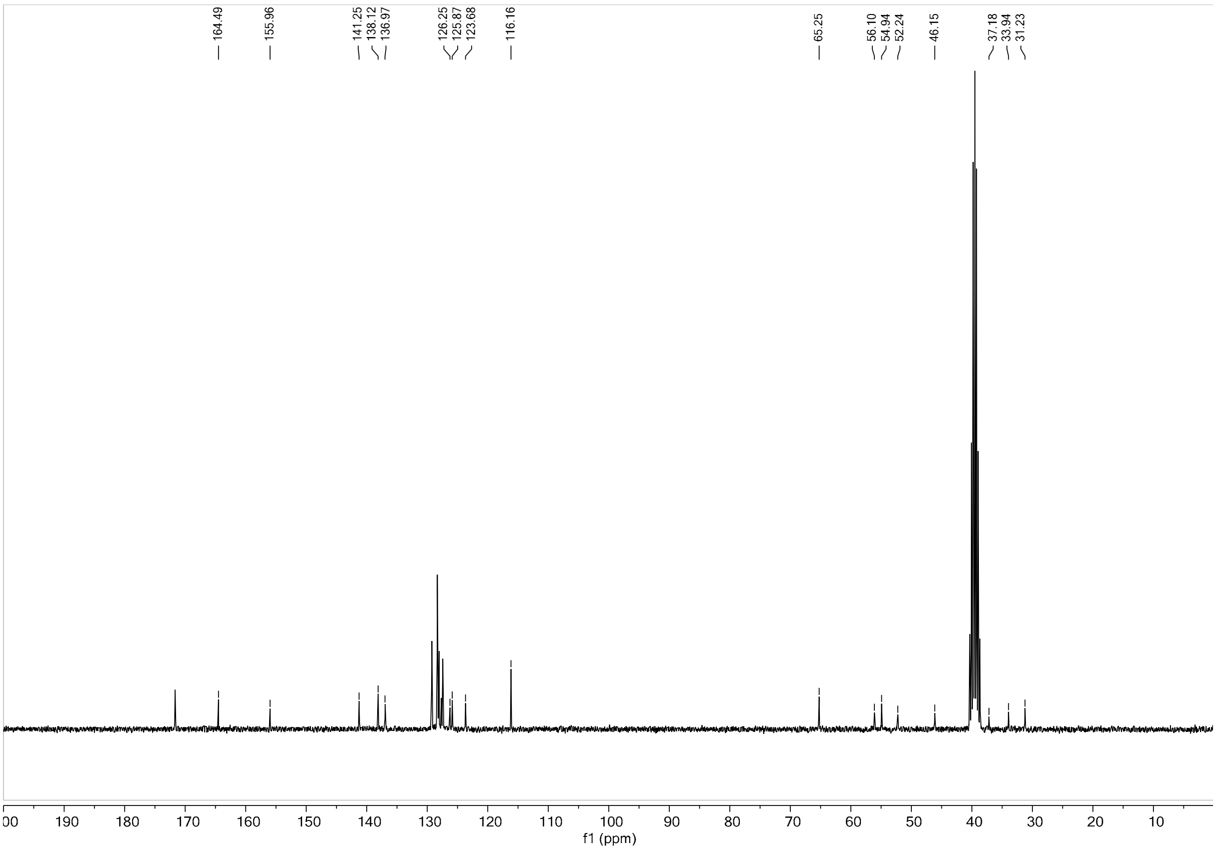

Figure S42. ^{13}C NMR of 24.

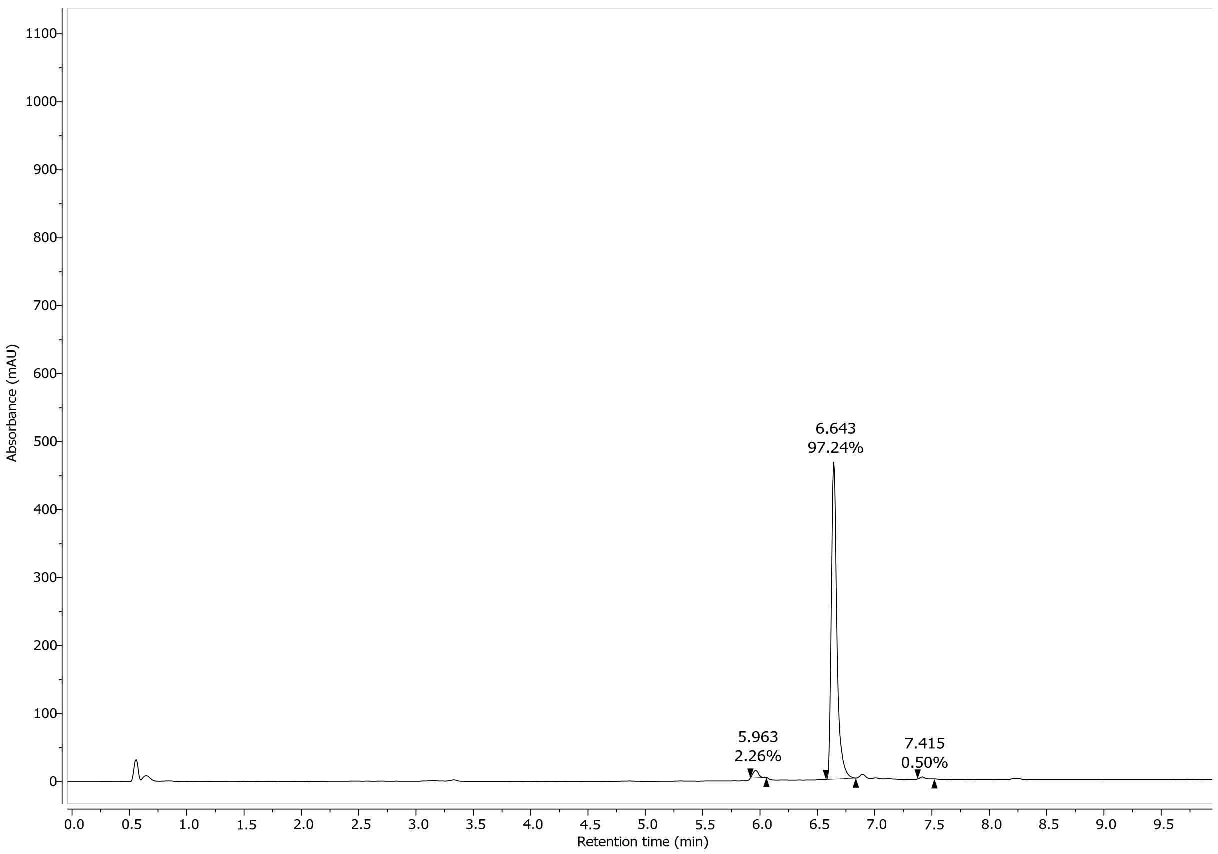

Figure S43. HPLC Chromatogram of 24 at 254 nm.

25, Benzyl ((*S*)-1-(((*S*)-1-cyano-3-phenylpropyl)amino)-1-oxo-3-phenylpropan-2-yl)carbamate

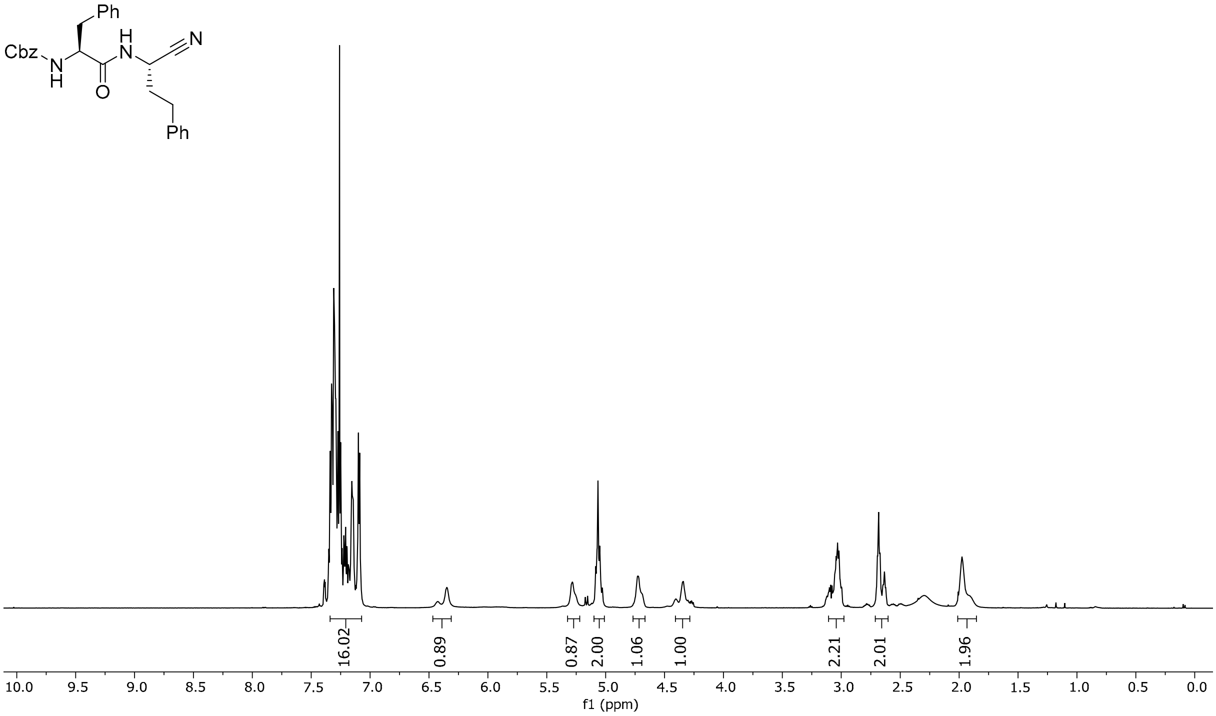

Figure S44. ^{1}H NMR of 25.

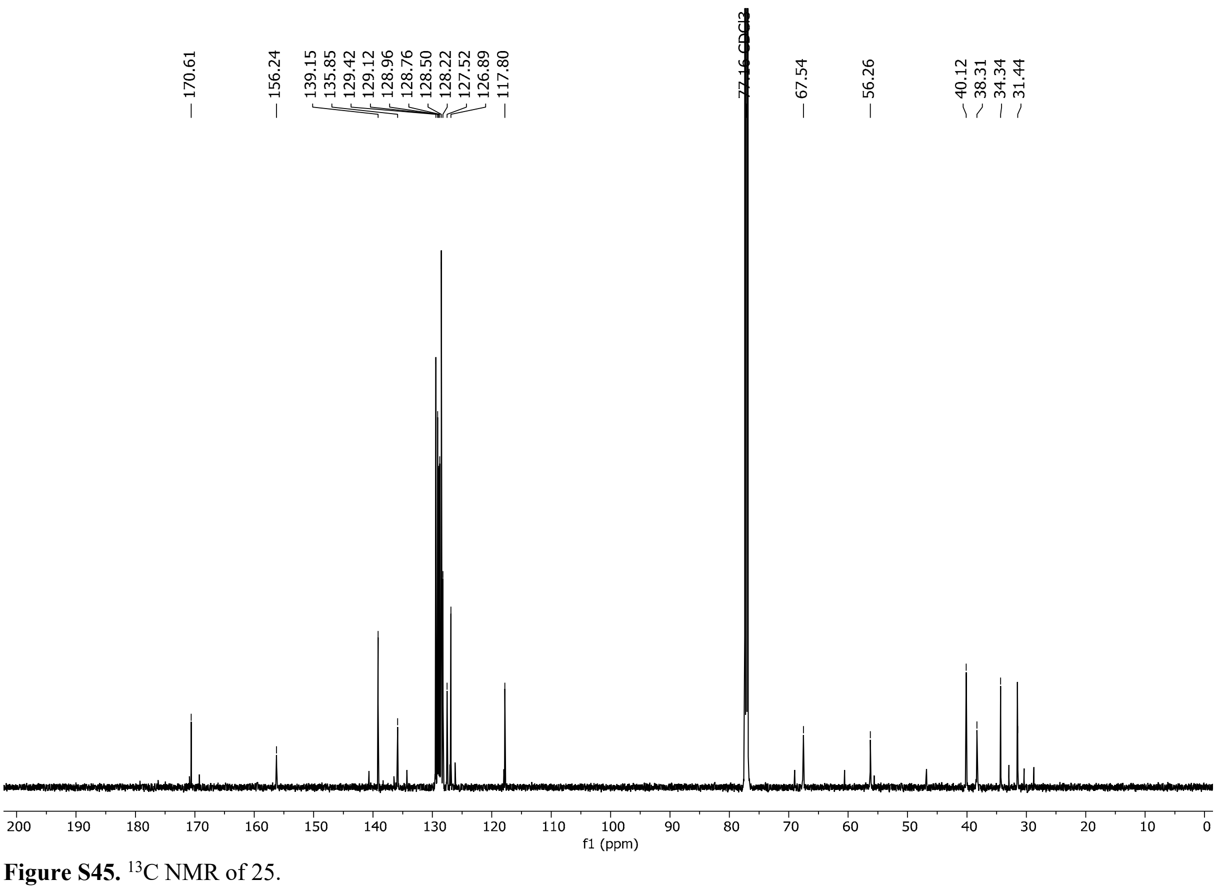

Figure S45. ^{13}C NMR of 25.

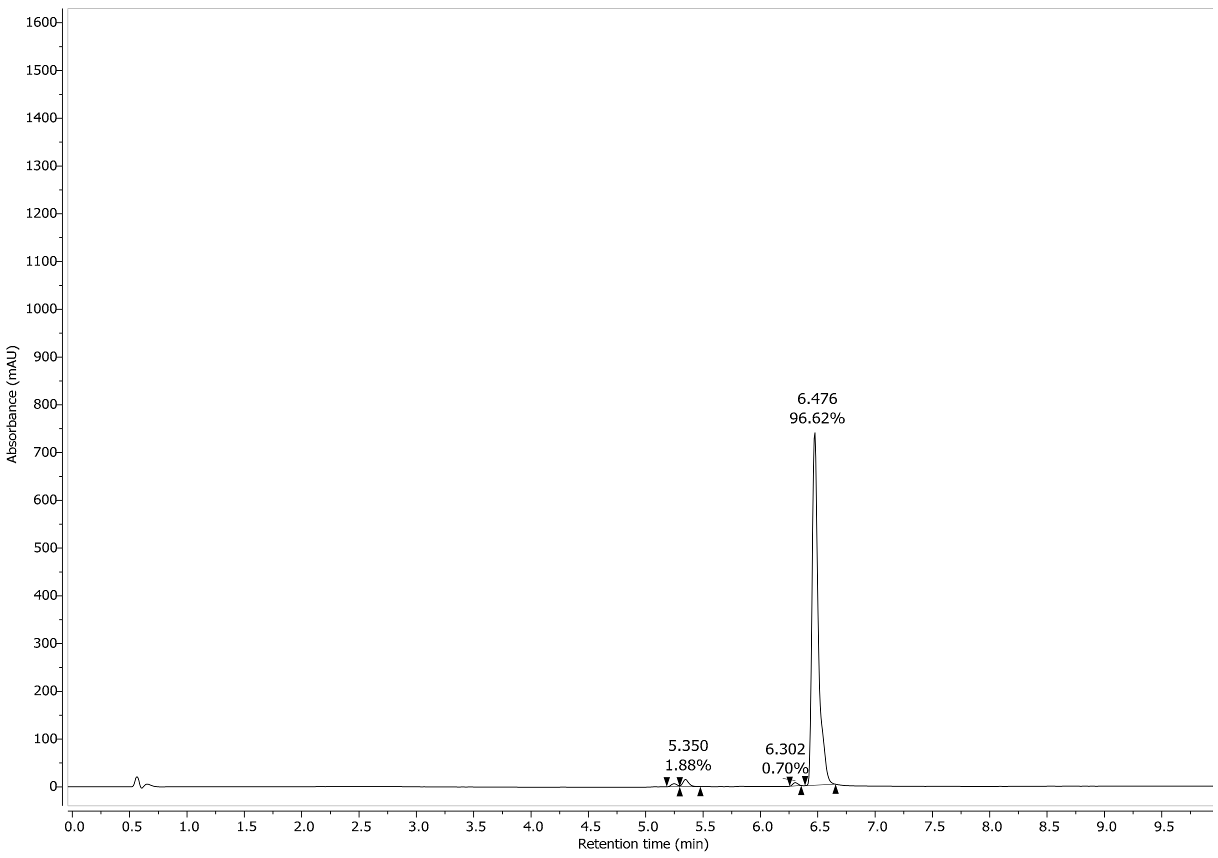

Figure S46. HPLC Chromatogram of 25 at 254 nm.

30, *N*-((*S*)-1-((*R*-2-(Benzyloxy)-1-cyanoethyl) amino)-3-cyclohexyl-1-oxopropan-2-yl) morpholine-4-carboxamideL-Cyclohexylalanine-methylester-hydrochloride

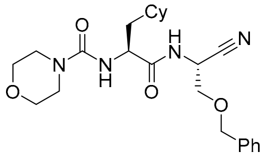

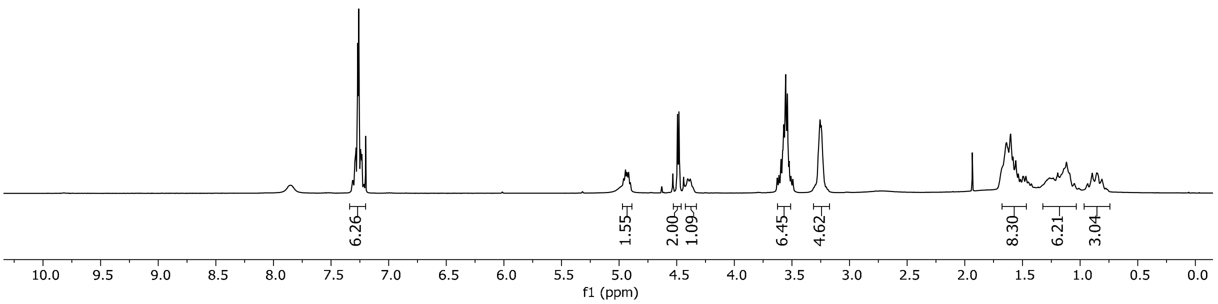

Figure S47. ^{1}H NMR of 30.

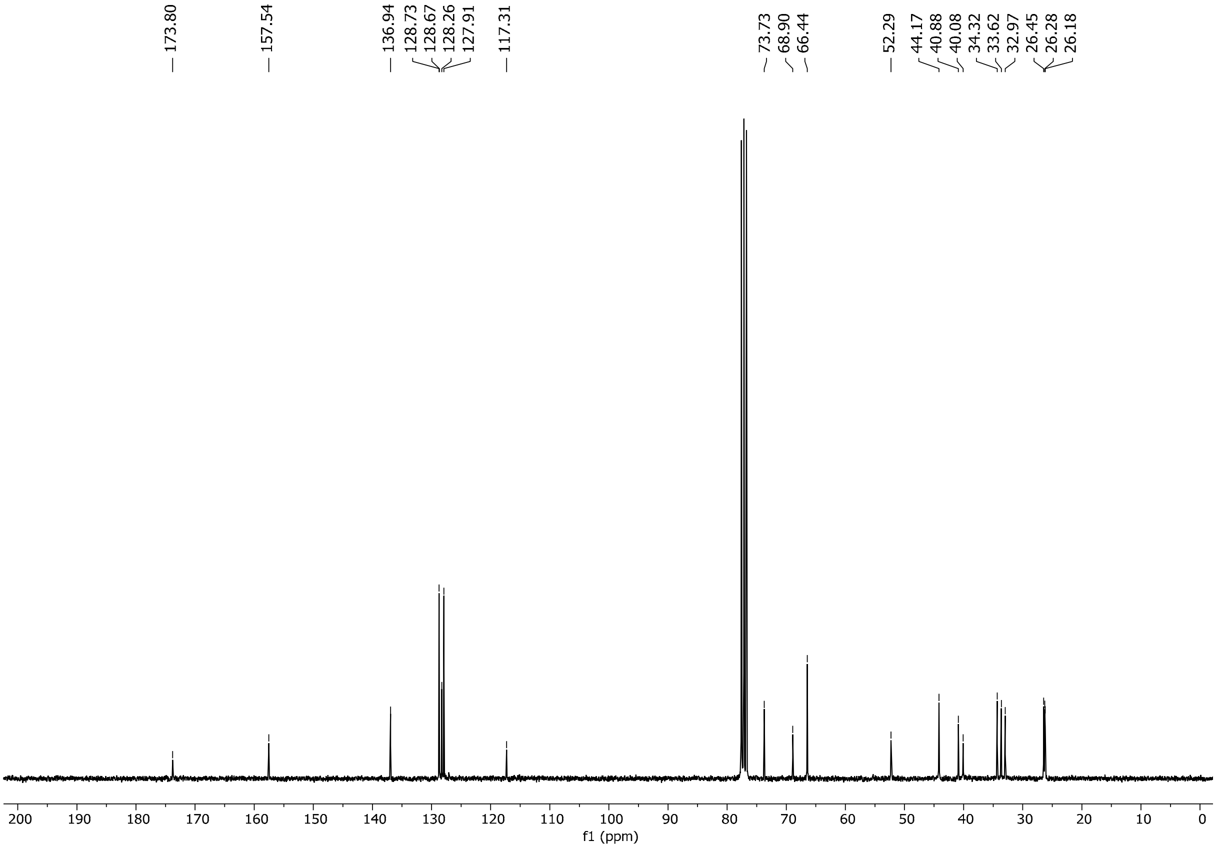

Figure S48. ^{13}C NMR of 30.

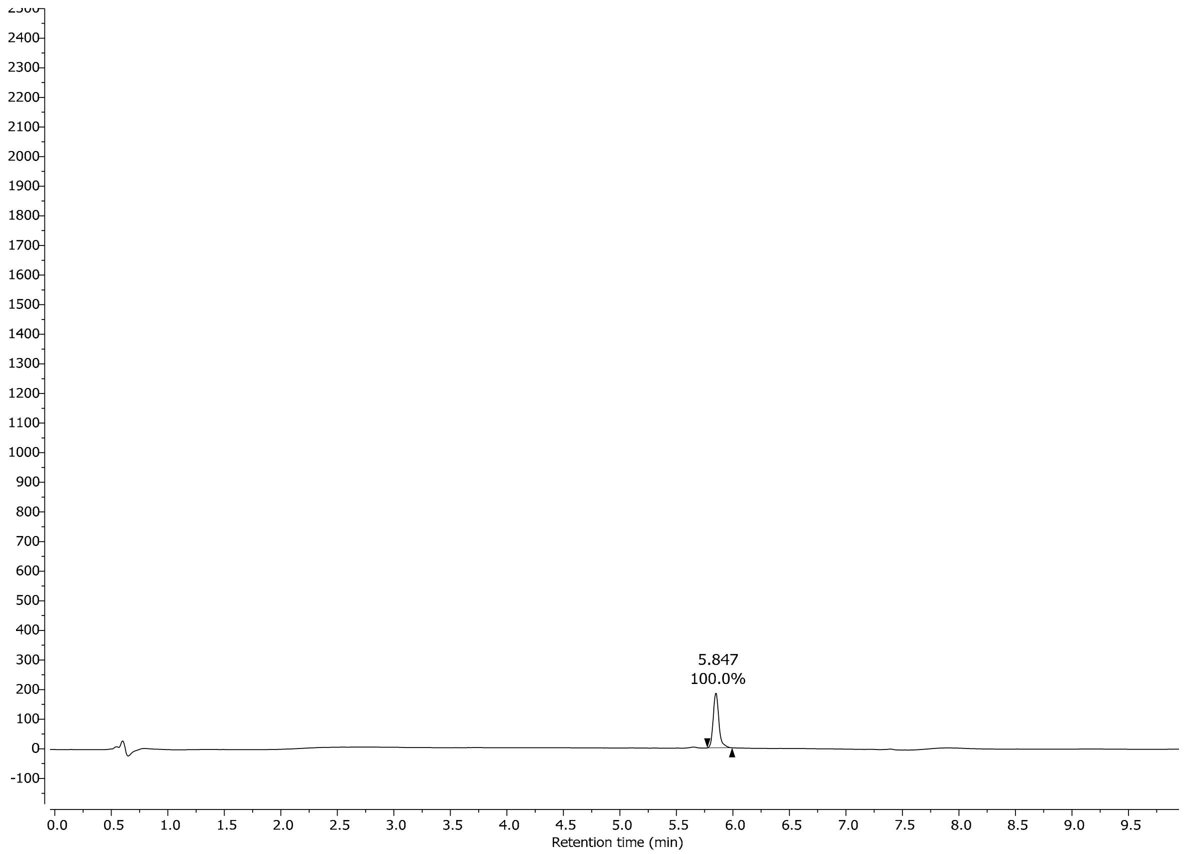

Figure S49. HPLC Chromatogram of 30 at 210 nm.

35, *N*-((*S*)-1-(((*S*,*E*)-1-(benzyloxy)-4-(phenylsulfonyl) but-3-en-2-yl) amino)-3-cyclohexyl-1-oxopropan-2-yl) morpholin-4-carboxamide

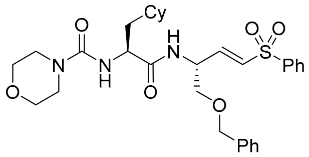

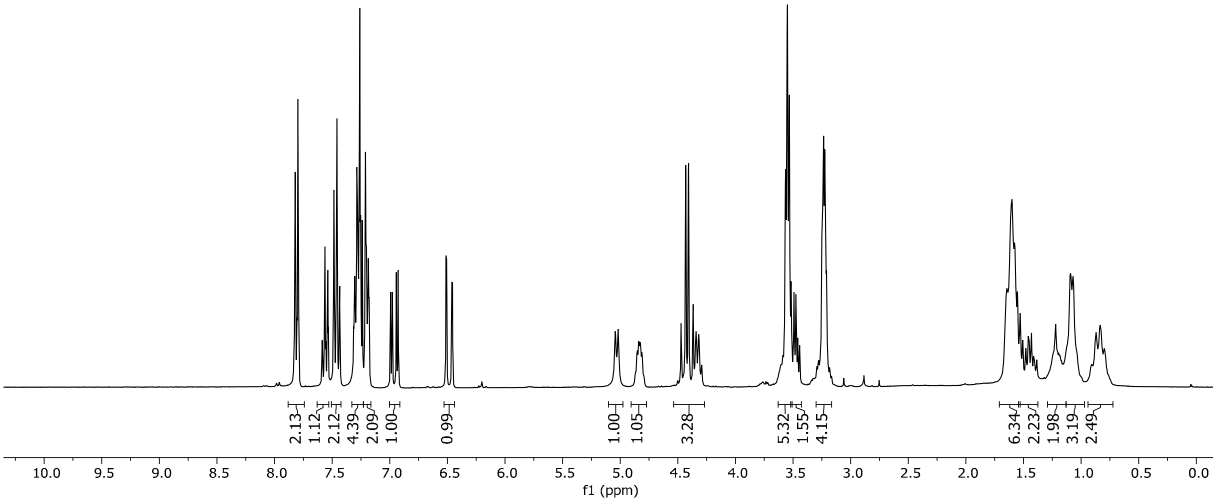

Figure S50. ^{1}H NMR of 35.

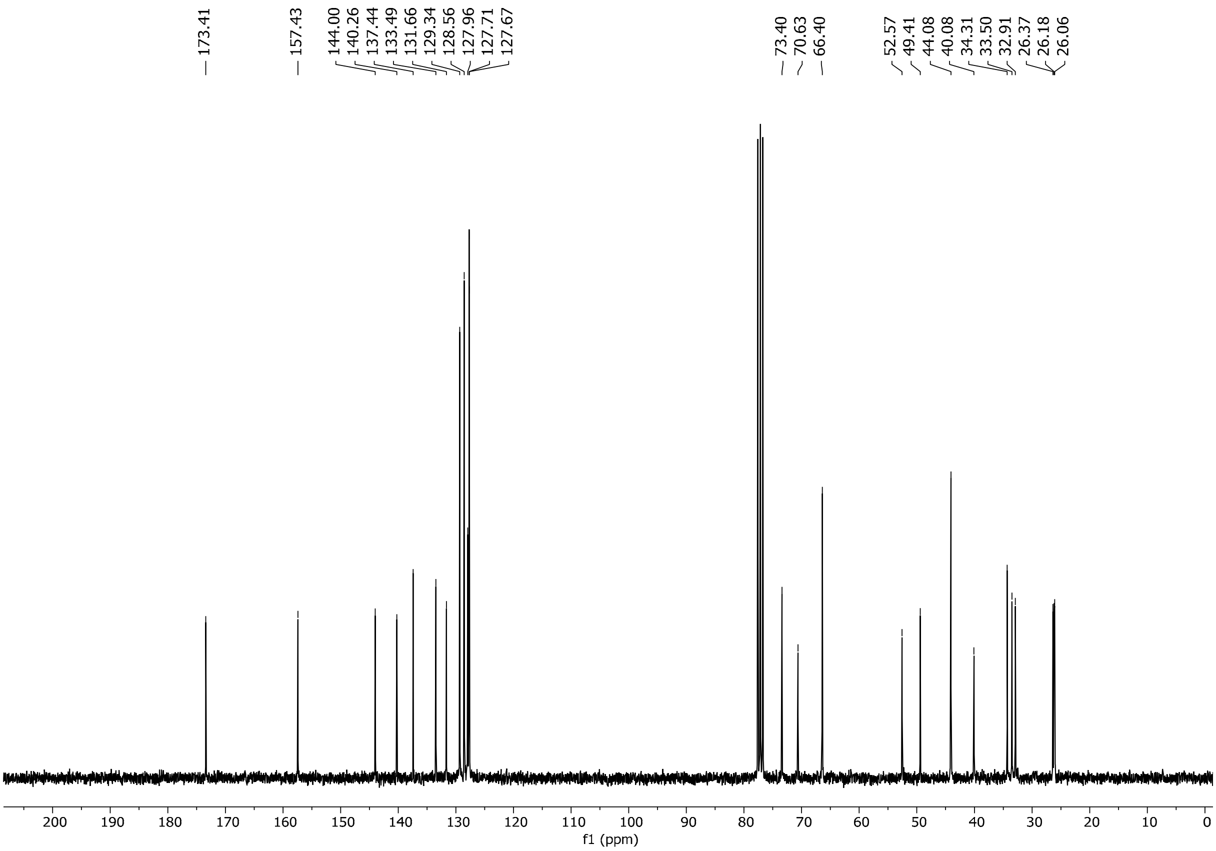

Figure S51. ^{13}C NMR of 35.

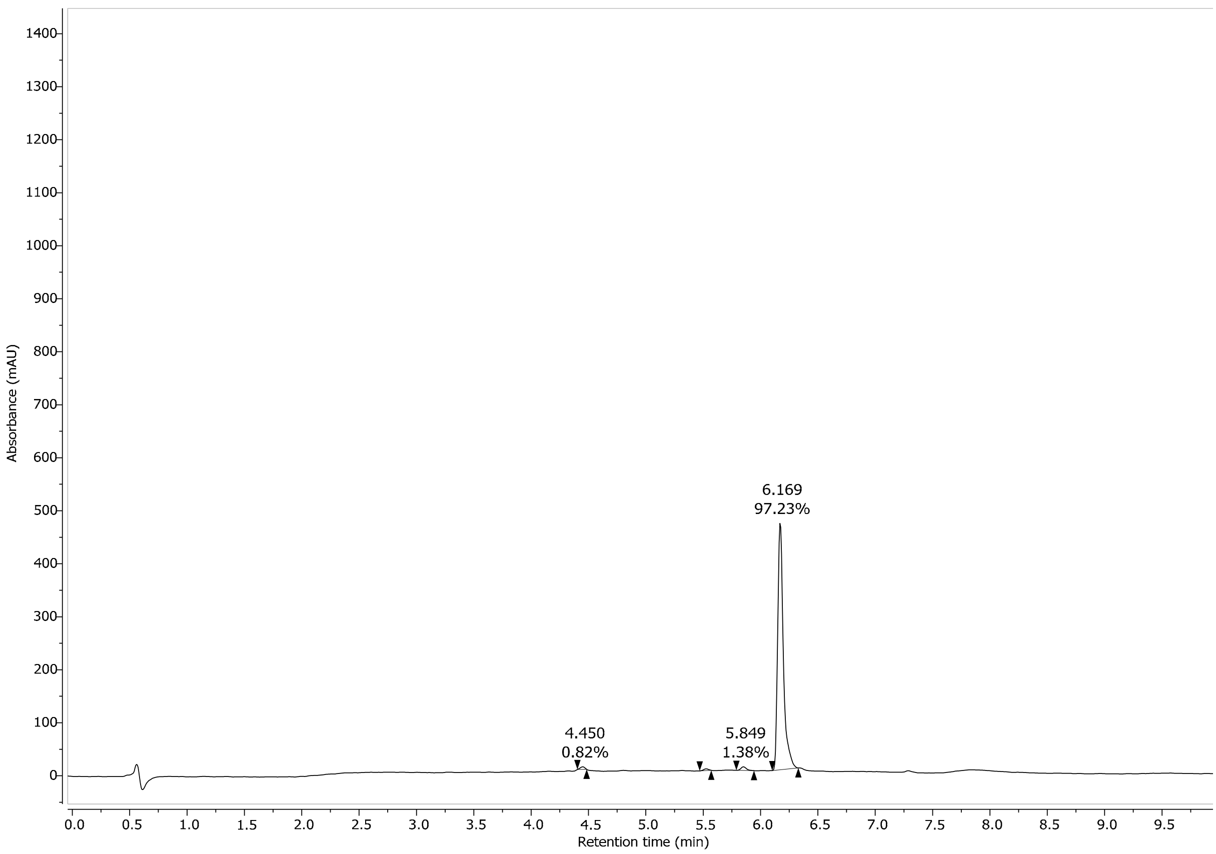

Figure S52. HPLC Chromatogram of 35 at 210 nm.

36, *N*-((*S*)-1-(((*S*,*E*)-1-(Benzyloxy)-4-fluoro-4-(phenylsulfonyl) but-3-en-2-yl) amino)-3-cyclohexyl-1-oxopropan-2-yl) morpholine-4-carboxamide

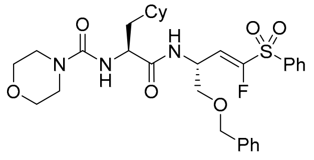

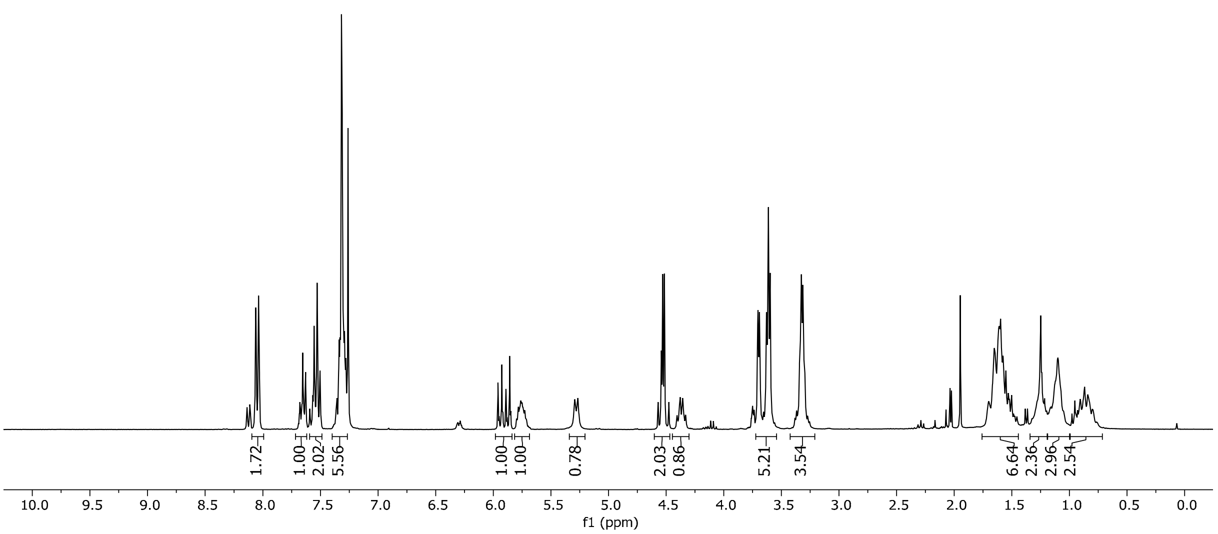

Figure S53. ^{1}H NMR of 36.

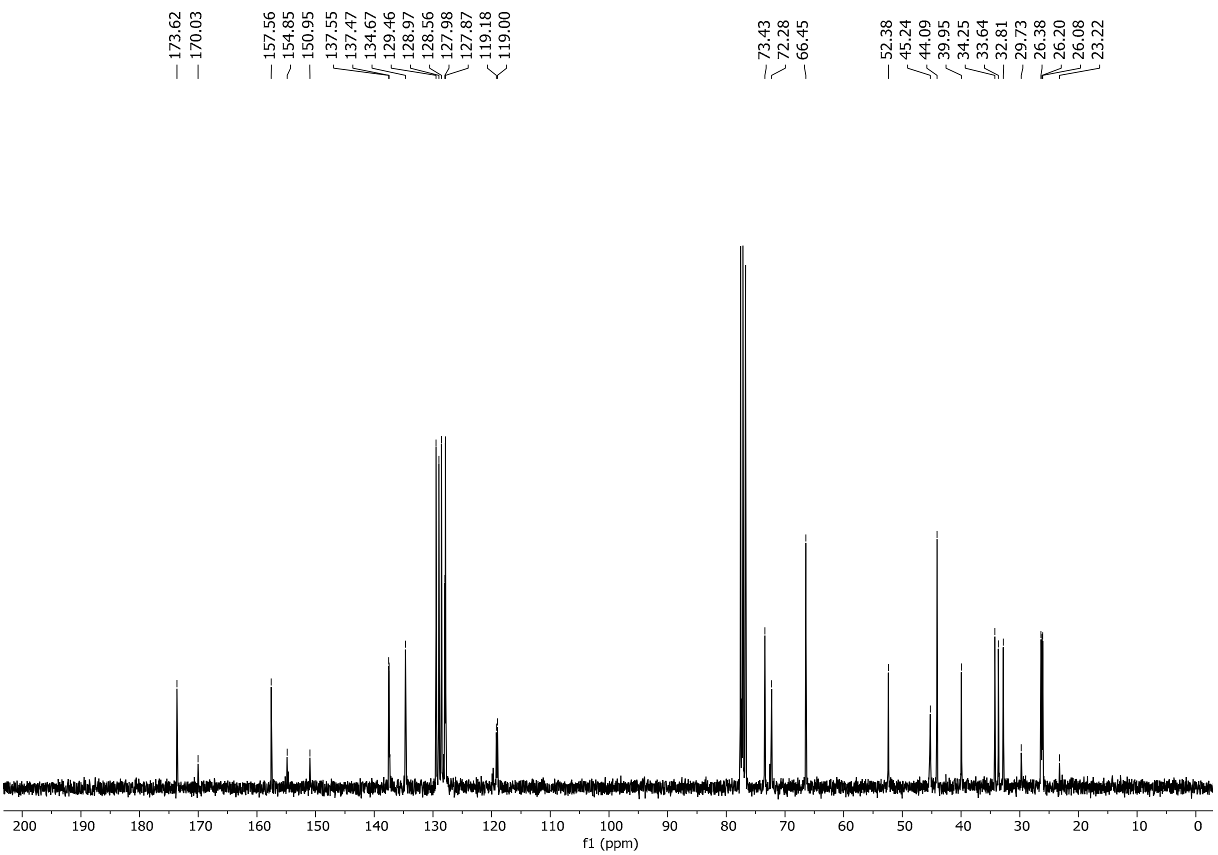

Figure S54. ^{13}C NMR of 36.

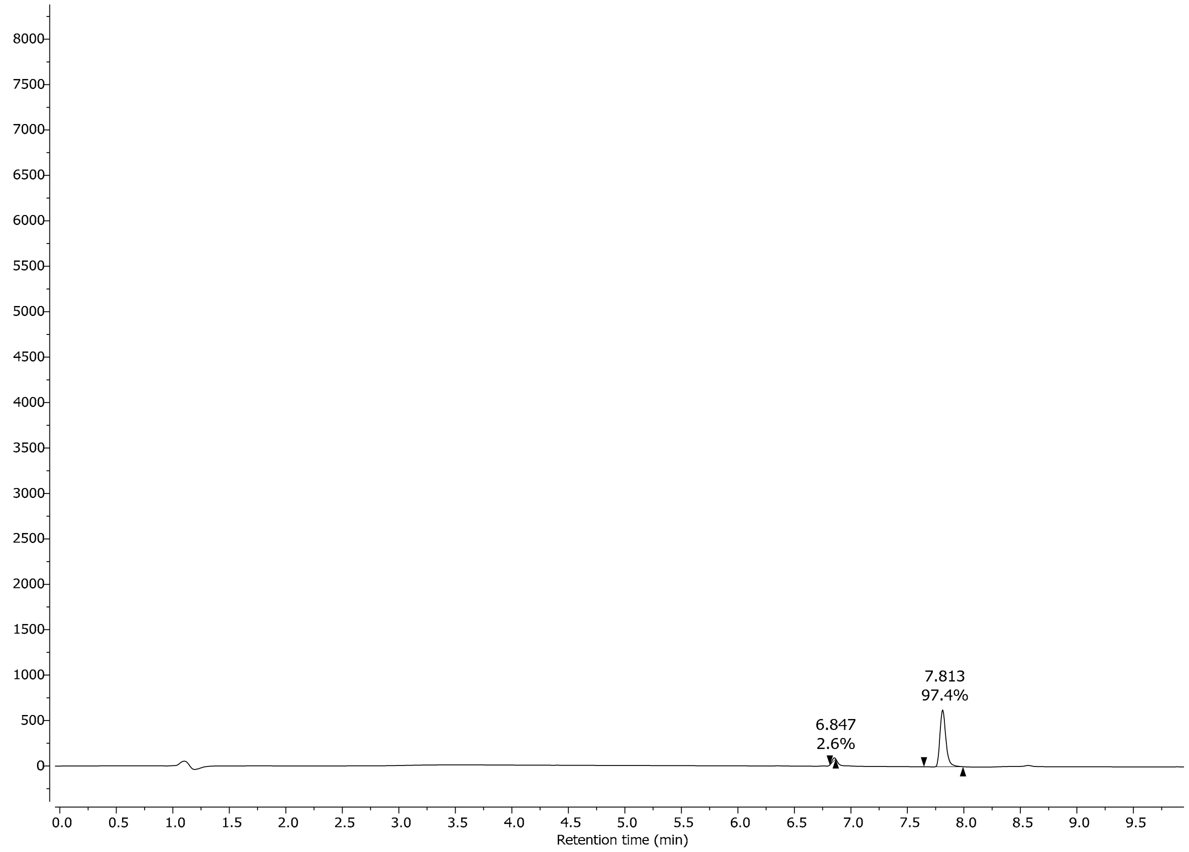

Figure S55. HPLC Chromatogram of 36 at 210 nm.

40, Ethyl (*S*,*E*)-6-(benzyloxy)-5-((*S*)-3-cyclohexyl-2-(morpholine-4-carboxamido) propanamido)-4-oxohex-2-enoate

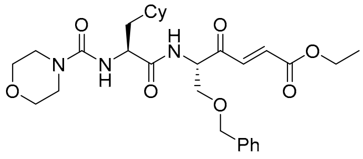

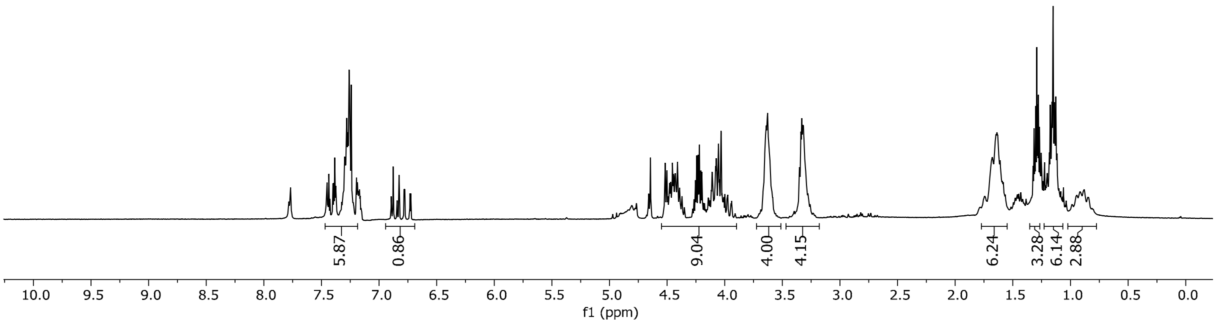

Figure S56. ^{1}H NMR of 40.

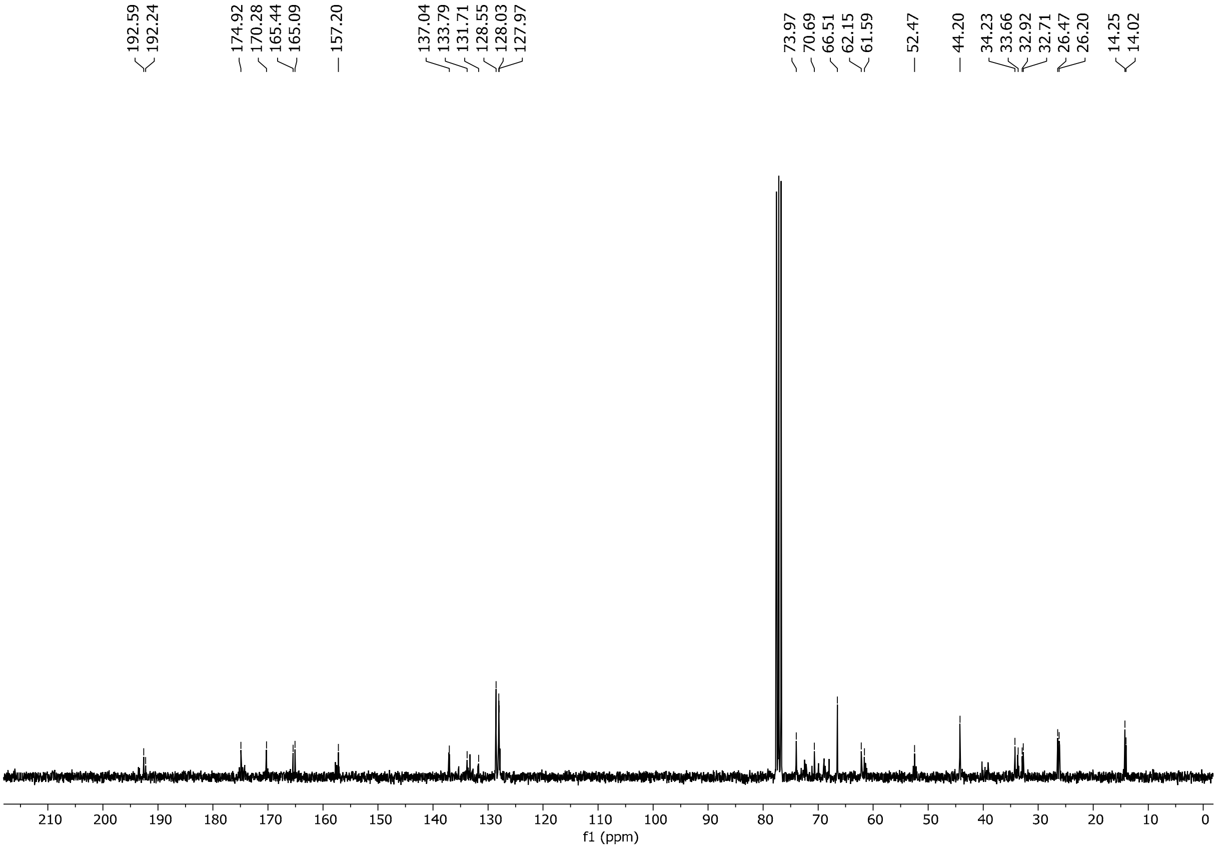

Figure S57. ^{13}C NMR of 40.

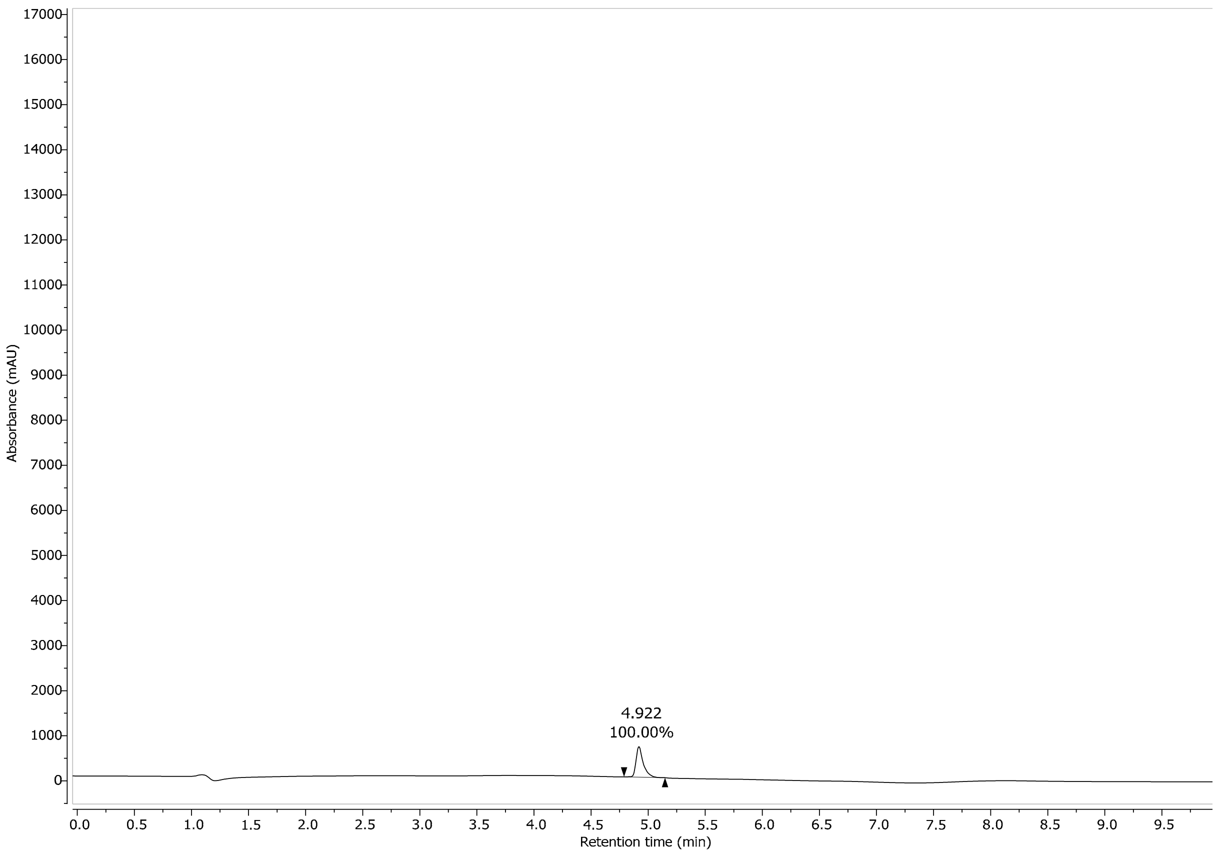

Figure S58. HPLC Chromatogram of 40 at 210 nm.

42, *N*-((*S*)-1-(((*S*)-1-(benzo[*d*]thiazol-2-yl)-3-(benzyloxy)-1-oxopropan-2-yl) amino)-3-cyclohexyl-1-oxopropan-2-yl) morpholine-4-carboxamide

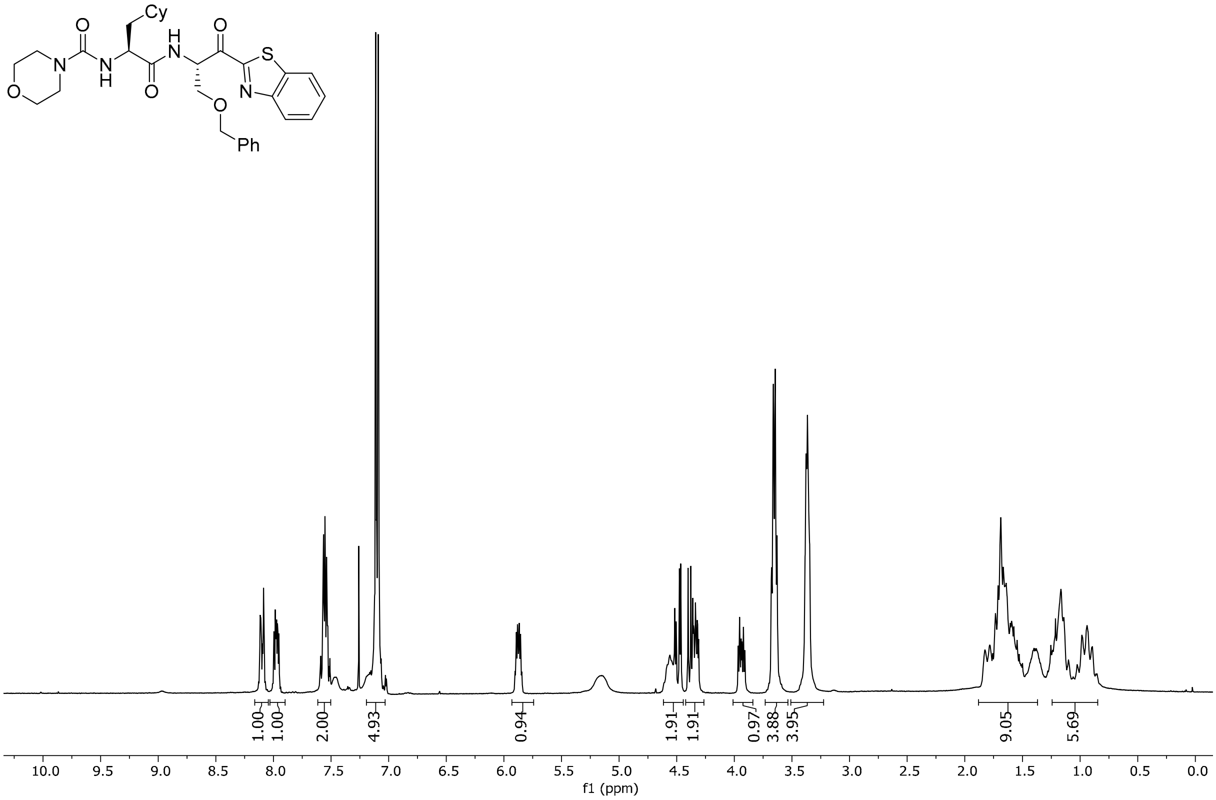

Figure S59. ^{1}H NMR of 42.

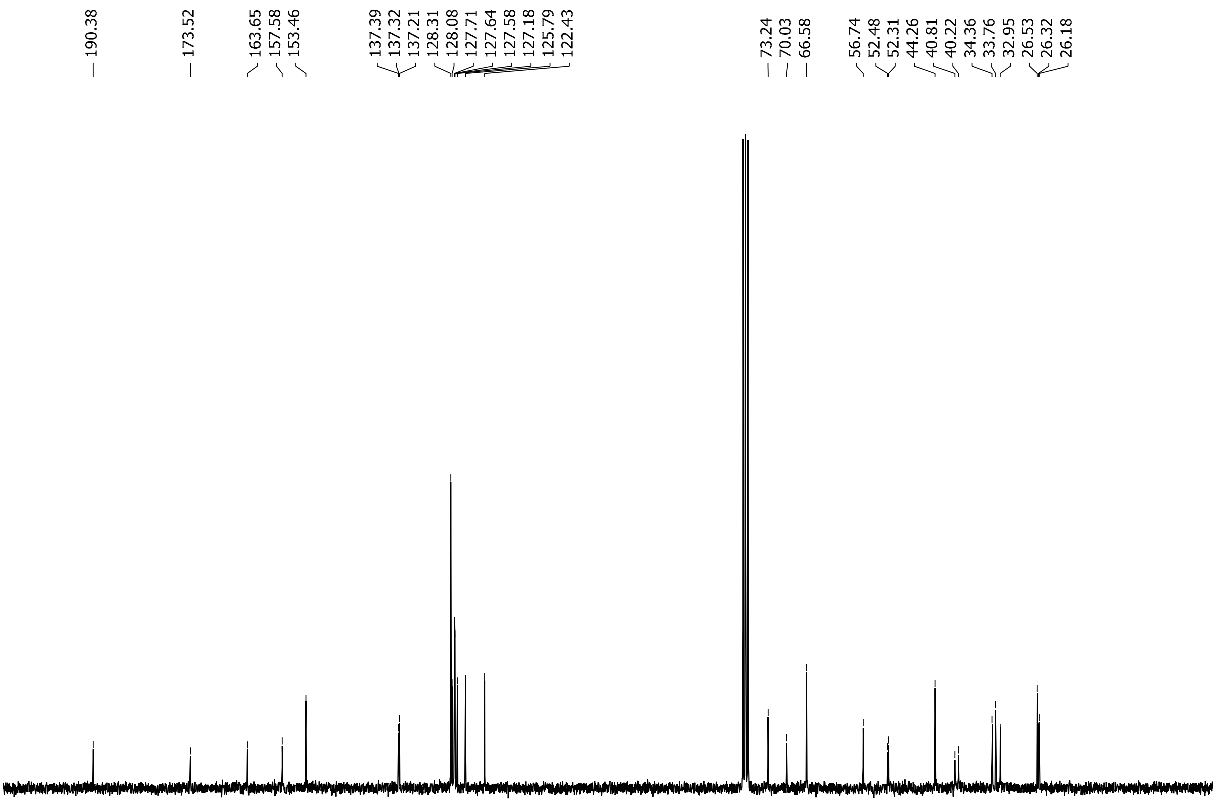

Figure S60. ^{13}C NMR of 42.

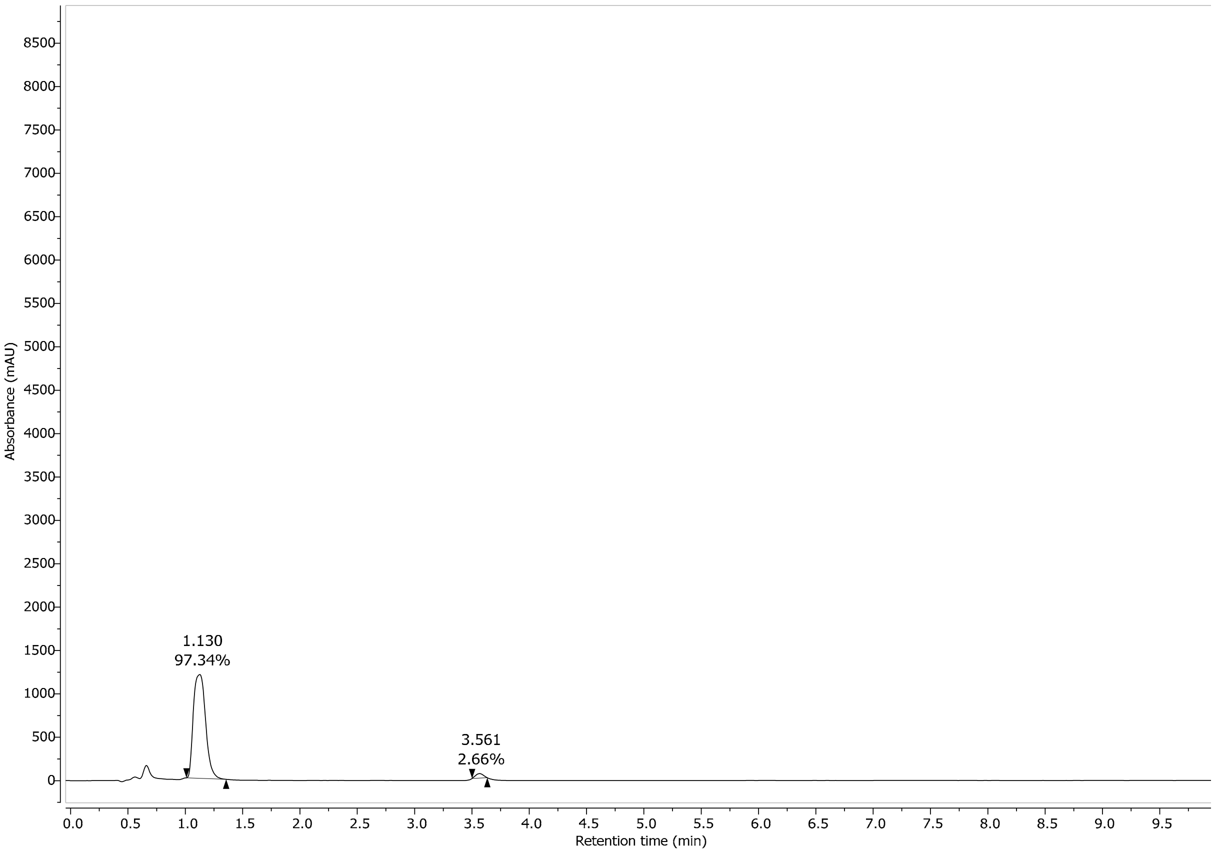

Figure S61. HPLC Chromatogram of 42 at 254 nm.

45, *N*-((2*S*)-1-(((2*S*)-3-(benzyloxy)-1-oxo-1-((2-oxo-1-phenylazetidin-3-yl) amino) propan-2-yl) amino)-3-cyclohexyl-1-oxopropan-2-yl) morpholine-4-carboxamide

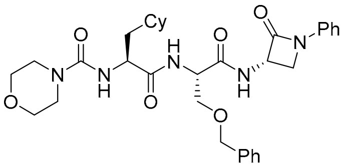

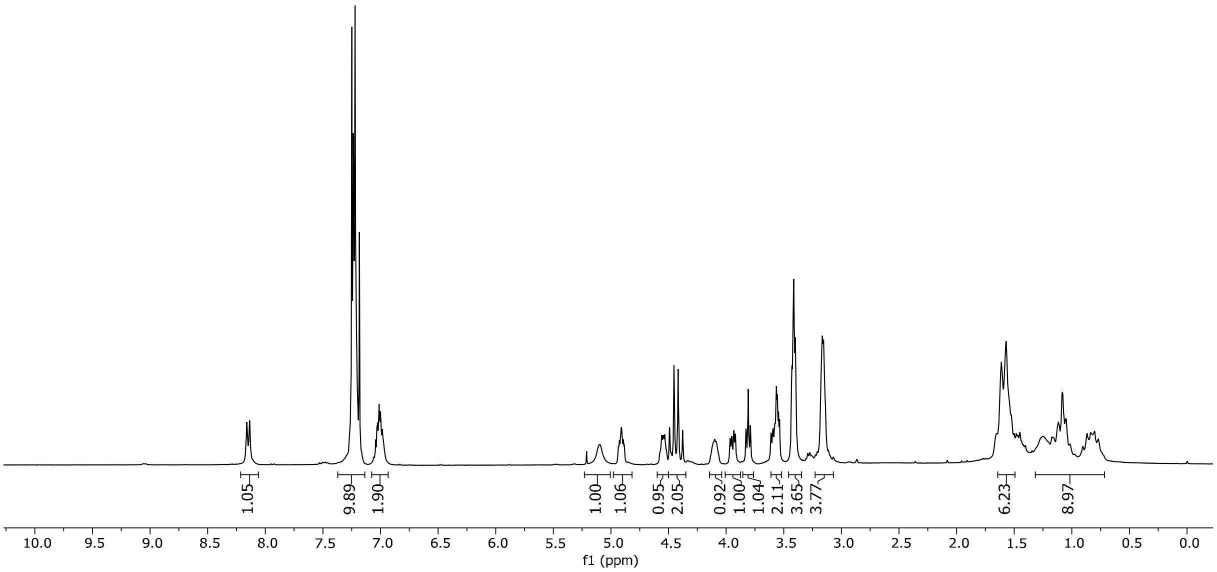

Figure S62. ^{1}H NMR of 45.

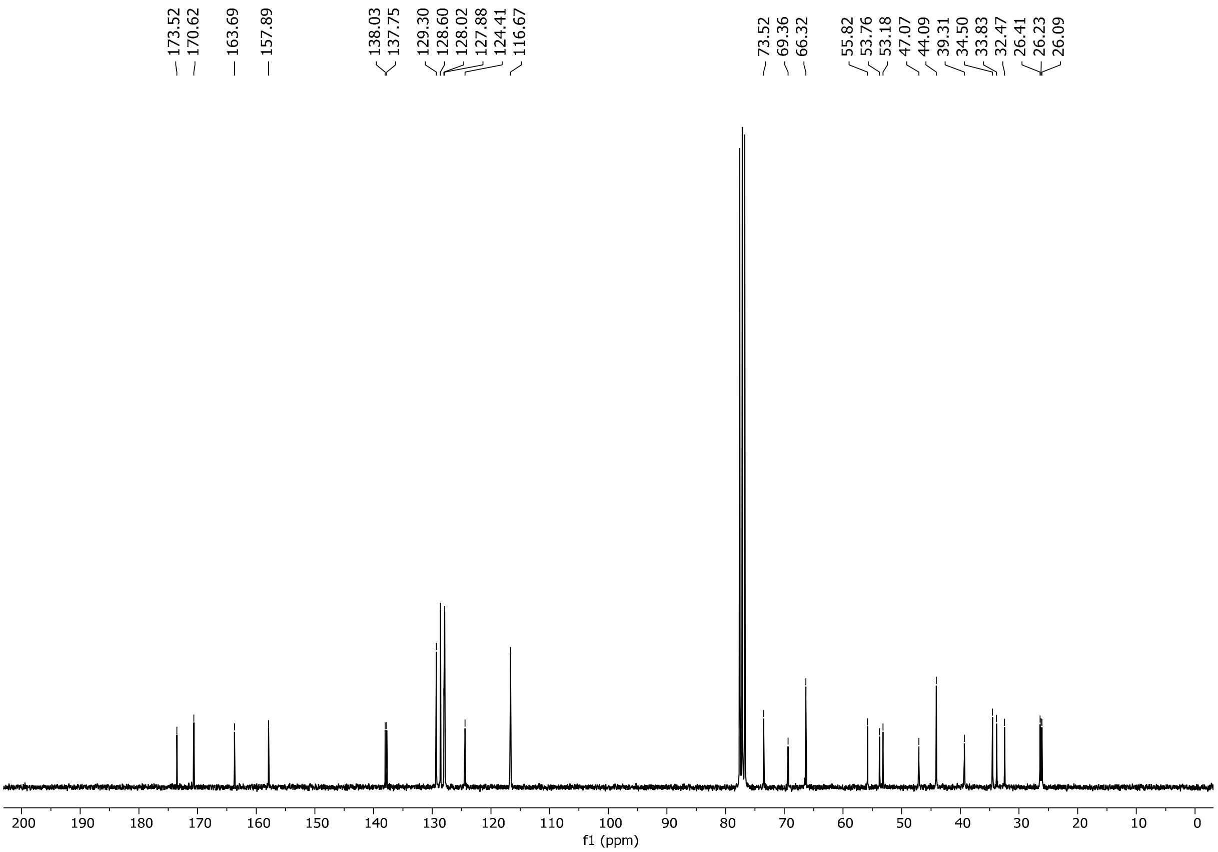

Figure S63. ^{13}C NMR of 45.

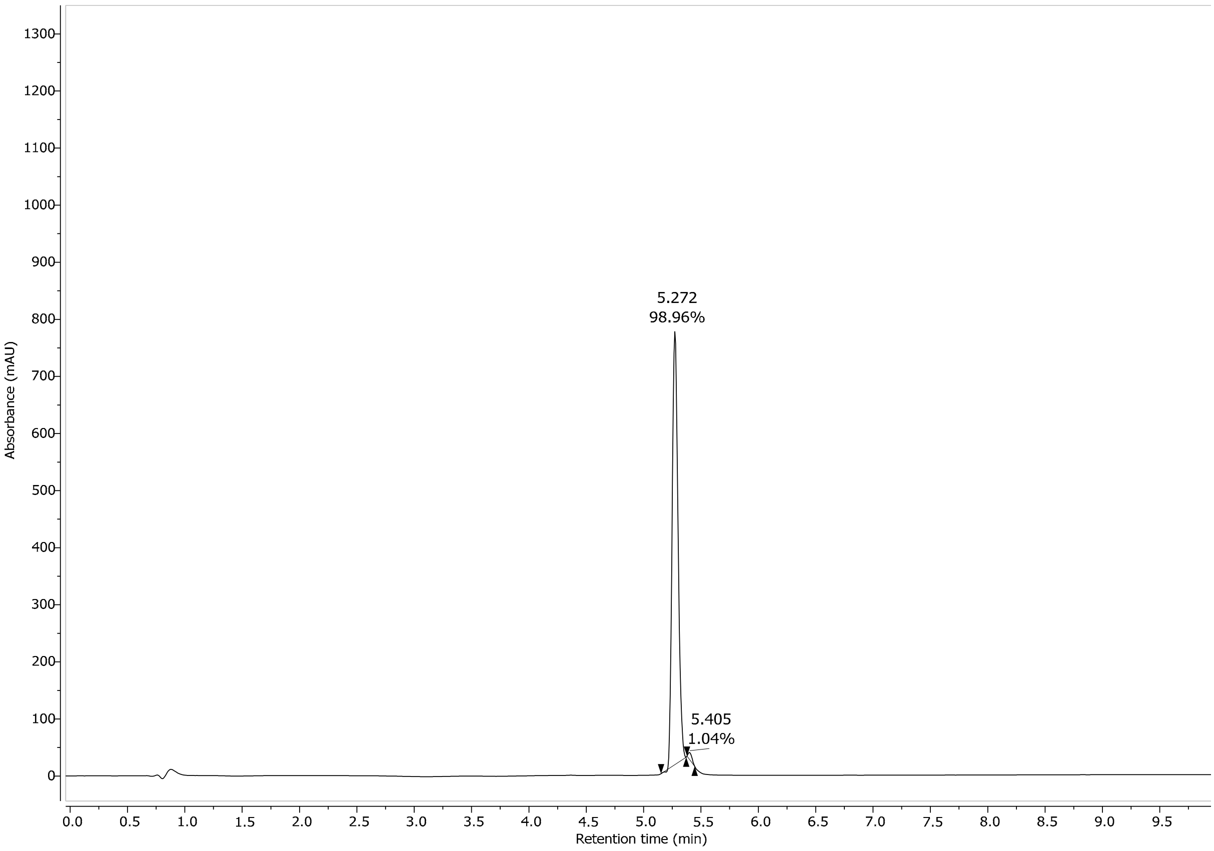

Figure S64. HPLC Chromatogram of 45 at 254 nm.

49, *N*-((*S*)-1-(((*R*,*E*)-1-(benzyloxy)-4-nitrobut-3-en-2-yl) amino)-3-cyclohexyl-1-oxopropan-2-yl) morpholine-4-carboxamide

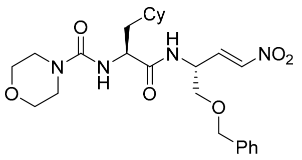

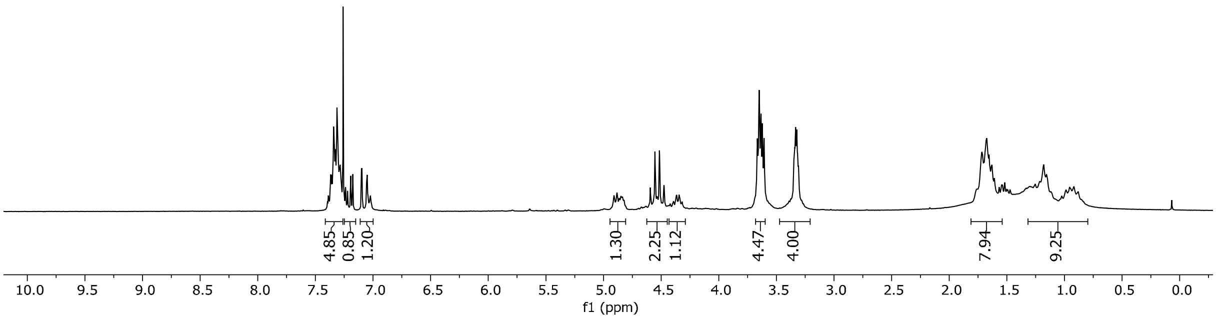

Figure S65. ^{1}H NMR of 49.

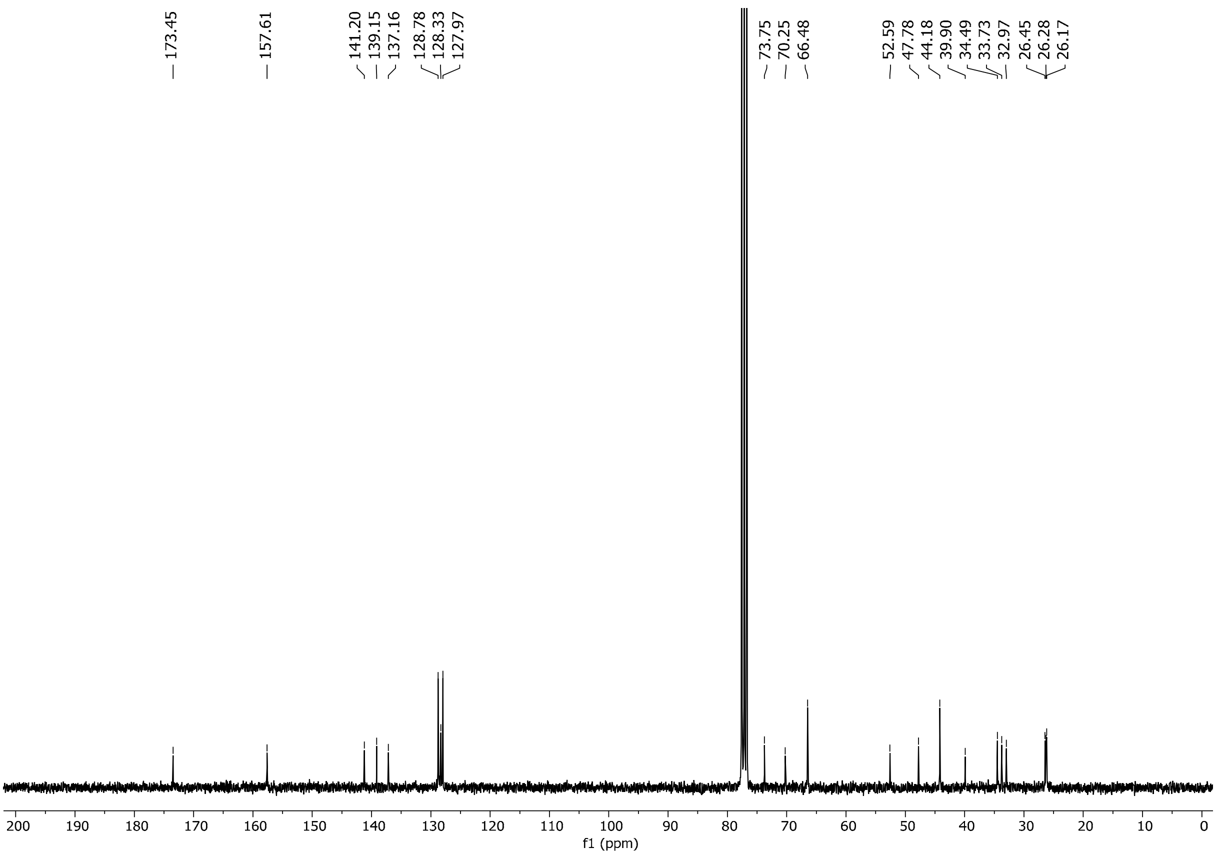

Figure S66. ^{13}C NMR of 49.

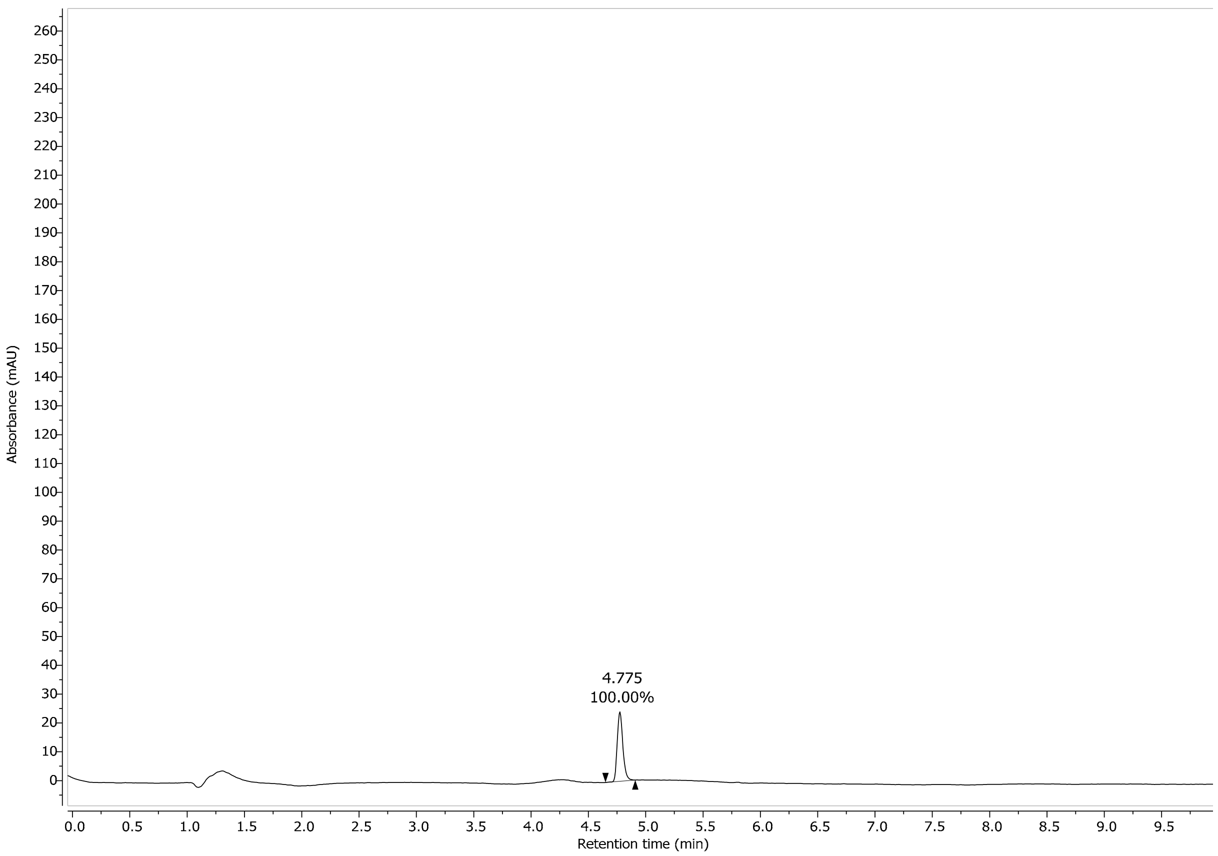

Figure S67. HPLC Chromatogram of 49 at 254 nm.

55, *N*-((*S*)-1-(((*S,E*)-1-fluoro-5-methyl-1-(phenylsulfonyl)hex-1-en-3-yl)amino)-1-oxo-3-phenylpropan-2-yl)pyrazine-2-carboxamide

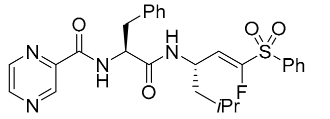

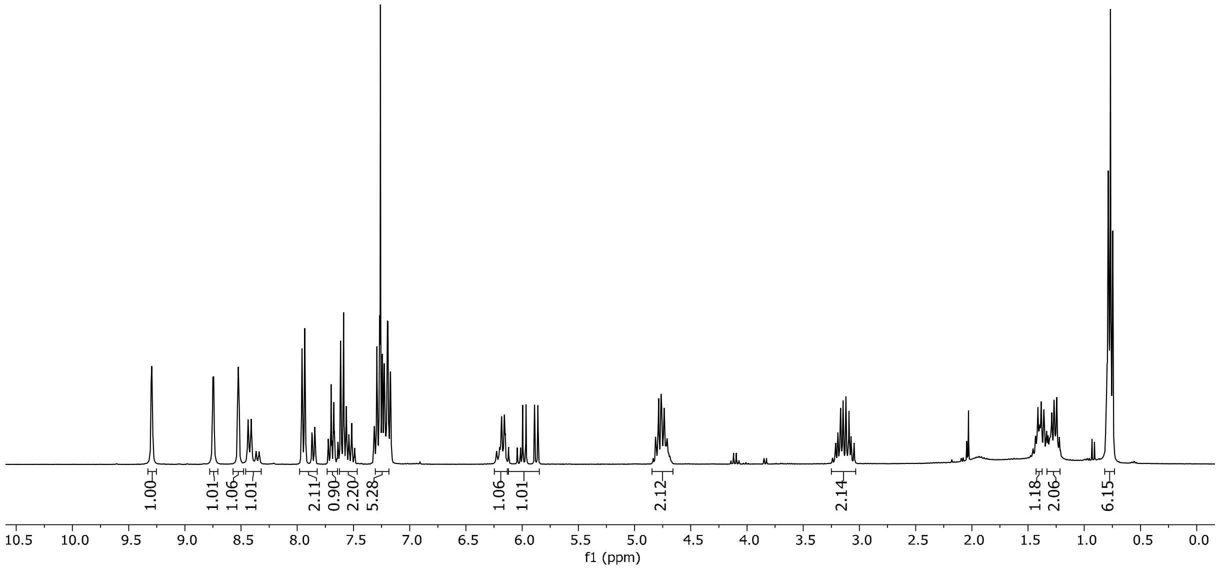

Figure S68. ^{1}H NMR of 55.

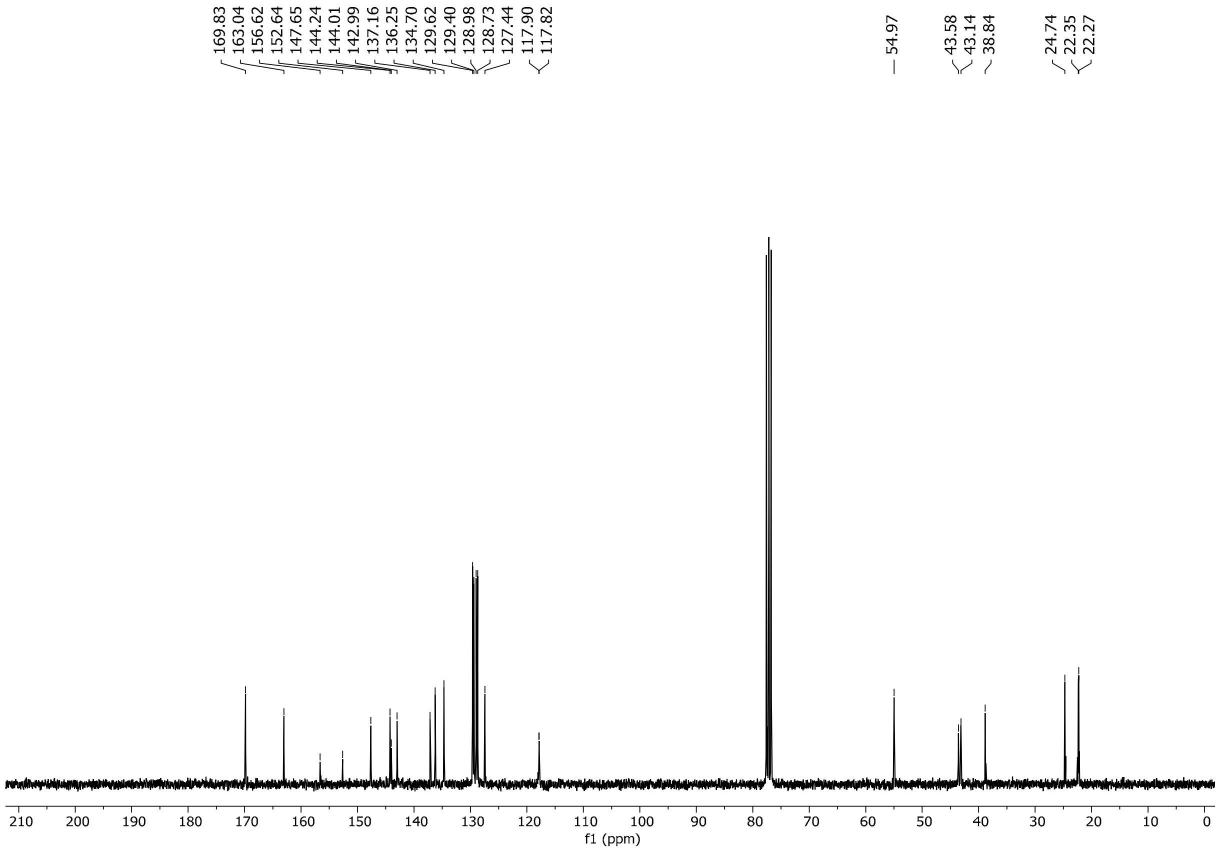

Figure S69. ^{13}C NMR of 55.

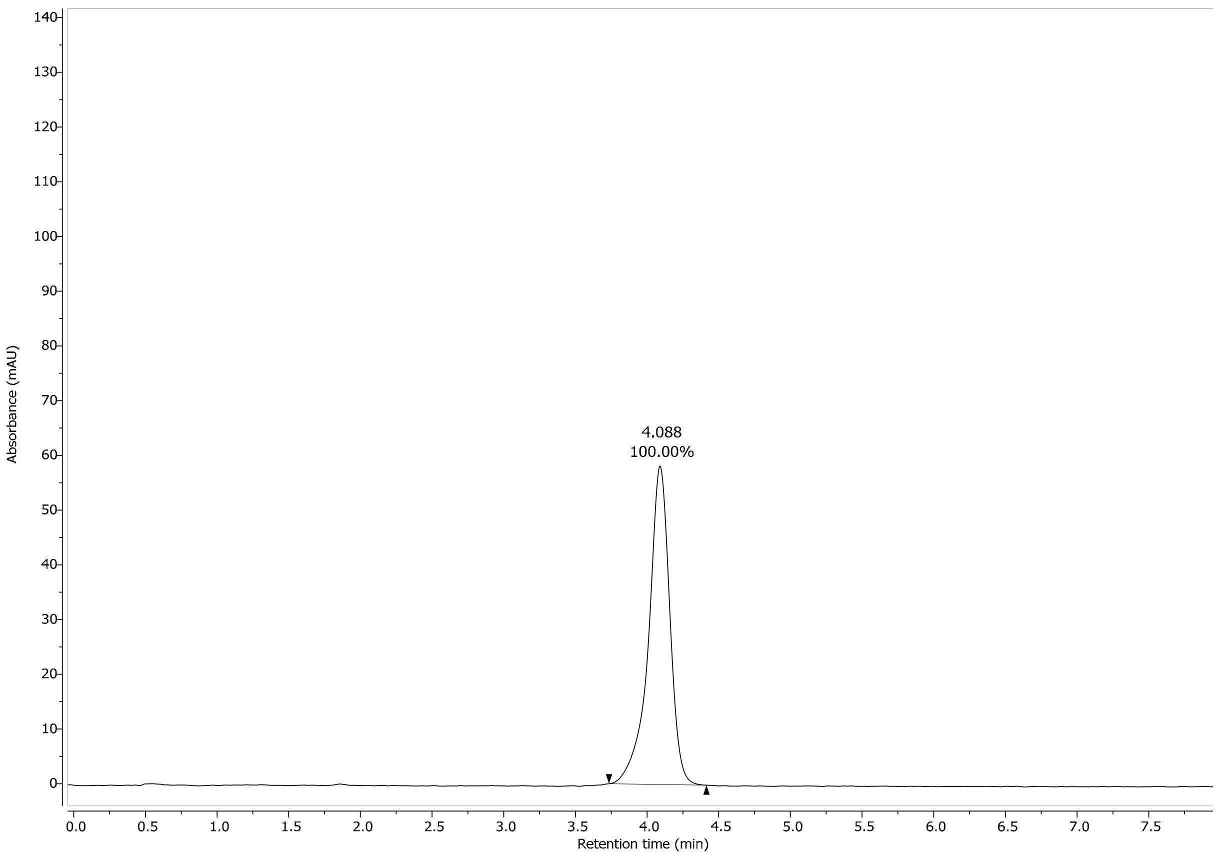

Figure S70. HPLC Chromatogram of 55 at 254 nm.

58, *N*-((*S*)-1-(((*S*,*E*)-5-Methyl-1-(phenylsulfonyl)hex-1-en-3-yl)amino)-1-oxo-3-phenylpropan-2-yl)pyrazine-2-carboxamide

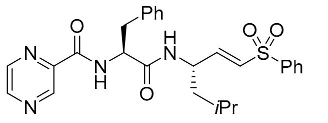

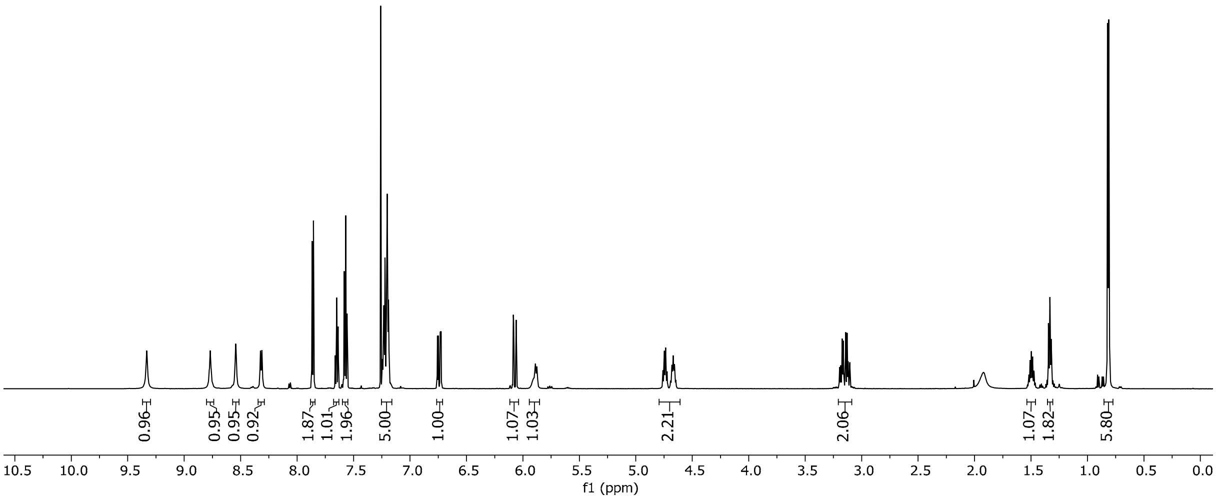

Figure S71. ^{1}H NMR of 58.

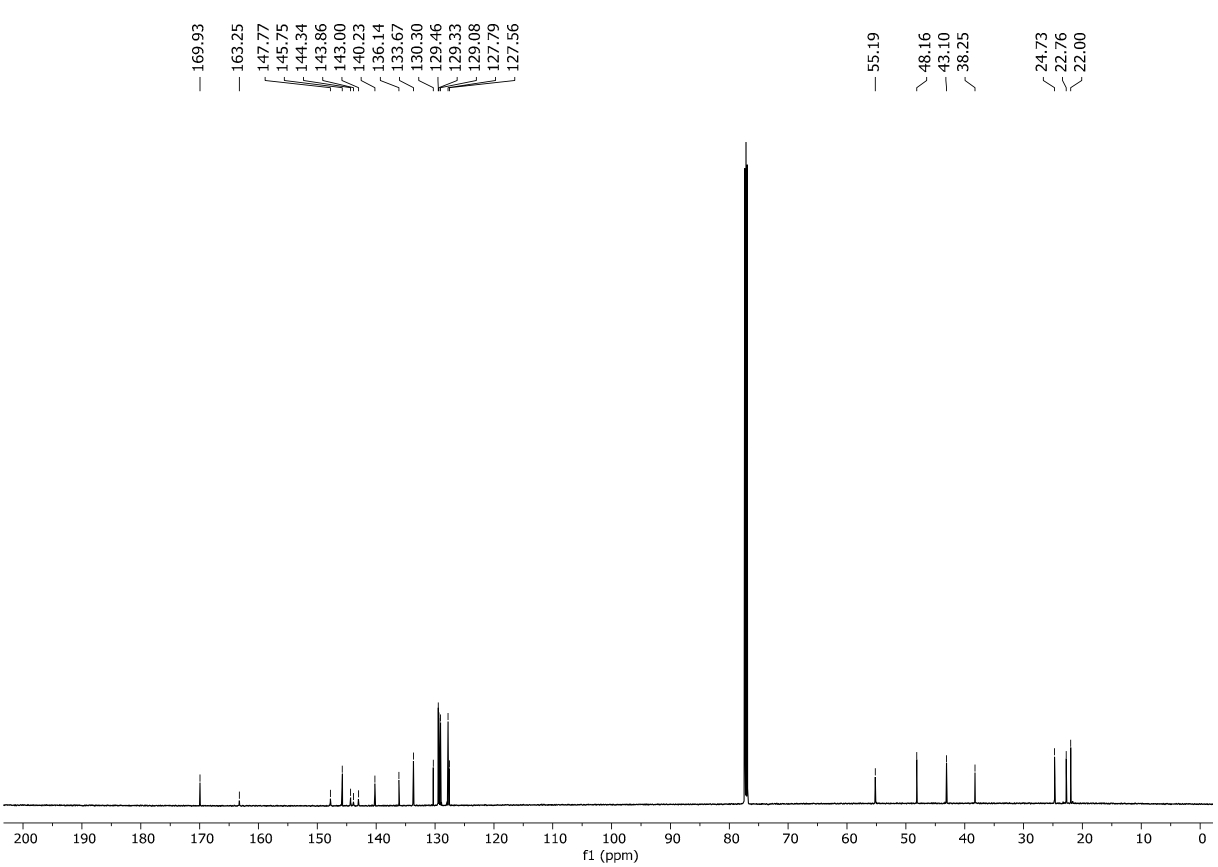

Figure S72. ^{13}C NMR of 58.

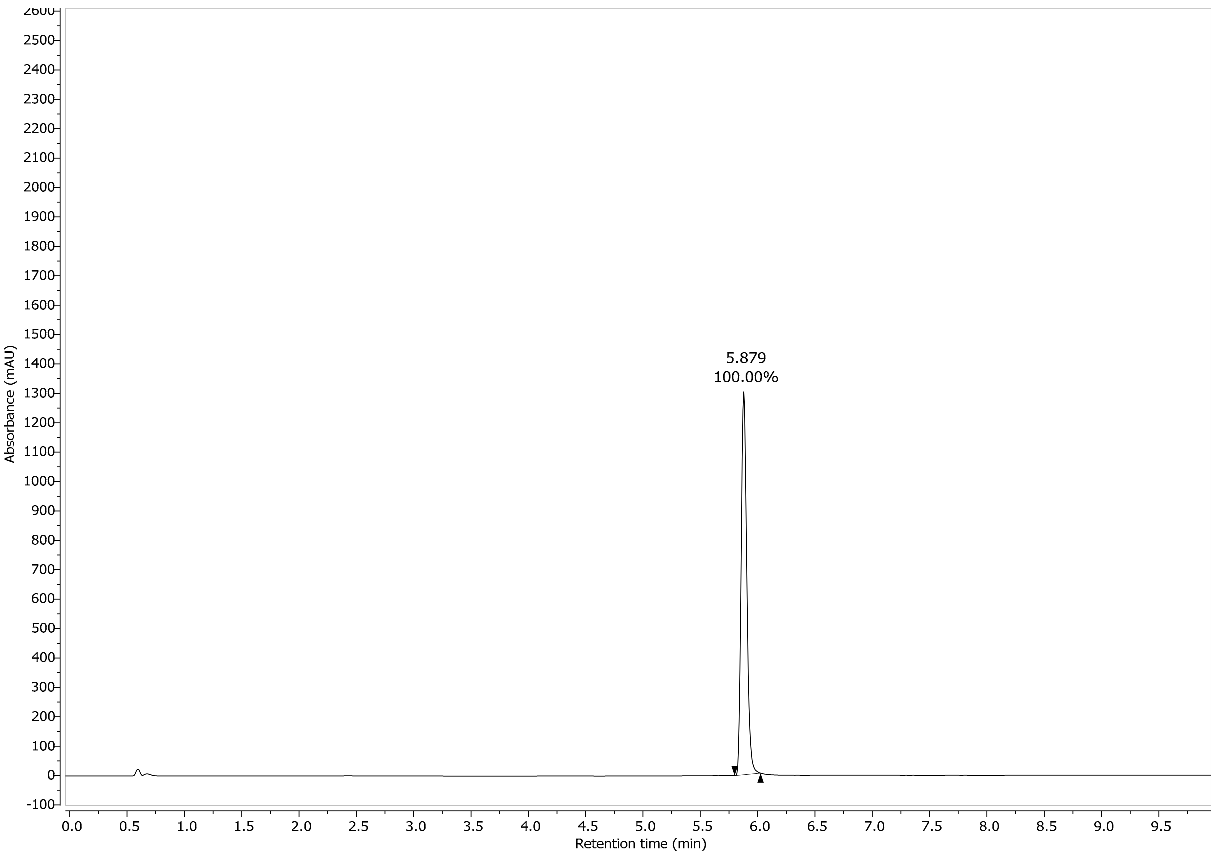

Figure S73. HPLC Chromatogram of 58 at 254 nm.

59, *N*-((*S*)-1-(((*S*)-5-Methyl-1-nitrohex-1-en-3-yl)amino)-1-oxo-3-phenylpropan-2-yl)pyrazine-2-carboxamide

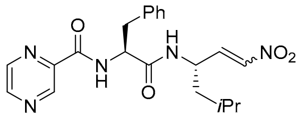

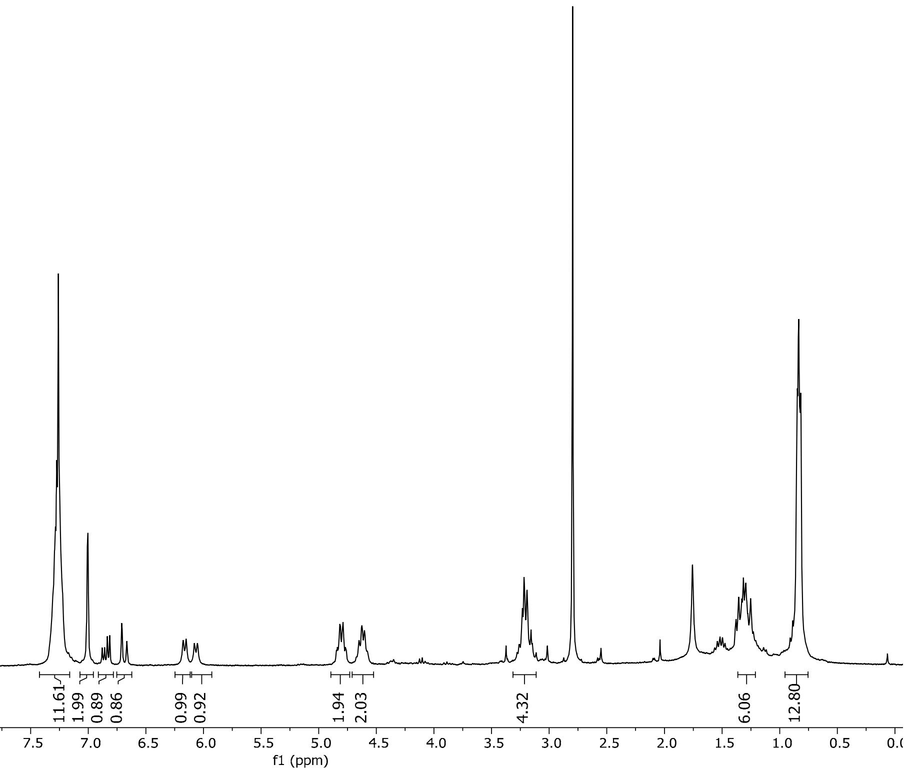

Figure S74. ^{1}H NMR of 59.

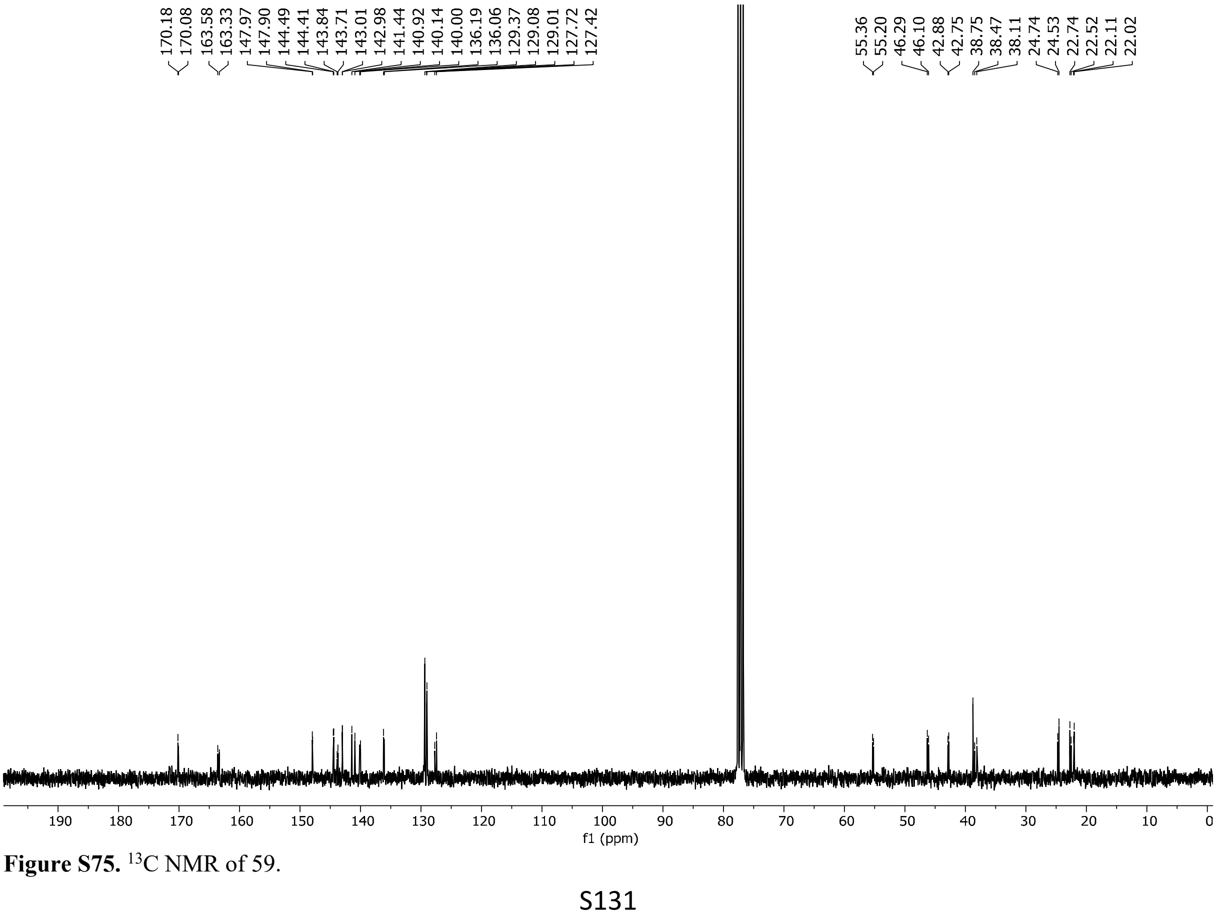

Figure S75. ^{13}C NMR of 59.

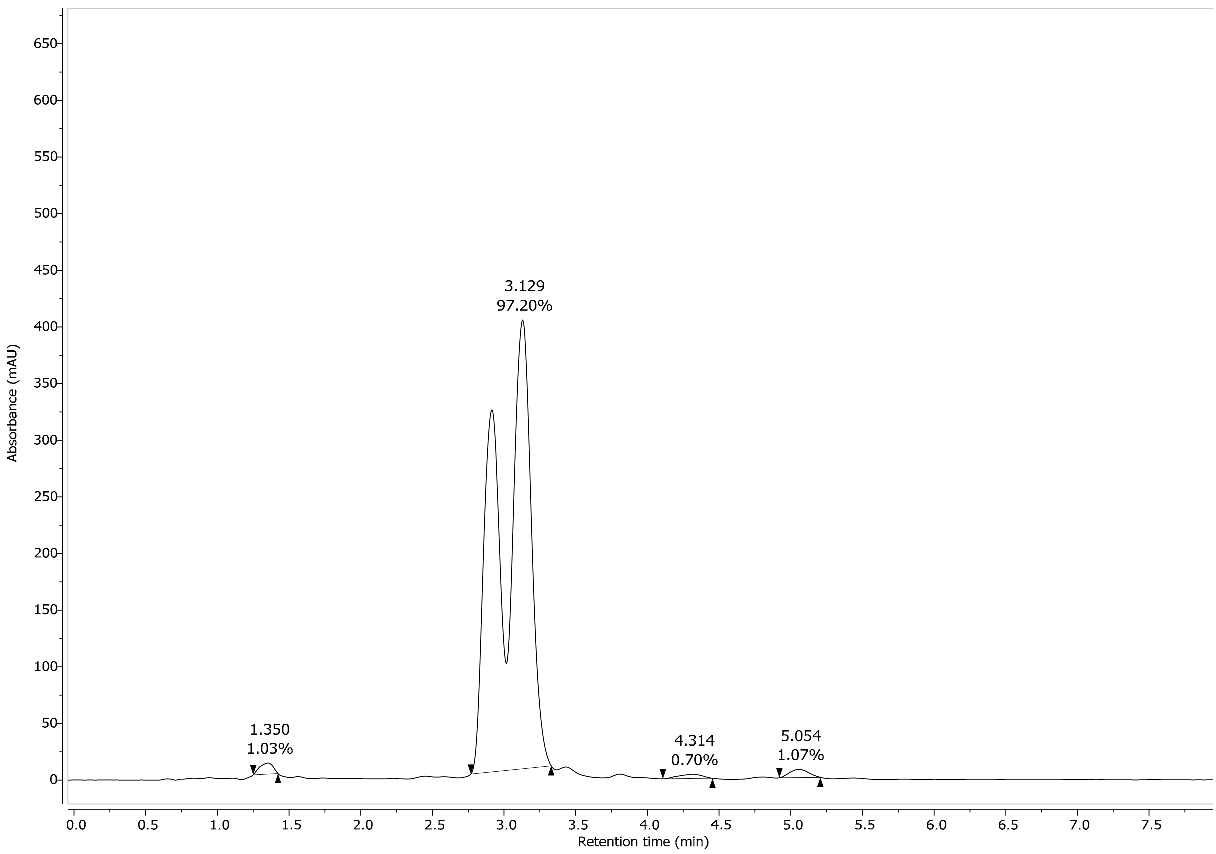

Figure S76. HPLC Chromatogram of 59 at 254 nm.

62, *N*-((*S*)-1-(((*S*)-1-(Benzo[*d*]thiazol-2-yl)-4-methyl-1-oxopentan-2-yl)amino)-1-oxo-3-phenylpropan-2-yl)pyrazine-2-carboxamide

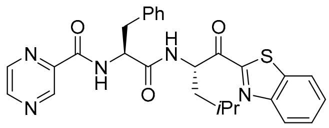

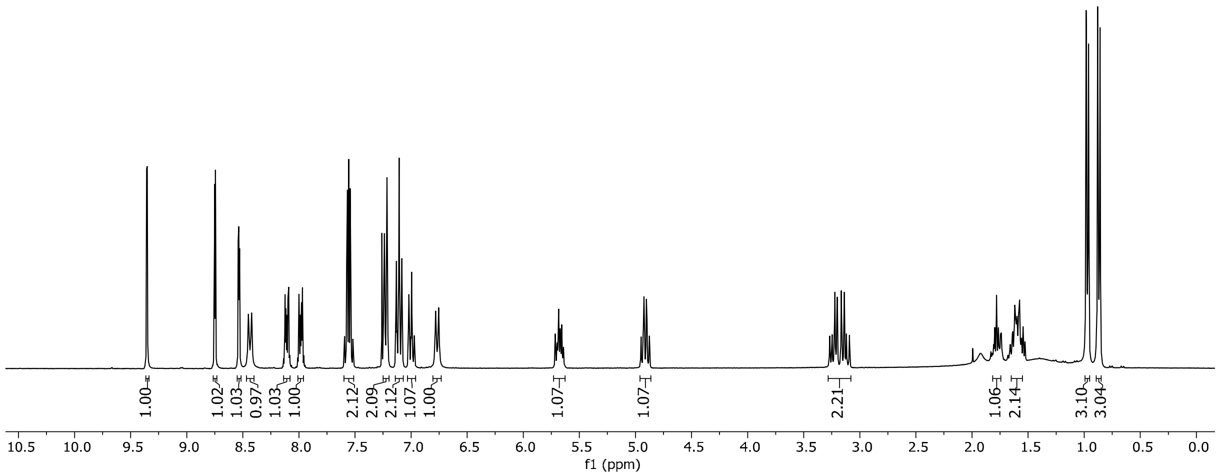

Figure S77. ^{1}H NMR of 62.

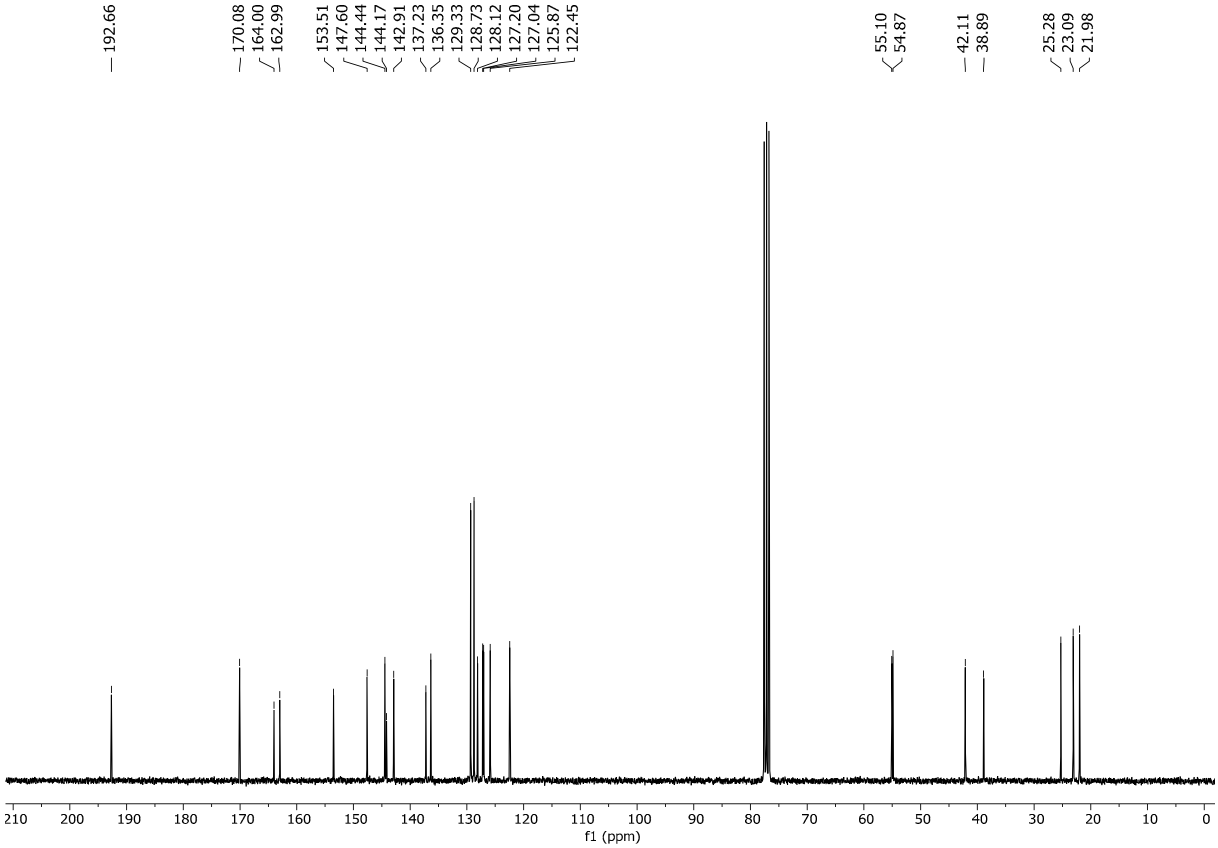

Figure S78. ^{13}C NMR of 62.

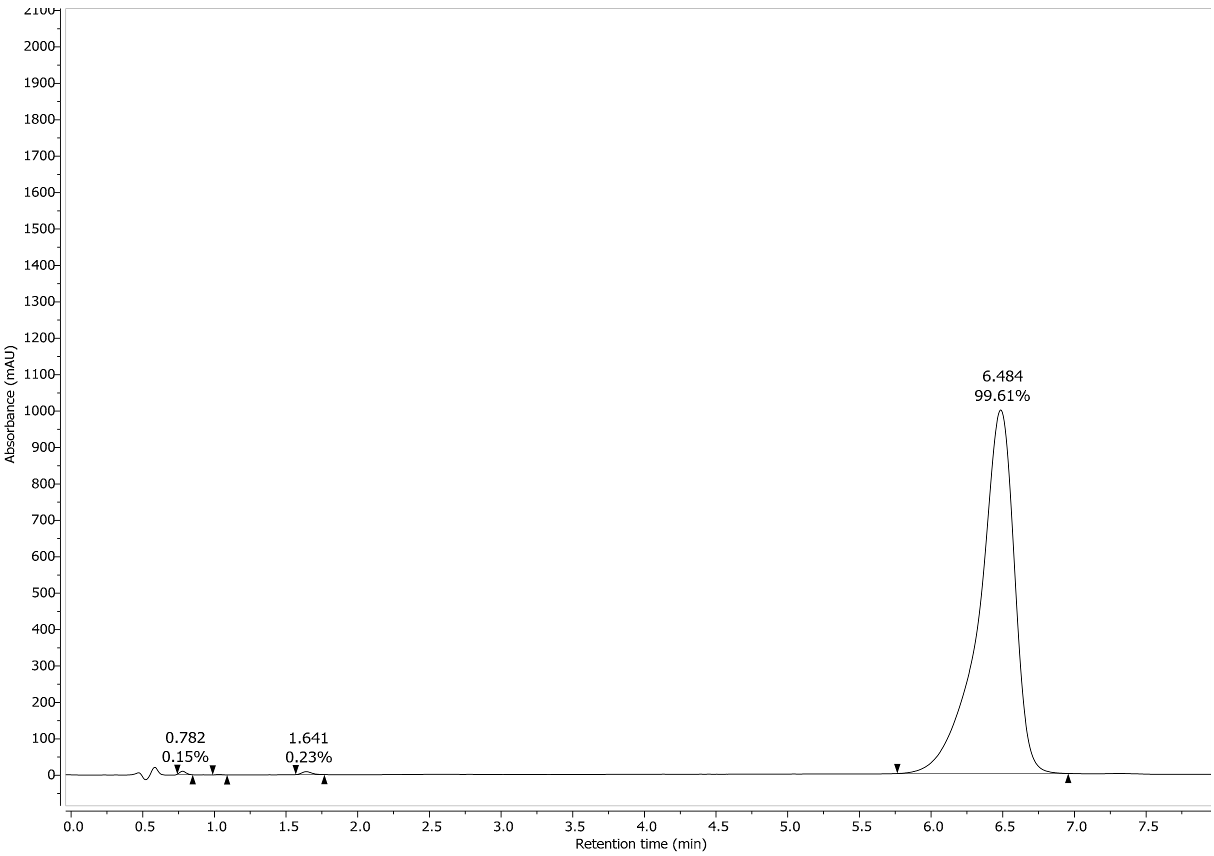

Figure S79. HPLC Chromatogram of 62 at 254 nm.

68, Ethyl (*S,E*)-7-methyl-4-oxo-5-((*S*)-3-phenyl-2-(pyrazine-2-carboxamido)propanamido)oct-2-enoate

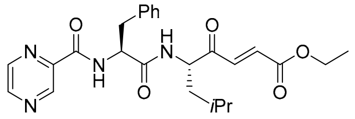

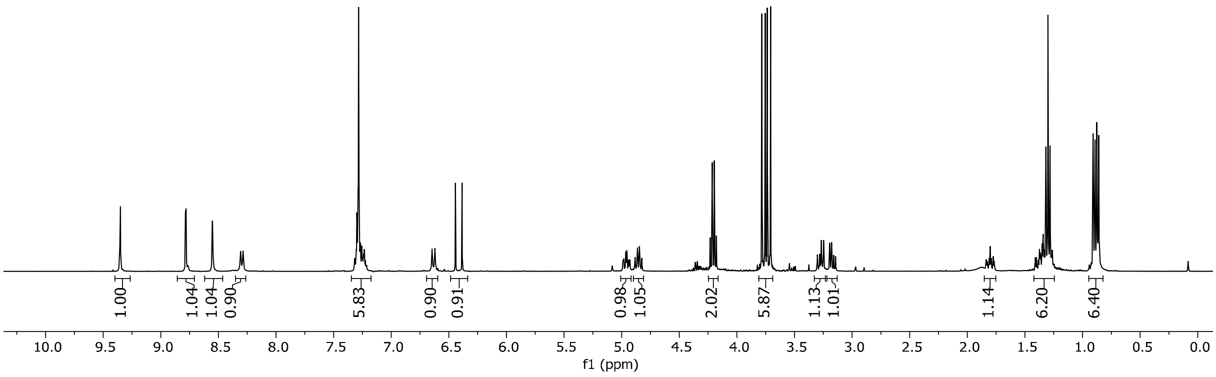

Figure S80. ^{1}H NMR of 68.

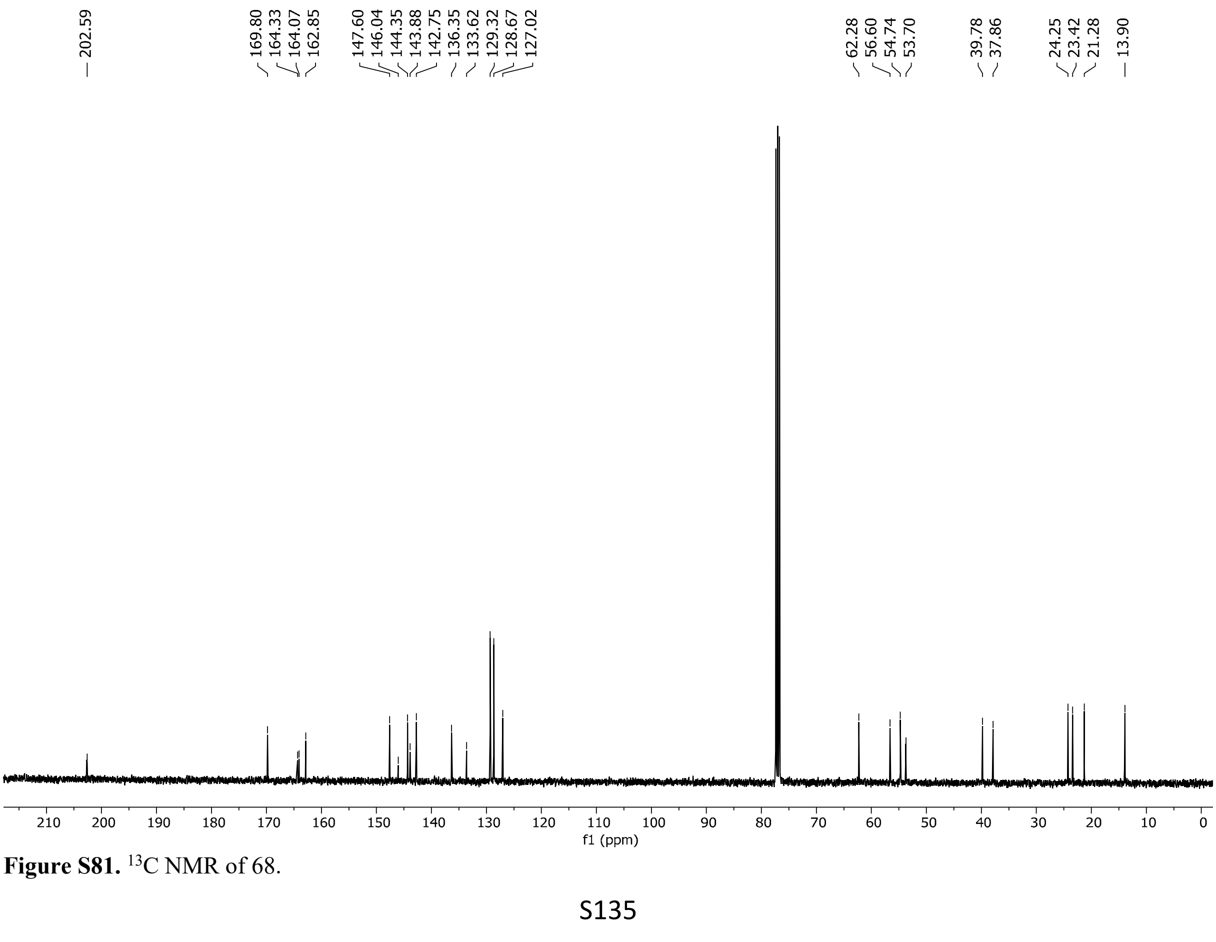

Figure S81. ^{13}C NMR of 68.

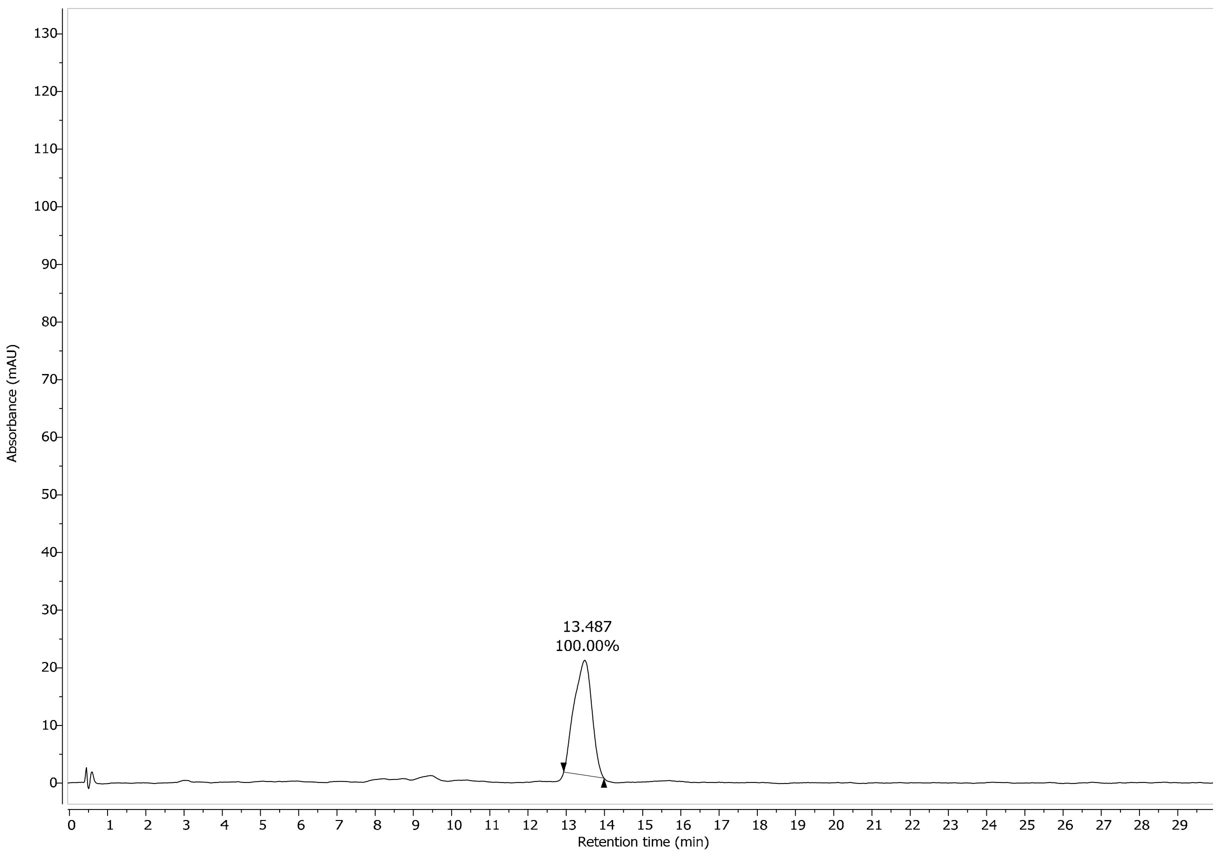

Figure S82. HPLC Chromatogram of 68 at 254 nm.

72, *N*-((*S*)-1-(((*S*)-4-Methyl-1-oxo-1-(((*S*)-2-oxo-1-phenylazetidin-3-yl)amino)pentan-2-yl)amino)-1-oxo-3-phenylpropan-2-yl)pyrazine-2-carboxamide

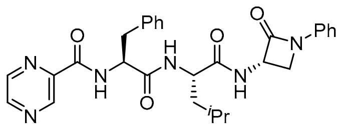

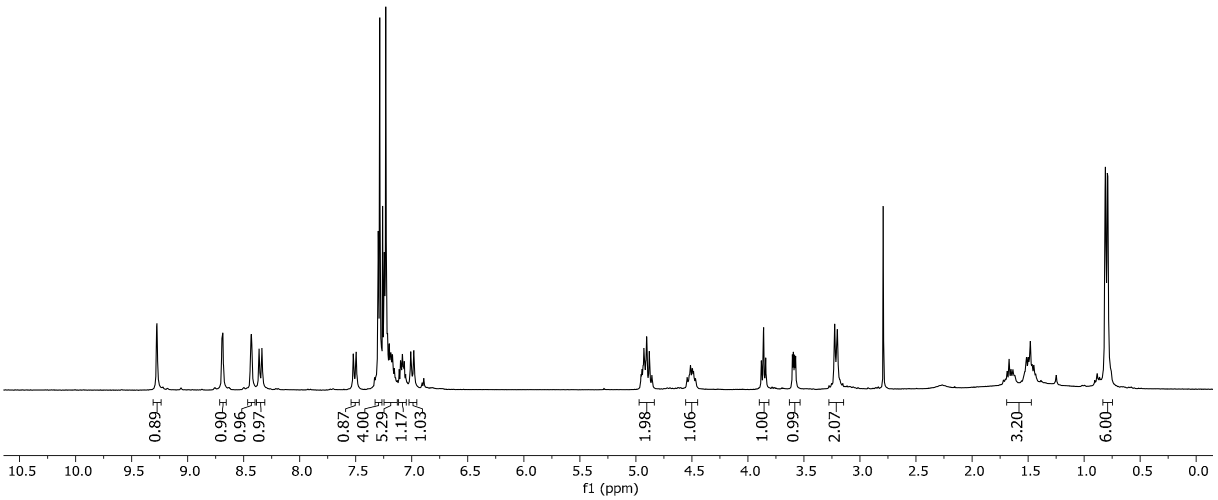

Figure S83. ^{1}H NMR of 72.

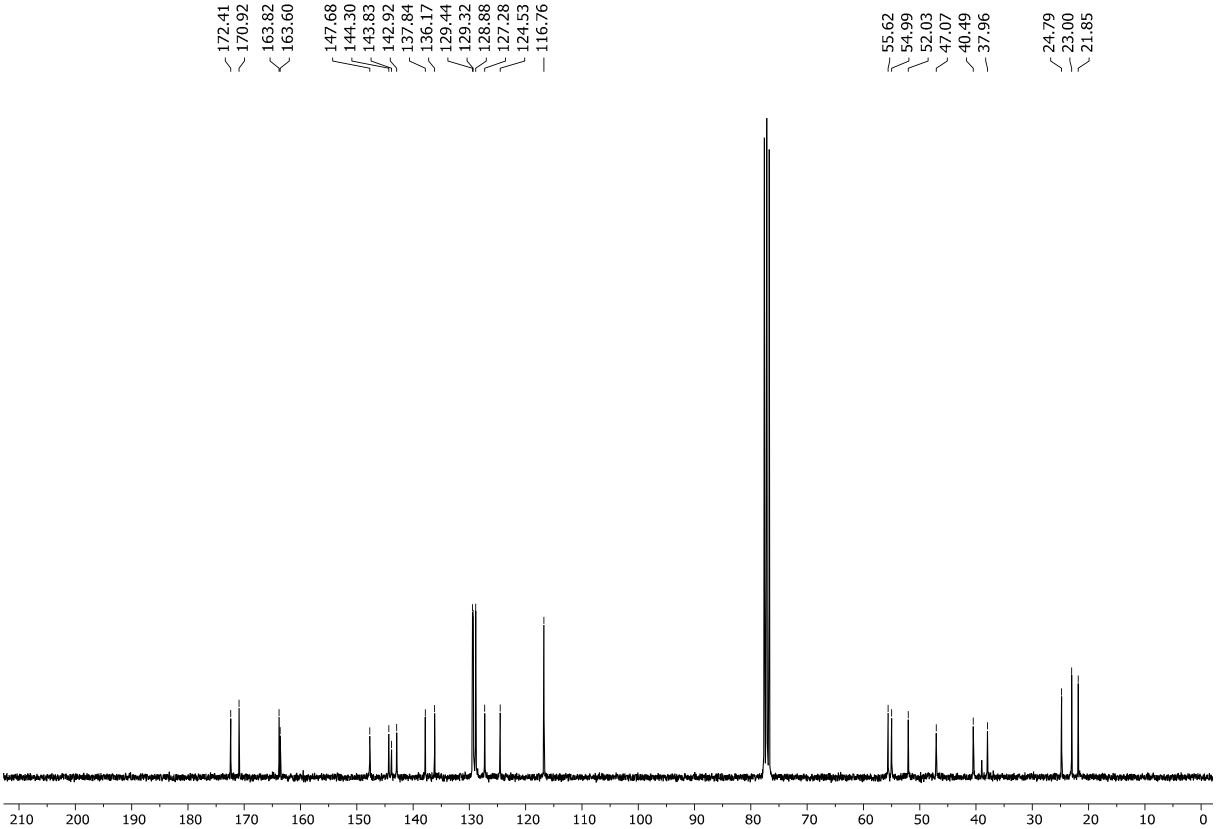

Figure S84. ^{13}C NMR of 72.

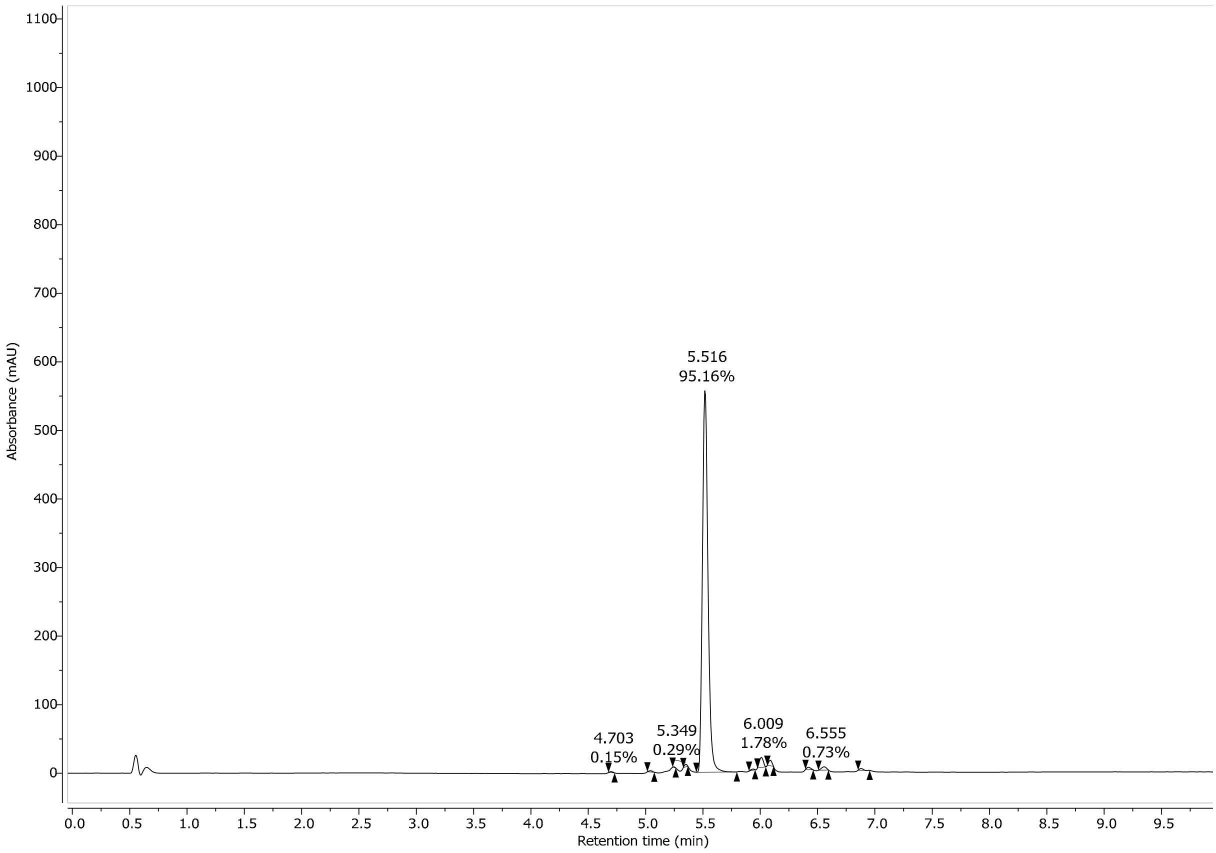

Figure S85. HPLC Chromatogram of 72 at 254 nm.

74, *N*-((*S*)-1-(((*S*)-1-Cyano-3-methylbutyl)amino)-1-oxo-3-phenylpropan-2-yl)pyrazine-2-carboxamide

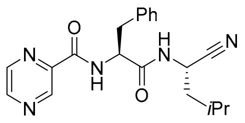

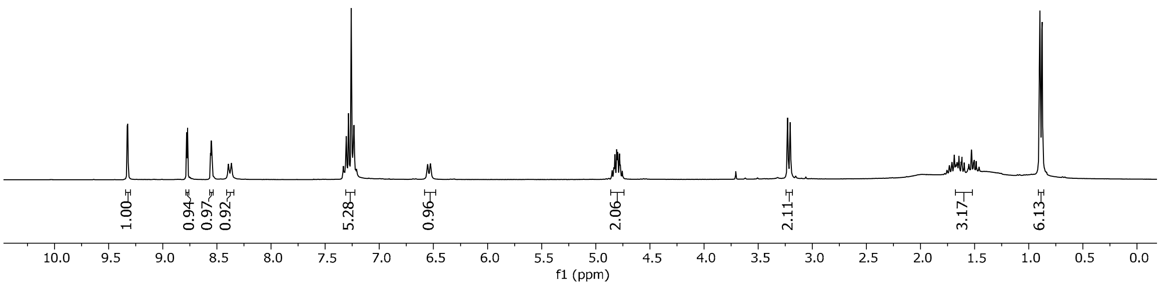

Figure S86. ^{1}H NMR of 74.

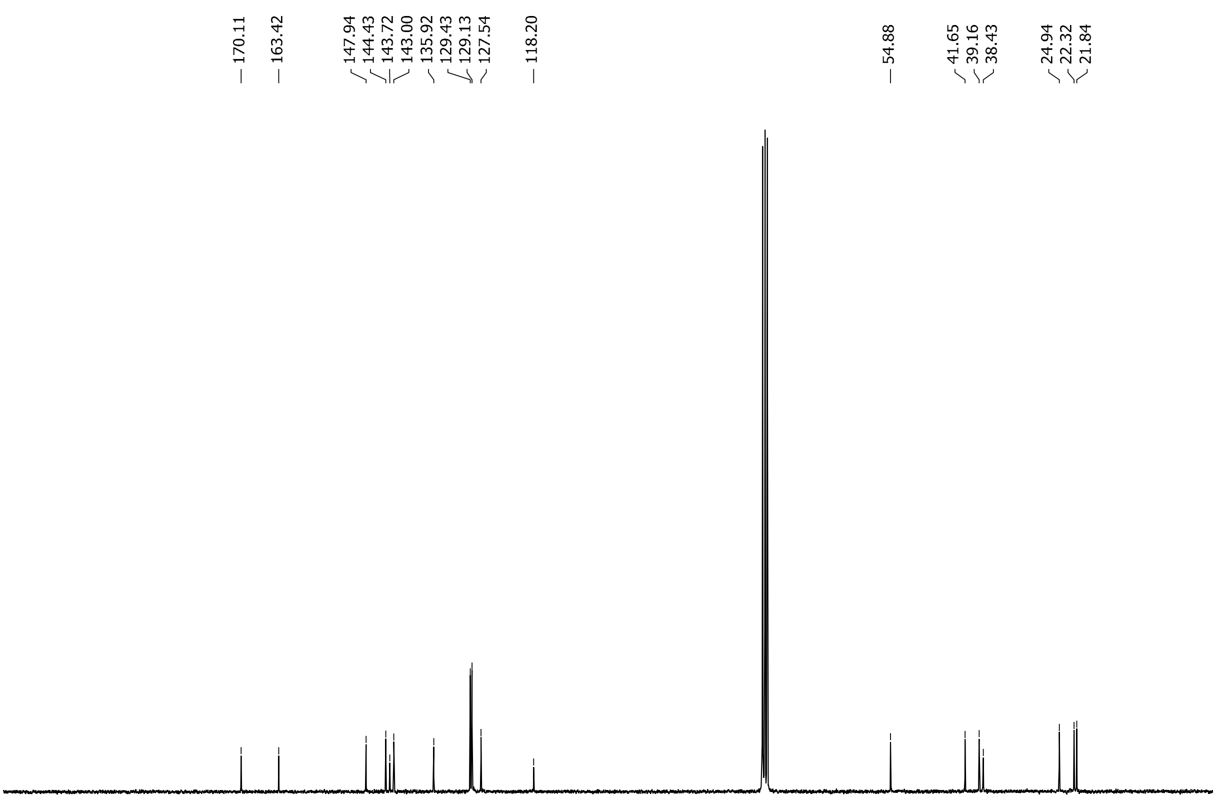

Figure S87. ^{13}C NMR of 74.

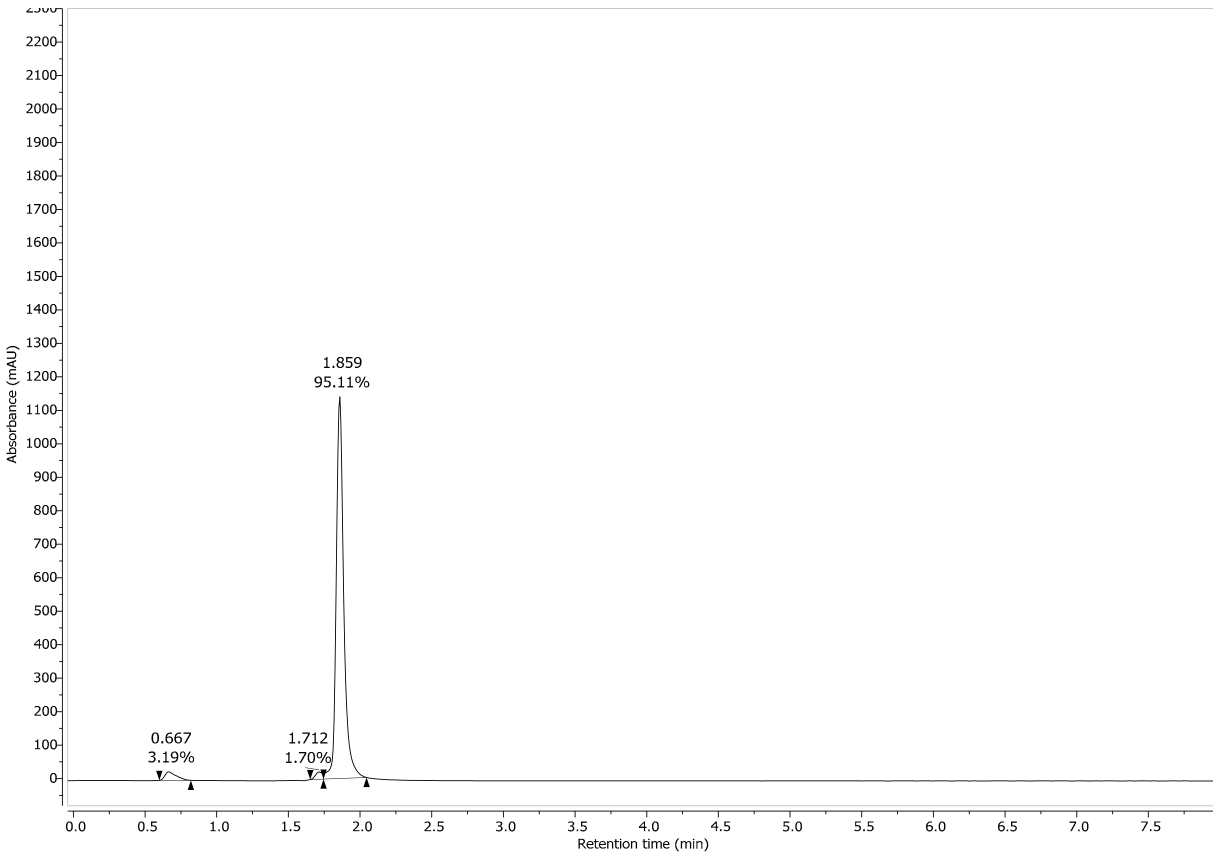

Figure S88. HPLC Chromatogram of 74 at 254 nm.

81, Ethyl (*S,E*)-5-((*S*)-2-(4-methoxy-1*H*-indole-2-carboxamido)-4-methylpentanamido)-4-oxo-6-((S)-2-oxopyrrolidin-3-yl)hex-2-enoate

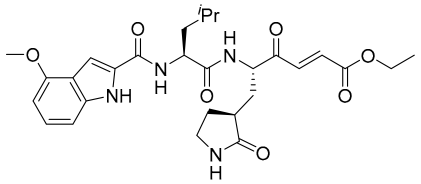

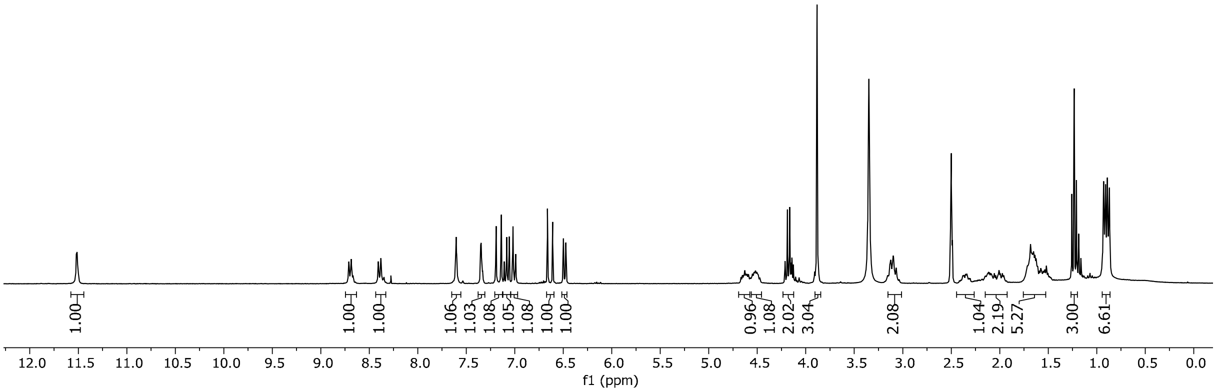

Figure S89. ^{1}H NMR of 81.

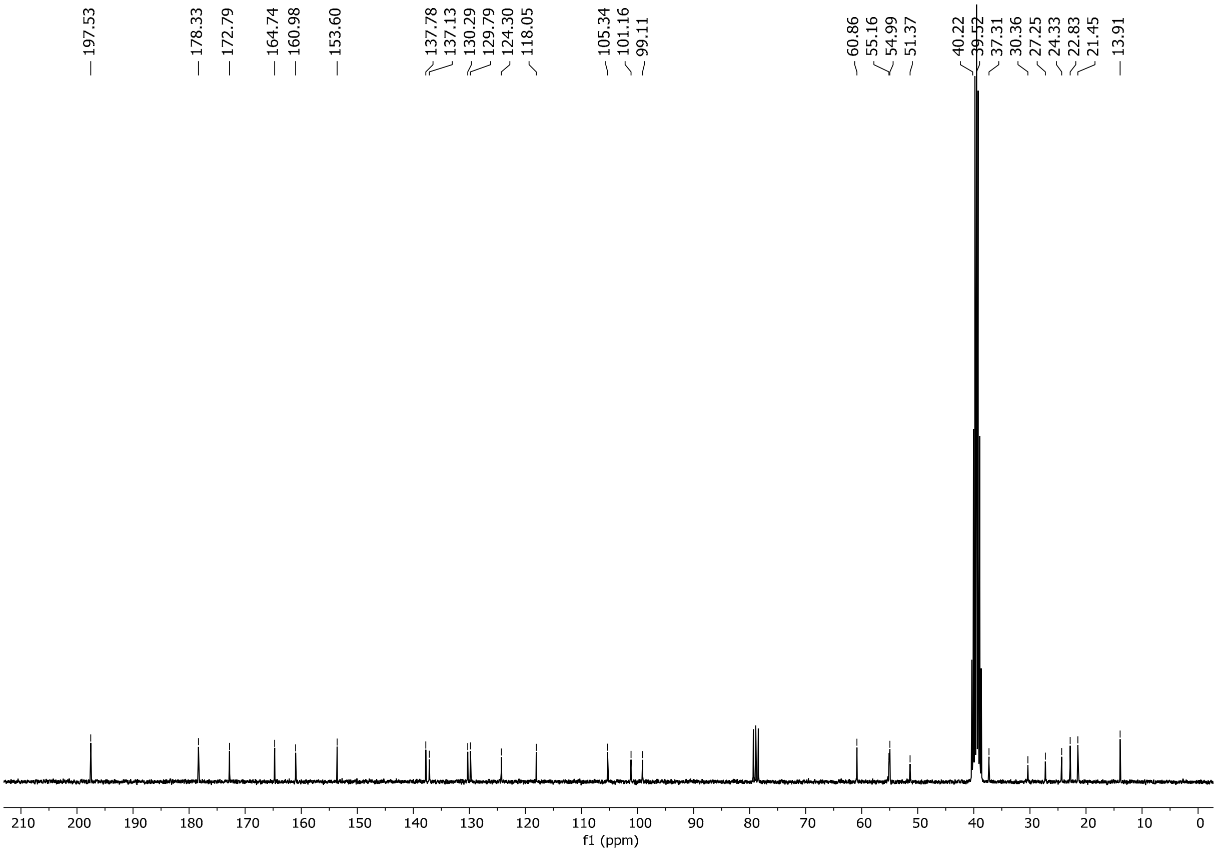

Figure S90. ^{13}C NMR of 81.

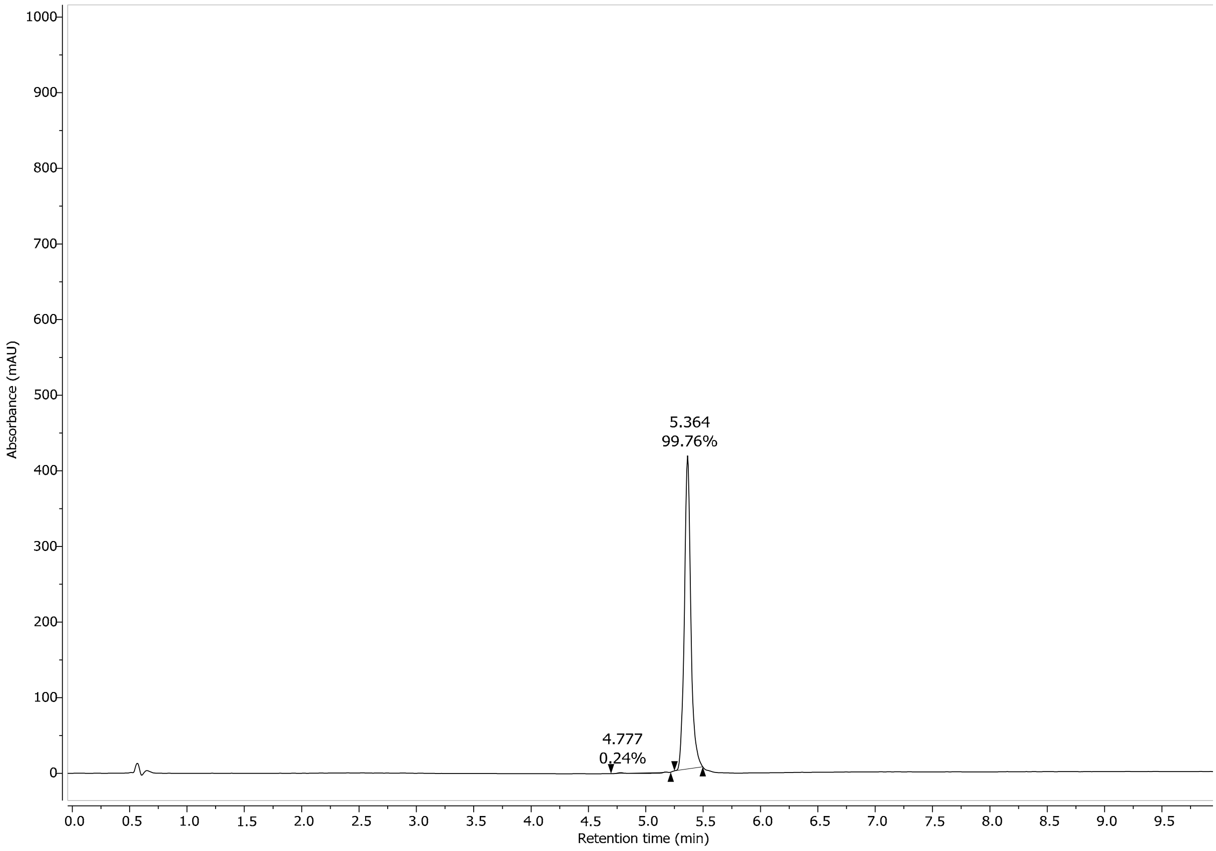

Figure S91. HPLC Chromatogram of 81 at 254 nm.

84, *N*-((*S*)-1-(((*S*)-1-Cyano-2-((*S*)-2-oxopyrrolidin-3-yl)ethyl)amino)-4-methyl-1-oxopentan-2-yl)-4-methoxy-1*H*-indole-2-carboxamide

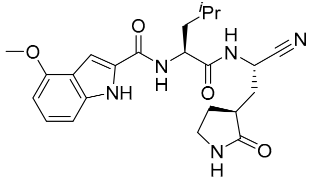

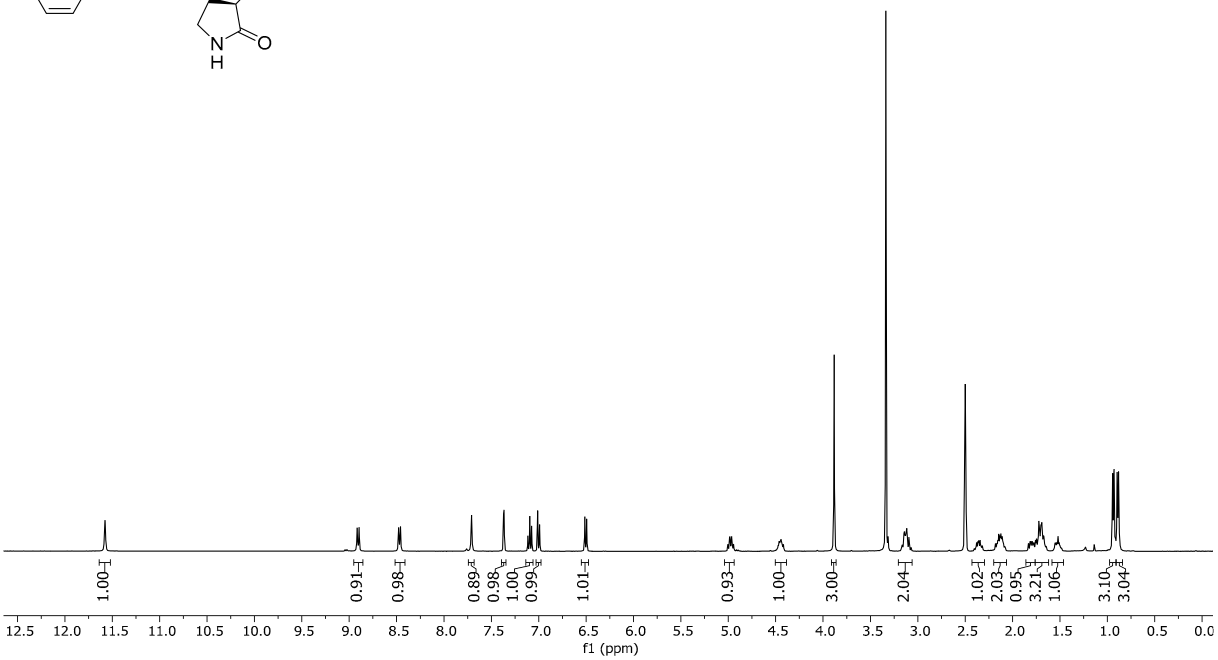

Figure S92. ^{1}H NMR of 84.

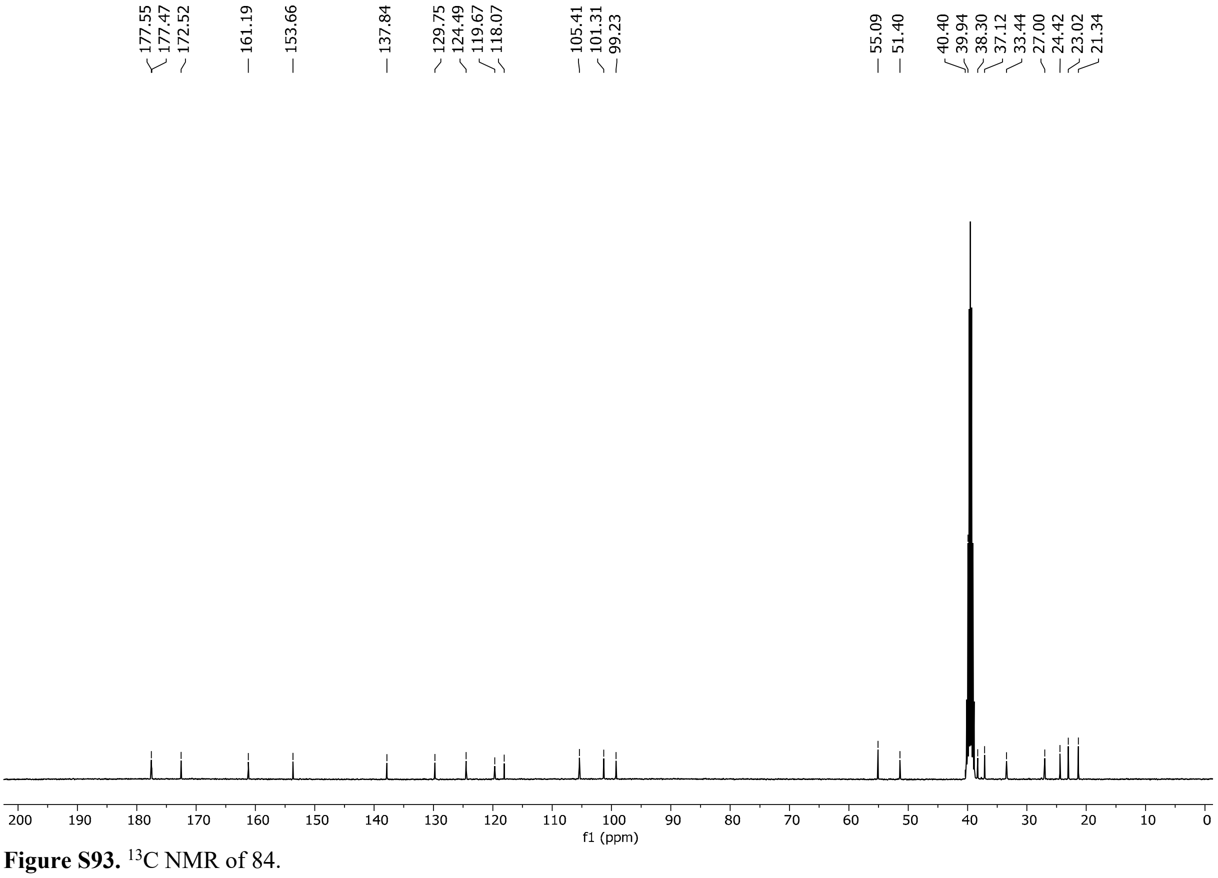

Figure S93. ^{13}C NMR of 84.

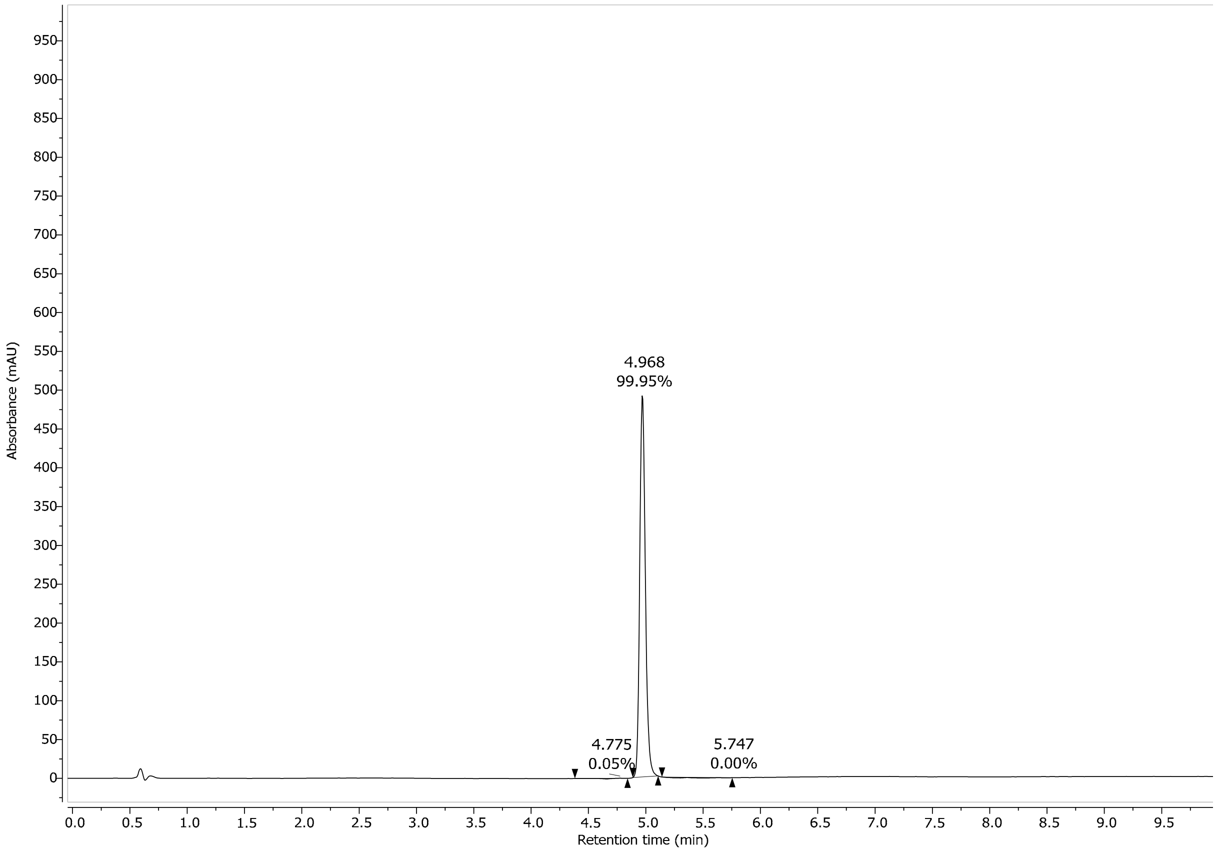

Figure S94. HPLC Chromatogram of 84 at 254 nm.

88, 4-Methoxy-*N*-((*S*)-4-methyl-1-(((*S*,*E*)-4-nitro-1-((*S*)-2-oxopyrrolidin-3-yl)but-3-en-2-yl)amino)-1-oxopentan-2-yl)-1*H*-indole-2-carboxamide

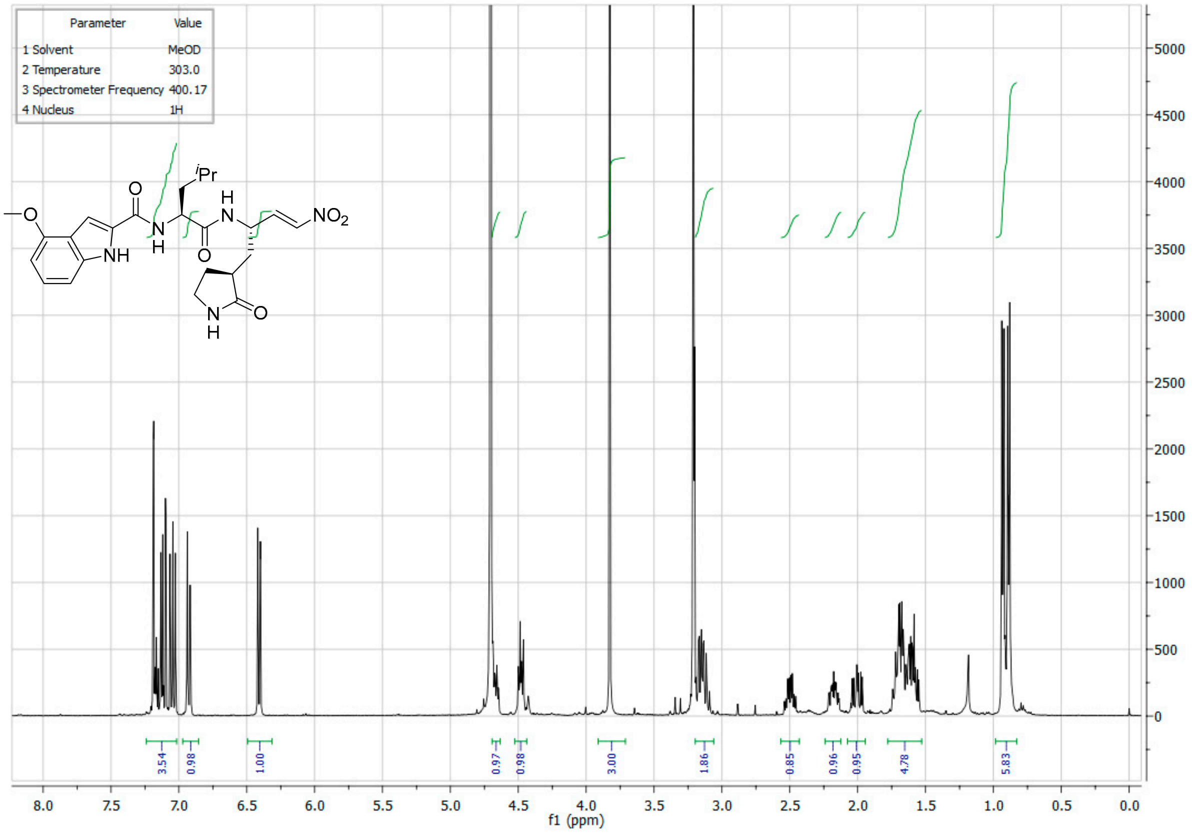

Figure S95. ^{1}H NMR of 88.

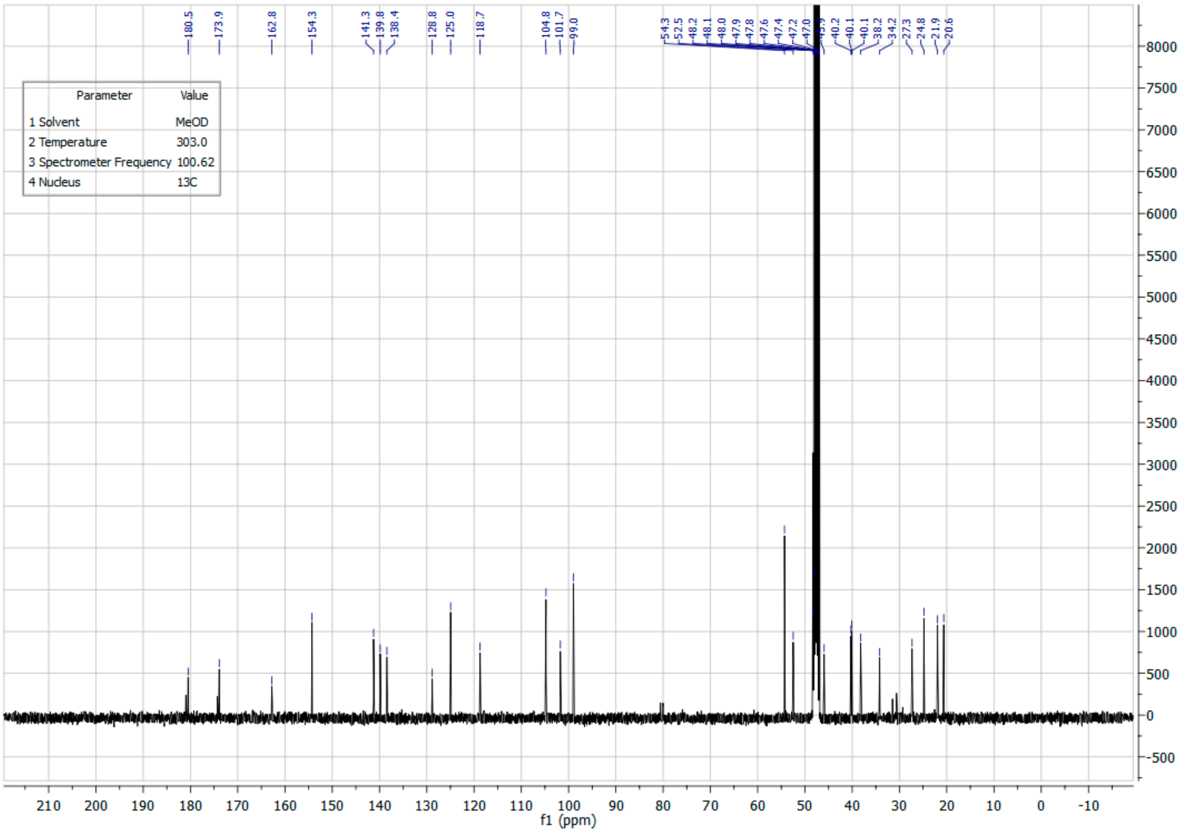

Figure S96. ^{13}C NMR of 88.

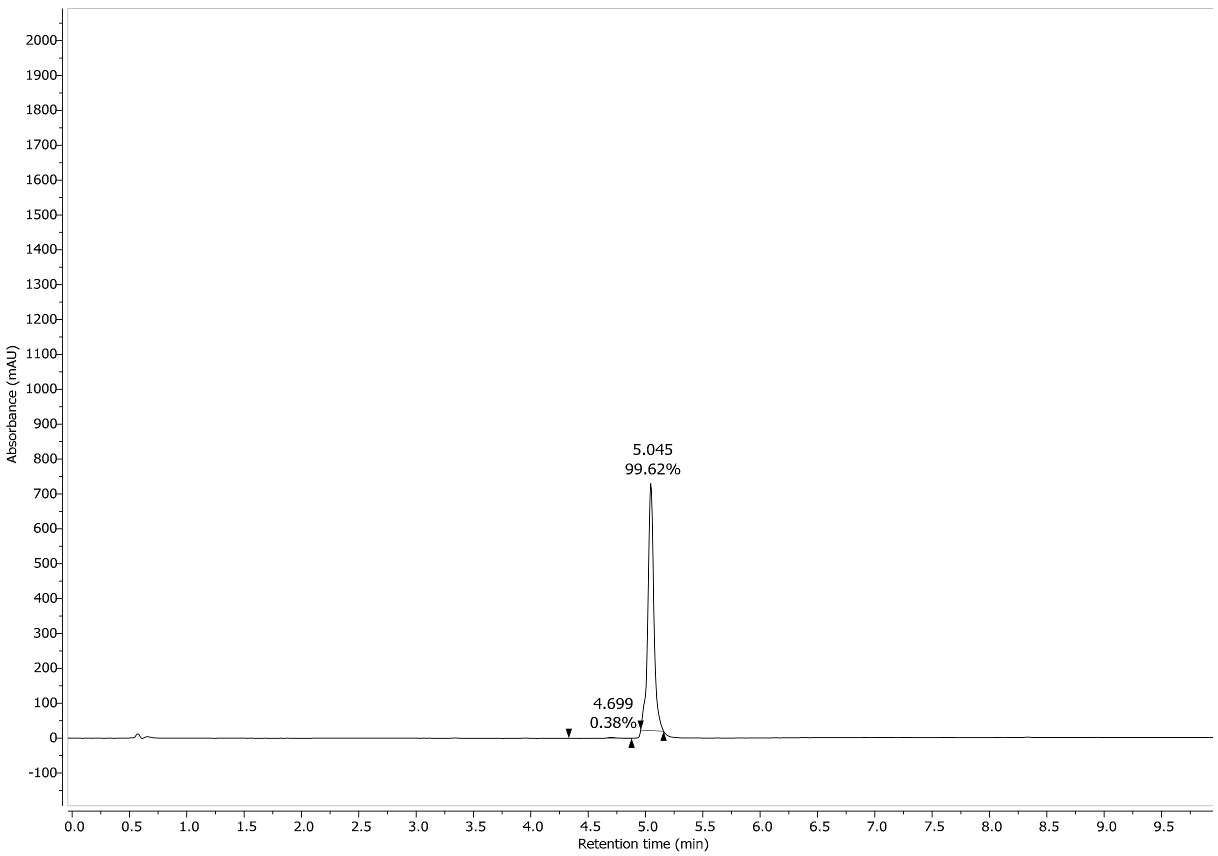

Figure S97. HPLC Chromatogram of 88 at 254 nm.

90, *N*-((*S*)-1-(((*S*)-1-(Benzo[*d*]thiazol-2-yl)-1-oxo-3-((*S*)-2-oxopyrrolidin-3-yl)propan-2-yl)amino)-4-methyl-1-oxopentan-2-yl)-4-methoxy-1*H*-indole-2-carboxamide

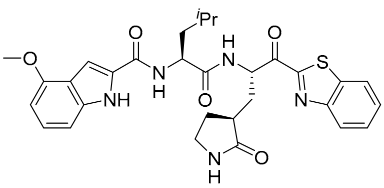

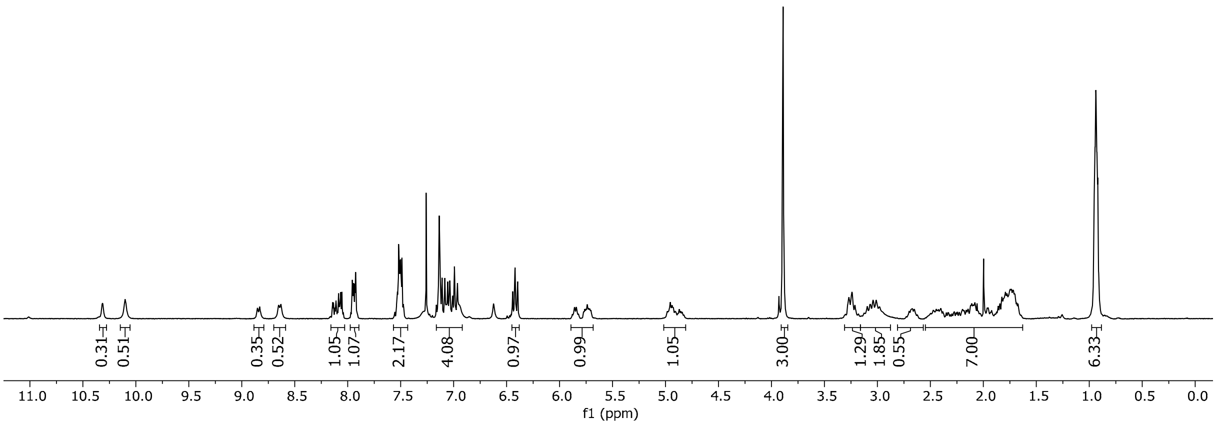

Figure S98. ^{1}H NMR of 90.

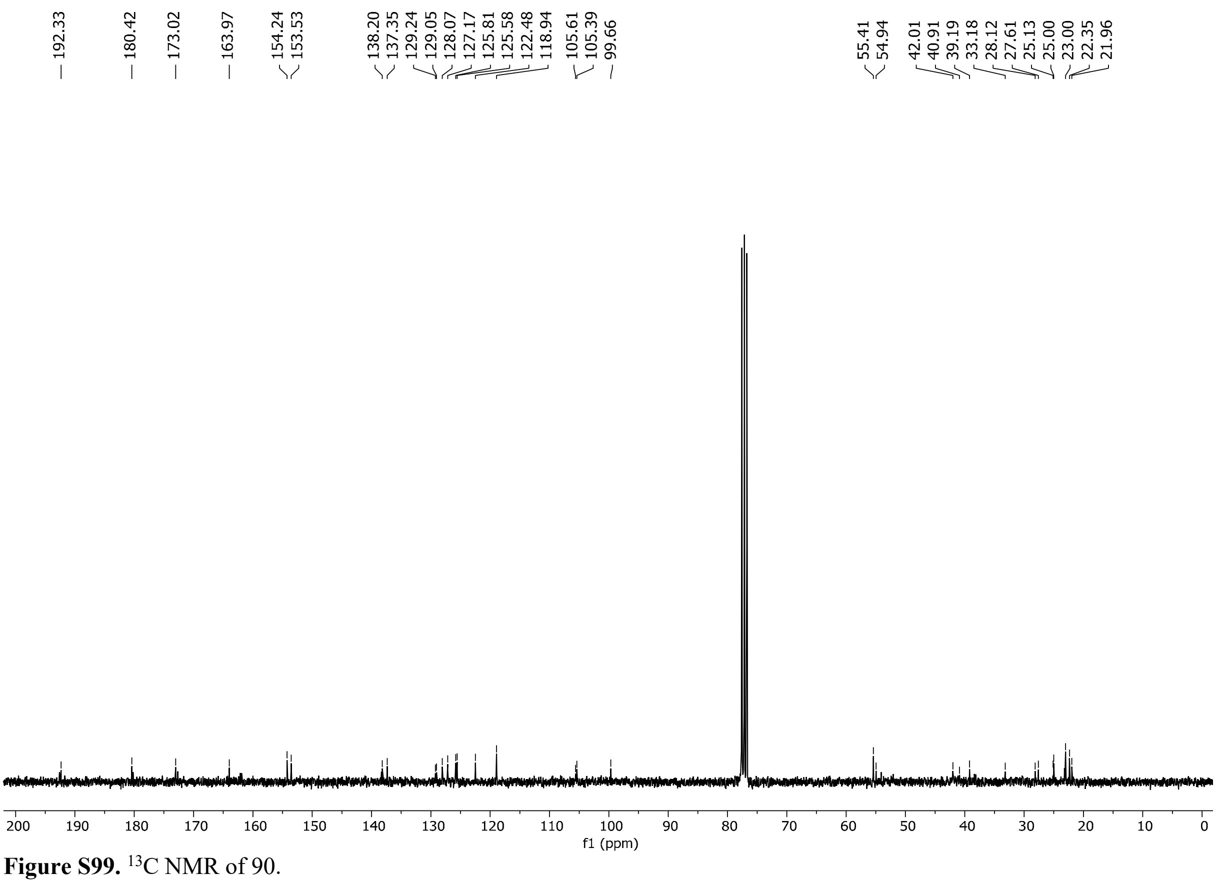

Figure S99. ^{13}C NMR of 90.

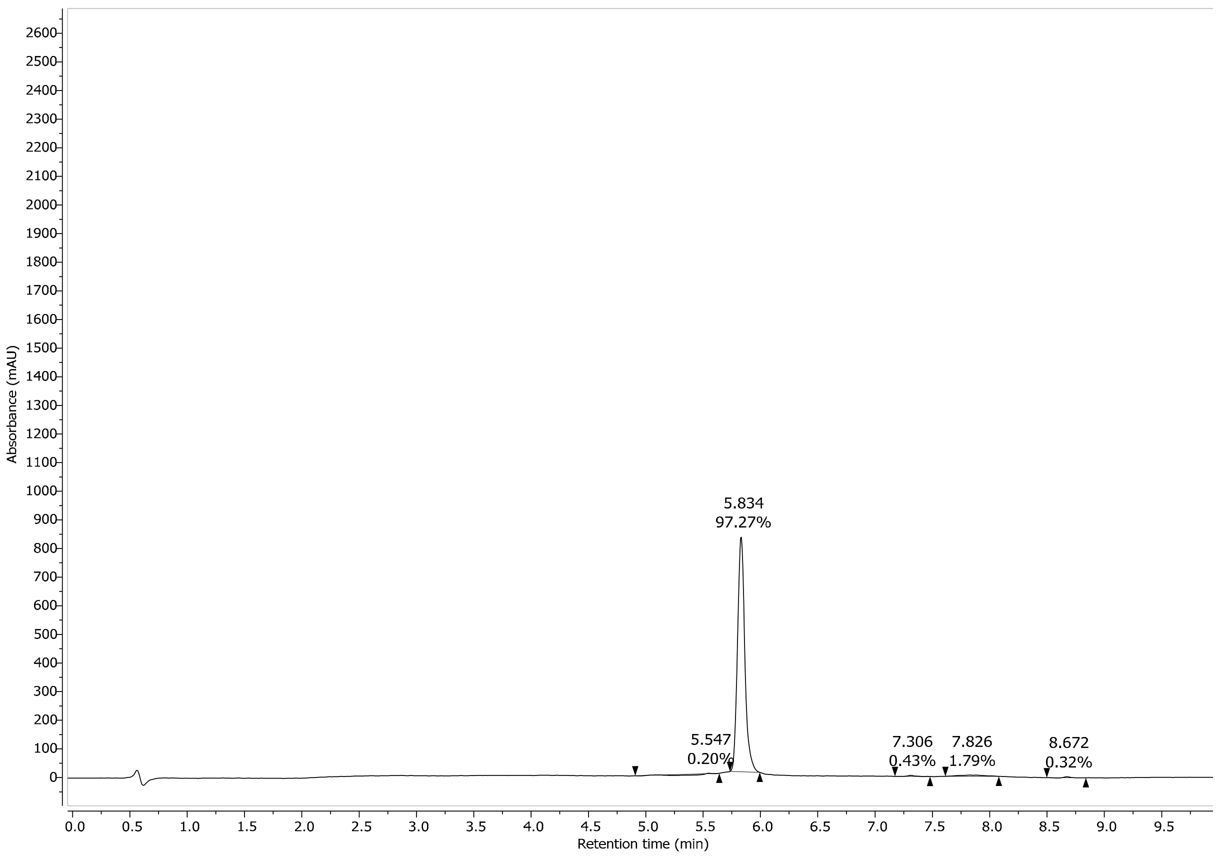

Figure S100. HPLC Chromatogram of 90 at 210 nm.

93, 4-Methoxy-*N*-((*S*)-4-methyl-1-oxo-1-(((*S*,*E*)-1-((S)-2-oxopyrrolidin-3-yl)-4-(phenylsulfonyl)but-3-en-2-yl)amino)pentan-2-yl)-1*H*-indole-2-carboxamide

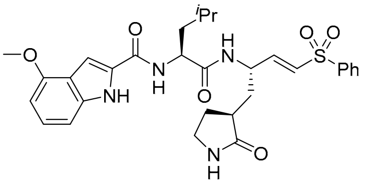

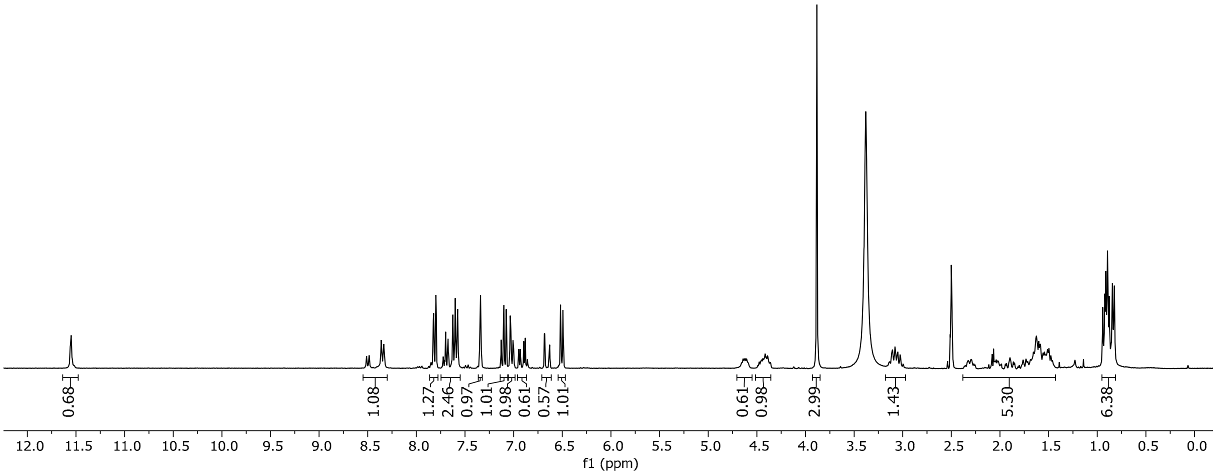

Figure S101. ^{1}H NMR of 93.

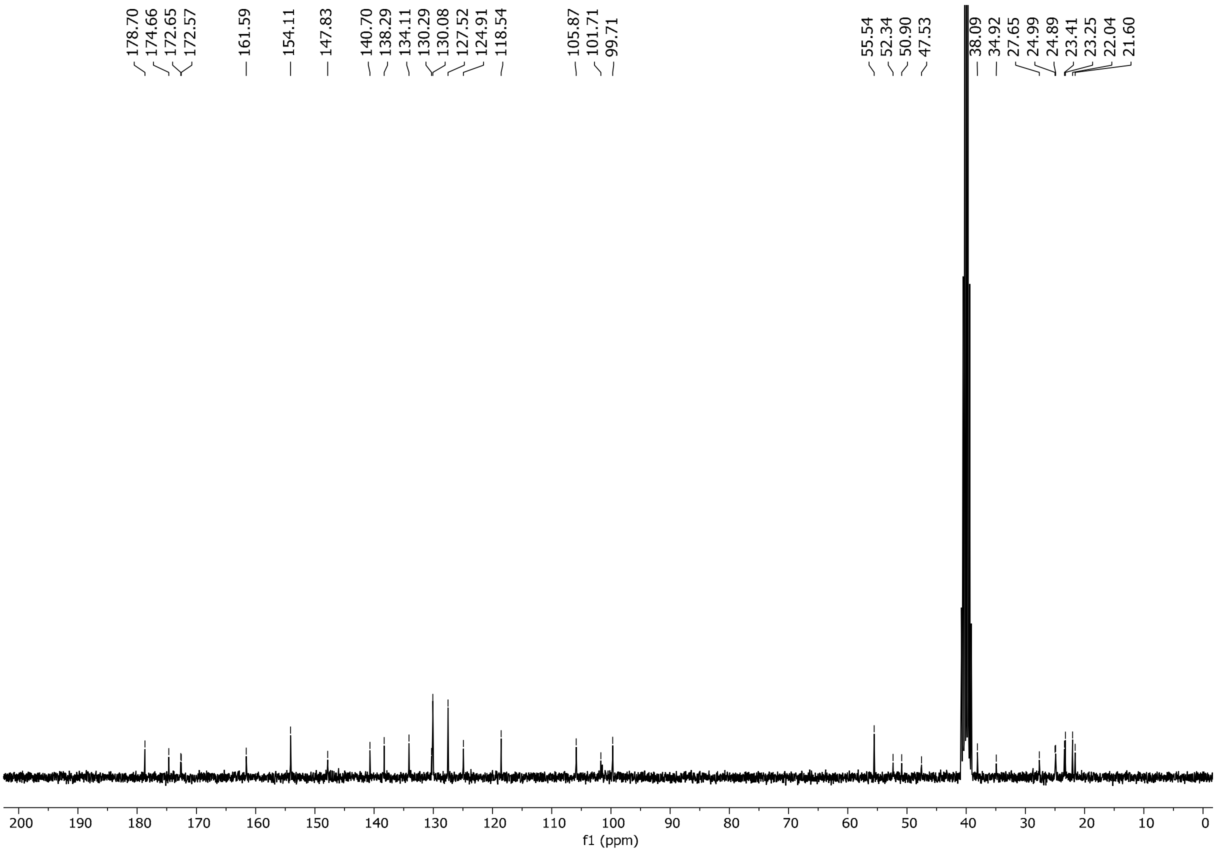

Figure S102. 13C NMR of 93.

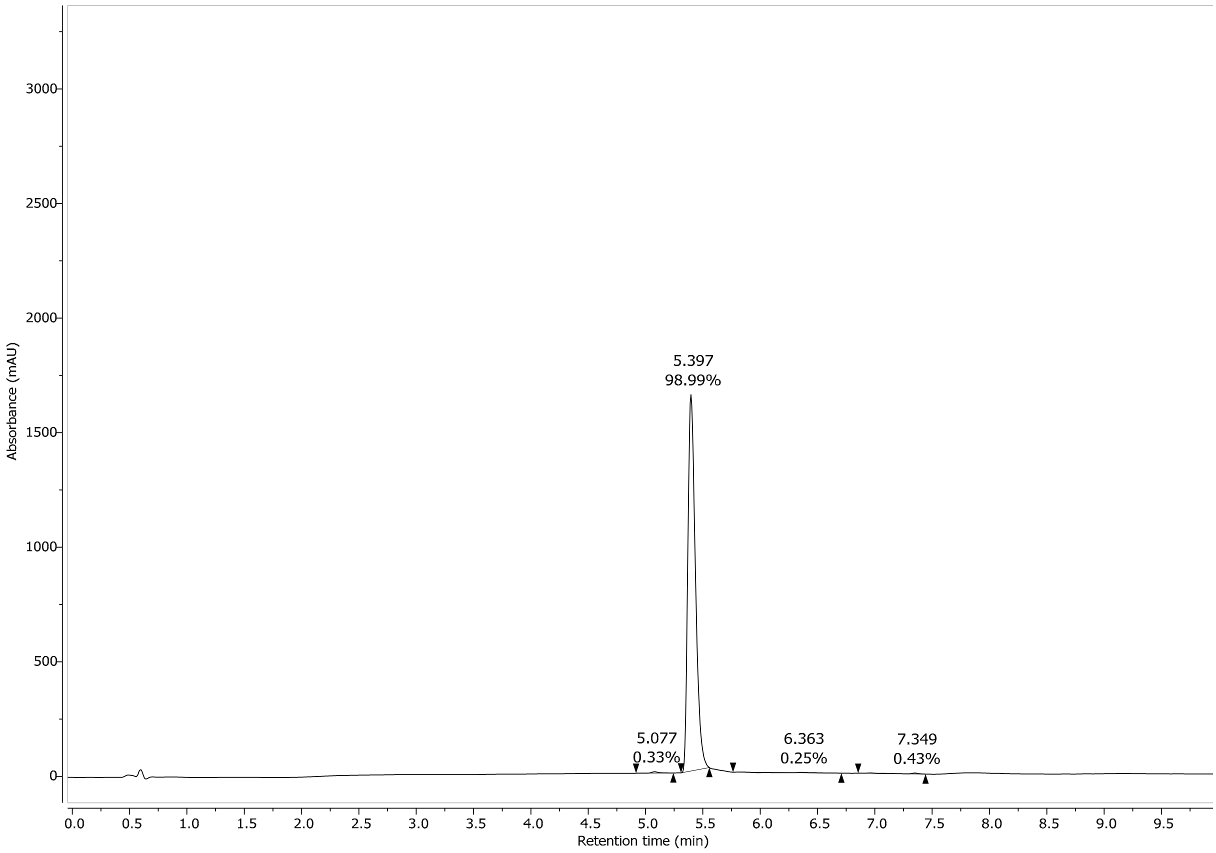

Figure S103. HPLC Chromatogram of 90 at 210 nm.

94, *N*-((*S*)-1-(((*S*,*E*)-4-Fluoro-1-((*S*)-2-oxopyrrolidin-3-yl)-4-(phenylsulfonyl)but-3-en-2-yl)amino)-4-methyl-1-oxopentan-2-yl)-4-methoxy-1*H*-indole-2-carboxamide

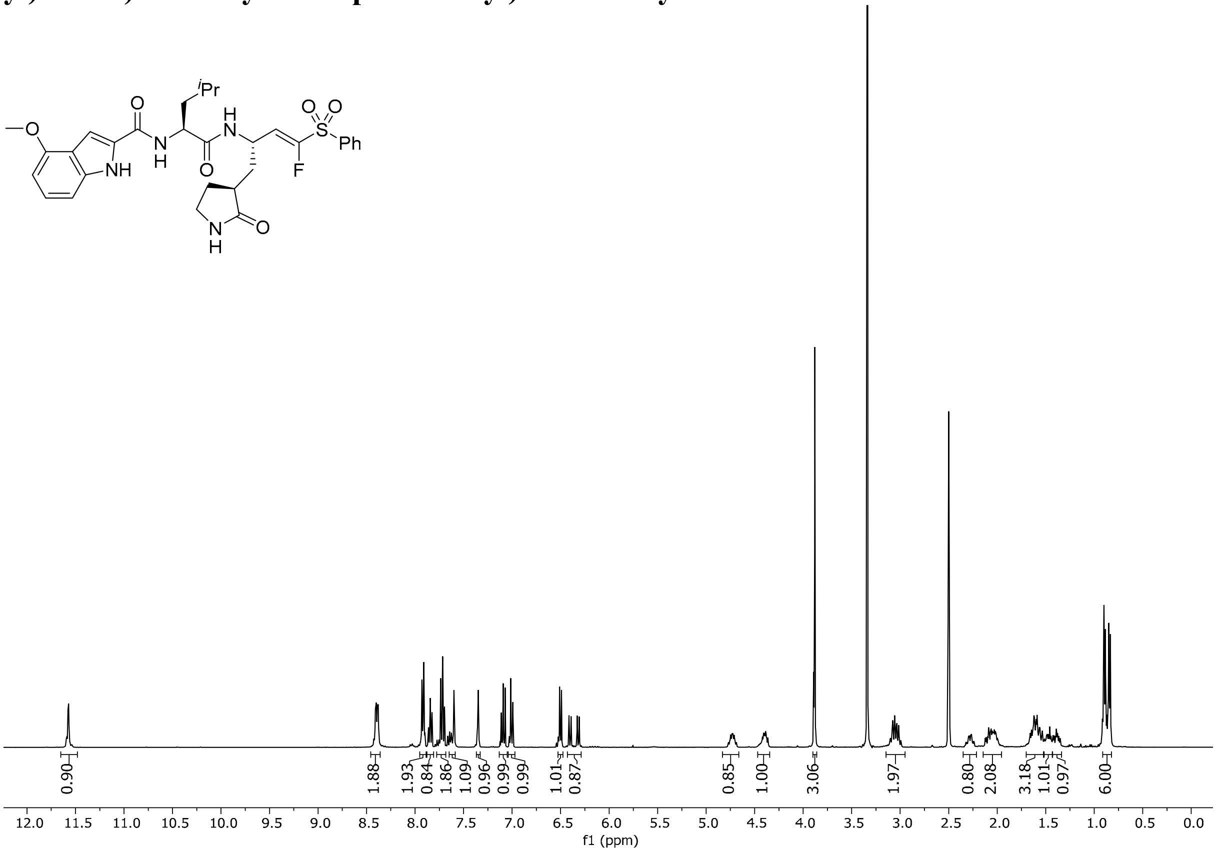

Figure S104. ^{1}H NMR of 94.

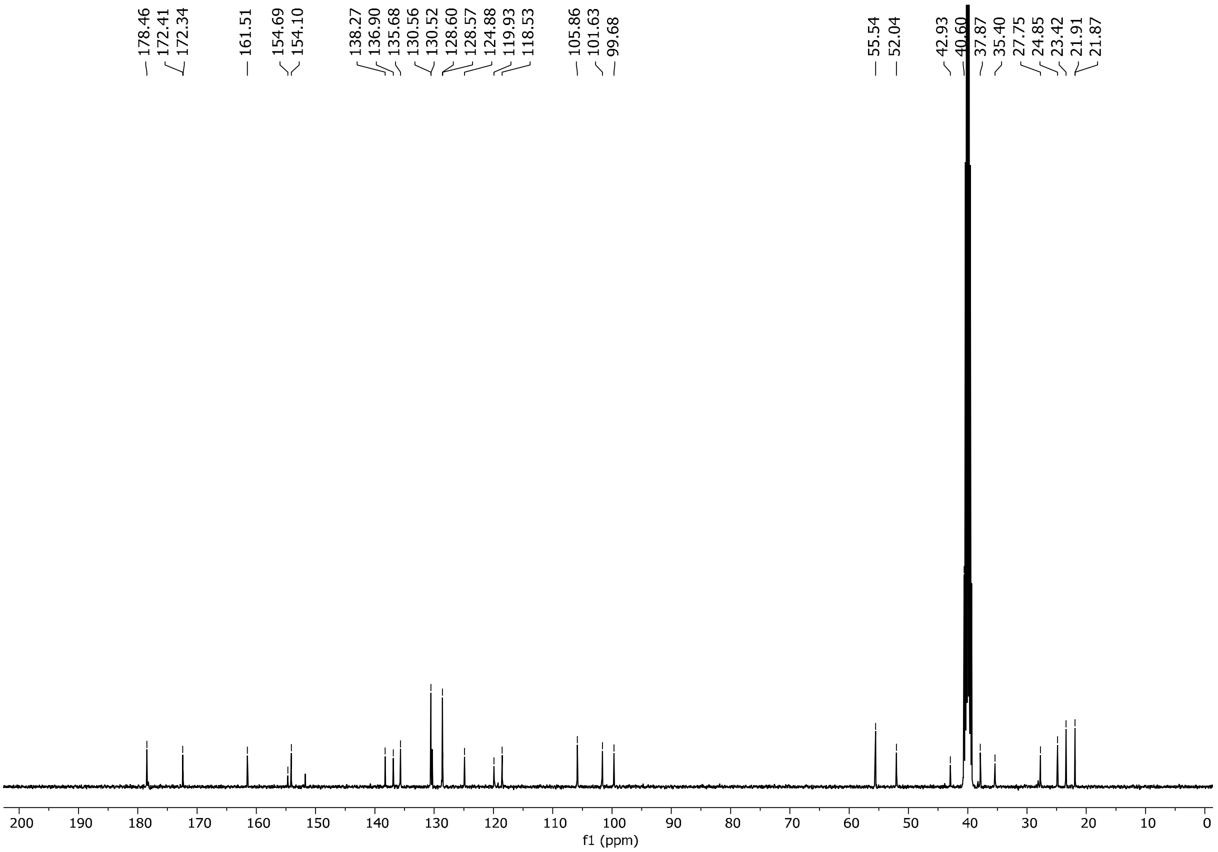

Figure S105. ^{13}C NMR of 94.

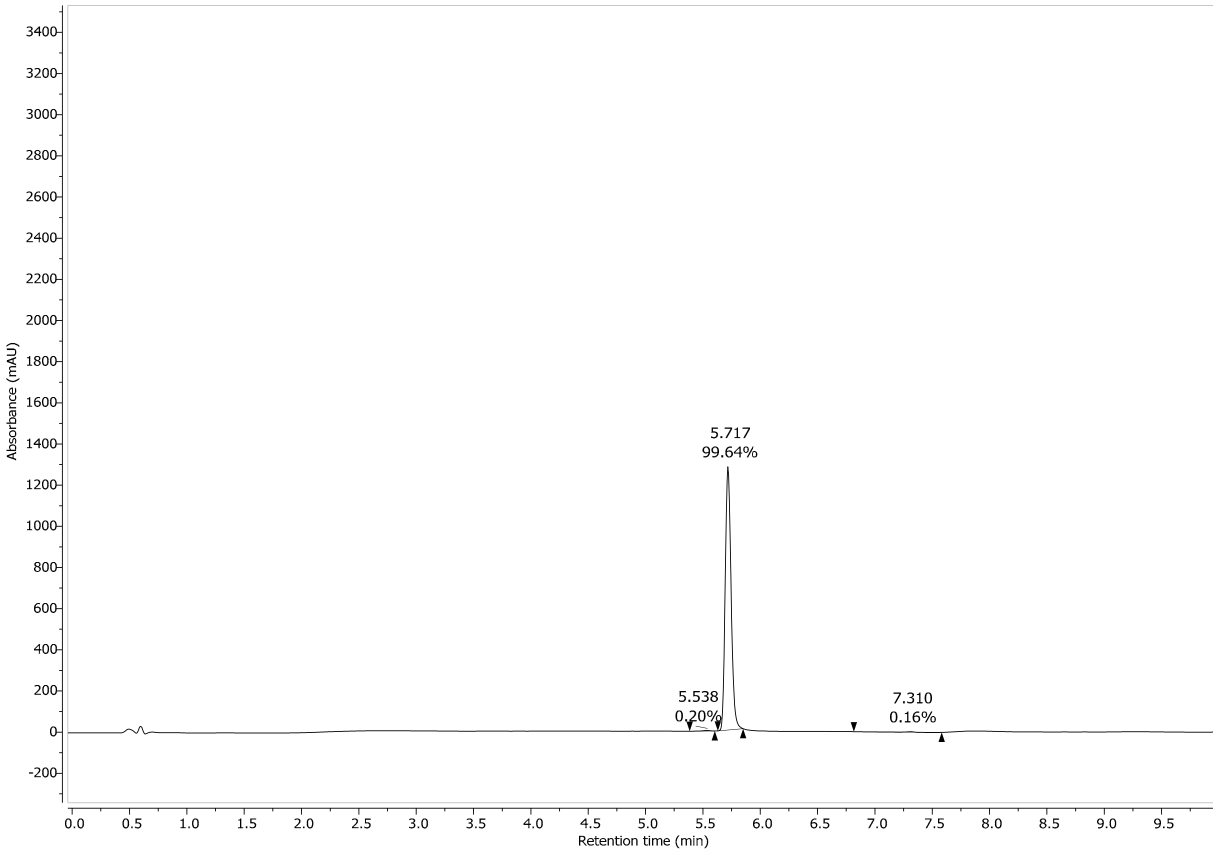

Figure S106. HPLC Chromatogram of 94 at 210 nm.

96, 4-Methoxy-*N*-((2*S*)-4-methyl-1-oxo-1-(((2*S*)-1-oxo-1-((2-oxo-1-phenylazetidin-3-yl)amino)-3-((*S*)-2-oxopyrrolidin-3-yl)propan-2-yl)amino)pentan-2-yl)-1*H*-indole-2-carboxamide

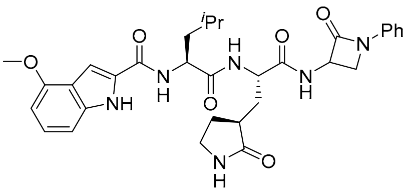

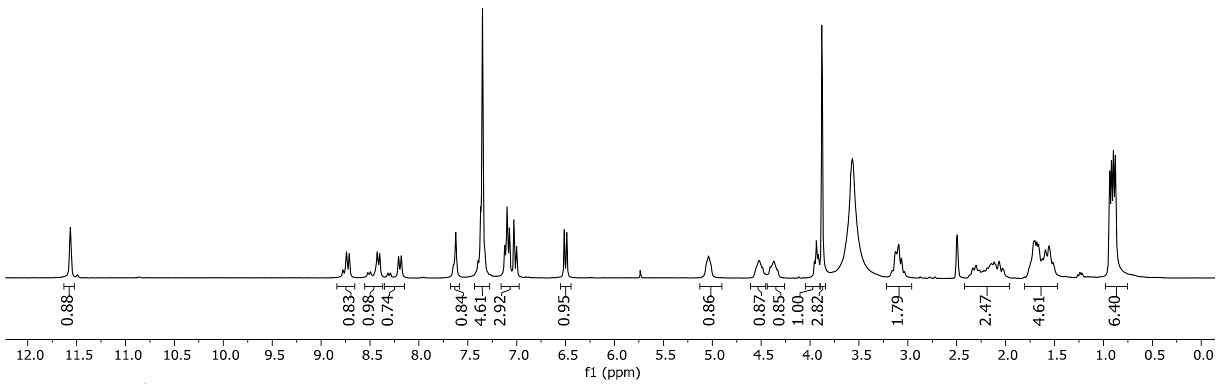

Figure S107. ^{1}H NMR of 96.

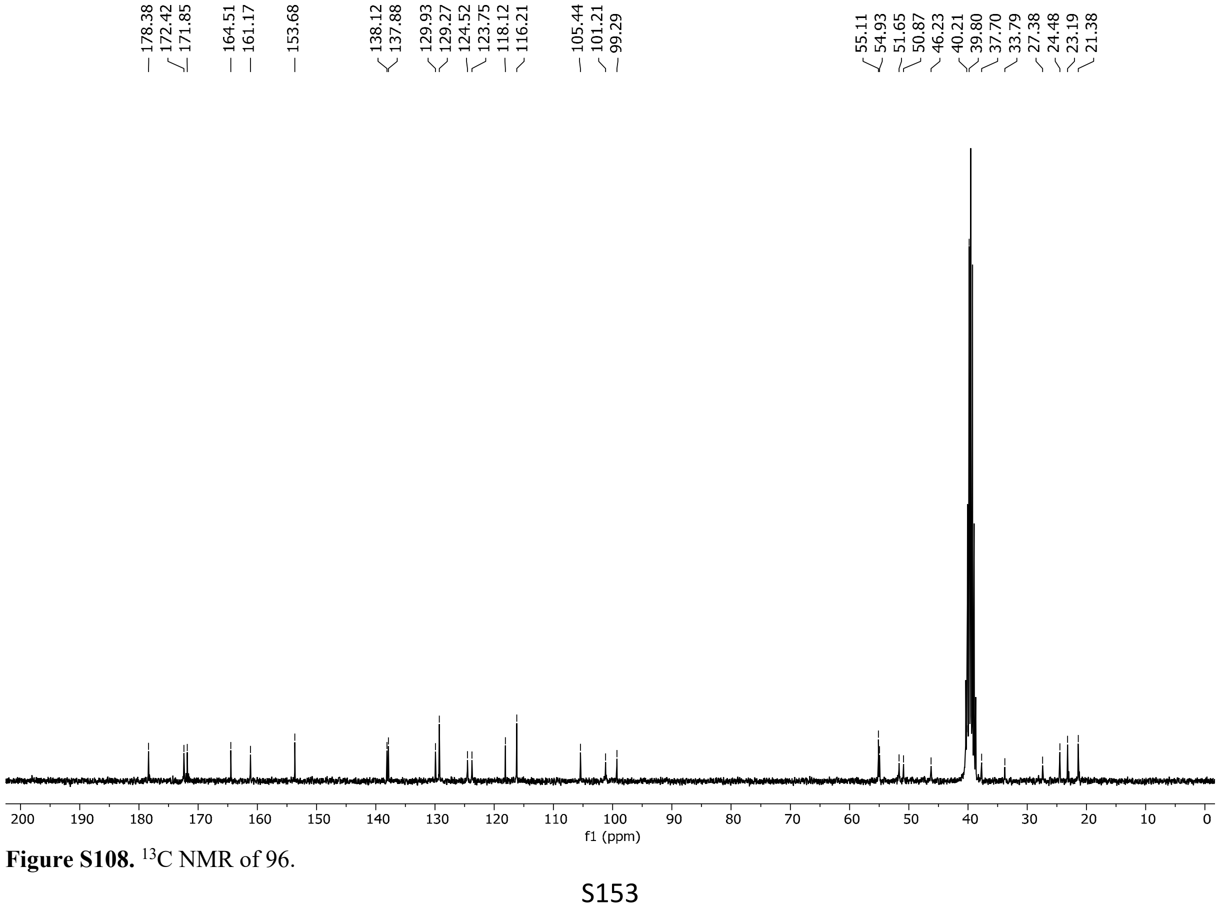

Figure S108. ^{13}C NMR of 96.

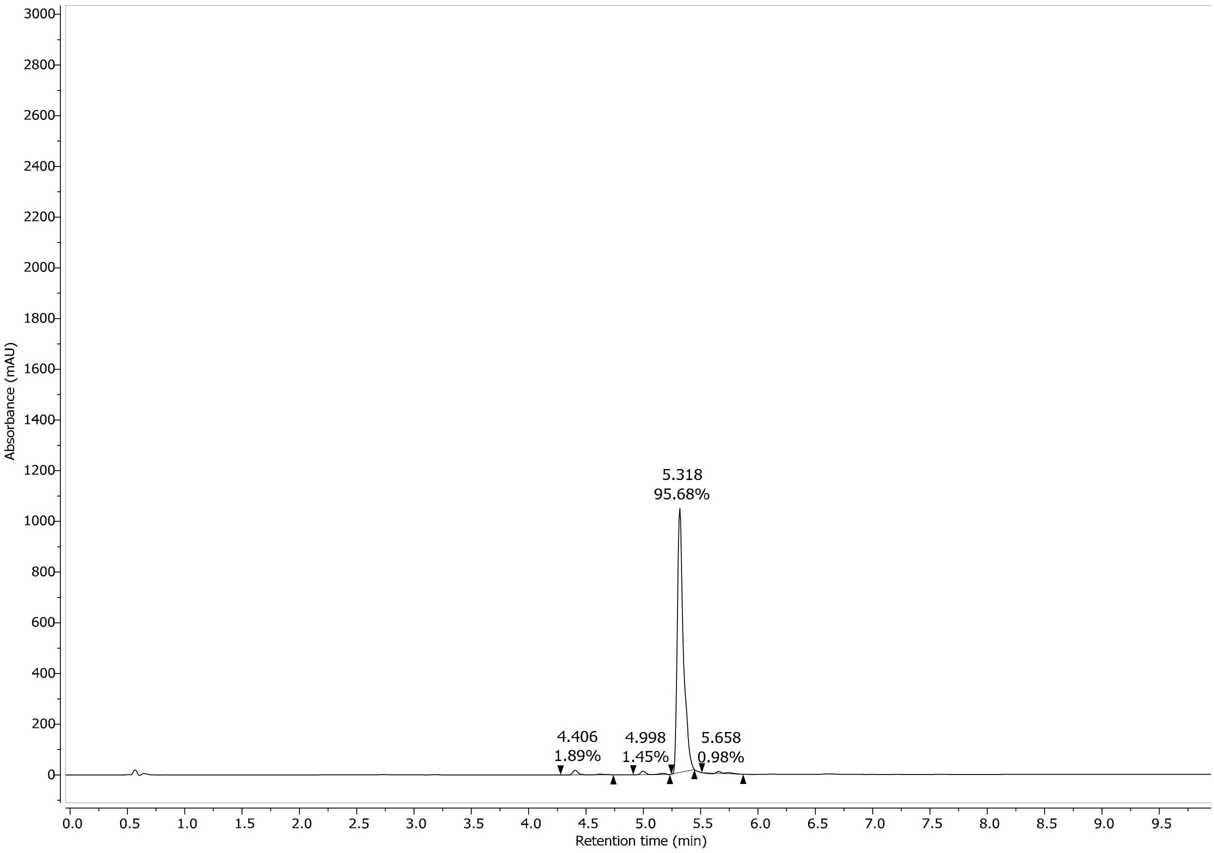

Figure S109. HPLC Chromatogram of 96 at 210 nm.

103, (2*S*,3*S*)-2-(2-Acetamidoacetamido)-*N*-((*S*)-1-(((*S*)-1-(benzo[*d*]thiazol-2-yl)-5-guanidino-1-oxopentan-2-yl)amino)-1-oxopropan-2-yl)-3-hydroxybutanamide

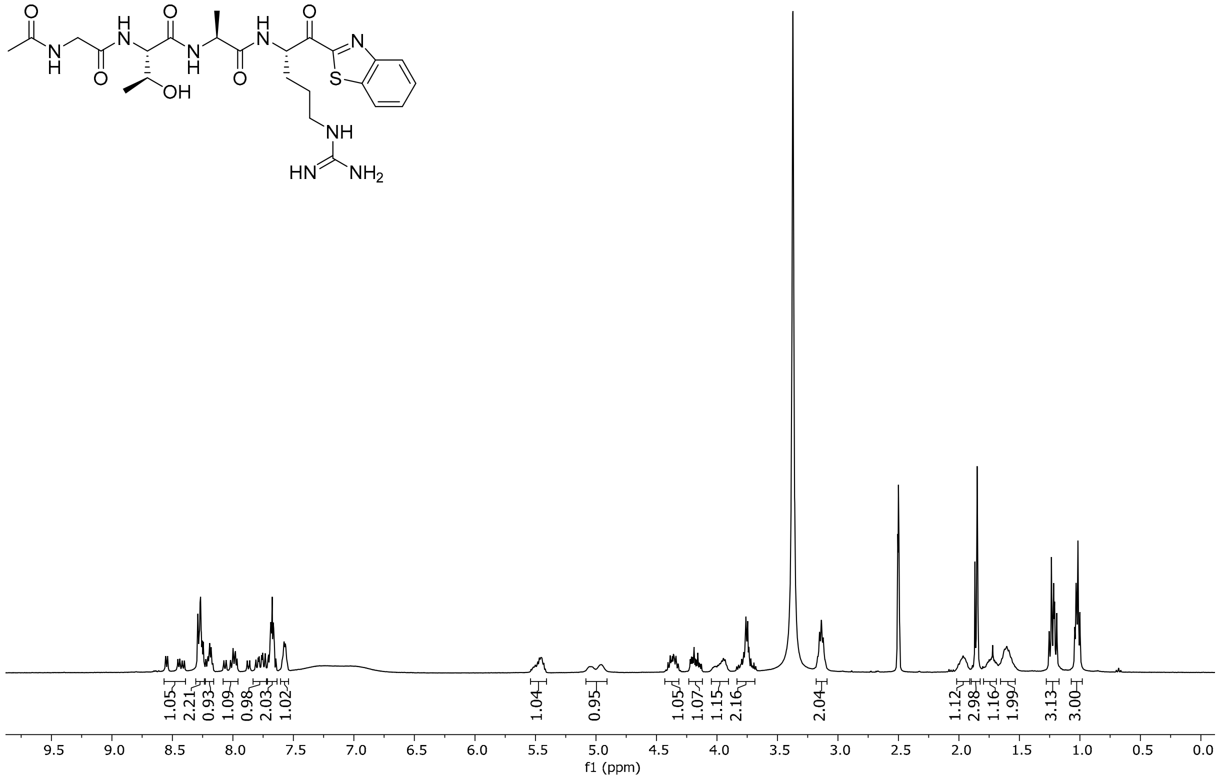

Figure S110. ^{1}H NMR of 103.

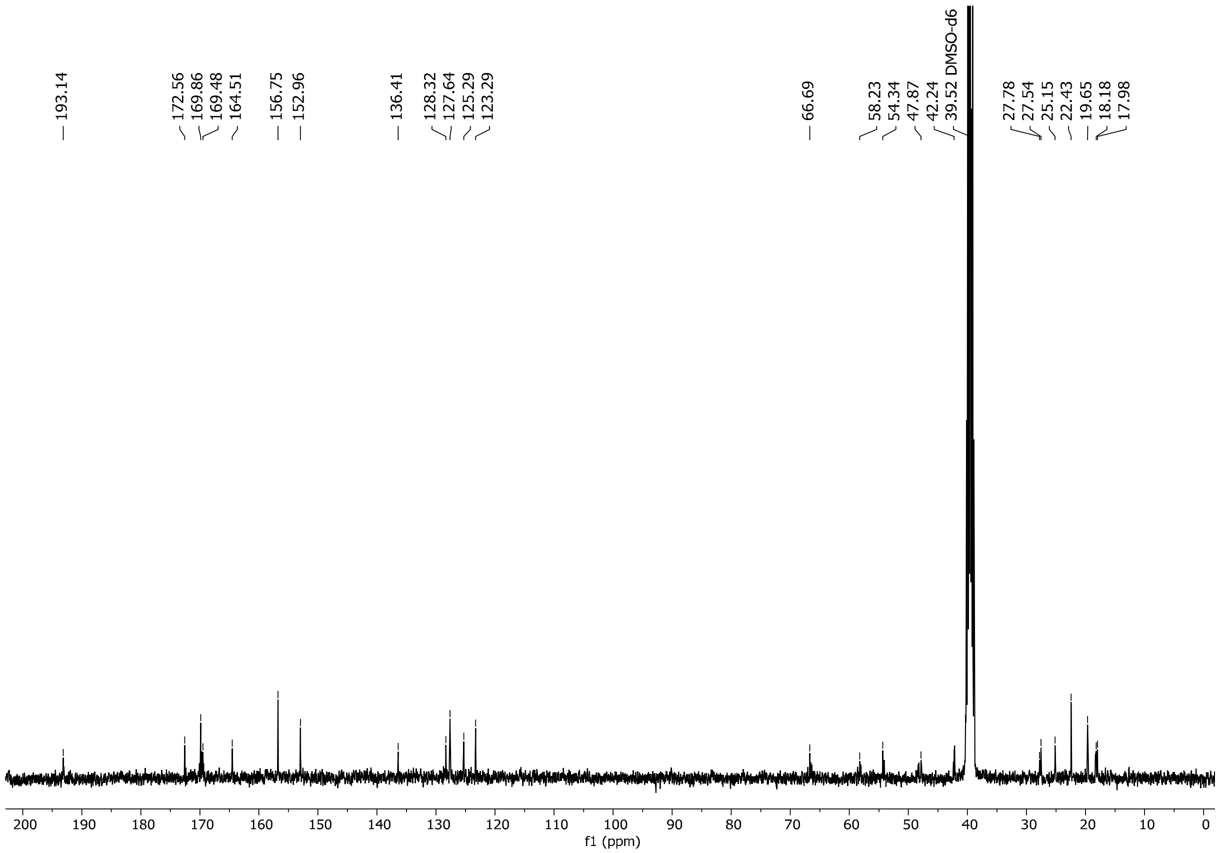

Figure S111. ^{13}C NMR of 103.

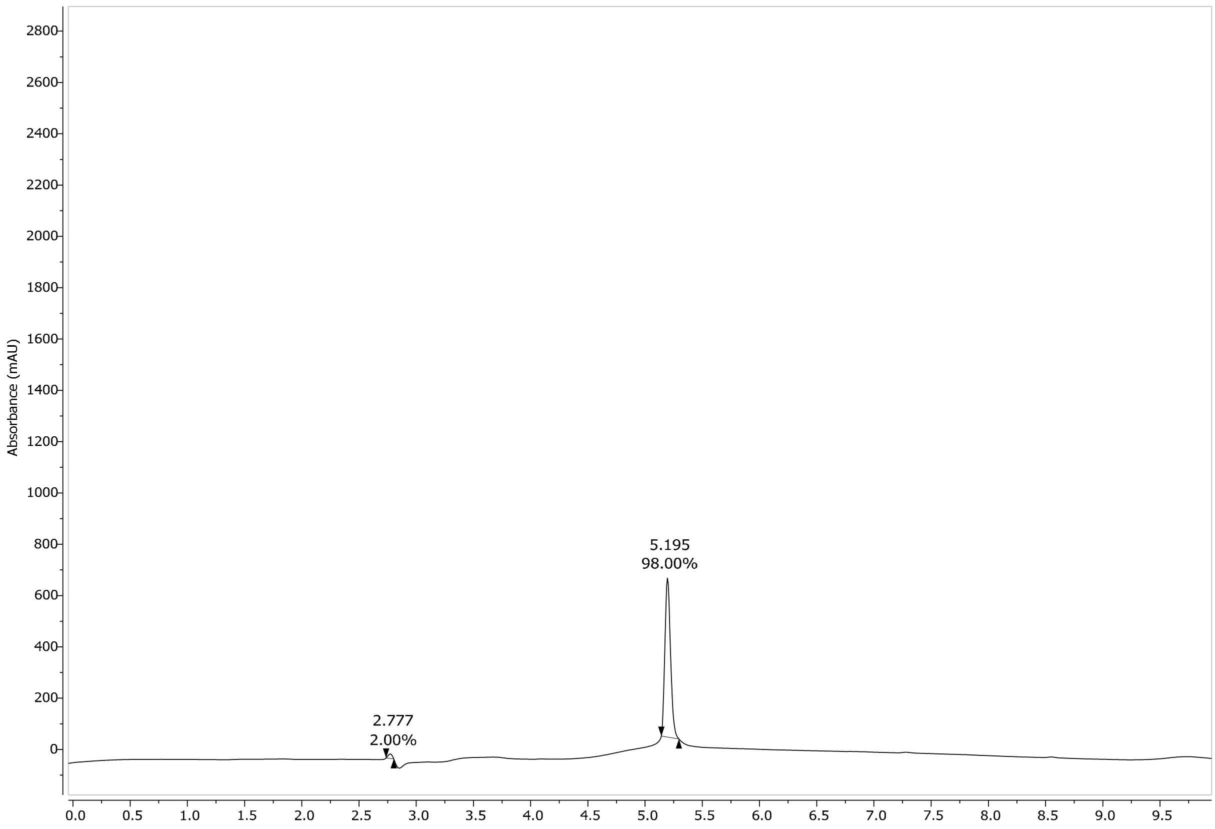

Figure S112. HPLC Chromatogram of 103 at 210 nm.

106, (2*S*,3*S*)-2-(2-acetamidoacetamido)-N-((*S*)-1-(((*S*,*E*)-6-guanidino-1-(phenylsulfonyl)hex-1-en-3-yl)amino)-1-oxopropan-2-yl)-3-hydroxybutanamide

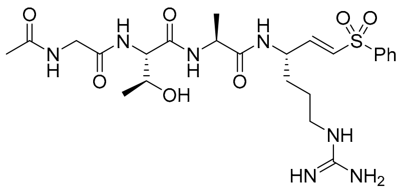

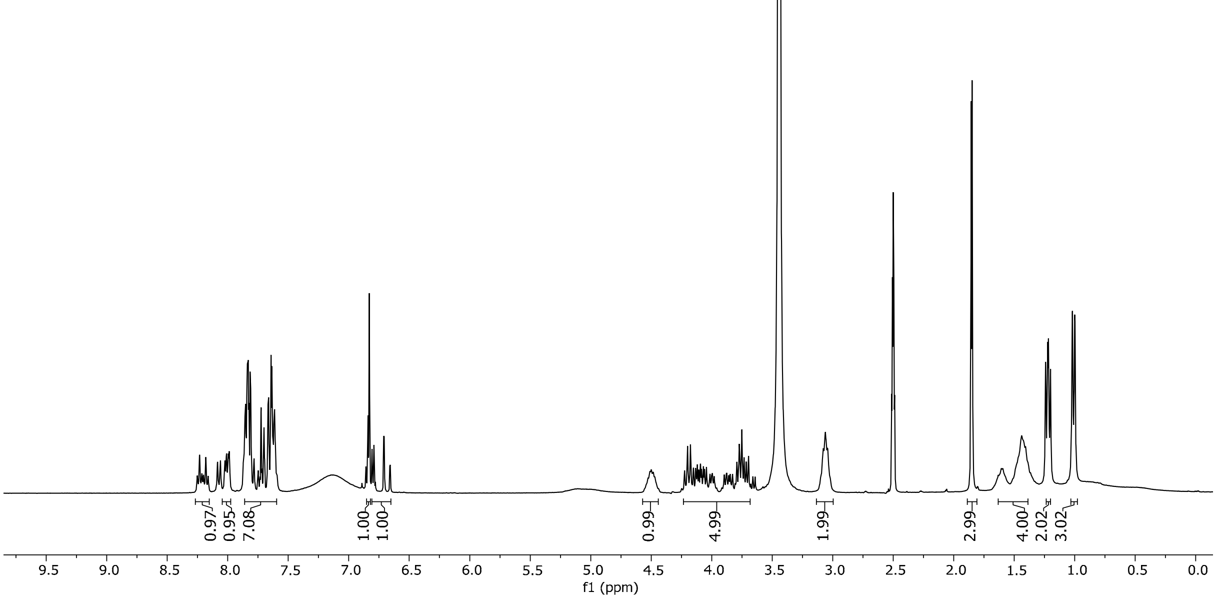

Figure S113. ^{1}H NMR of 106.

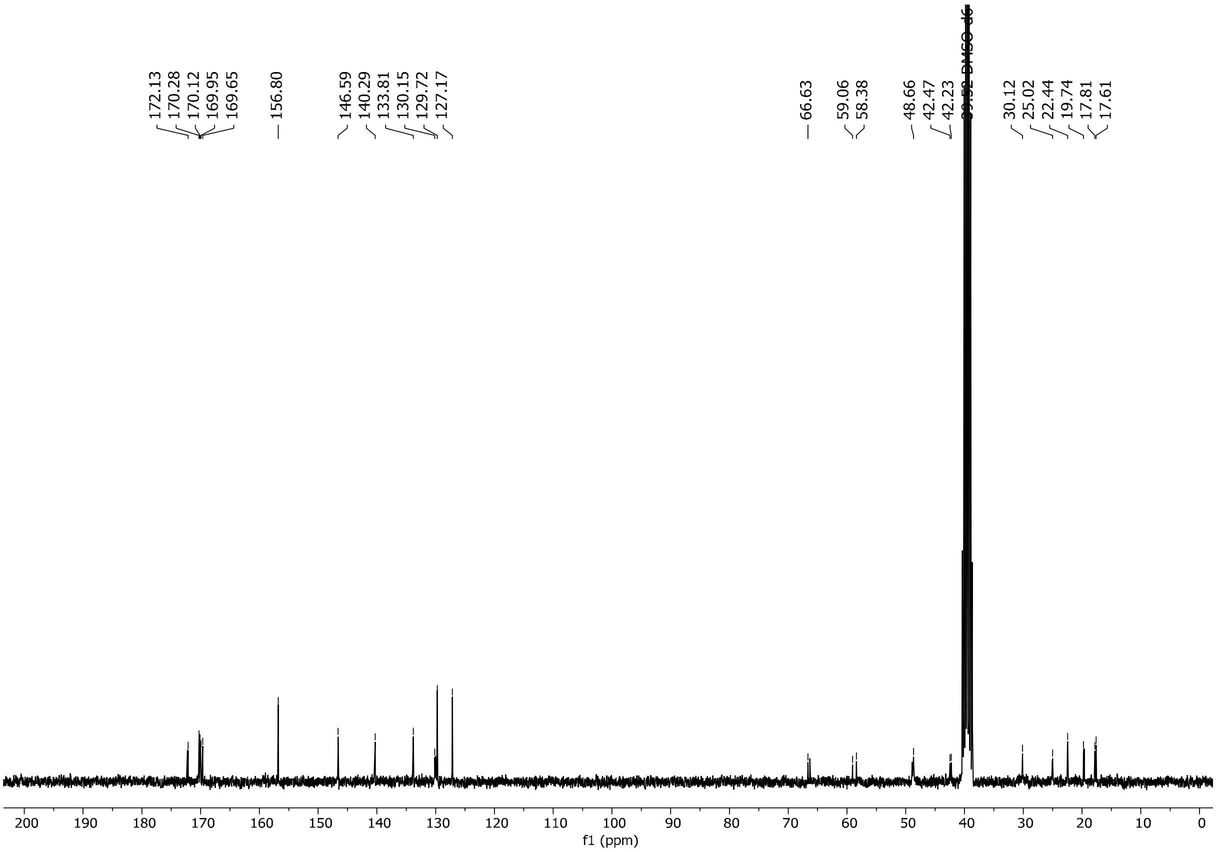

Figure S114. ^{13}C NMR of 106.

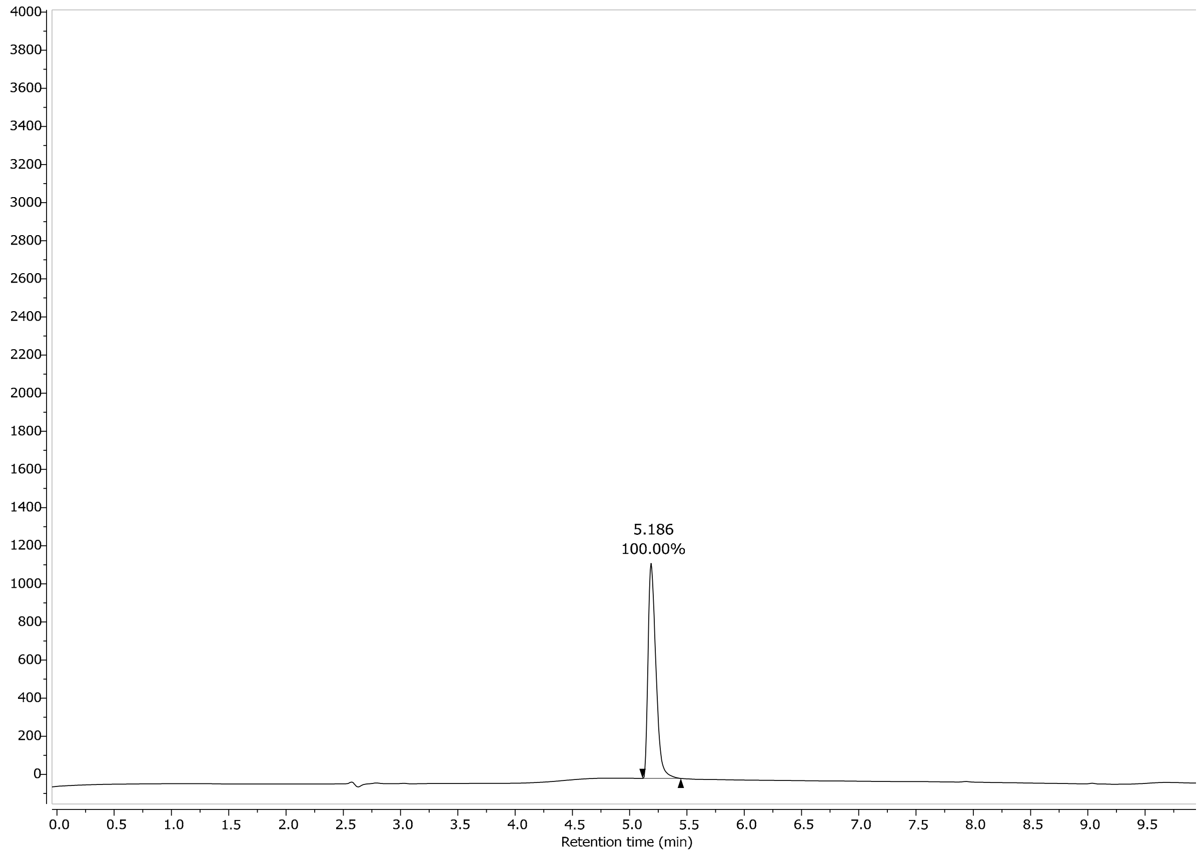

Figure S115. HPLC Chromatogram of 106 at 210 nm.

107, (2*S*,3*S*)-2-(2-acetamidoacetamido)-*N*-((*S*)-1-(((*S*,*E*)-1-fluoro-6-guanidino-1-(phenylsulfonyl)hex-1-en-3-yl)amino)-1-oxopropan-2-yl)-3-hydroxybutanamide

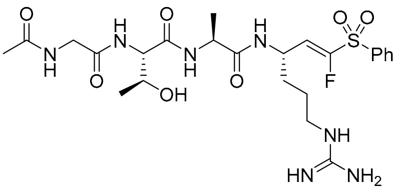

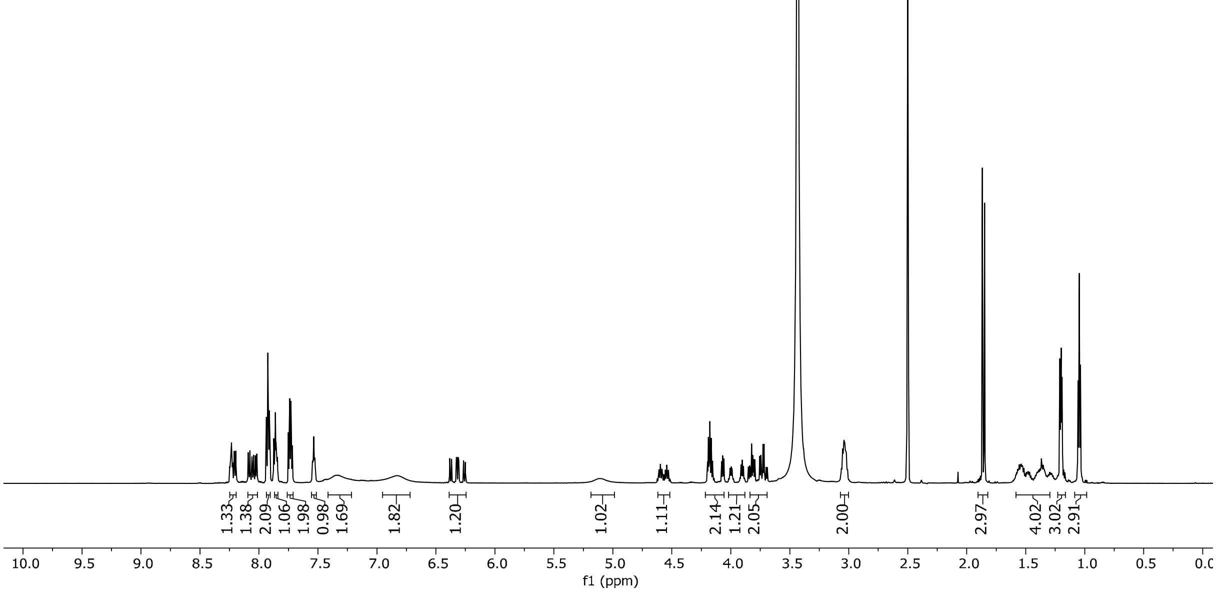

Figure S116. ^{1}H NMR of 107.

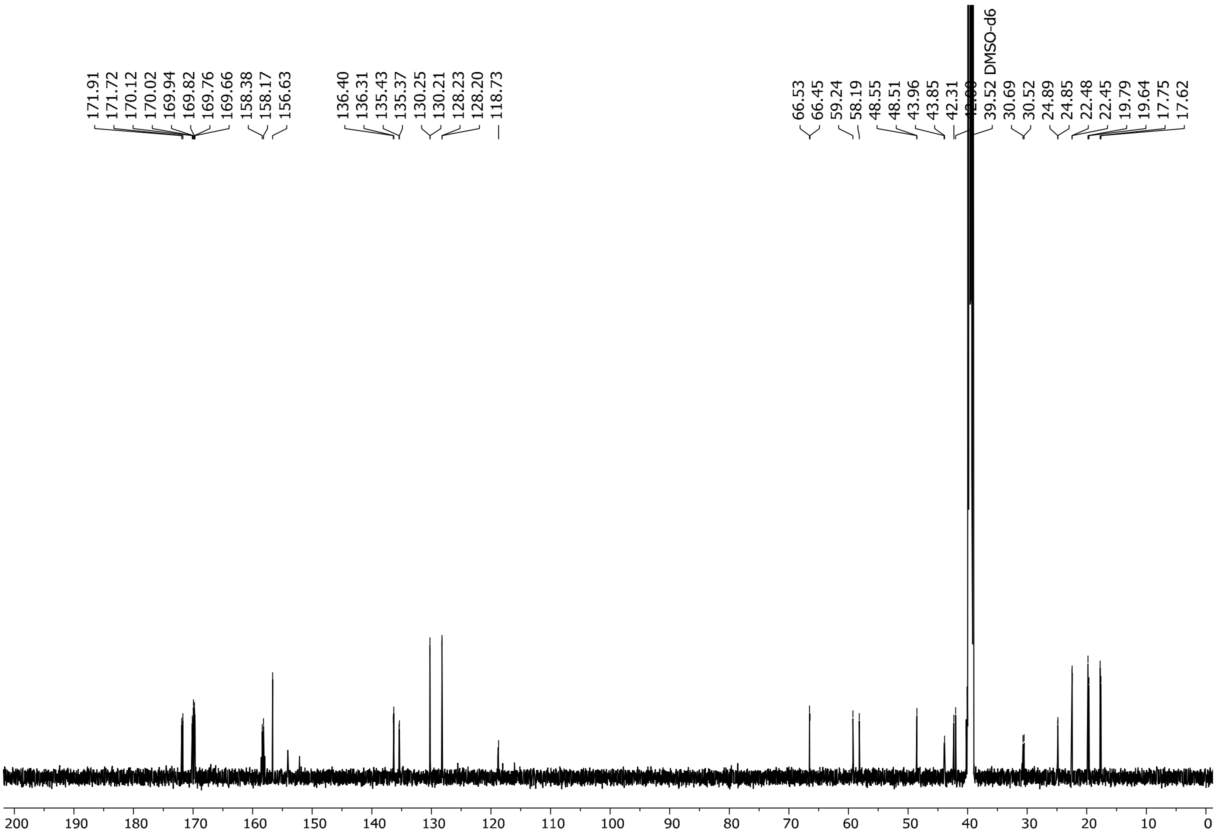

Figure S117. ^{13}C NMR of 107.

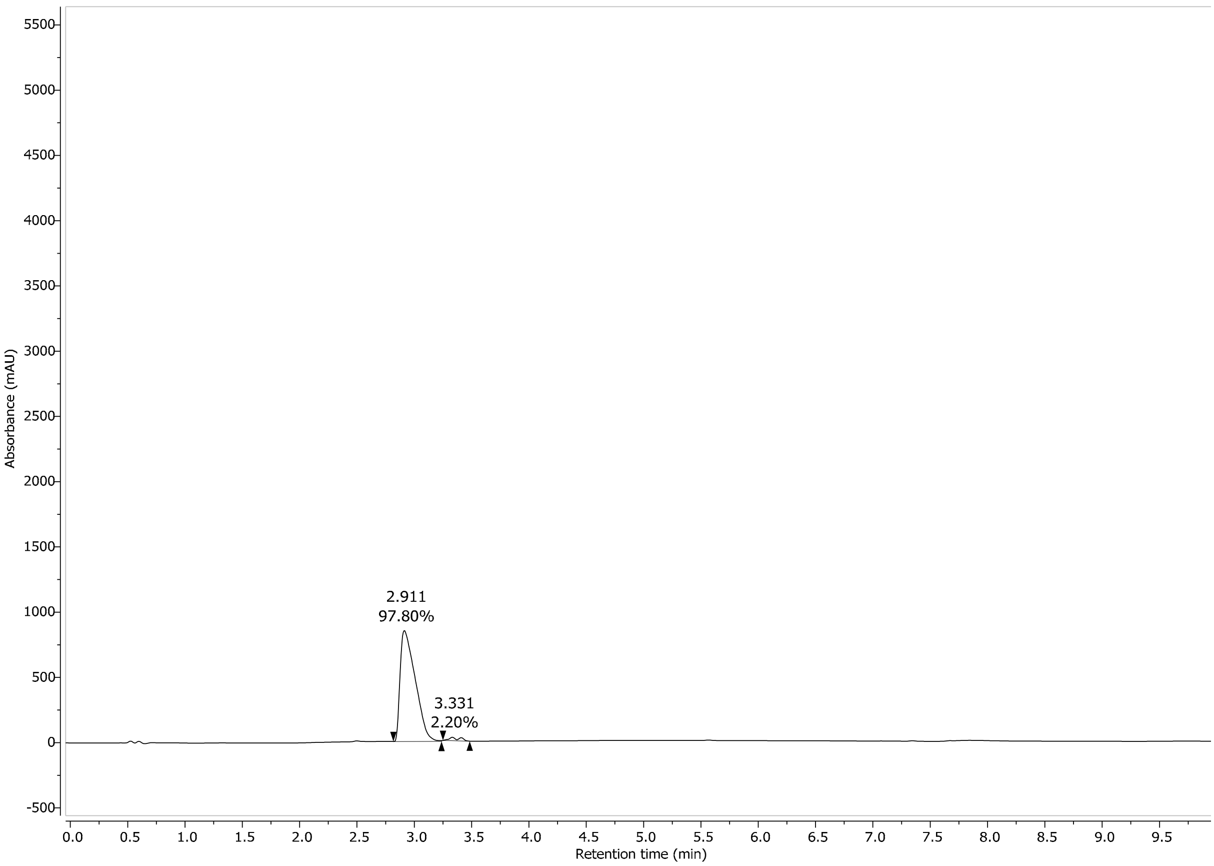

Figure S118. HPLC Chromatogram of 107 at 210 nm.

108, *tert*-Butyl ((2S)-4-methyl-1-oxo-1-((2-oxo-1-phenylazetidin-3-yl) amino) pentan-2-yl) carbamate

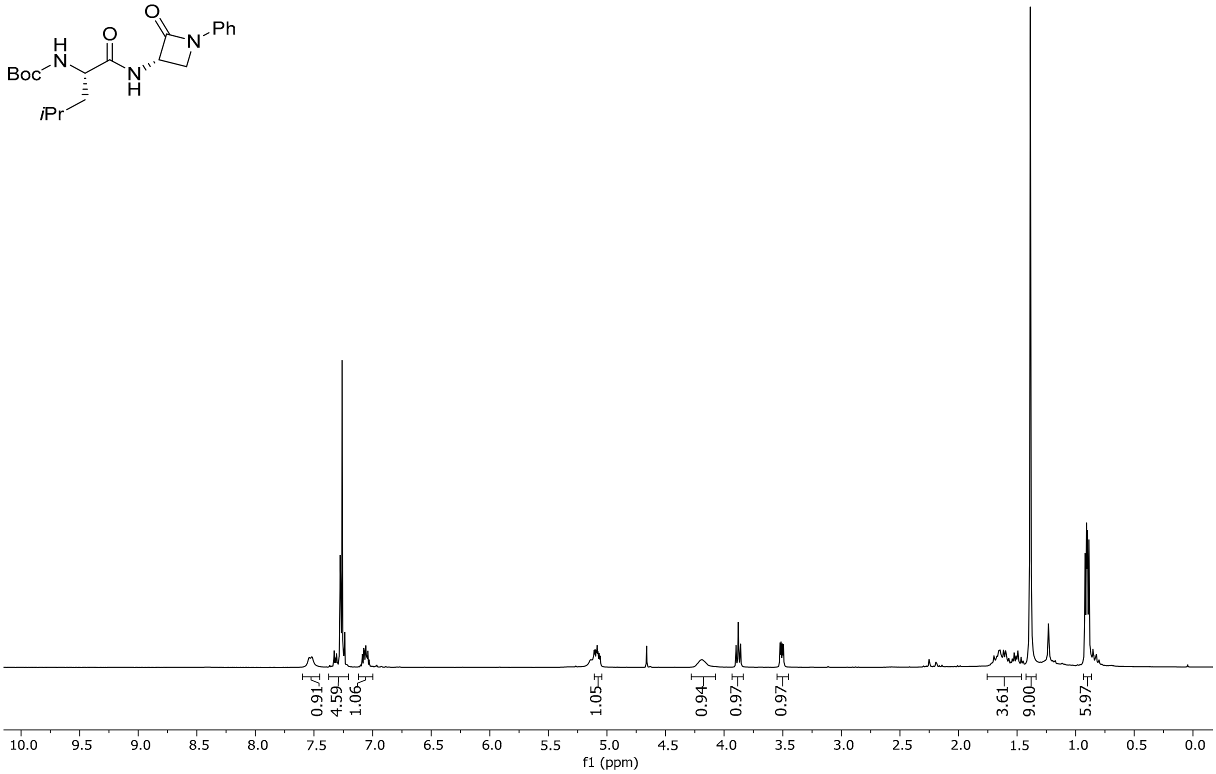

Figure S119. ^{1}H NMR of 108.

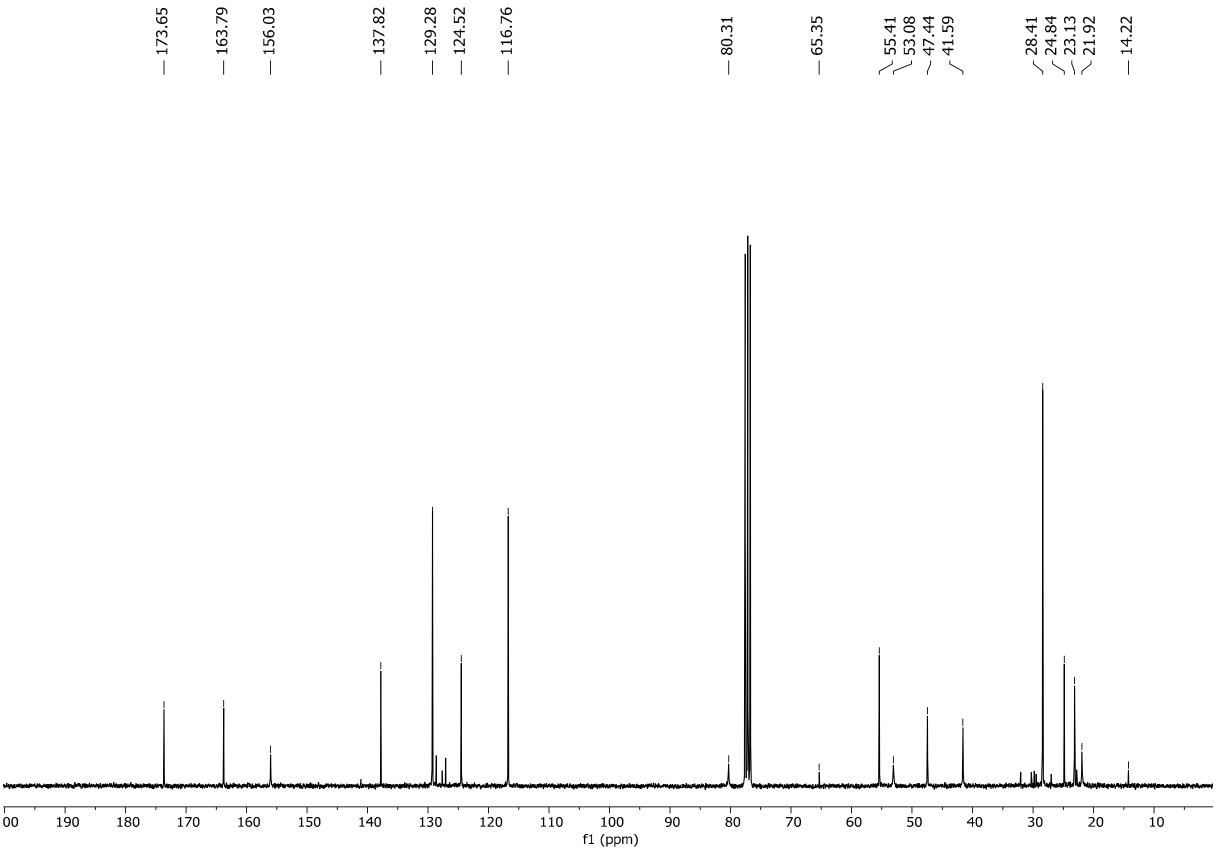

Figure S120. ^{13}C NMR of 108.

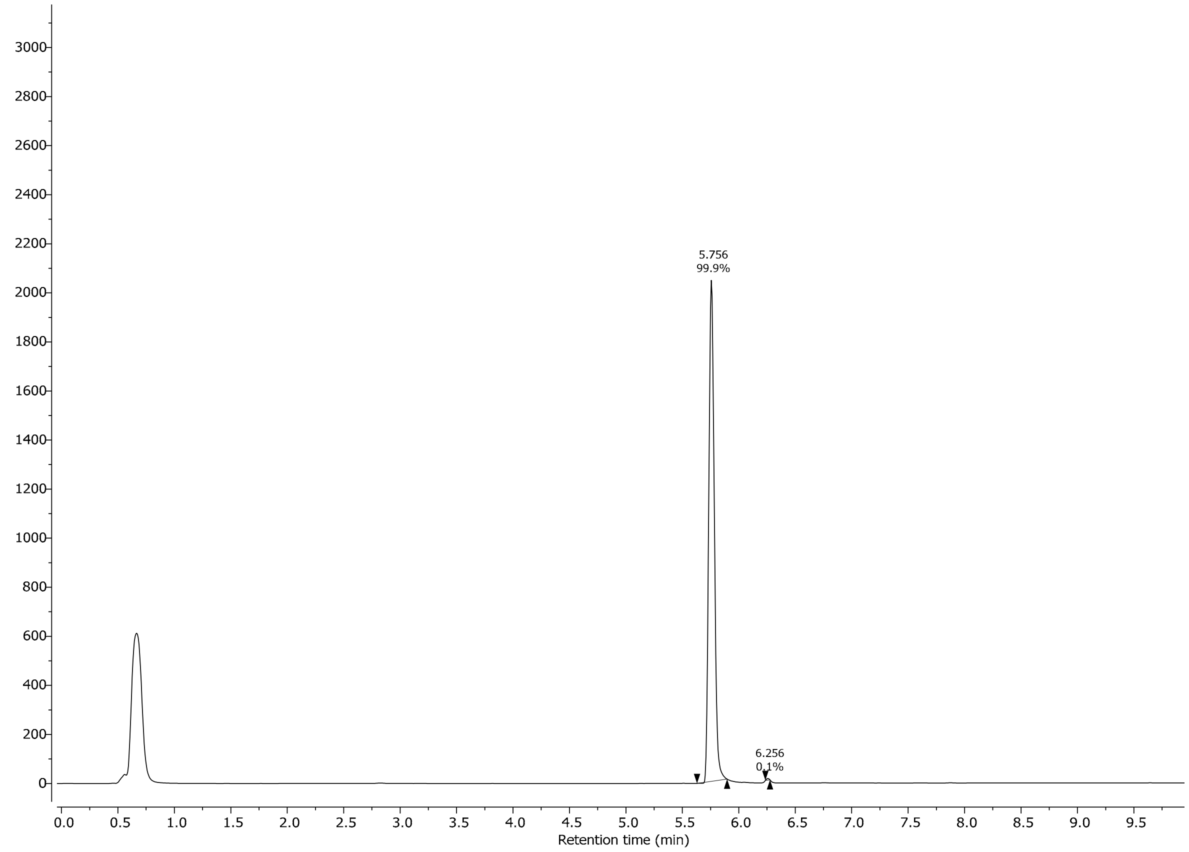

Figure S121. HPLC Chromatogram of 108 at 254 nm.

109, *tert*-Butyl (*S,E*)-(5-methyl-1-(phenylsulfonyl)hex-1-en-3-yl)carbamate

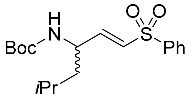

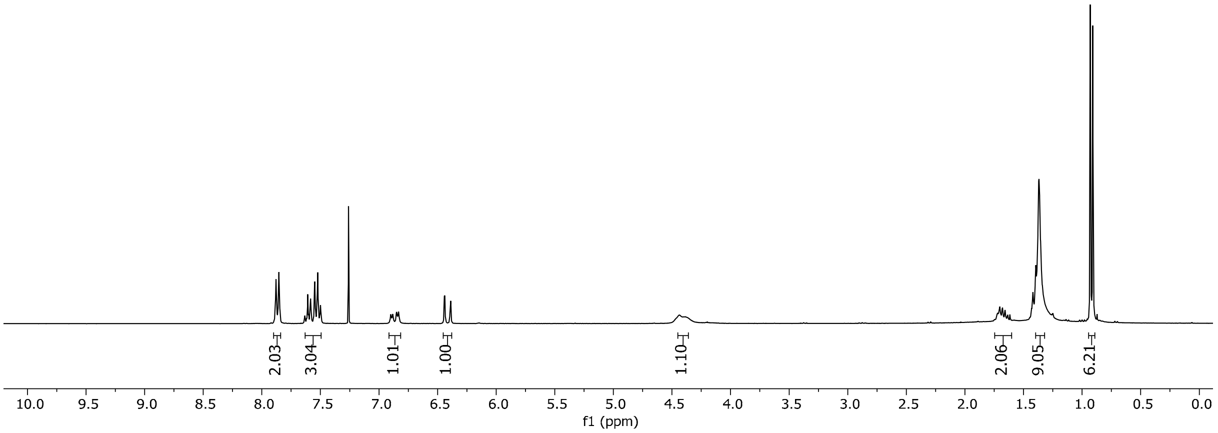

Figure S122. ^{1}H NMR of 109.

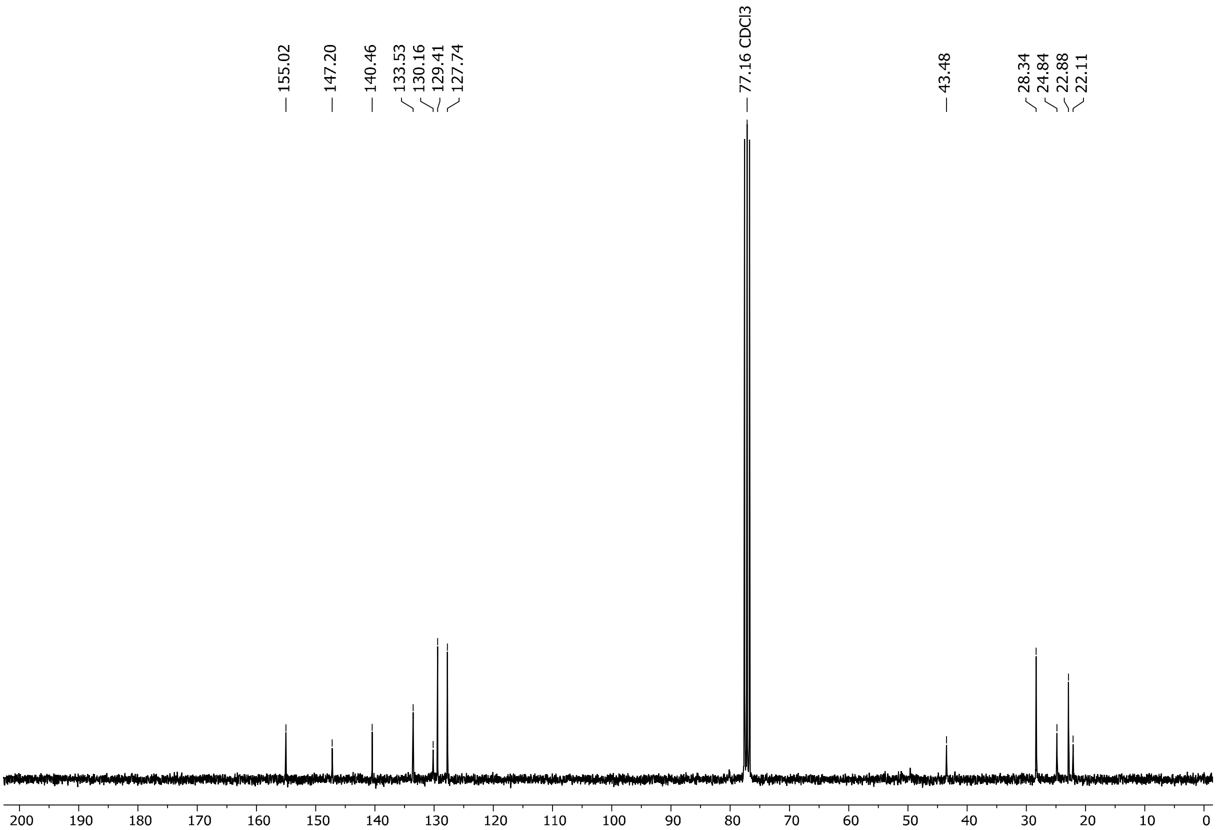

Figure S123. ^{13}C NMR of 109.

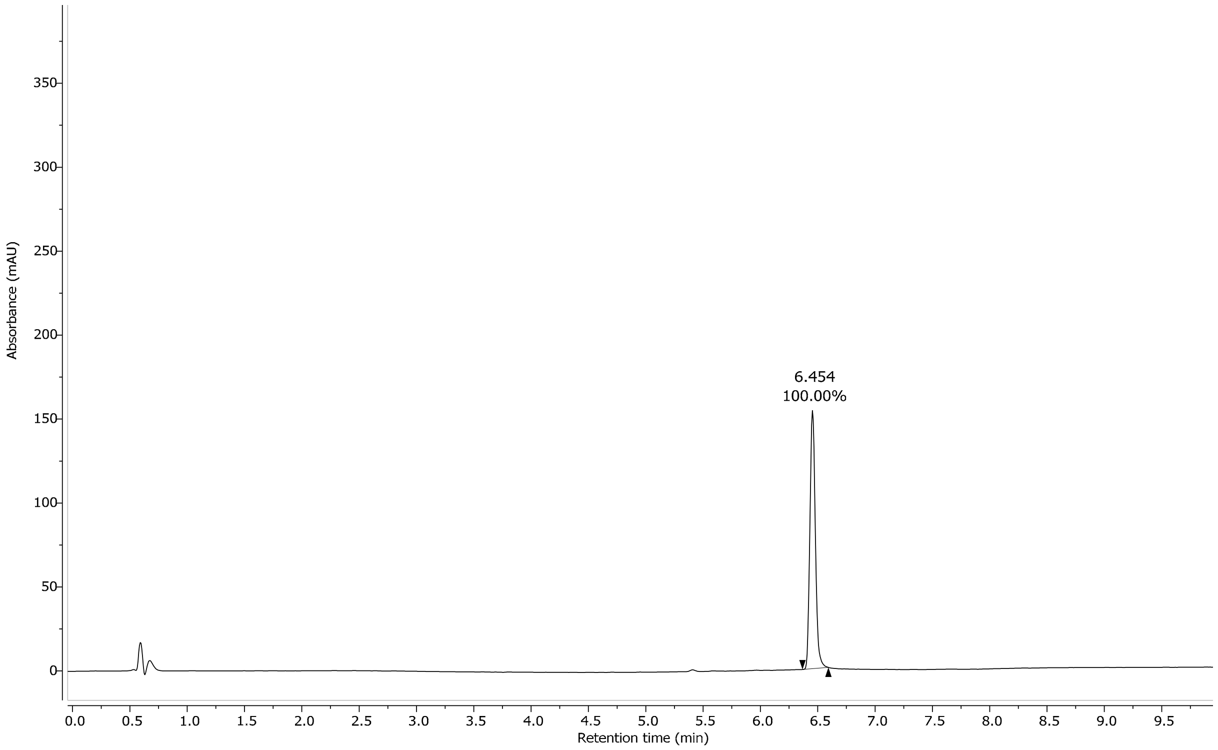

Figure S124. HPLC Chromatogram of 109 at 254 nm.

110, *tert*-Butyl (*S,E*)-(1-fluoro-5-methyl-1-(phenylsulfonyl)hex-1-en-3-yl)carbamate

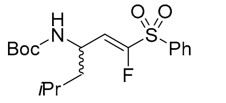

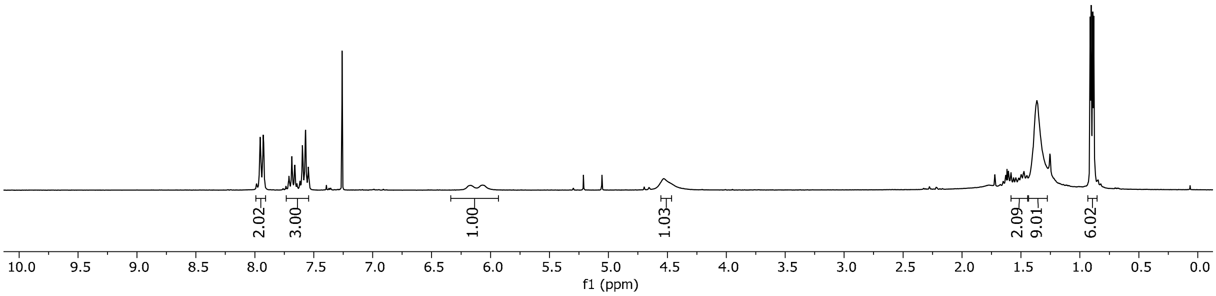

Figure S125. ^{1}H NMR of 110.

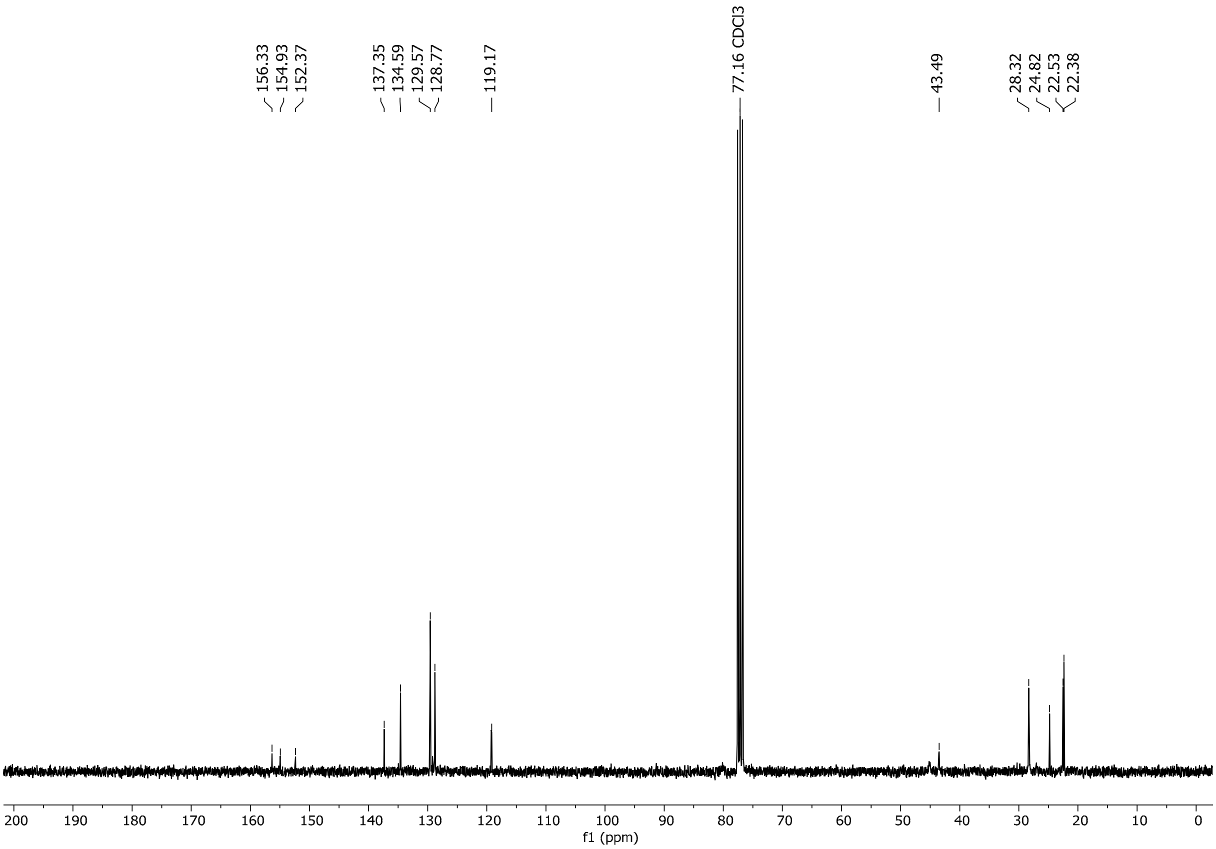

Figure S126. ^{13}C NMR of 110.

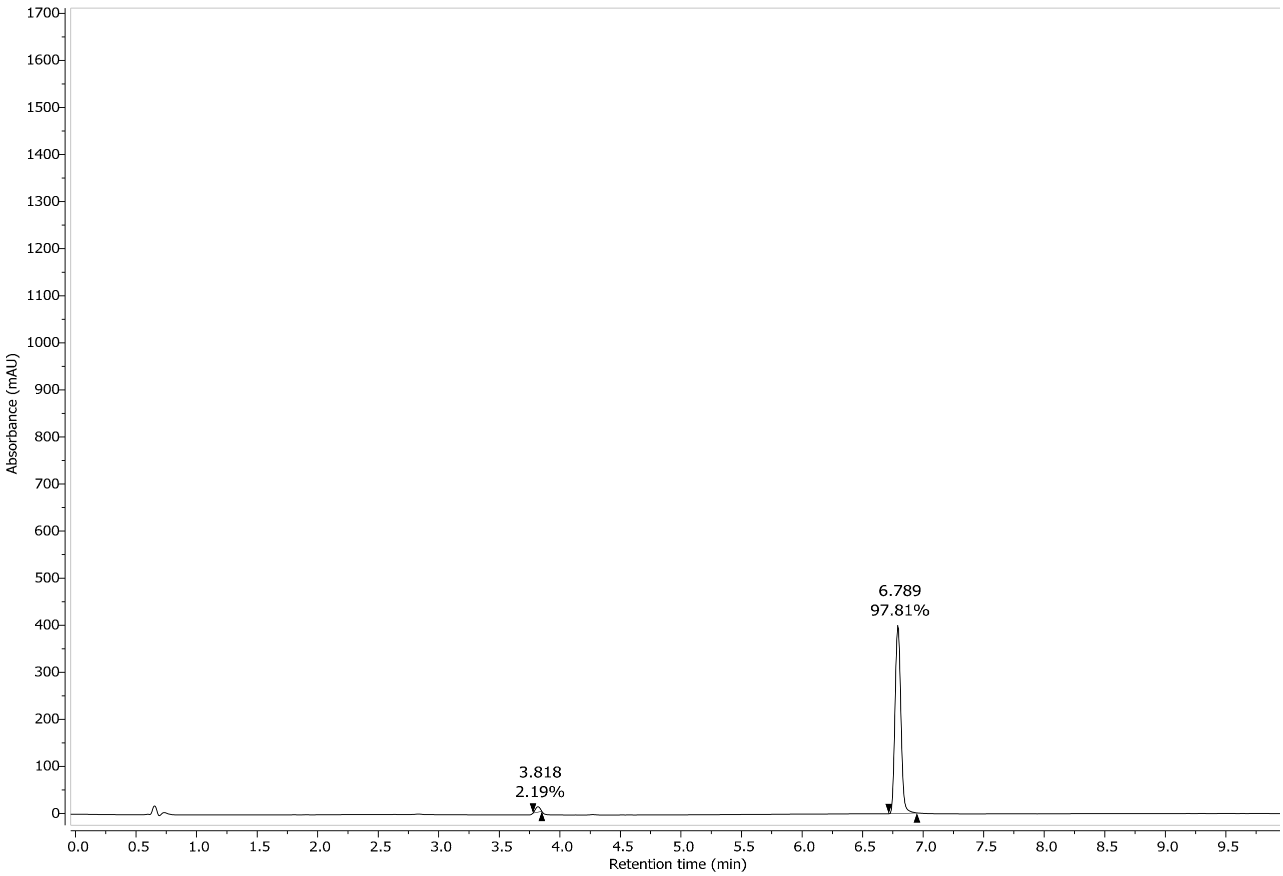

Figure S127. HPLC Chromatogram of 110 at 254 nm.

111, *tert*-Butyl ((3*S*)-2-hydroxy-5-methyl-1-nitrohexan-3-yl) carbamate

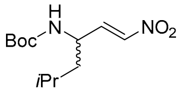

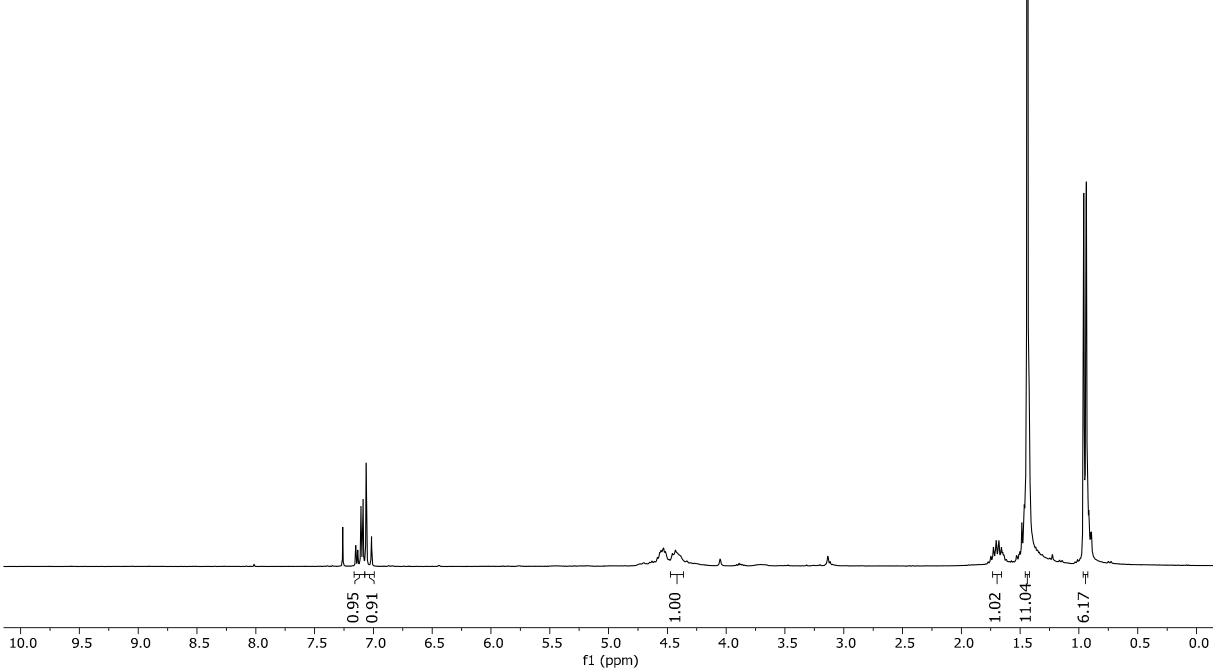

Figure S128. ^{1}H NMR of 111.

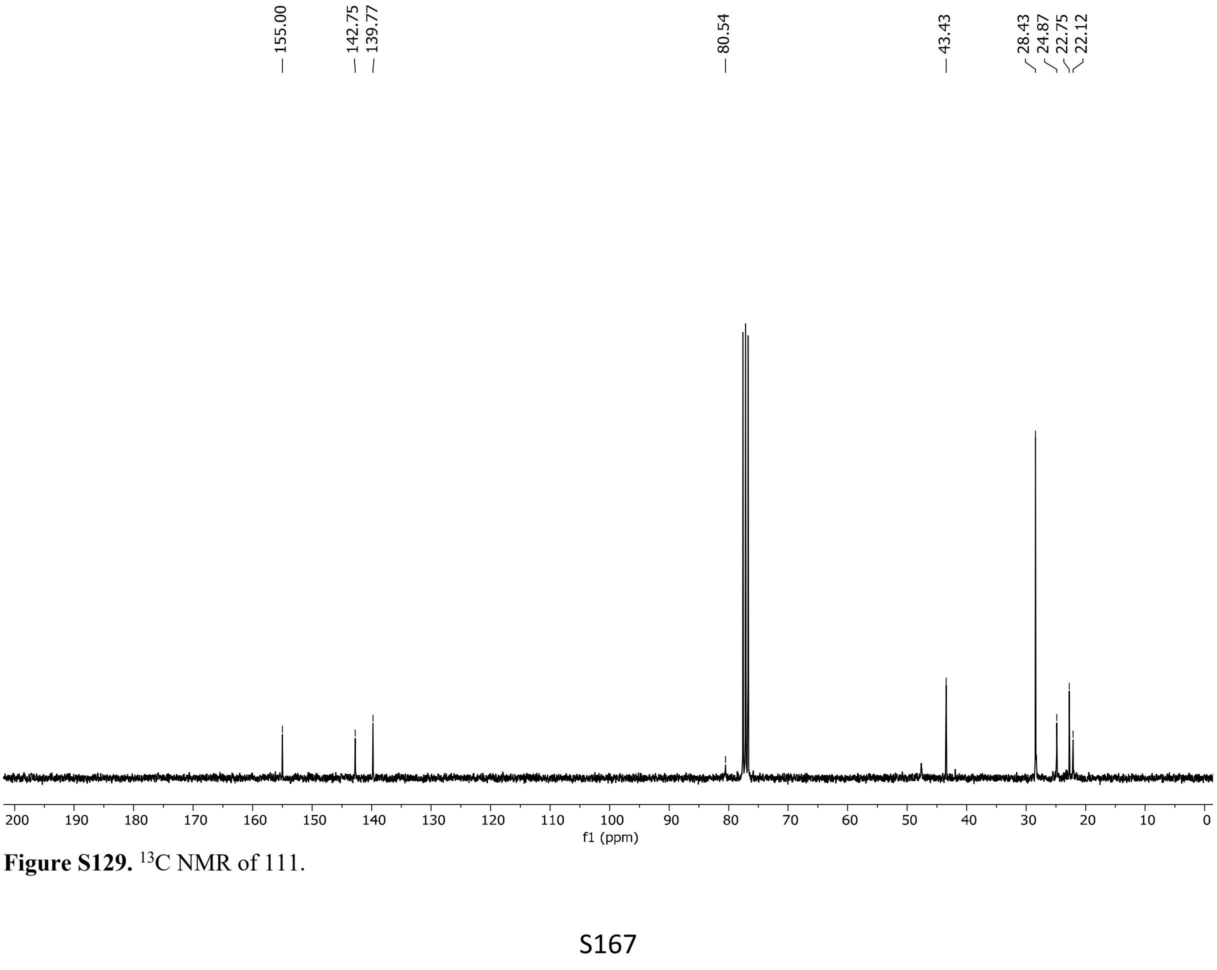

Figure S129. ^{13}C NMR of 111.

112, Ethyl (*S,E*)-5-((*tert*-butoxycarbonyl)amino)-7-methyl-4-oxooct-2-enoate

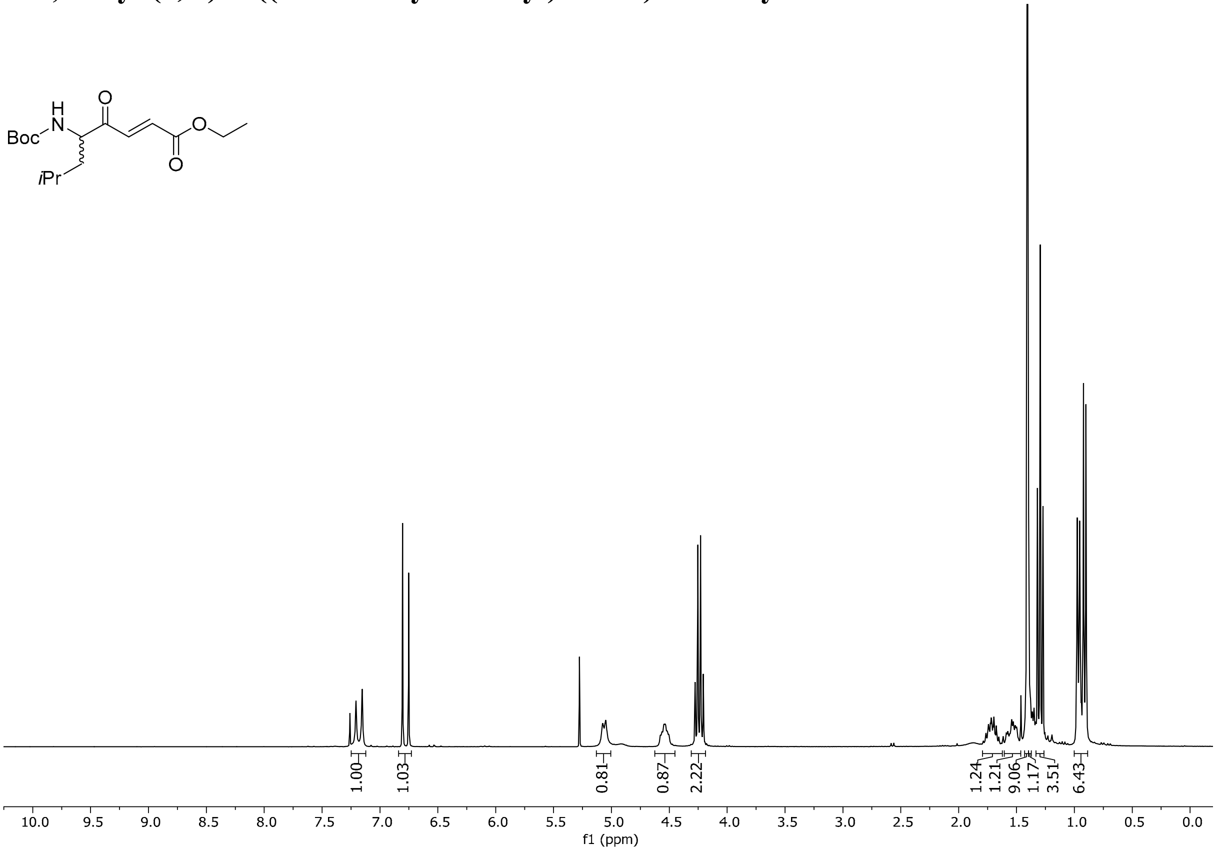

Figure S130. ^{1}H NMR of 112.

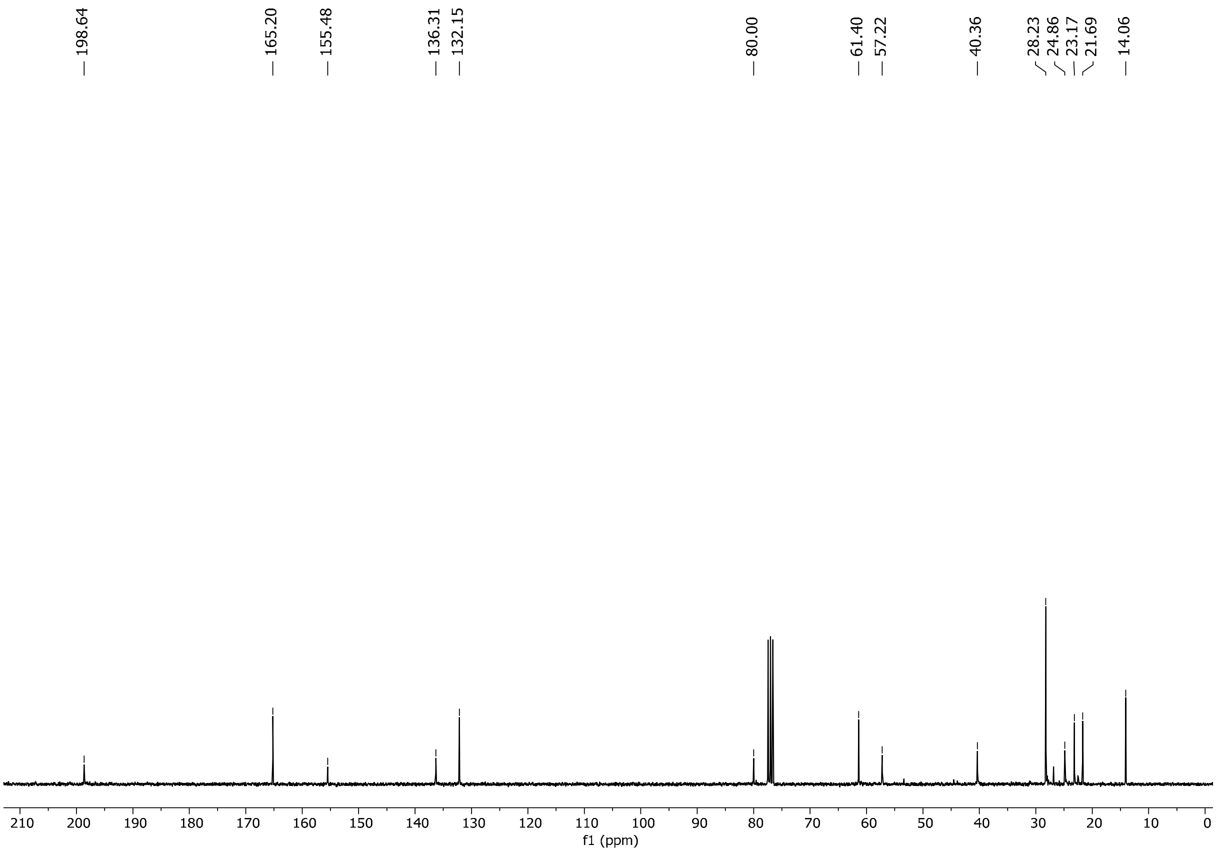

Figure S131. ^{13}C NMR of 112.

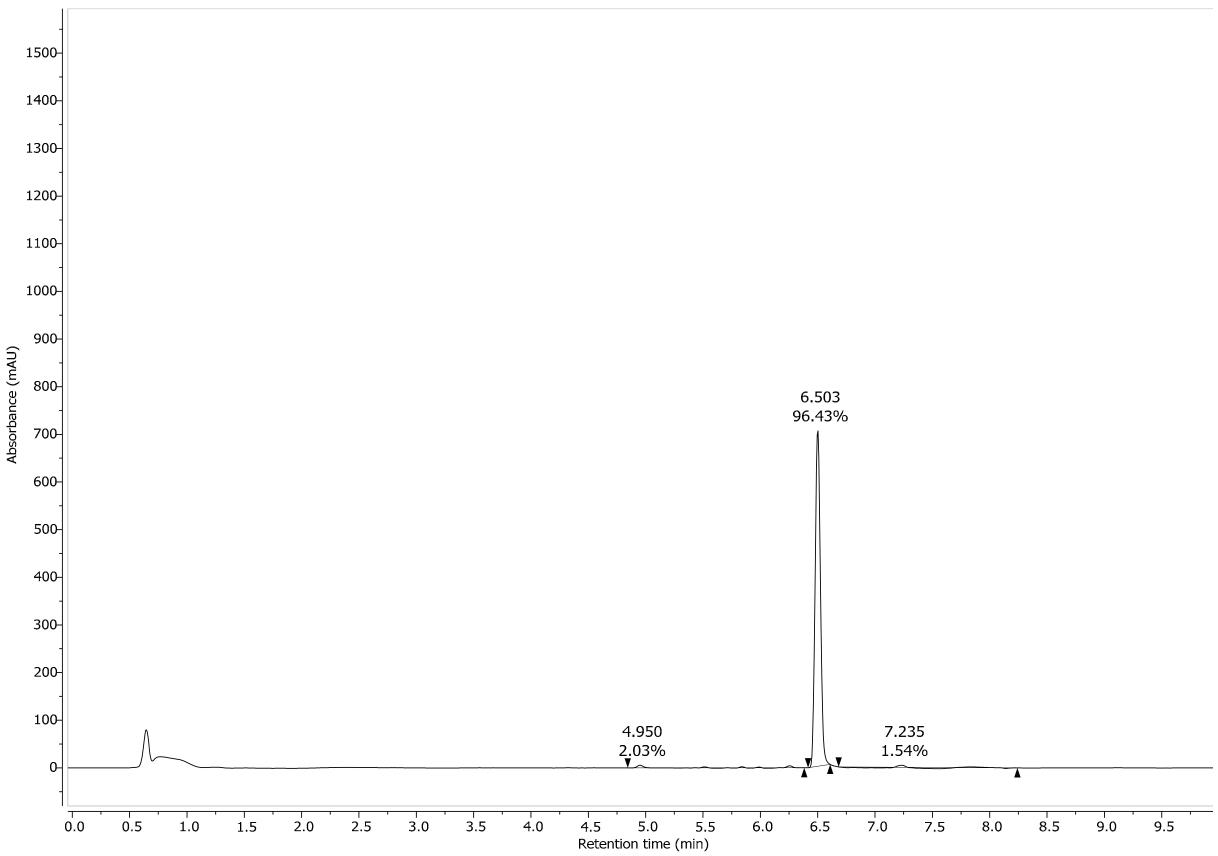

Figure S132. HPLC Chromatogram of 112 at 210 nm.

115, *tert*-Butyl (1-(benzo[*d*]thiazol-2-yl)-4-methyl-1-oxopentan-2-yl-1-^{13}C)carbamate

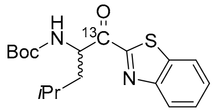

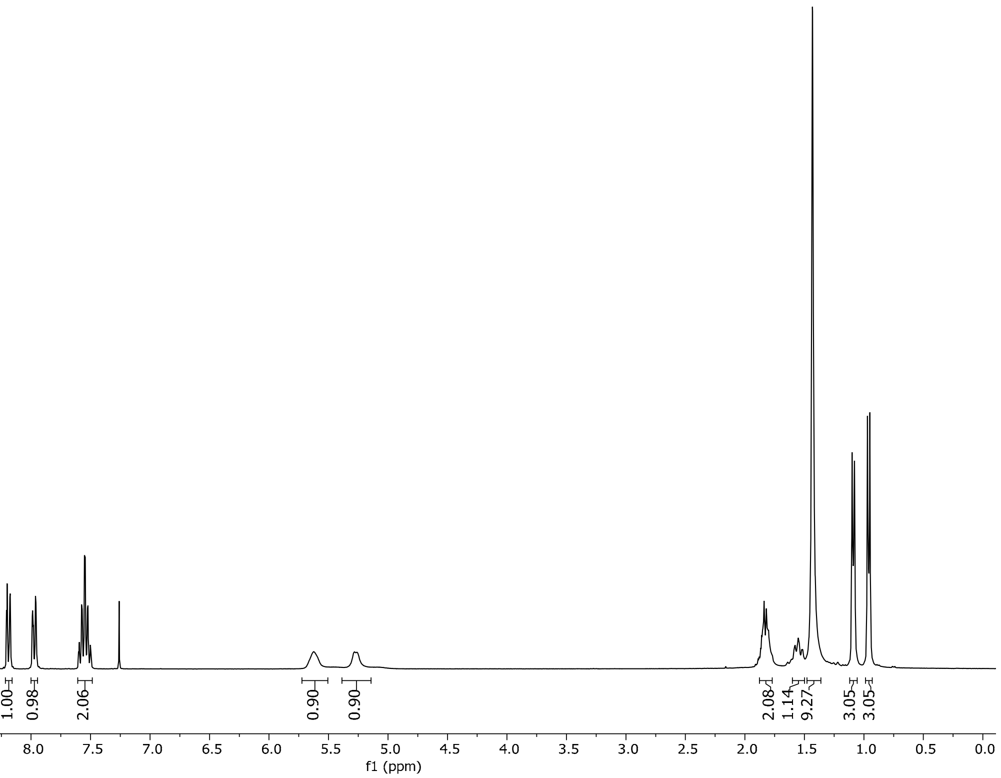

Figure S133. ^{1}H NMR of 115.

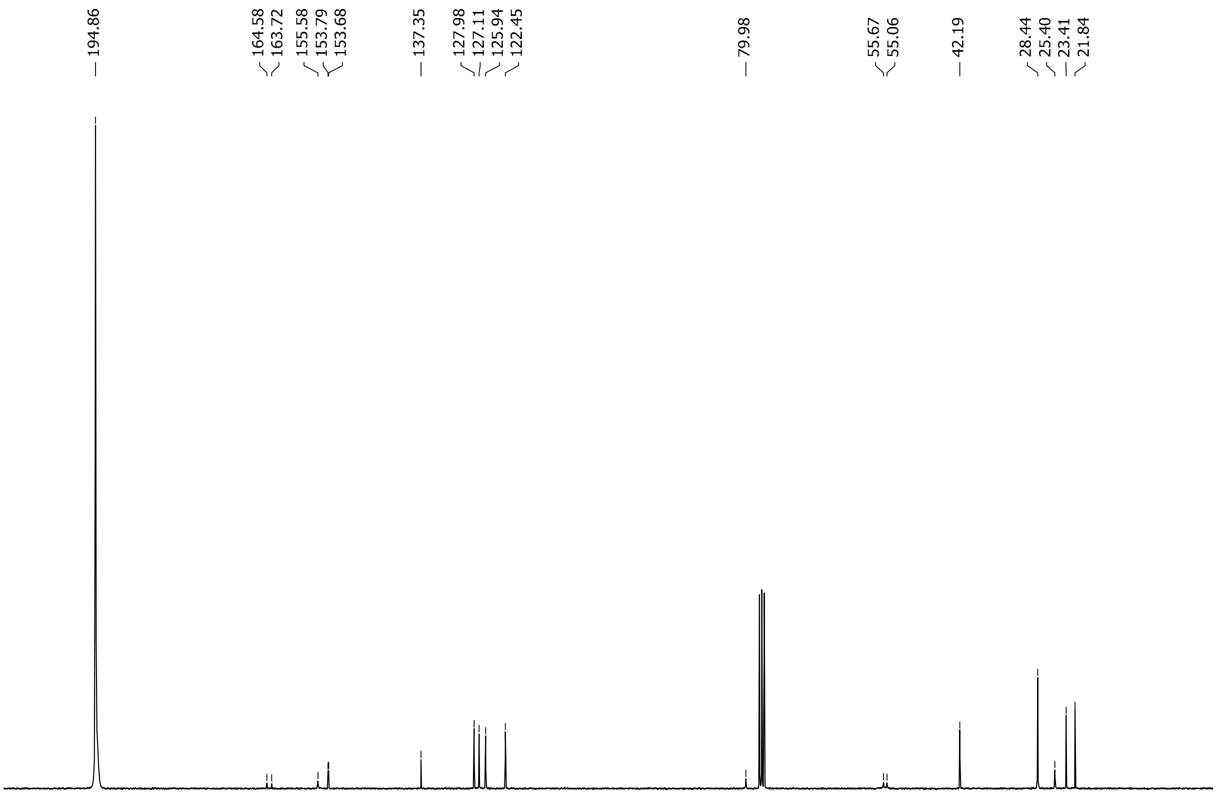

Figure S134. ^{13}C NMR of 115.

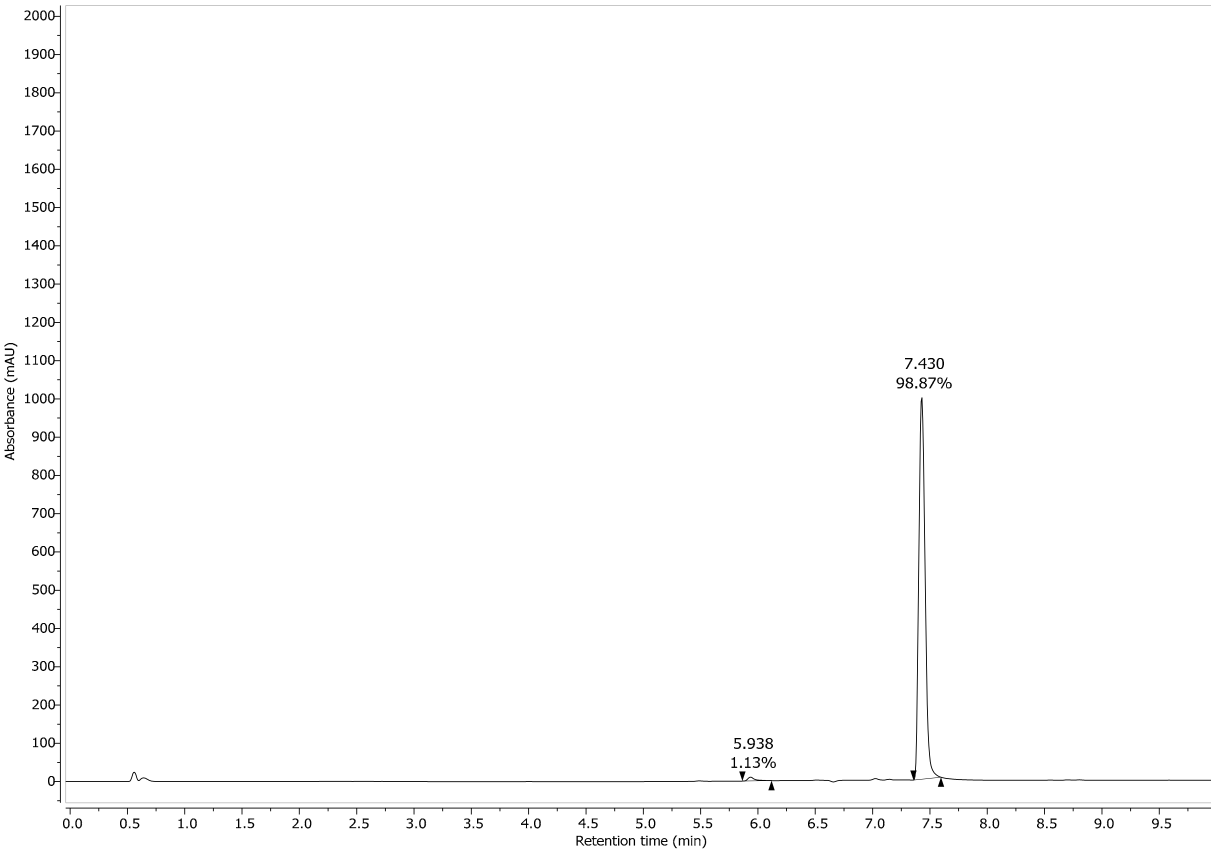

Figure S135. HPLC Chromatogram of 112 at 210 nm.

117, *tert*-Butyl (*S*)-(1-(cyano-^{13}C)-3-methylbutyl)carbamate

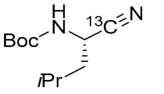

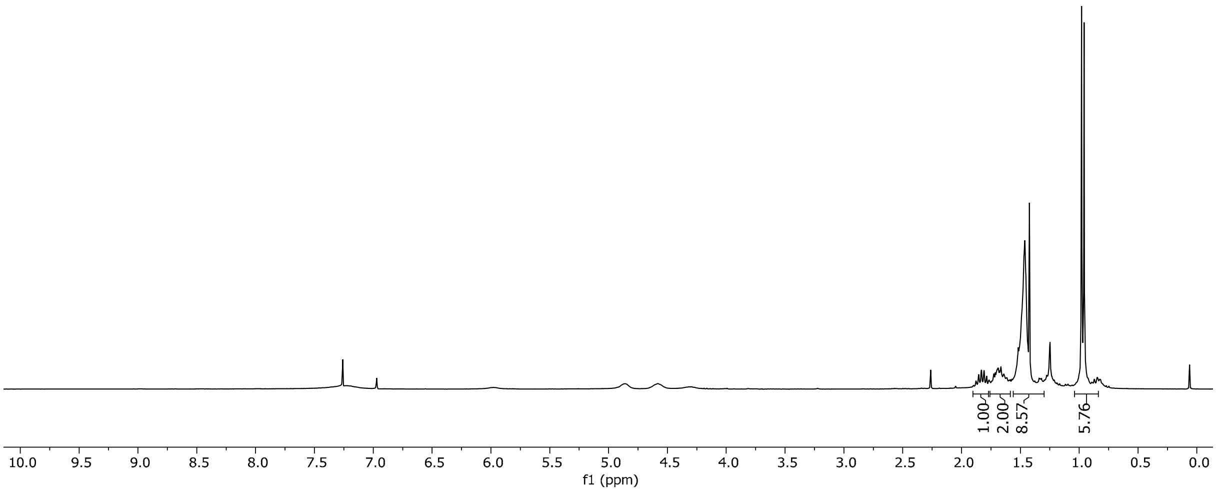

Figure S136. ^{1}H NMR of 117.

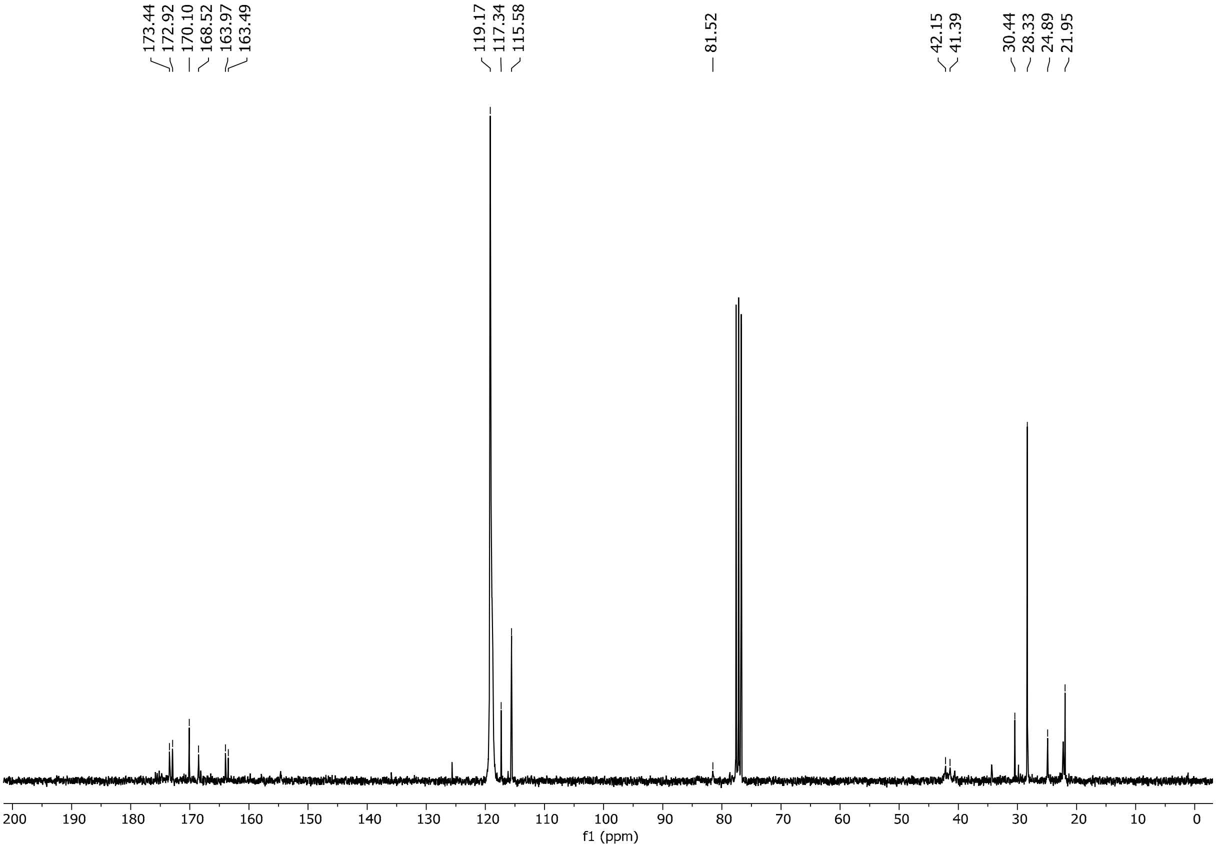

Figure S137. ^{13}C NMR of 117.

References

[1] S. Ludewig, M. Kossner, M. Schiller, K. Baumann, T. Schirmeister, *Curr. Top. Med. Chem.* **2010**, *10*, 368–382.

[2] F. Barthels, G. Marincola, T. Marciniak, M. Konhäuser, S. Hammerschmidt, J. Bierlmeier, U. Distler, P. R. Wich, S. Tenzer, D. Schwarzer, W. Ziebuhr, T. Schirmeister, *ChemMedChem* **2020**, *15*, 839–850.

[3] G. Amendola, R. Ettari, S. Previti, C. Di Chio, A. Messere, S. Di Maro, S. J. Hammerschmidt, C. Zimmer, R. A. Zimmermann, T. Schirmeister, M. Zappalà, S. Cosconati, *J. Chem. Inf. Model.* **2021**, *61*, 2062–2073.

[4] T. Schirmeister, J. Kesselring, S. Jung, T. H. Schneider, A. Weickert, J. Becker, W. Lee, D. Bamberger, P. R. Wich, U. Distler, S. Tenzer, P. Johé, U. A. Hellmich, B. Engels, *J. Am. Chem. Soc.* **2016**, *138*, 8332–8335.

[5] C. R. Caffrey, E. Hansell, K. D. Lucas, L. S. Brinen, A. Alvarez Hernandez, J. Cheng, S. L. Gwaltney, W. R. Roush, Y.-D. Stierhof, M. Bogyo, D. Steverding, J. H. McKerrow, *Mol. Biochem. Parasitol.* **2001**, *118*, 61–73.

[6] H. M. Berman, *Nucleic Acids Res.* **2000**, *28*, 235–242.

[7] I. D. Kerr, J. H. Lee, C. J. Farady, R. Marion, M. Rickert, M. Sajid, K. C. Pandey, C. R. Caffrey, J. Legac, E. Hansell, J. H. McKerrow, C. S. Craik, P. J. Rosenthal, L. S. Brinen, *J. Biol. Chem.* **2009**, *284*, 25697–25703.

[8] Y. D. Ward, D. S. Thomson, L. L. Frye, C. L. Cywin, T. Morwick, M. J. Emmanuel, R. Zindell, D. McNeil, Y. Bekkali, Marc Girardot, M. Hrapchak, M. DeTuri, K. Crane, D. White, S. Pav, Y. Wang, M.-H. Hao, C. A. Grygon, M. E. Labadia, D. M. Freeman, W. Davidson, J. L. Hopkins, M. L. Brown, D. M. Spero, *J. Med. Chem.* **2002**, *45*, 5471–5482.

[9] E. Zeslawska, U. Jacob, A. Schweinitz, G. Coombs, W. Bode, E. Madison, *J. Mol. Biol.* **2003**, *328*, 109–118.

[10] H. Berman, K. Henrick, H. Nakamura, *Nat. Struct. Mol. Biol.* **2003**, *10*, 980–980.

[11] J. Schrader, F. Henneberg, R. A. Mata, K. Tittmann, T. R. Schneider, H. Stark, G. Bourenkov, A. Chari, *Science (80-.).* **2016**, *353*, 594–598.

[12] Molecular Operating Environment (MOE), 2020.09, Chemical Computing Group ULC, **2020**.

[13] T. A. Halgren, *J. Comput. Chem.* **1999**, *20*, 720–729.

[14] LeadIT/FlexX, Version 2.3.2; GmbH, BioSolveIT, **2017**.

[15] The PyMOL Molecular Graphics System, 2.5.2, Schrödinger, LLC.

[16] I. Reulecke, G. Lange, J. Albrecht, R. Klein, M. Rarey, *ChemMedChem* **2008**, *3*, 885–897.

[17] J. B. F. and D. J. F. M. J. Frisch, G. W. Trucks, H. B. Schlegel, G. E. Scuseria, M. A. Robb, J. R. Cheeseman, G. Scalmani, V. Barone, G. A. Peterson, H. Nakatsuji, X. Li, M. Caricato, A. V. Marenich, J. Bloino, B. G. Janesko, R. Gomperts, B. Mennucci, H. P. Hratchian, J. V. O, **n.d.**

[18] J.-D. Chai, M. Head-Gordon, *Phys. Chem. Chem. Phys.* **2008**, *10*, 6615.

[19] A. V. Marenich, C. J. Cramer, D. G. Truhlar, *J. Phys. Chem. B* **2009**, *113*, 6378–6396.

[20] J. R. Pliego Jr, J. M. Riveros, *Phys. Chem. Chem. Phys.* **2002**, *4*, 1622–1627.

[21] A. Ben-Naim, *J. Phys. Chem.* **1978**, *82*, 792–803.

[22] T. Dražić, S. Kopf, J. Corridan, M. M. Leuthold, B. Bertoša, C. D. Klein, *J. Med. Chem.* **2020**, *63*, 140–156.

[23] R. Spina, E. Colacino, J. Martinez, F. Lamaty, *Chem. - A Eur. J.* **2013**, *19*, 3817–3821.

[24] A. Ho, K. Cyrus, K.-B. Kim, *European J. Org. Chem.* **2005**, *2005*, 4829–4834.

[25] W. Vuong, J. C. Vederas, *J. Org. Chem.* **2021**, *86*, 13104–13110.

[26] Q. Tian, N. K. Nayyar, S. Babu, L. Chen, J. Tao, S. Lee, A. Tibbetts, T. Moran, J. Liou, M. Guo, T. P. Kennedy, *Tetrahedron Lett.* **2001**, *42*, 6807–6809.

[27] C. St-Georges, A. Désilets, F. Béliveau, M. Ghinet, S. P. Dion, É. Colombo, P.-L. Boudreault, R. J. Najmanovich, R. Leduc, É. Marsault, *Eur. J. Med. Chem.* **2017**, *129*, 110–123.

[28] M. J. Costanzo, S. C. Yabut, H. R. Almond, P. Andrade-Gordon, T. W. Corcoran, L. de Garavilla, J. A. Kauffman, W. M. Abraham, R. Recacha, D. Chattopadhyay, B. E. Maryanoff, *J. Med. Chem.* **2003**, *46*, 3865–3876.

[29] J. Engel-Andreasen, I. Wellhöfer, K. Wich, C. A. Olsen, *J. Org. Chem.* **2017**, *82*, 11613–11619.

[30] F. E. Dutton, B. H. Lee, S. S. Johnson, E. M. Coscarelli, P. H. Lee, *J. Med. Chem.* **2003**, *46*, 2057–2073.
